# Comparative transcriptomic and physiological analyses of weedy rice and cultivated rice to identify vital differentially expressed genes and pathways regulating the ABA response

**DOI:** 10.1038/s41598-021-92504-5

**Published:** 2021-06-18

**Authors:** Hong Lang, Yuting He, Faliang Zeng, Fan Xu, Minghui Zhao, Dianrong Ma

**Affiliations:** Key Laboratory of Northern Japonica Rice Genetics and Breeding, Ministry of Education and Liaoning Province, Key Laboratory of Northeast Rice Biology and Genetics and Breeding, Ministry of Agriculture, Rice Research Institute of Shenyang Agricultural University, Shenyang, 110866 China

**Keywords:** Gene expression analysis, Gene expression profiling, Developmental biology, Physiology, Plant sciences

## Abstract

Weedy rice is a valuable germplasm resource characterized by its high tolerance to both abiotic and biotic stresses. Abscisic acid (ABA) serves as a regulatory signal in plant cells as part of their adaptive response to stress. However, a global understanding of the response of weedy rice to ABA remains to be elucidated. In the present study, the sensitivity to ABA of weedy rice (WR04-6) was compared with that of *temperate japonica* Shennong9816 (SN9816) in terms of seed germination and post-germination growth via the application of exogenous ABA and diniconazole, an inhibitor of ABA catabolism. Physiological analysis and a transcriptomic comparison allowed elucidation of the molecular and physiological mechanisms associated with continuous ABA and diniconazole treatment. WR04-6 was found to display higher ABA sensitivity than SN9816, resulting in the rapid promotion of antioxidant enzyme activity. Comparative transcriptomic analyses indicated that the number of differentially expressed genes (DEGs) in WR04-6 seedlings treated with 2 μM ABA or 10 μM diniconazole was greater than that in SN9816 seedlings. Genes involved in stress defense, hormone signal transduction, and glycolytic and citrate cycle pathways were highly expressed in WR04-6 in response to ABA and diniconazole. These findings provide new insight into key processes mediating the ABA response between weedy and cultivated rice.

## Introduction

Weedy rice (*Oryza. sativa* f. *spontanea*) is taxonomically classified as the same species as cultivated rice (*Oryza. sativa* L.)^1^ and is among the most persistent and problematic weeds found in paddy fields worldwide. The phenotypic characteristics of weedy rice are highly variable and appear to be intermediate between wild rice and cultivated rice, retaining the characteristics of both the wild relatives (black hull, long awn, red pericarp, and seed shattering) and the developed crop mimicries (plant height and heading time), enhancing their adaptation and competitiveness in the agroecosystem^2^. Weedy rice is considered a valuable germplasm resource because of its enhanced tolerance to deep sowing^3^, higher nitrogen use efficiency^4^, greater photosynthetic activity^5^, and resistance to blast disease^6^ compared with cultivated rice. Furthermore, weedy rice displays significant versatility in its adaption to a wide range of adverse conditions. It has many useful stress-related genes that respond to biotic and abiotic stresses^7^. There are weedy rice genotypes that tolerate short-term low temperature^8,9^, high salinity, and drought stress^10^ at the seedling stage owing to excellent stress adaptation mechanisms. Crop domestication and improvement have dramatically reduced genetic variability by 50–60% in comparison with ancestral wild species^11^, therefore, weedy rice provides the possibility to widen the gene pool of cultivated rice and breed cultivars with improved yield, quality, and stress tolerance.

To adapt and survive in adverse conditions, plants have evolved and established mechanisms that are plastic at multiple levels, including molecular, physiological, and developmental levels, which permit them to cope with unfavorable environments^12,13^. Abscisic acid (ABA) is generally considered a stress hormone, and its biosynthesis plays a role in rapid metabolic adjustment in response to stress^14^. The expression of stress-inducible genes in plants is primarily regulated by both ABA-dependent and ABA-independent pathways^15^. When plants encounter stress conditions, endogenous ABA rapidly accumulates. ABA receptors (PYR/RCARs) sense ABA and bind to subclass A protein type 2C phosphatases (PP2Cs), resulting in the activation of SNF-related protein kinases (SnRK2s)^16^. Activated SnRK2s are able to phosphorylate ABRE binding factor (ABF) transcription factors, which in turn promote the expression of ABA-dependent genes^17^. The ABRE-binding proteins/AREB-binding factors (AREB/ABFs) transcription factors belong to an ABA-responsive gene family. Of these, *AREB1*/*ABF2*, *AREB2*/*ABF4*, and *ABF3* are induced by dehydration, high salinity, or ABA treatment during the vegetative stage^18^, and overexpression of *AREB*/*ABFs* in transgenic *Arabidopsis* displays enhanced drought tolerance via an ABA-dependent pathway^19^. Additional signaling factors associated with the principal ABA signaling pathway have also been identified. Stress-inducible SlMYB102 and GhMYB73 belonging to the R2R3 MYB family enhance salt tolerance in transgenic tomatoes and *Arabidopsis*, respectively^20,21^. Dehydration-responsive element-binding (DREB) transcription factors play a crucial role in plant growth, development, and stress responses. They function as components of both ABA-dependent and ABA-independent pathways in response to abiotic stress^22^. In rice, *DREB1A*, *DREB1B*, and *DREB1C* regulate plant cold tolerance by recognizing the GCC box of the target gene^23^. *DREB2A* and *DREB2B* become highly induced by dehydration and salinity, and overexpression of soybean *GmDREB2* in *Arabidopsis* enhances tolerance to salinity^24^.

ABA catabolism is an alternative method of regulating ABA levels. ABA is primarily catabolized to form 8ʹ-hydroxy ABA through hydroxylation by ABA 8ʹ-hydroxylase and final rearrangement to phaseic acid (PA)^25^. ABA 8′-hydroxylase is presumed to be a rate-limiting enzyme in the ABA catabolic pathway. Studies have shown that ABA catabolism under stress and recovery is principally regulated at the transcriptional level of the ABA 8ʹ-hydroxylase gene. In *Arabidopsis*, there are four genes (*AtCYP707A1-4*) that encode ABA 8ʹ-hydroxylase. The expression of the four *CYP707As* is induced by dehydration stress and subsequent rehydration^26^. Of the three ABA 8ʹ-hydroxylase genes (*OsABA8ox1*-*3*) in rice, the expression of *OsABA8ox1* is induced by cold stress^27^. Overall, ABA 8ʹ-hydroxylase plays an important role in controlling ABA homeostasis when experiencing abiotic stress.

The regulation of ABA in abiotic stress in plants has been extensively studied, while ABA-associated regulatory networks in response to the accumulation of ABA in weedy rice remain elusive. In the present study, the sensitivity of weedy and cultivated rice to ABA during seed germination and post-germination growth was compared following the administration of exogenous ABA and diniconazole, an inhibitor of ABA catabolism. Physiological analysis was combined with a comparative transcriptomic investigation to elucidate the molecular and physiological mechanisms associated with continuous ABA or diniconazole treatment. The present study aimed to identify differentially expressed genes and pathways regulating the ABA response and provide deeper insights into the regulatory networks of ABA in weedy rice that could provide information for further crop improvements.

## Results

### Variation in ABA sensitivity during seed germination

**Figure 1:**
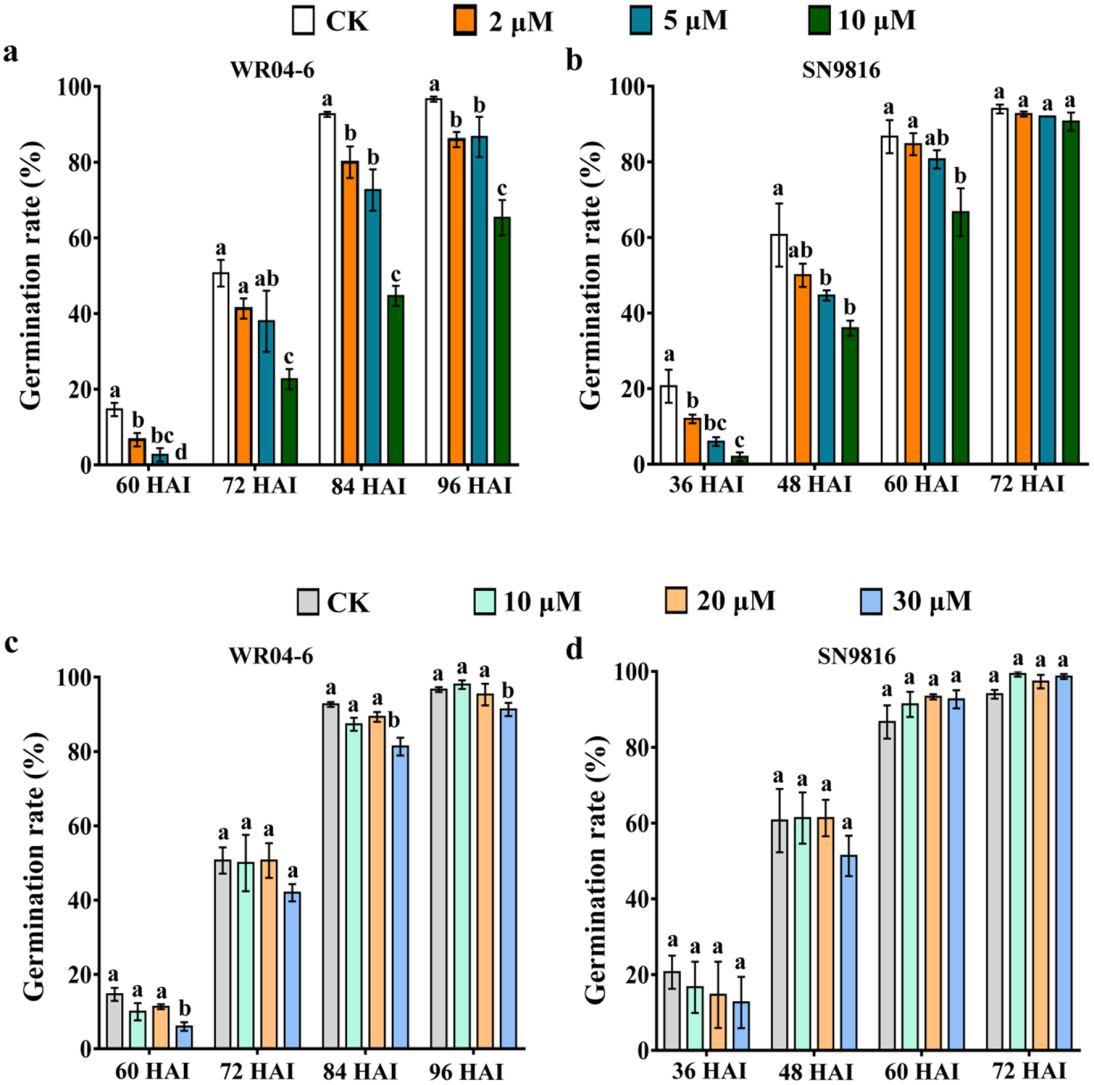
Germination rate (%) of WR04-6 and SN9816 seeds for different concentrations of ABA or diniconazole. (**a**) Germination rate (%) of WR04-6 seeds after ABA treatment. (**b**) Germination rate (%) of SN9816 seeds after ABA treatment. (**c**) Germination rate (%) of WR04-6 seeds after diniconazole treatment. (**d**) Germination rate (%) of SN9816 seeds after diniconazole treatment. Different letters above the bars indicate significant differences in the germination rate for different concentrations of ABA or diniconazole (one-way ANOVA. *p* < 0.05). Error bars represent SEs (n = 3).

The effects of ABA on the germination of WR04-6 and SN9816 seeds after exposure to ABA and its catabolic inhibitor diniconazole were examined. Homogeneity of variance test (*p* > 0.05), frequency test (Skewness and Kurtosis values were less than 1), and single-sample K–S test (Z and P values were more than 0.05) showed that the phenotypic data were in line with normal distribution, and the results of ANOVA indicated the significant difference on germination rate of WR04-6 and SN9816 after ABA and diniconazole treatment. The WR04-6 seeds began to germinate 60 h after imbibition (HAI) and reached a 100% germination rate at 96 HAI under normal conditions. The proportion of germinated seeds decreased significantly when seeds were treated with ABA in a concentration-dependent manner (Fig. 1a). At 10 μM ABA, the seed germination rate was only 65% at 96 HAI. Conversely, SN9816 seeds were capable of germination at all ABA concentrations, although slightly delayed, indicating that the SN9816 seeds were insensitive to ABA relative to WR04-6 seeds (Fig. 1b).

Diniconazole treatment increased the ABA content in seeds by inhibiting ABA catabolism^28^. Compared with untreated WR04-6 seeds, low concentrations of inhibitor (10 μM or 20 μM) had no significant effect on seed germination, but a relatively lower germination rate was observed with 30 μM diniconazole, suggesting that catabolism of ABA plays a role in seed germination (Fig. 1c). In contrast, there was no significant effect of treatment with diniconazole on the germination of SN9816 seeds (Fig. 1d).

### Effects of ABA on post-germination growth

**Figure 2:**
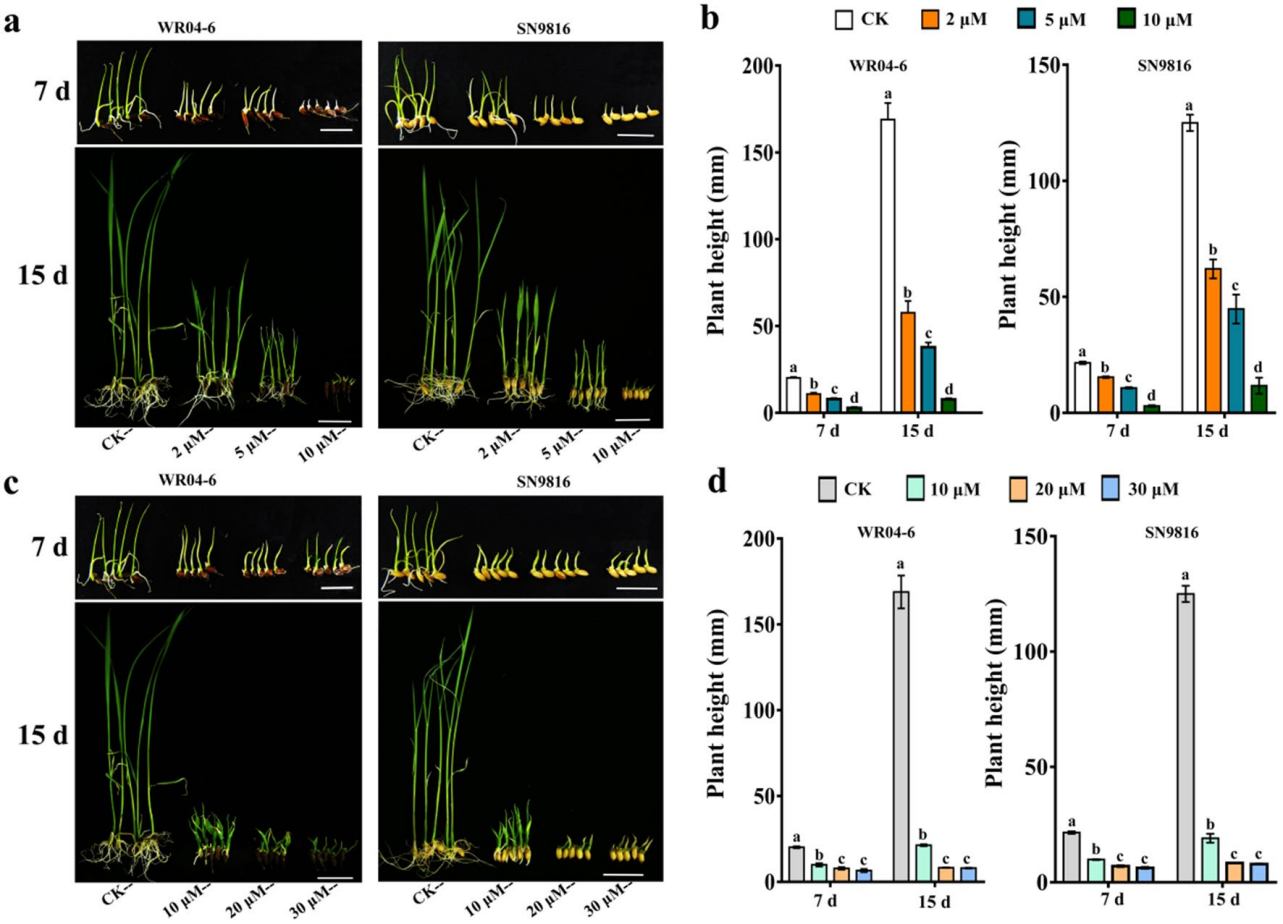
The growth and development of WR04-6 and SN9816 seedlings were retarded when they were continuously treated with ABA or diniconazole. (**a**) Growth of WR04-6 and SN9816 seedlings after 7 d and 15 d of continuous treatment with ABA. Bar = 2 cm. (**b**) Comparison of plant height (mm) of WR04-6 and SN9816 seedlings after treatment with different concentrations of ABA for 7 days and 15 days. (**c**) Growth of WR04-6 and SN9816 seedlings with continuous diniconazole treatment for 7 d and 15 d. Bar = 2 cm. (**d**) Comparison of plant height (mm) of WR04-6 and SN9816 seedlings after treatment with different concentrations of diniconazole for 7 and 15 days. Error bars represent SEs (n = 3). Different letters over the bars indicate treatments that are significantly different (*p* < 0.05).

To further measure the inhibition of post-germination growth when seedlings were continuously exposed to ABA or diniconazole, the development of seedlings at 7 days and 15 days was investigated (Fig. 2). The height of WR04-6 plants grown hydroponically without treatment was approximately 169 mm, which was significantly higher than that of SN9816 after 15 days (125 mm, *t* < 0.05) (Fig. 2a, b). ABA increasingly retarded both WR04-6 and SN9816 plant development at increasing concentrations. Similarly, the height of WR04-6 and SN9816 plants decreased significantly with increasing diniconazole concentrations at 7 days and 15 days (Fig. 2c, d). Plant growth was completely inhibited in both WR04-6 and SN9816 plants when diniconazole concentrations in the medium increased to 20 μM and 30 μM. It should be noted that across different ABA and diniconazole concentrations, the degree of reduction in plant height for WR04-6 was much greater than that for SN9816 at 15 days.

### Evaluation of oxidative stress and antioxidative enzyme activity

**Figure 3:**
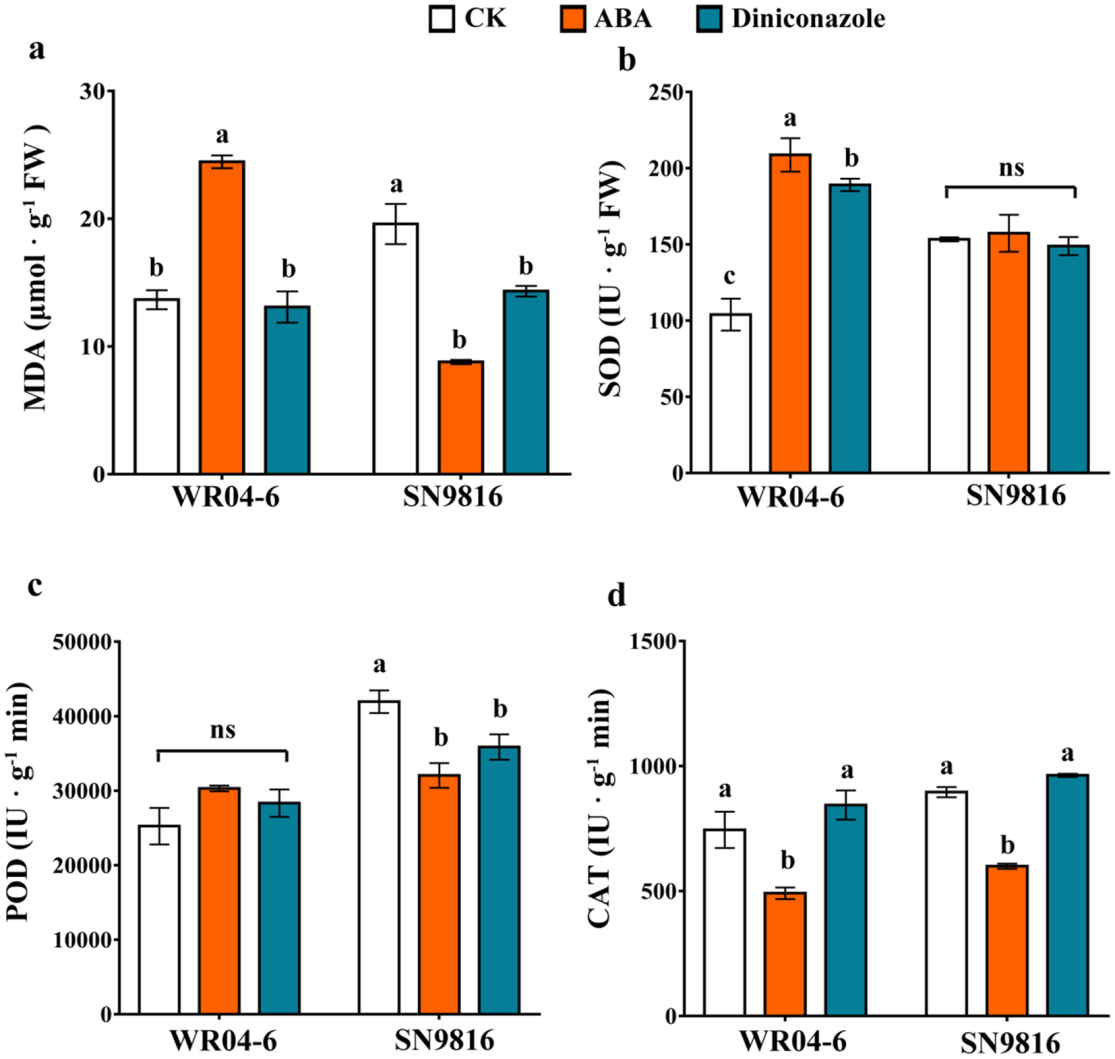
Evaluation of MDA concentration and antioxidant enzyme activity in WR04-6 and SN9816 seedlings after treatment with 2 μM ABA or 10 μM diniconazole. (**a**) MDA content. (**b**) SOD activity. (**c**) POD activity. (**d**) CAT activity. Error bars represent SEs (n = 3). Different letters indicate significant differences between treatments. ns indicates no difference (*p* < 0.05).

In preliminary experiments to determine the effective ABA and diniconazole concentrations for plant growth, seedlings were treated with 2 μM ABA and 10 μM diniconazole for the analysis of physiological indices. A significant accumulation of malondiadehyde (MDA), 1.79-fold greater than that of the control group, was observed in WR04-6 seedlings following ABA treatment (Fig. 3a), while diniconazole treatment did not induce MDA accumulation. The MDA concentration in unstressed SN9816 seedlings was higher than that in the WR04-6 control group. Both ABA and diniconazole resulted in a considerable decrease in the MDA concentration in SN9816 seedlings, which was 0.45- and 0.73-fold lower than that in the unstressed seedlings.

The activity of the antioxidative enzymes SOD, POD, and CAT was evaluated after treatment with ABA and diniconazole. WR04-6 seedlings exposed to ABA and diniconazole experienced significantly increased SOD activity (Fig. 3b). The increased SOD activity resulting from ABA treatment was significantly greater than that after diniconazole treatment. However, there was no difference in SOD activity in the control compared with SN9816 seedlings treated with ABA or diniconazole. Similarly, POD activity in WR04-6 seedlings treated with ABA or diniconazole did not change significantly (Fig. 3c), while a significant decline was observed in SN9816 seedlings treated with ABA or diniconazole compared with the control. CAT activity in both varieties displayed similar trends when plants were exposed to stimulated stress conditions, decreasing considerably when treated with ABA but not changing when treated with diniconazole (Fig. 3d).

### Transcriptome sequencing and de novo assembly

To elucidate the possible molecular mechanisms of the difference in response to exogenous ABA or diniconazole in WR04-6 and SN9816 plants, eighteen cDNA libraries (three replicates per treatment: WR_CK, WR_A, WR_D, SN_CK, SN_A, and SN_D) were constructed, and RNA-Seq libraries were sequenced (Table S1). After the removal of low-quality reads, an average of 53,584,168 high-quality clean reads were obtained from each sample, accounting for 96.79% of the 53,697,638 raw reads. The mean GC content was 54.62%. The proportions of Q20 and Q30 quality scores were 97.88% and 94.02%, respectively, indicating that all libraries were of high quality. Clean reads of each sample were sequentially aligned with the designated reference, and the alignment efficiency ranged from 96.42%to 97.39%.

### Differential transcriptome response to exogenous treatments

**Figure 4:**
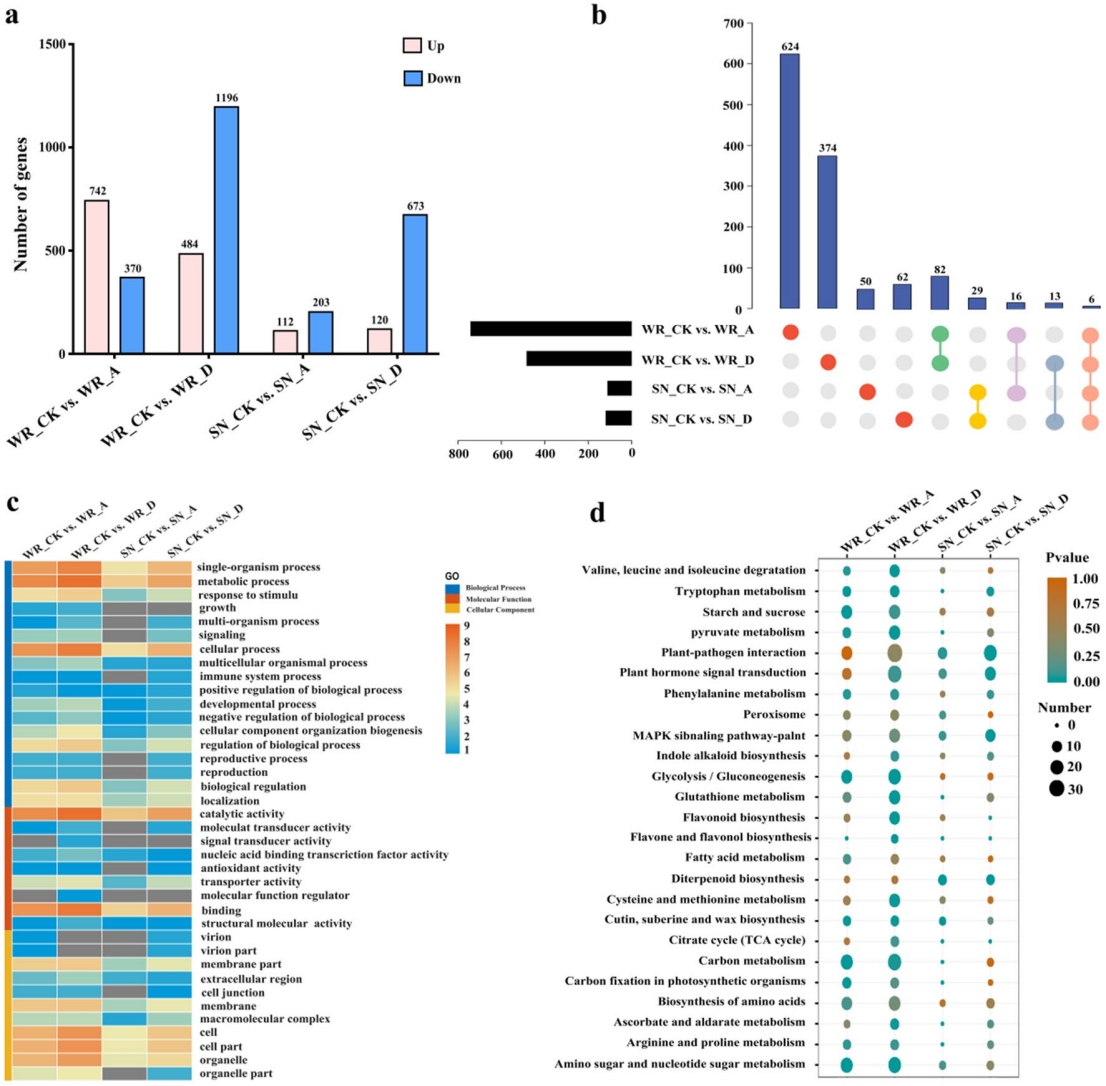
Plot of differentially expressed genes (DEGs) from different comparisons of samples from WR04-6 and SN9816 plants after ABA and diniconazole treatments. (**a**) Numbers of DEGs resulting from pairwise comparison of samples between WR04-6 and SN9816 after ABA and diniconazole treatments. A in WR_A or SN_A indicates ABA treatment, D in WR_D or SN_D indicates diniconazole treatment, and CK in WR_CK or SN_CK indicates the control group without treatment. (**b**) Congruence of the up-regulation of DEGs from pairwise comparisons of samples after treatment with ABA or diniconazole in WR04-6 and SN9816 plants. Red represents DEGs uniquely expressed in each treatment for WR04-6 and SN9816 plants; green represents DEGs uniquely expressed in WR_A and WR_D; yellow represents DEGs uniquely expressed in SN_A and SN_D; purple represents DEGs uniquely expressed in WR_A and SN_A; Blue-grey represents DEGs uniquely expressed in WR_D and SN_D; and pink represents DEGs uniquely expressed after both ABA and diniconazole treatments in the two varieties. (**c**) GO enrichment analysis of the differentially expressed genes presented as a heat map. (**d**) KEGG enrichment analysis of differentially expressed genes presented as a bubble chart.

WR04-6 and SN9816 seedlings displayed a significantly different transcriptional response to different treatments. A total of 1112 genes were differentially expressed in the WR_CK versus WR_A group, of which 742 were up-regulated and 370 were down-regulated (Fig. 4a; Table S2). A total of 1680 genes were differentially expressed in the WR_CK versus WR_D group, of which 484 were significantly up-regulated and 1196 were down-regulated (Fig. 4a; Table S3). A considerably smaller number of genes in SN9816 displayed altered expression levels compared with WR04-6 when exposed to ABA and diniconazole. Only 112 up-regulated and 203 down-regulated genes were identified in the comparison between SN_CK and SN_A in SN9816 (Table S4), and 120 up-regulated and 673 down-regulated genes were identified for the comparison SN_CK versus SN_D (Table S5).

To better understand the difference in the transcriptional response of WR04-6 and SN9816 seedlings to exogenous treatment, the congruence of genes from pairwise comparisons of samples after ABA or diniconazole treatment was explored by screening up-regulated DEGs (Fig. 4b). A total of 624, 374, 50, and 62 DEGs were identified that were uniquely expressed in the WR_CK versus WR_A, WR_CK versus WR_D, SN_CK versus SN_A, and SN_CK versus SN_D groups, respectively. There were 82, 29, 16, and 13 common DEGs that were shared across the comparisons WR_A versus WR_D, SN_A versus SN_D, WR_A versus SN_A, and WR_D versus SN_D, respectively. However, only six genes were commonly up-regulated in the two varieties in response to ABA or diniconazole treatment.

### DEG annotation and functional categorization

GO enrichment analysis was performed on the DEGs obtained from the four pairwise transcriptome comparisons. GO annotation allocated all DEGs into three categories: biological process, molecular function, or cellular process. A total of 36 terms were significantly enriched in both the WR_CK versus WR_A and WR_CK versus WR_A comparisons, while 24 and 35 terms were significantly enriched in the SN_CK versus SN_A and SN_CK versus SN_D comparisons, respectively (Fig. 4c). The enriched terms varied greatly in WR_CK versus WR_A compared with SN_CK versus SN_A. The enriched terms were grouped into biological processes (signaling, growth, reproduction, reproductive process, immune system process, and multi organism), molecular functions (organelle part, cell junction, virion, and virion part), and cellular processes (antioxidant activity and molecular transducer activity), suggesting that the WR04-6 and SN9816 seedlings had different sensitivities to ABA. However, WR04-6 and SN9816 exhibited similar GO terms when exposed to diniconazole (Fig. S1).

KEGG pathway enrichment analysis was conducted to determine the changes in metabolic pathways following treatment with ABA and diniconazole. The DEGs were enriched in 103 and 105 KEGG pathway terms in WR04-6 plants after ABA and diniconazole treatments, respectively. However, a total of 58 and 78 pathways were found to be enriched in SN9816 after ABA and diniconazole treatment, respectively. The top 25 enrichment pathways for each group of DEGs are shown in Fig. 4d. DEGs common to both varieties were enriched in pathways related to plant-pathogen interaction (KO04626), plant hormone signal transduction (KO04075), and MAPK signaling pathways (KO04016). Furthermore, several genes involved in carbon metabolism (KO01200), glycolysis/gluconeogenesis (KO00010), starch and sucrose metabolism (KO00500), biosynthesis of amino acids (KO01230), glutathione metabolism (KO00480), arginine and proline metabolism (KO00330), and peroxisomes (KO04146) were expressed at higher levels in WR04-6 plants than in SN9816 plants.

### Effect of exogenous ABA and diniconazole on ROS homeostasis

**Figure 5:**
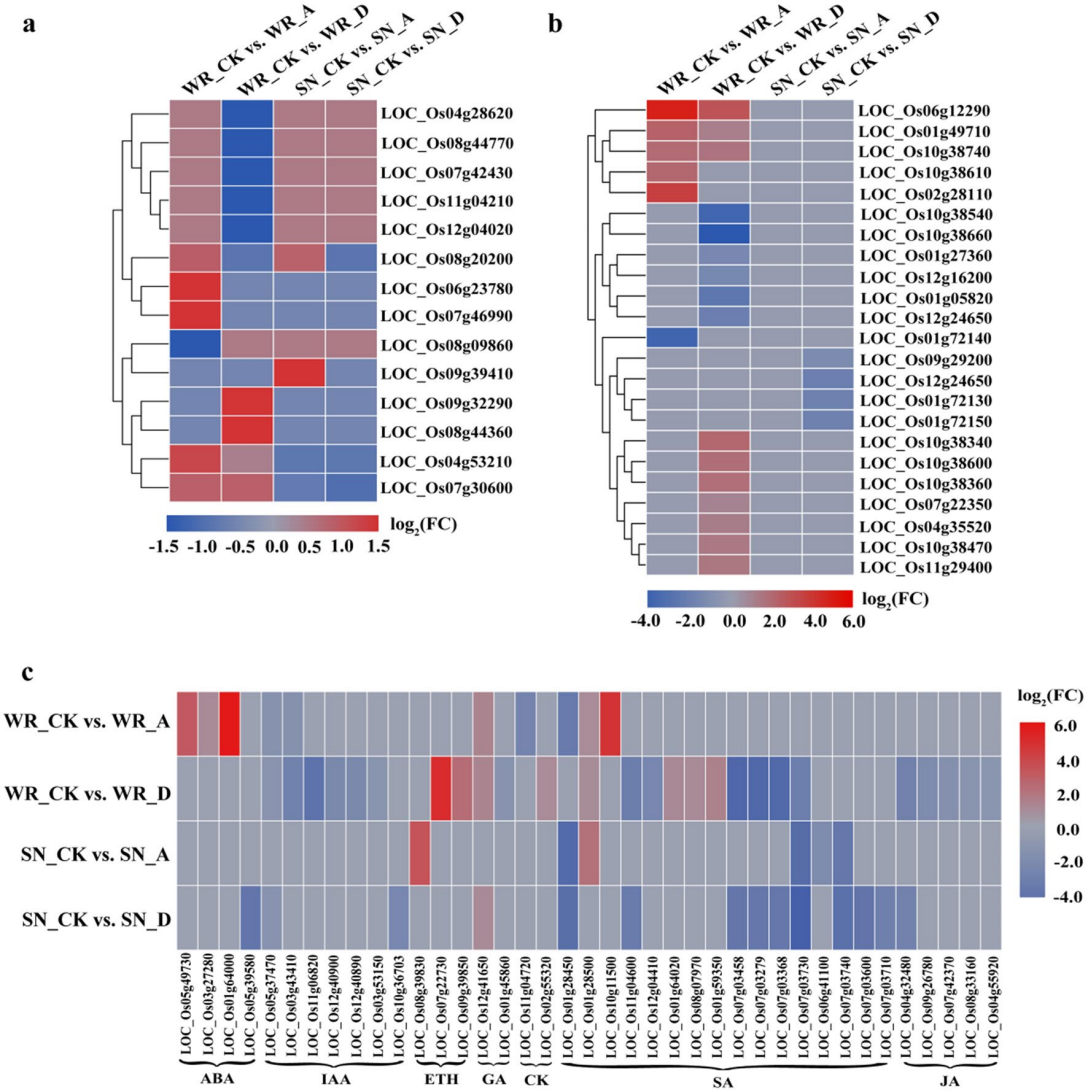
DEGs relevant to peroxisome, glutathione metabolism, and hormone signaling after treatment with ABA or diniconazole. (**a**) Change in transcript expression levels associated with peroxisomes in WR04-6 and SN9816 plants after ABA and diniconazole treatment. (**b**) Expression patterns of genes involved in the glutathione metabolism pathway in the four pairwise transcriptome comparisons. (**c**) DEGs involved in hormone signaling in response to ABA and diniconazole treatment in WR04-6 and SN9816 plants. Gene expression levels were transformed by log_2_(FPKM) values. Red and blue represent up-regulation and down-regulation, respectively. A in WR_A or SN_A indicates ABA treatment, D in WR_D or SN_D indicates diniconazole treatment, and CK in WR_CK or SN_CK indicates the control group without treatment.

Reactive oxygen species (ROS) serve as signals of cellular homeostasis, particularly in the cells of the immune system. Several genes related to peroxidase had higher expression in WR04-6 than in SN9816 under ABA and diniconazole treatments. In the WR_CK versus WR_A group, five genes encoding peroxidase were identified, of which four were up-regulated and only one was down-regulated (Fig. 5a). There were eight genes related to peroxidase in the WR_CK versus WR_D group, of which only three (*LOC_Os04g53210*, *LOC_Os09g32290*, and *LOC_Os08g44360*) were up-regulated. A smaller number of genes with altered expression levels were identified in SN9816 than in WR04-6 when exposed to ABA and diniconazole. One down-regulated and two up-regulated genes were identified in a comparison of SN_CK and SN_A in SN9816. In the SN_CK versus SN_D group, only *LOC_Os07g30600* was enriched and significantly down-regulated.

Furthermore, the induction of glutathione metabolic genes was prominent in WR04-6 under ABA and diniconazole treatments. Seven genes encoding glutathione S-transferases (GSTs) were identified in WR_CK versus WR_A, of which five were up-regulated and two were down-regulated. There were more DEGs in the WR_CK versus WR_D group than in the WR_CK versus WR_A group, and a total of 16 genes were identified, of which 10 were up-regulated and six were down-regulated. However, no gene was differentially expressed in SN9816 after ABA treatment, and four genes were differentially expressed in the SN_CK versus SN_D group and were significantly down-regulated.

### Response of phytohormone-related genes to exogenous treatment

Genes related to phytohormone signaling were also differentially expressed in response to ABA and diniconazole. A total of 40 DEGs involved in seven hormones, including abscisic acid (ABA), auxin (IAA), ethylene (ETH), gibberellic acid (GA), cytokinin (CK), salicylic acid (SA), and jasmonic acid (JA), were identified from four pairwise transcriptome comparisons (Fig. 5c, Table S6). A total of 10 DEGs were identified in the WR_CK versus WR_A group, and genes related to ABA-, GA-, and SA-responsive genes were dramatically up-regulated, while two (*LOC_Os05g37470* and *LOC_Os03g43410*) IAA-responsive genes and one (*LOC_Os11g04720*) CK-responsive gene were down-regulated. There were more DEGs in the WR_CK versus WR_D group than in the WR_CK versus WR_A group. IAA and JA displayed a negative response to diniconazole, as all DEGs related to IAA- and JA-responsive genes were significantly down-regulated. Of the ten DEGs related to the SA signaling pathway, four (*LOC_Os01g28500*, *LOC_Os01g64020*, *LOC_Os08g07970*, and *LOC_Os01g59350*) were significantly up-regulated, and six (*LOC_Os11g04600*, *LOC_Os12g04410*, *LOC_Os07g03458*, *LOC_Os07g03279*, *LOC_Os07g03368*, and *LOC_Os07g03730*) were down-regulated. In addition, ETH and GA displayed a positive regulatory effect due to exogenous treatment. *SLRL1* (*LOC_Os01g45860*), orthologous to SLR1 and a negative regulator of the GA signaling pathway, was significantly down-regulated in the WR_CK versus WR_D group. Expression levels of DEGs in SN9816 were considerably lower than those in WR04-6 after treatment with ABA and diniconazole. Only one ETH-responsive gene (*LOC_Os08g39830*) was identified in the SN_CK versus SN_A group, and that was up-regulated. The expression of SA-responsive genes was lower in response to ABA treatment, with four genes (*LOC_Os01g28450*, *LOC_Os07g03730*, *LOC_Os06g41100*, and *LOC_Os07g03740*) significantly down-regulated and one gene, *LOC_Os01g28500*, up-regulated. A total of 14 DEGs were identified in the SN_CK versus SN_D group, of which one GA-responsive gene (*LOC_Os12g41650*) was up-regulated while the other genes involved in the ABA, IAA, SA, and JA signaling pathways were down-regulated.

### Cluster analysis of defense-related genes in exogenous treatments

**Figure 6:**
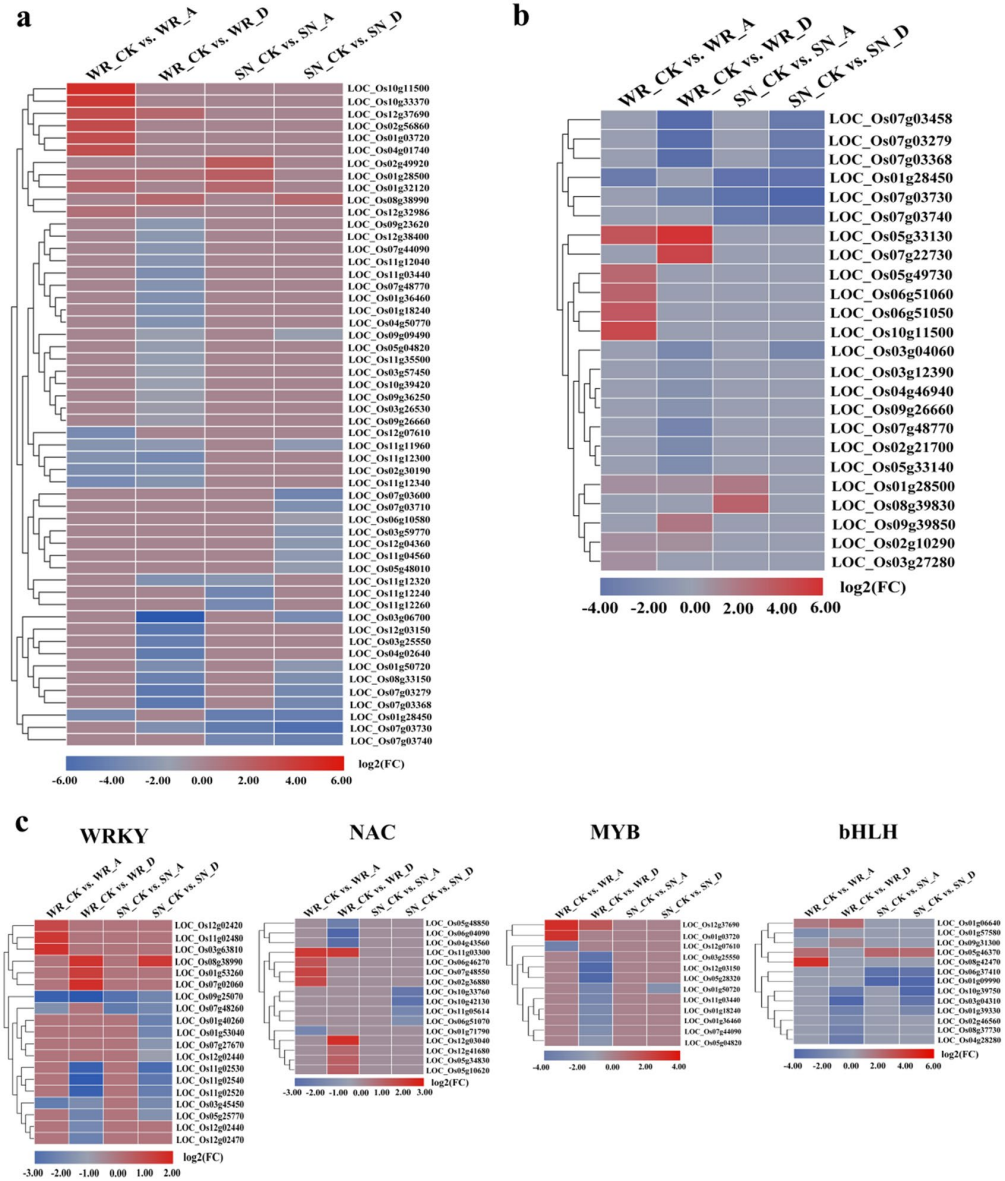
Expression profiles of defense-related genes in WR04-6 and SN9816 plants after exogenous treatments. Comparative analysis of pathogen interactions (**a**), MAPK signaling pathway transduction (**b**), and the expression of transcription factor related genes (**c**) in WR04-6 and SN9816 plants after exogenous treatment. Gene expression levels were transformed by log_2_(FPKM) values. Red and blue represent up-regulation and down-regulation, respectively. A in WR_A or SN_A indicates ABA treatment, D in WR_D or SN_D indicates diniconazole treatment, and CK in WR_CK or SN_CK indicates the control group without treatment.

An analysis of DEGs indicated the presence of a milieu of defense-related genes that participated in the ABA and diniconazole response. In the WR_CK versus WR_A group, 15 genes were identified that participate in the interaction between plants and pathogens, of which nine were up-regulated and six were down-regulated. There were 35 genes involved in the plant-pathogen interaction pathway in the WR_CK versus WR_D group, of which only three (*LOC_Os01g28500*, *LOC_Os08g38990*, and *LOC_Os12g37690*) were up-regulated (Fig. 6a, b, Table S7). A total of nine and 20 genes were identified that were involved in the plant-pathogen interaction pathway in SN9816 following ABA or diniconazole treatment, respectively. Three genes (*LOC_Os01g28500*, *LOC_Os01g32120*, and *LOC_Os02g49920*) were found to be up-regulated in SN_CK versus SN_A, while one (*LOC_Os08g38990*) was identified in SN_CK versus SN_D.

The mitogen-activated protein kinase (MAPK) signaling pathway plays a positive role in plants exposed to adverse conditions. The pathogen-related genes *LOC_Os01g28500*, *LOC_Os07g48770*, *LOC_Os07g03730*, *LOC_Os09g26660* (*OsrbohB*), *LOC_Os01g28450*, *LOC_Os07g03740*, and LOC_Os10g11500 were also involved in the MAPK signaling pathway. *LOC_Os05g33130*, *LOC_Os05g49730*, *LOC_Os06g51050*, and *LOC_Os06g51060* were uniquely expressed in the WR_CK versus WR_A group (Fig. 6b).

Transcription factors (TFs) also play an important role in the response of plants to stress. There were 56 (41 up-regulated, 15 down-regulated), 111 (39 up-regulated, 72 down-regulated), 20 (6 up-regulated, 14 down-regulated), and 50 (7 up-regulated, 43 down-regulated) TFs identified in the WR_CK versus WR_A, WR_CK versus WR_D, SN_CK versus SN_A, and SN_CK versus SN_D groups, respectively (Table S8). The up-regulated TFs in the present study were members of a variety of families, with the majority representing WRKY, NAC, MYB, and bHLH families in response to ABA or diniconazole treatment. The genes *LOC_Os11g02480*, *LOC_Os12g02420*, and *LOC_Os03g63810*, belonging to the WRKY family, were up-regulated in the WR_CK versus WR_A group. Twelve WRKY family genes were identified in the WR_CK versus WR_D group, of which *LOC_Os08g38990*, *LOC_Os07g02060*, and *LOC_Os01g53260* were up-regulated. Only one gene (*LOC_Os08g38990*) was positively regulated in SN9816 in response to diniconazole treatment (Fig. 6c, Table S8). Furthermore, genes belonging to the NAC, MYB, and bHLH TF families were positively regulated in WR04-6 after ABA or diniconazole treatment. Considerably fewer NAC, MYB, and bHLH TFs were identified in the SN_CK versus SN_A and SN_CK versus SN_D groups, and the majority were down-regulated.

### DEGs involved in glycolysis and the citrate (TCA) cycle

**Figure 7:**
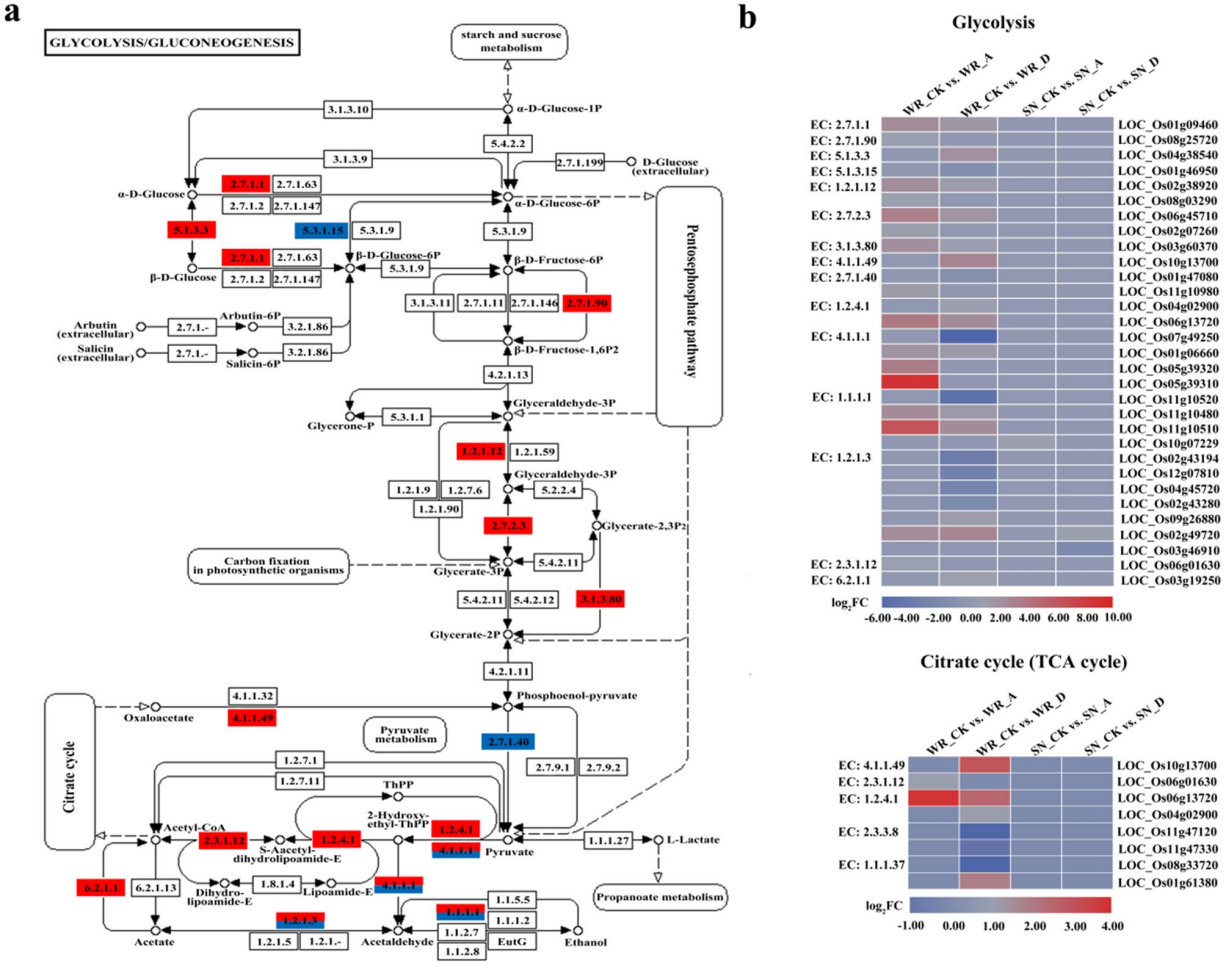
DEGs relevant to glycolytic metabolism and the citrate cycle after treatment with ABA or diniconazole. (**a**) Change in transcript expression levels associated with glycolysis and citrate cycle pathways in WR04-6 and SN9816 plants after ABA and diniconazole treatment. (**b**) Expression patterns of genes involved in glycolysis and citrate cycle pathways in the four pairwise transcriptome comparisons.

The distribution of DEGs revealed that glycolysis/gluconeogenesis and the citrate cycle (TCA cycle) were strongly affected in WR04-6 plants following treatment with 2 μM ABA and 10 μM diniconazole (Fig. 7a). In the present study, 31 DEGs that participate in the glycolysis pathway were identified in the WR_CK versus WR_A and WR_CK versus WR_D groups, of which 21 encoding enzymes important to glycolysis were significantly up-regulated (Fig. 7b). These were hexokinase (EC: 2.7.1.1), diphosphate-dependent phosphofructokinase (EC: 2.7.1.90), aldose 1-epimerase (EC: 5.1.3.3), glyceraldehyde 3-phosphate dehydrogenase (EC: 1.2.1.12), phosphoglycerate kinase (EC: 2.7.2.3), 2,3-bisphosphoglycerate 3-phosphatase (EC: 3.1.3.80), phosphoenolpyruvate carboxykinase (EC: 4.1.1.49), pyruvate kinase (EC: 2.7.1.40), pyruvate dehydrogenase E1 (EC: 1.2.4.1), pyruvate decarboxylase (EC: 4.1.1.1), alcohol dehydrogenase 1/7 (EC: 1.1.1.1), aldehyde dehydrogenase (EC: 1.2.1.3), pyruvate dehydrogenase E2 (EC: 2.3.1.12), and acetyl-CoA synthetase (EC: 6.2.1.1). Only three DEGs (two up-regulated and one down-regulated) were identified in the SN_CK versus SN_A and SN_CK versus SN_D groups. The up-regulated genes encoded alcohol dehydrogenase 1/7 (EC: 1.1.1.1) and aldehyde dehydrogenase (EC: 1.2.1.3).

A total of eight DEGs (five up-regulated and three down-regulated) were identified from the WR_CK versus WR_A and WR_CK versus WR_D groups that participated in the TCA cycle (Fig. 7b). These genes primarily encode key enzymes, including phosphoenolpyruvate carboxykinase (EC: 4.1.1.49), pyruvate dehydrogenase E2 (EC: 2.3.1.12), pyruvate dehydrogenase E1 (EC: 1.2.4.1), ATP citrate (pro-S)-lyase (EC: 2.3.3.8), and malate dehydrogenase (EC: 1.1.1.37). However, no genes from the SN_CK versus SN_A and SN_CK versus SN_D groups participated in the TCA cycle.

### Validation of RNA-Seq data by RT-qPCR

**Figure 8:**
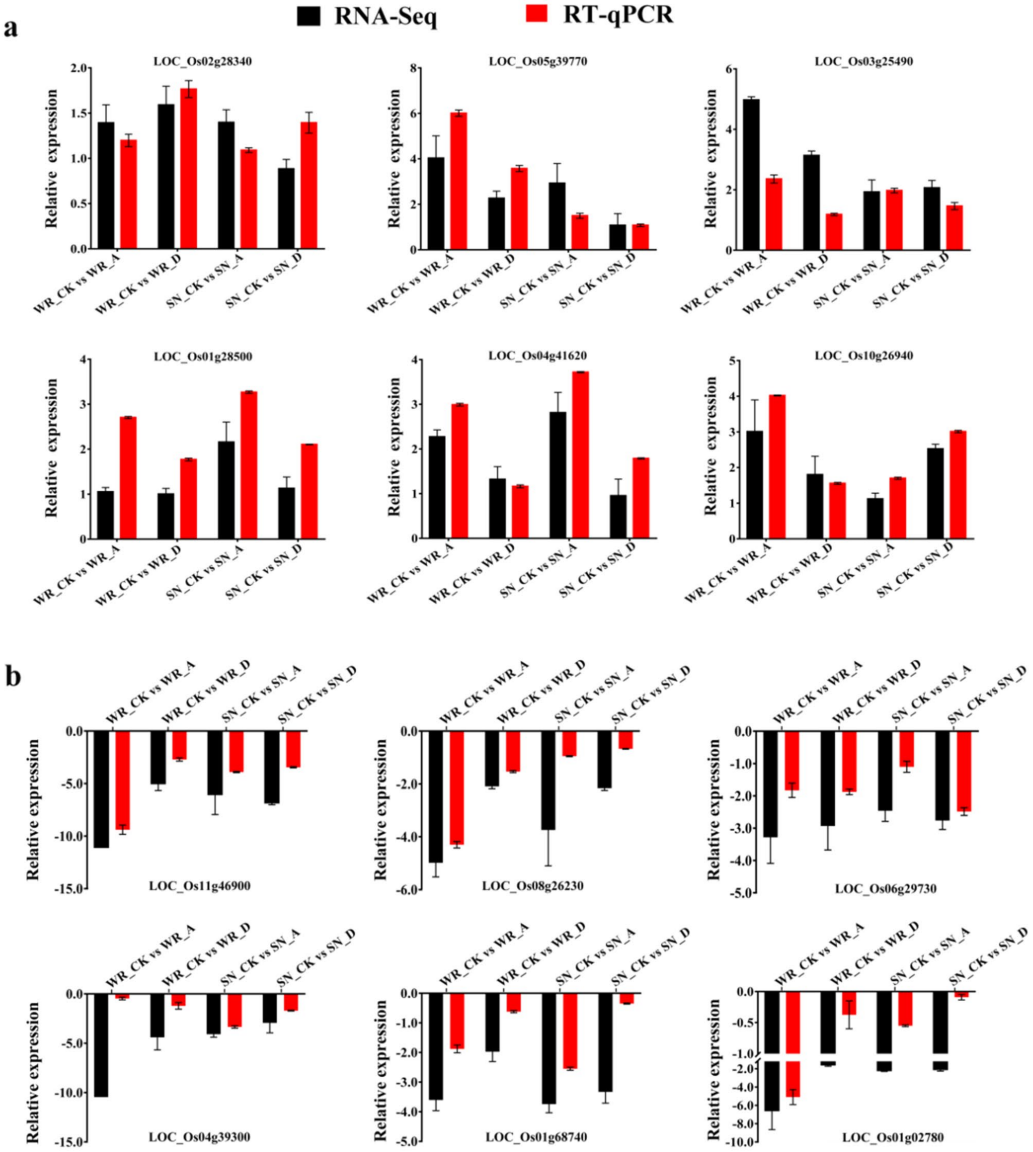
Comparison of relative expression level of twelve genes by RNA-Seq and RT-qPCR. (**a**) up-regulated genes; (**b**) down-regulated genes. Black and red bas represent relative expression level from FPKM and RT-qPCR, respectively. Error bars represent SEs (n = 3).

To further validate the RNA-Seq expression profile data, twelve genes were selected from representative transcripts commonly expressed in each treatment for RT-qPCR assay. The Pearson correlation coefficients (PCCs) between the expression profiles determined by RNA-Seq and by RT-qPCR were analyzed by a Gaussian distribution yielding 95% confidence for a PCC = 0.890. The results indicate that the expression profiles of the selected genes were largely consistent with data derived from the RNA-Seq results, suggesting that they were reliable and repeatable (Fig. 8).

## Discussion

ABA is generally known to inhibit seed germination and post-germination growth^29^. The current analysis revealed that ABA delayed seed germination of WR04-6 and SN9816 plants in an ABA concentration-dependent manner. This is similar to *Arabidopsis*, where a statistical correlation was confirmed between ABA concentration and the rate of seed germination^30^. However, a slight delay in SN9816 seed germination was observed for each concentration of ABA (Fig. 1a). ABA represses germination through metabolism and signaling^31^. Diniconazole has been shown to inhibit ABA 8′-hydroxylase activity, resulting in a twofold increase in endogenous ABA concentration in potatoes^32^. As expected, high concentrations of diniconazole slightly delayed seed germination in WR04-6 but had no effect on the germination of SN9816 at the same concentration (Fig. 1b). Furthermore, continuous ABA or diniconazole treatment significantly inhibited the post-germination growth of both WR04-6 and SN9816 seeds, although ABA and diniconazole more significantly inhibited plant height in WR04-6 (Fig. 2). Similar results have been observed in *Arabidopsis*^33^, which demonstrated that the ABA 8′-hydroxylase gene inhibited seedling development by influencing ABA metabolism. These results indicate that ABA plays a larger role in WR04-6 seed germination and post-germination growth than it does in SN9816 plants.

Damage to membranes and H_2_O_2_ accumulation are considered the principal modes of damage that comprise the oxidative stress process^34^. MDA is the product of membrane lipid peroxidation and is indicative of compromised membrane integrity and structure. ABA significantly increased the content of MDA in WR04-6, while the opposite was observed in SN9816 (Fig. 3a). Plants enhance stress tolerance by regulating antioxidant activity. SOD is considered a critical enzyme for the regulation of O_2_^-^ and H_2_O_2_ levels^35^. In the present study, SOD activity increased significantly following exogenous ABA or diniconazole treatment in WR04-6 plants (Fig. 3b). Similar results have previously been observed in tomato plants after ABA treatment^36^, demonstrating up-regulation of SOD activity. However, only a slight effect was observed on SOD activity in SN9816 plants after ABA or diniconazole treatment. POD and CAT break down H_2_O_2_, producing water and molecular oxygen^37^. POD activity in WR04-6 changed slightly after ABA or diniconazole application, but CAT activity decreased significantly after the application of ABA. Surprisingly, POD and CAT activity in SN9816 plants decreased significantly after ABA or diniconazole treatment (Fig. 3c, d). In agreement with this result, several antioxidant enzyme-related genes were differentially expressed, including GSTs, SODs, and APXs (Fig. 5a, b). In this study, *LOC_Os01g49710*, *LOC_Os06g12290*, and *LOC_Os10g38740*, which are involved in glutathione metabolism, were up-regulated in WR04-6 under both ABA and diniconazole applications. *LOC_Os10g38470*, *LOC_Os11g29400*, *LOC_Os07g22350*, *LOC_Os10g38600*, *LOC_Os10g38340*, and *LOC_Os10g38360* were uniquely up-regulated in the WR_CK versus WR_D group, and these genes may play an important role in maintaining ROS homeostasis in WR04-6 plants.

Hormonal regulation plays an important role and could be an important factor that defines sensitivity and tolerance in plant systems^38^. A complex relationship in stress-responsive genes that depends on the transduction of phytohormone signaling likely exists in the present study. Exposure of WR04-6 seedlings to ABA significantly increased the expression of ABA-related genes, and genes related to SA signal transduction were additionally induced. Subsequently, IAA and CK signal transduction was negatively regulated by ABA, further suppressing cell enlargement and plant growth. Distinct from ABA treatment, the accumulation of endogenous ABA induced by diniconazole promoted the expression of EIL- and ERF-related genes. As JAZs are located upstream of ERFs, overexpression of ERF feedback suppresses JAZs, thus inhibiting the JA signal transduction pathway (Fig. 5c). A similar hormone regulatory network was established in wheat roots after ABA treatment^15^, indicating that phytohormone signaling transduction pathways are critical in stress responses.

SA can increase the activity of superoxide dismutase-producing enzymes, leading to increased H_2_O_2_ and causing a series of hypersensitive responses in plants that ultimately enhance resistance to plant disease^39^. Sixteen SA-related genes were identified in this study, with genes *LOC_Os01g28500 LOC_Os01g28450*, and *LOC_Os07g03730* identified in both varieties. *LOC_Os01g64020*, *LOC_Os08g07970*, and *LOC_Os01g59350* (*OsbZIP64*) had unique expression profiles in the WR_CK versus WR_D group, and these genes may play an important role in plant disease resistance in WR04-6 plants.

The WRKY, MYB, NAC, and bHLH families of TFs play an important role in plant immunity by inducing the expression of downstream defense-related genes. Previous studies have shown that drought, low temperature, and bacterial infection induce the overexpression of WRKY family TFs^39–41^. In the present study, *LOC_Os11g02480* (*OsWRKY46a*), *LOC_Os12g02420* (*OsWRKY46b*), and *LOC_Os03g63810* (*OsWRKY80*), which belong to the WRKY family, were uniquely up-regulated in the WR_CK versus WR_A group (Fig. 6c). In addition, *LOC_Os07g02060* (*OsWRKY29*) and *LOC_Os01g53260* (*OsWRKY23*) were uniquely up-regulated in the WR_CK versus WR_D group. It has been reported that *LOC_Os03g63810* (*OsWRKY80*) functions as a positive transcriptional regulator against *Rhizoctonia solani* by directly binding to the promoter of *OsWRKY4*^42^. These results are consistent with WRKY TFs being involved in plant defense responses. NAC TFs appear to be widespread in plants, and the expression of multiple *NAC* genes is induced by abiotic and biotic stress^43^. Many studies have used NAC TFs to improve stress tolerance in plants by genetic engineering^44^. In the present study, 17 NAC TFs were identified in the two plant varieties, and the genes *LOC_Os11g03300* (*OsNAC10*), *LOC_Os07g48550* (–), *LOC_Os06g46270* (*OMTN4*), *LOC_Os02g36880* (*OMTN1*), *LOC_Os05g34830* (*OsNAC52*), *LOC_Os12g03040* (*ONAC131*), *LOC_Os12g41680* (*OMTN3*), and *LOC_Os05g10620* (–) were uniquely expressed in the WR_CK versus WR_A and WR_CK versus WR_D groups. The rice pathogen-responsive gene *LOC_Os12g03040* (*ONAC131*), plays important roles in the disease resistance response through the regulated expression of other defense- and signaling-related genes^45^. Overexpression of *LOC_Os11g03300* (*OsNAC10*) and *LOC_Os05g34830* (*OsNAC52*) can improve tolerance to drought and low temperature, respectively^46,47^. These data suggest that NAC TFs possess potential utility in improving stress tolerance in crops. Additionally, the novel genes *LOC_Os12g37690* and *LOC_Os01g03720* belonging to the MYB family of TFs, and *LOC_Os08g42470*, *LOC_Os01g06640*, and *LOC_Os09g31300* genes belonging to the bHLH family were expressed in WR04-6 after application of ABA or diniconazole.

Changes in carbohydrate content are of particular importance and affect the activity of plant physiological processes, such as photosynthesis, respiration, and metabolism^48^. Previous studies have shown that the carbohydrate metabolic pathway is the most sensitive in plants to stress conditions^40^. DEGs identified by transcriptome sequencing were mostly related to glycolysis, starch and sucrose metabolism, the TCA cycle, and pyruvate metabolic pathways. These observations are consistent with our results in which multiple DEGs related to carbohydrate metabolism were mapped to glycolysis and TCA cycle pathways. Glycolysis is a key respiratory pathway that provides ATP, reducing agents, and metabolites for plant growth and development^49^. When experiencing abiotic stress, glycolysis is often involved in the regulation of adaptability of plants to the environment^50^. In the present study, both ABA and diniconazole treatment in WR04-6 plants caused enrichment in the glycolysis pathway, with 16 and 22 DEGs enriched after ABA and diniconazole treatment, respectively. However, only one gene (*LOC_Os10g07229*) that participates in glycolysis was induced in SN9816 after ABA treatment, and only two genes (*LOC_Os02g49720* and *LOC_Os03g46910*) were induced after diniconazole treatment. The TCA cycle is the principal means of aerobic decomposition of glucose during respiration, which gradually oxidizes and decomposes the glycolytic product pyruvate into CO_2_ and H_2_O_2_, generating NADH, FADH_2_, and ATP that support stress tolerance^51^. RNA-Seq data demonstrated that three genes were enriched after ABA treatment and seven after the application of diniconazole. Similar results have been previously observed in *Dendrobium officinale*^52^ and *Poa pratensis*^53^ exposed to abiotic stress conditions. These results indicate that WR04-6 can respond to ABA by actively regulating glycolysis and TCA cycle pathway-related genes and enzyme activities. Taken together, weedy rice (WR04-6) and cultivated rice (SN9816) showed different transcriptional characteristics under ABA or diniconazole treatment. Several differentially expressed genes had unique expression profiles in the WR_CK versus WR_A and WR_CK versus WR_D groups, these genes may serve as candidates to be used for further crop improvement (Table S9).

## Conclusion

This study revealed different physiological and molecular mechanisms in response to ABA and diniconazole between weedy rice and cultivated rice with contrasting ABA sensitivity. The transcriptomic analysis uncovered global and weedy-specific reactions of WR04-6 in response to ABA and diniconazole, such as the enhancement of antioxidative actions, stress defense, hormone signaling transduction, and glycolytic and citrate cycle activities. The candidate genes screened in this study will provide novel material for genetic and functional studies of stress in cultivated rice, which can be used for further crop improvement and breeding of resistant varieties.

## Materials and methods

### Materials

WR04-6, a representative weedy rice accession and *temperate japonica* Shennong9816 (SN9816) as a control rice sample were obtained from the Rice Research Institute of Shenyang Agricultural University. Both of them were grown in germplasm resource field at Rice Research Institute of Shenyang Agricultural University, Shenyang, China. The samples were collected from the germplasm resource field with the permission of Rice Research Institute of Shenyang Agricultural University. Seeds were sown on April 15, 2019, with seedlings transplanted to their final locations on May 26, 2019. Plants were spaced 30.0 × 13.4 cm apart. Fertilizer and water management followed the local standard management. Seeds were harvested 35 days after anthesis and stored at 4 °C until use for experimentation. Experimental research and field studies on plants must comply with relevant institutional, national, and international guidelines and legislation.

### Seed germination and coleoptile sheath length after treatment with ABA and diniconazole

For germination assays, seeds were presoaked in a thermostatic incubator at 50 °C for 3 days to prevent any possible dormancy. Fifty sterilized WR04-6 and SN9816 seeds were sown in distilled water supplemented with different concentrations of ABA (0, 2, 5, or 10 μM) or the ABA catabolism inhibitor diniconazole (0, 10, 20, or 30 μM). Seeds were considered germinated when radicles had emerged from the seed coats. The percentage (%) germination was calculated every 12 h after initiation. All experiments were performed independently three times. After germination, 30 germinated seeds with uniform growth potential for each treatment were transferred to 1/2 × Murashige and Skoog (MS) medium (pH = 5.8) supplemented with ABA or diniconazole appropriate for the experimental grouping and grown at 30 °C within a 16 h light/8 h dark cycle. The coleoptile sheath length was measured and photographed on days 7 and 15 to determine the growth performance for each treatment.

### Analysis of malondialdehyde (MDA) and antioxidant enzyme activity

Following exposure to continuous ABA and diniconazole treatment, seedlings treated with 2 μM ABA and 10 μM diniconazole were selected for analysis of physiological indices. Approximately 0.9 g of fresh leaves of each treatment were collected and ground into powder in liquid nitrogen and homogenized in an ice bath with 5 mL of 50 mmol/L sodium phosphate buffer (pH = 7.8) containing 5 mmol/L ethylenediaminetetraacetic acid (EDTA) and 1% polyvinylpyrrolidone. The homogenate was centrifuged at 12,000 rpm for 20 min at 4 °C, and the supernatant was analyzed for MDA concentration and catalase (CAT), peroxidase (POD), and superoxide dismutase (SOD) activity.

MDA concentration was measured using the thiobarbituric acid (TBA) method, as described in a previous study^54^ with slight modifications. A 2.5 mL mixture of 0.5% trichloroacetic acid (TCA) and 0.5% TBA was added to 1 mL of leaf supernatant, and the sample was heated for 15 min and then cooled quickly in an ice bath. The mixture was then centrifuged at 3000 rpm for 30 min, and the absorbance of the supernatant was measured at 532 nm, from which nonspecific absorption at 600 nm was subtracted. The results were expressed as μmol g^−1^ FW.

SOD activity was measured using a nitroblue tetrazolium (NBT) assay^55^. The 3 mL reaction mixture contained enzyme extract, 130 mM methionine, 0.75 mM NBT, 0.1 mM EDTA, 20 μM riboflavin and 50 mM phosphate buffer (pH 7.8). Tubes were reacted at 4000 lx for 20 min, after which absorbance at 560 nm was measured using a spectrophotometer (Hitachi, U-5100). POD activity was measured in accordance with the method of Zhang et al.^56^. The reaction mixture contained 0.1 mL of enzyme extract, 0.3 mL of 0.6% H_2_O_2_, and 2.6 mL of 0.3% guaiacol. The oxidation of guaiacol by H_2_O_2_ was quantified at 420 nm as a measure of POD activity. CAT activity was measured in accordance with the method described by Kraus et al.^57^. The reaction mixture comprised 0.2 of mL distilled water, 2.8 mL of 60 mM H_2_O_2_, and 0.2 mL of enzyme extract. The decrease in absorbance at 240 nm due to H_2_O_2_ over 3 min was recorded. The results are expressed as IU·g^−1^ FW and IU·g^−1^ min for POD and CAT, respectively.

### RNA isolation, cDNA library construction, and RNA sequencing

Seedlings treated with 2 μM ABA and 10 μM diniconazole were selected for further RNA-Seq analysis. For each treatment, leaf tissue from fifteen plants (three biological replicates with five plants per replicate) with the same growth status was collected 15 days after treatment with hormones. Total RNA was extracted from each sample using TRIzol reagent (Thermo Fisher Scientific, MA, USA) in accordance with the manufacturer’s protocol. RNA concentration was assessed in a Nanodrop 8000 spectrophotometer (Thermo Fisher Scientific, MA, USA), and integrity was verified by agarose gel electrophoresis. After total RNA was extracted, mRNA was enriched by oligo (dT) beads, and then the enriched mRNA was fragmented into short fragments by fragmentation buffer and reverse transcribed into cDNA with random primers. Second-strand cDNA was synthesized by DNA polymerase I, RNase H, dNTPs and buffer. Then, the cDNA fragments were purified with a QiaQuick PCR extraction kit (Qiagen, Venlo, The Netherlands), end repaired, poly(A) added, and ligated to Illumina sequencing adapters. The ligation products were size selected by agarose gel electrophoresis and PCR amplified. The cDNA libraries were sequenced using an Illumina HiSeq 2500 sequencing platform (Illumina Crop., San Diego, CA, USA) at Gene De novo Biotechnology Co., Ltd. (Guangzhou, China).

### Normalization and analysis of differentially expressed genes (DEGs)

Raw data were processed through a quality check using the FastQC protocol (https://github.com/OpenGene/fastp), and clean reads were obtained by removing adapter sequences, low-quality sequences (bases with Q-value ≤ 20) and reads with poly-N. The reference genome of *Oryza sativa* ssp. *japonica* (Os-Nipponbare-Reference-IRGSP-1.0) was obtained from The Rice Annotation Project Database (RAP-DB) (https://rapdb.dna.affrc.go.jp/index.html). Cleaned short reads were aligned to all exon sequences using HISAT2^58^. Gene expression was calculated with the RSEM tool^59^ using default parameters. Relative expression levels were normalized in terms of fragments per kilobase of exon per million reads (FPKM). DEGs were identified using DESeq2^60^, and genes with |Log2 (Fold Change, FC)|> 1 and a false discovery rate < 0.05 were considered to be significant DEGs. The DEGs underwent gene ontology (GO) enrichment (available online: http://www.geneontology.org/) and Kyoto Encyclopedia of Genes and Genomes (KEGG) (available online: http://kobas.cbi.pku.edu.cn/) pathway analysis. TBtools software was used to determine the differential expression and overlap of transcripts between comparison sets for the treatment and control groups (WR04-6 and SN9816, respectively).

### Quantitative real-time PCR validation

Commonly expressed genes were quantified in samples from each treatment using RT-qPCR. Gene-specific primers were designed using Primer Premier 5 software, as detailed in Table S10. First-strand cDNA was synthesized using a PrimeScript RT regent kit combined with gDNA Eraser (TaKaRa, Dalian, China) in accordance with the manufacturer’s instructions. TB Green Premix Ex Taq II (Tli RNaseH Plus, RR820A) (TaKaRa, Dalian, China) was used to perform RT-qPCR in an Applied Biosystems 7500 Real-Time PCR System (Thermo Fisher Scientific, USA). The reaction conditions were as follows: 95 °C for 30 s, then 40 cycles of 95 °C for 5 s and 60 °C for 34 s. The expression of transcripts was normalized to the reference gene *OsActin* using the 2^−ΔΔCt^ method^61^. Three biological replicates were quantified by RT-qPCR analysis.

### Statistical analysis

Treatment differences were statistically evaluated via one-way analysis of variance (ANOVA), followed by individual comparisons with Duncan’s multiple range tests, using SPSS software v19.0. Student’s *t*-tests (*t* < 0.05) were performed to compare expression between samples. The results are presented as the means ± standard error (SE) of three biological replicates. Pearson correlation analysis was performed to evaluate the correlation between RNA-Seq and RT-qPCR (*p* < 0.05).

## Data availability statement

Raw sequence data were deposited in the NCBI Short Read Archive (SRA; http://www.ncbi.nlm.nih.gov/sra) under accession number PRJNA723912.

## Supplementary Information

Supplementary Information.
