# Supplementary material for: Comparative transcriptomic and physiological analyses of weedy rice and cultivated rice to identify vital differentially expressed genes and pathways regulating the ABA response
Source: Sci Rep. 2021 Jun 18;11:12881. doi: 10.1038/s41598-021-92504-5 (PMC8213743; doi:10.1038/s41598-021-92504-5)
Supplement: Supplementary file 1 — Supplementary Information. [file 41598_2021_92504_MOESM1_ESM.pdf]

Comparative transcriptomic and physiological analyses of weedy rice and cultivated rice to identify vital differentially expressed genes and pathways regulating the ABA response

Hong Lang[1], Yuting He[1], Faliang Zeng[1], Fan Xu[1], Minghui Zhao[1], Dianrong Ma[1,2]

[1]Rice Research Institute of Shenyang Agricultural University, Key Laboratory of Northern Japonica Rice Genetics and Breeding, Ministry of Education and Liaoning Province, Key Laboratory of Northeast Rice Biology and Genetics and Breeding, Ministry of Agriculture, Shenyang 110866, China

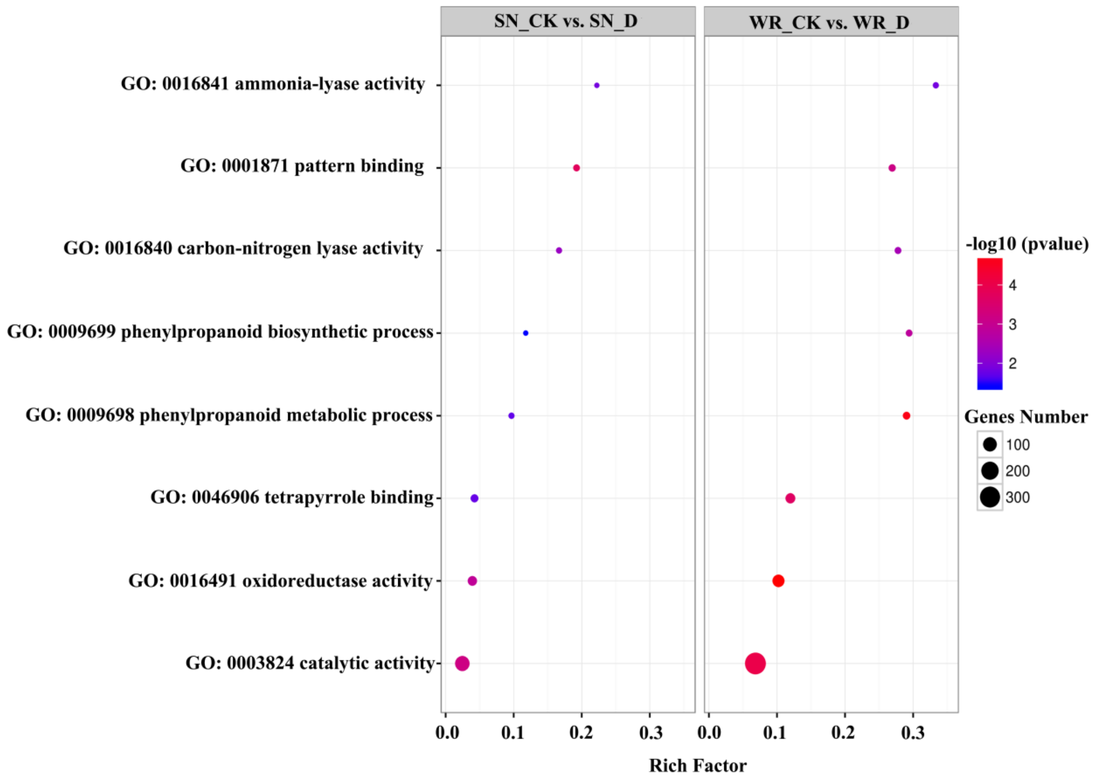

Figure S1 Gene ontology analysis of similar GO terms between WR04-6 and SN9816 after exposure to diniconazole. D in WR_D or SN_D indicates diniconazole treatment, and CK in WR_CK or SN_CK indicates the control group without treatment.

Table S1. Summary details of the RNA sequencing results for eighteen leaf samples of WR04-6 and SN9816 after ABA and diniconazole treatment.

Group	Samples	Raw reads	Clean reads	Total mapped (%)	GC (%)	Q20 (%)	Q30 (%)
WR_CK	WR_CK_1	53549036	53422588	96.7	55.45	97.82	93.88
	WR_CK_2	47926634	47846308	96.85	54.89	97.95	94.01
	WR_CK_3	53769818	53644604	96.65	54.02	97.81	93.85
WR_A	WR_A_1	47310438	47207728	97.02	55.15	97.75	93.59
	WR_A_2	56375100	56241814	96.88	53.82	97.92	94.21
	WR_A_3	65621634	65486240	97.06	55.52	97.99	94.36
WR_D	WR_D_1	58872000	58746030	96.53	54.06	97.98	94.33
	WR_D_2	55722280	55598808	96.71	53.92	98.11	94.64
	WR_D_3	53498312	53385098	96.43	55.07	97.83	93.94
SN_CK	SN_CK_1	43769394	43690262	96.84	54.47	98.06	94.49
	SN_CK_2	37780812	37695718	96.42	53.85	97.85	94.03
	SN_CK_3	52919878	52794934	96.54	53.86	97.56	93.23
SN_A	SN_A_1	60520142	60393278	97.06	55.7	97.88	93.95
	SN_A_2	45169776	45083542	96.59	54.52	98.01	94.34
	SN_A_3	66824966	66701164	97.39	55.87	97.78	93.67
SN_D	SN_D_1	54921596	54799182	96.73	54.18	97.82	93.89
	SN_D_2	58494748	58372434	96.82	54.06	97.89	94.12
	SN_D_3	53510914	53405292	97.02	54.92	97.84	93.79

Table S2. Statistical data of total differentially expressed genes (DEGs) for WR_CK vs. WR_A.

id	WR_CK _mean	WR_A _mean	log2(fc)	PValue	FDR	Symbol
LOC_Os03g25490	0.1400	4.4067	4.9762	9.00E-34	2.49E-29	CYP734A1
LOC_Os01g22249	24.1833	2.9767	-3.0222	3.55E-29	4.92E-25	PER1
LOC_Os01g02930	0.6933	8.0467	3.5368	5.27E-28	4.87E-24	pomgnt2
LOC_Os04g41640	23.8800	153.9500	2.6886	1.21E-26	8.42E-23	Cht4
LOC_Os04g34290	6.2500	0.4767	-3.7128	1.93E-25	1.07E-21	At2g19130
LOC_Os02g54140	0.5800	9.2200	3.9906	7.07E-24	3.27E-20	HSP18.6
LOC_Os01g73250	297.1500	32.3567	-3.1991	4.09E-22	1.62E-18	ASR2
LOC_Os08g26230	34.9133	1.1000	-4.9882	9.08E-22	3.15E-18	-
LOC_Os06g21240	4.2600	34.2800	3.0084	1.78E-19	5.47E-16	-
LOC_Os04g41620	60.1367	291.0067	2.2747	1.62E-18	4.43E-15	Cht4
LOC_Os03g16920	0.4167	9.6833	4.5385	1.76E-18	4.43E-15	HSP70
LOC_Os12g43450	15.8867	110.6933	2.8007	5.23E-18	1.21E-14	tlp
LOC_Os04g41680	18.6000	86.5567	2.2183	7.78E-18	1.66E-14	Cht5
LOC_Os11g24070	8.0067	89.0867	3.4759	2.11E-16	4.18E-13	LTP
LOC_Os01g62260	0.1300	6.9867	5.7480	3.26E-16	6.03E-13	-
LOC_Os11g41034	28.5433	3.2267	-3.1450	1.20E-15	2.09E-12	-
LOC_Os03g60580	31.3467	353.7867	3.4965	2.22E-15	3.62E-12	ADF3
LOC_Os06g13720	0.9067	13.8733	3.9356	8.17E-15	1.24E-11	Os06g0246500
LOC_Os01g54030	0.4867	9.3133	4.2583	8.51E-15	1.24E-11	-
ChrSy.fgenesh.gene.14	1.8600	0.0010	-10.8611	1.04E-14	1.44E-11	WAK3
LOC_Os11g46900	2.1567	0.0010	-11.0746	1.11E-14	1.46E-11	WAK5
LOC_Os01g02780	6.7633	0.0700	-6.5942	1.16E-14	1.46E-11	At1g67000
LOC_Os04g31710	0.2433	6.4133	4.7201	1.21E-14	1.46E-11	-
LOC_Os01g12580	0.3000	5.8300	4.2805	3.44E-14	3.97E-11	LEA14-A
LOC_Os02g13800	1.1600	12.0500	3.3768	8.12E-14	9.00E-11	HSFC2A
LOC_Os01g24710	5.3733	88.5300	4.0423	1.51E-13	1.61E-10	SALT
LOC_Os06g17000	0.0300	5.5433	7.5296	2.22E-13	2.28E-10	BURP10
LOC_Os08g15149	27.8200	1.1267	-4.6260	3.25E-13	3.17E-10	-
LOC_Os09g17560	0.1733	3.9867	4.5236	3.32E-13	3.17E-10	ZRP4
LOC_Os05g50260	0.1000	1.9867	4.3123	3.43E-13	3.17E-10	At1g48100
LOC_Os02g04780	0.1400	5.1467	5.2001	4.31E-13	3.86E-10	-
LOC_Os02g18070	1.6200	0.0500	-5.0179	5.58E-13	4.84E-10	RGA2
LOC_Os12g32760	0.7733	22.7833	4.8807	1.50E-12	1.26E-09	PLT5
LOC_Os12g17160	2.5867	22.7867	3.1390	2.93E-12	2.35E-09	SOT5
LOC_Os06g48210	1.1433	14.1233	3.6268	2.97E-12	2.35E-09	Os06g0697200
LOC_Os02g30100	2.4833	22.9000	3.2050	3.40E-12	2.62E-09	CYP81E7
LOC_Os07g14740	48.8300	5.0333	-3.2782	3.85E-12	2.89E-09	-
LOC_Os11g47550	0.1233	7.9400	6.0085	4.17E-12	3.05E-09	Chib3H-h
LOC_Os10g28080	9.9800	57.2533	2.5202	4.41E-12	3.13E-09	-

LOC_Os02g16995	2.9967	0.4667	-2.6829	5.85E-12	3.97E-09	-
LOC_Os01g68740	11.8267	0.9733	-3.6030	5.87E-12	3.97E-09	-
LOC_Os03g19290	3.2833	32.8800	3.3240	6.03E-12	3.98E-09	OEP162
LOC_Os02g47390	0.8167	33.3333	5.3511	7.01E-12	4.52E-09	-
LOC_Os06g09660	2.3700	8.3733	1.8209	8.62E-12	5.43E-09	ARF16
LOC_Os01g52240	0.0010	40.3500	15.3003	1.05E-11	6.48E-09	CAB2R
LOC_Os09g04339	2.8133	37.2067	3.7252	1.21E-11	7.27E-09	-
LOC_Os06g46149	54.0333	4.0733	-3.7296	1.32E-11	7.80E-09	-
MSTRG.17789	0.0767	5.6933	6.2145	1.41E-11	8.14E-09	--
LOC_Os06g46900	2.2067	14.1633	2.6822	2.16E-11	1.22E-08	comA
LOC_Os09g02180	2.1400	32.9067	3.9427	2.43E-11	1.35E-08	-
LOC_Os12g27830	0.0400	3.4467	6.4291	3.49E-11	1.90E-08	HSD1
LOC_Os04g45810	16.5933	88.3133	2.4120	5.27E-11	2.81E-08	HOX22
LOC_Os09g19380	2.4967	0.0010	-11.2858	7.32E-11	3.83E-08	At1g51890
LOC_Os04g30240	7.3867	1.0833	-2.7694	9.55E-11	4.90E-08	WAK2
LOC_Os06g45710	1.7800	17.7800	3.3203	1.46E-10	7.36E-08	-
LOC_Os03g52370	2.5267	19.0500	2.9145	1.54E-10	7.63E-08	-
LOC_Os03g48760	0.2333	8.1333	5.1234	1.59E-10	7.71E-08	Os03g0694000
LOC_Os11g26790	32.7233	673.6200	4.3635	1.62E-10	7.73E-08	RAB21
LOC_Os05g44340	0.3633	3.7233	3.3572	1.74E-10	8.17E-08	CLPB1
LOC_Os12g03040	0.2900	1.8933	2.7068	1.79E-10	8.28E-08	ONAC010
LOC_Os11g37970	21.2867	87.1333	2.0333	1.93E-10	8.75E-08	PR4A
LOC_Os03g42830	1.1300	5.8100	2.3622	2.02E-10	9.02E-08	TT12
LOC_Os05g33130	0.6333	11.5500	4.1888	2.07E-10	9.13E-08	Cht2
LOC_Os12g03899	9.3967	31.3433	1.7379	2.17E-10	9.41E-08	ZIFL1
LOC_Os01g32780	20.1500	129.4267	2.6833	2.63E-10	1.12E-07	At3g01520
LOC_Os06g02040	0.8433	6.0067	2.8324	3.91E-10	1.64E-07	-
MSTRG.990	10.2833	2.6733	-1.9436	4.16E-10	1.72E-07	--
LOC_Os08g29760	0.4233	4.1667	3.2990	4.61E-10	1.88E-07	-
LOC_Os01g71710	8.2200	0.7200	-3.5131	5.42E-10	2.18E-07	BAT1
LOC_Os01g47300	0.2100	6.5733	4.9682	5.83E-10	2.31E-07	-
LOC_Os03g21710	2.5300	0.0767	-5.0444	6.84E-10	2.67E-07	WRKY70
LOC_Os03g02050	0.4100	9.2267	4.4921	7.58E-10	2.92E-07	LTP-2
LOC_Os09g19400	1.2400	0.0010	-10.2761	7.94E-10	3.02E-07	At1g51810
LOC_Os05g31890	2.0867	0.1467	-3.8306	8.32E-10	3.12E-07	-
LOC_Os03g12890	7.0233	53.8233	2.9380	8.51E-10	3.15E-07	BCAT5
LOC_Os04g52190	7.7600	0.9933	-2.9657	8.98E-10	3.25E-07	VSR7
LOC_Os08g15710	1.3033	0.0010	-10.3480	9.04E-10	3.25E-07	-
LOC_Os05g39770	2.5100	41.1000	4.0334	9.91E-10	3.52E-07	At3g08860
LOC_Os11g26760	8.6767	77.1800	3.1530	1.07E-09	3.77E-07	RAB16C
LOC_Os02g50700	1.3967	7.0567	2.3370	1.14E-09	3.96E-07	-
LOC_Os11g02480	3.3200	9.4567	1.5101	1.26E-09	4.31E-07	WRKY55
LOC_Os09g19390	1.9167	0.0010	-10.9044	1.30E-09	4.39E-07	At1g05700

LOC_Os12g37690	1.6967	14.9167	3.1362	1.32E-09	4.42E-07	MYB108
LOC_Os03g13140	1.5433	127.2700	6.3657	1.40E-09	4.61E-07	HB1
MSTRG.26079	0.6433	15.3967	4.5809	1.62E-09	5.27E-07	--
LOC_Os03g22680	1.7233	6.7067	1.9604	1.71E-09	5.53E-07	Rchy1
LOC_Os11g26750	0.3700	5.3633	3.8575	1.83E-09	5.84E-07	RAB16D
LOC_Os07g44430	0.1833	2.2700	3.6302	1.97E-09	6.21E-07	Os07g0638300
LOC_Os12g42070	6.3467	1.9667	-1.6902	2.24E-09	6.94E-07	WAK2
LOC_Os01g04380	0.6933	4.7200	2.7672	2.25E-09	6.94E-07	HSP16.9B
LOC_Os06g05320	4.6133	12.7800	1.4700	2.54E-09	7.73E-07	-
LOC_Os03g48750	0.5267	18.1667	5.1083	3.11E-09	9.38E-07	Os03g0693700
LOC_Os12g16540	8.4033	1.6700	-2.3311	3.33E-09	9.91E-07	WAK3
LOC_Os04g43170	0.1033	2.6167	4.6624	3.43E-09	1.01E-06	SOP1
LOC_Os03g16940	0.3867	8.2533	4.4158	4.26E-09	1.24E-06	-
LOC_Os04g40070	0.3833	2.0600	2.4260	4.53E-09	1.31E-06	At5g50170
LOC_Os04g22120	0.8667	0.0300	-4.8524	5.68E-09	1.62E-06	LECRK91
LOC_Os11g47530	0.0633	6.3033	6.6370	6.27E-09	1.77E-06	Chib3H-h
LOC_Os06g03560	44.2867	11.5100	-1.9440	6.58E-09	1.84E-06	OPT7
LOC_Os03g19600	0.0900	0.9567	3.4100	6.79E-09	1.88E-06	TRO
LOC_Os03g59320	20.6400	102.2533	2.3086	6.85E-09	1.88E-06	-
LOC_Os10g12190	0.4733	2.3967	2.3401	7.28E-09	1.98E-06	At3g30340
LOC_Os10g25040	0.9733	7.5000	2.9459	7.69E-09	2.05E-06	rccR
LOC_Os03g02260	2.7733	12.4367	2.1649	7.70E-09	2.05E-06	-
LOC_Os11g03300	0.3733	1.7500	2.2288	7.81E-09	2.06E-06	ONAC010
LOC_Os07g09050	0.4067	4.0833	3.3278	9.51E-09	2.49E-06	At5g03795
LOC_Os08g42610	15.5767	4.4067	-1.8216	1.09E-08	2.83E-06	PCMP-H33
LOC_Os12g09720	2.6633	0.1633	-4.0273	1.39E-08	3.56E-06	GOS9
LOC_Os03g20020	7.4067	18.9133	1.3525	1.42E-08	3.61E-06	NIN1
LOC_Os03g52390	4.8400	44.3667	3.1964	1.45E-08	3.65E-06	-
LOC_Os01g64000	0.0333	1.9467	5.8679	1.52E-08	3.80E-06	ABI5
LOC_Os07g05365	0.8767	9.0300	3.3646	1.59E-08	3.92E-06	PSBR
LOC_Os01g01610	1.7000	6.9400	2.0294	1.60E-08	3.92E-06	At2g16710
LOC_Os03g12790	9.5000	1.9400	-2.2919	1.66E-08	4.03E-06	DTXL2
LOC_Os11g19864	1.7667	23.1900	3.7144	1.80E-08	4.34E-06	GSTT3
LOC_Os11g05410	0.8467	9.0700	3.4212	1.89E-08	4.51E-06	PAP20
LOC_Os01g71720	34.8633	8.0833	-2.1087	2.00E-08	4.75E-06	BAT1
LOC_Os06g12290	0.2533	7.8833	4.9597	2.04E-08	4.79E-06	GSTU10
LOC_Os01g63210	13.6033	79.9400	2.5550	2.12E-08	4.93E-06	At3g10130
LOC_Os05g38530	2.7267	33.7067	3.6278	2.39E-08	5.53E-06	HSP70
LOC_Os03g04560	0.5267	0.0333	-3.9819	2.55E-08	5.85E-06	-
LOC_Os03g11910	1.2200	7.6767	2.6536	2.85E-08	6.49E-06	HSP70-8
LOC_Os04g12390	32.7633	9.8167	-1.7388	3.12E-08	7.02E-06	JAL19
LOC_Os07g25810	1.8667	10.2900	2.4627	3.23E-08	7.22E-06	Sbsn
LOC_Os12g14440	39.9733	3.8067	-3.3924	3.27E-08	7.26E-06	SALT

LOC_Os04g44830	3.2700	14.8900	2.1870	3.53E-08	7.77E-06	Os04g0530600
LOC_Os08g40680	0.4633	12.0033	4.6952	4.24E-08	9.25E-06	Chib3H-h
LOC_Os05g47730	0.2400	5.1433	4.4216	4.29E-08	9.29E-06	-
LOC_Os02g14760	15.0367	42.7200	1.5064	4.65E-08	9.99E-06	CID9
LOC_Os01g12660	0.0367	0.5500	3.9069	4.71E-08	1.00E-05	SPCC24B10.10c
LOC_Os11g26570	4.5433	112.8000	4.6339	4.87E-08	1.03E-05	DHN3
LOC_Os04g39300	1.4067	0.0010	-10.4581	5.22E-08	1.10E-05	-
MSTRG.27714	1.1067	7.0900	2.6796	5.27E-08	1.10E-05	--
LOC_Os02g51110	23.8867	7.8933	-1.5975	5.34E-08	1.10E-05	NIP2-1
LOC_Os03g44270	1.2800	0.2200	-2.5406	5.37E-08	1.10E-05	TMM
LOC_Os11g10510	0.2000	17.8033	6.4760	5.79E-08	1.18E-05	ADH2
LOC_Os01g52830	0.0333	1.7167	5.6865	6.31E-08	1.28E-05	-
LOC_Os05g46370	1.0367	4.5233	2.1254	6.38E-08	1.28E-05	BHLH87
LOC_Os06g48300	3.7833	25.0400	2.7265	6.41E-08	1.28E-05	Os06g0698300
LOC_Os01g52070	0.0100	0.8033	6.3279	6.49E-08	1.29E-05	Os01g0718700
LOC_Os02g15860	6.5900	156.6200	4.5708	6.60E-08	1.30E-05	-
LOC_Os12g26290	3.9467	31.1200	2.9791	6.85E-08	1.34E-05	DOX1
LOC_Os08g01370	3.0767	41.4333	3.7514	8.52E-08	1.65E-05	-
LOC_Os01g72140	2.0733	0.1467	-3.8213	8.97E-08	1.73E-05	HSP26-A
LOC_Os04g34170	15.6433	85.3533	2.4479	9.28E-08	1.77E-05	FP1
LOC_Os07g44060	1.7100	13.1633	2.9445	9.35E-08	1.78E-05	-
LOC_Os01g68290	0.1733	7.6933	5.4720	1.05E-07	1.99E-05	-
LOC_Os09g09510	1.1033	0.1033	-3.4165	1.12E-07	2.10E-05	LECRK41
LOC_Os05g33630	389.0733	136.6667	-1.5094	1.27E-07	2.37E-05	rihA
LOC_Os01g06310	1.1567	51.3833	5.4733	1.47E-07	2.71E-05	-
LOC_Os03g17790	3.3633	48.2567	3.8428	1.52E-07	2.80E-05	LT101.2
LOC_Os05g23924	0.0500	0.6800	3.7655	1.56E-07	2.85E-05	xlnA
LOC_Os07g25800	0.8267	13.8567	4.0671	1.57E-07	2.85E-05	At2g05970
LOC_Os10g35050	0.1333	2.2700	4.0896	1.67E-07	3.00E-05	TIP3-1
LOC_Os12g38760	2.3600	0.5267	-2.1638	1.70E-07	3.04E-05	PAP1
LOC_Os01g50910	3.4500	45.7900	3.7304	1.75E-07	3.11E-05	LEA3
LOC_Os02g17500	37.8700	108.4033	1.5173	1.80E-07	3.17E-05	At1g67300
LOC_Os04g49980	1.4033	30.2067	4.4279	1.84E-07	3.23E-05	-
LOC_Os02g56860	1.2267	10.8933	3.1506	1.96E-07	3.41E-05	KCS12
LOC_Os05g08970	1.4100	4.3367	1.6209	2.01E-07	3.47E-05	SSRP1-B
LOC_Os03g45960	2.0400	10.0800	2.3049	2.01E-07	3.47E-05	Zlp
LOC_Os12g38270	14.7833	77.8933	2.3975	2.14E-07	3.67E-05	MT4A
LOC_Os11g47500	4.3700	161.7667	5.2101	2.19E-07	3.72E-05	RIXI
LOC_Os04g44100	0.5633	3.4267	2.6047	2.26E-07	3.79E-05	-
LOC_Os08g43120	0.1467	0.9600	2.7105	2.26E-07	3.79E-05	ABCG45
LOC_Os06g51060	29.4700	297.3033	3.3346	2.49E-07	4.15E-05	Cht1
LOC_Os08g01910	0.8833	8.2367	3.2210	2.58E-07	4.29E-05	-
LOC_Os02g30910	0.4000	5.9400	3.8924	2.67E-07	4.40E-05	SWEET15

LOC_Os05g43690	0.2967	2.1433	2.8529	2.73E-07	4.49E-05	PDCB3
LOC_Os11g04104	5.0900	16.9333	1.7341	2.83E-07	4.62E-05	ZIFL1
LOC_Os03g46454	1.1333	22.7600	4.3279	2.86E-07	4.63E-05	IRT2
LOC_Os10g41749	1.9600	5.7900	1.5627	2.87E-07	4.63E-05	SPAC977.11
LOC_Os08g43020	0.0333	2.9933	6.4886	2.95E-07	4.72E-05	RAS
MSTRG.23921	0.1967	3.3900	4.1075	2.96E-07	4.72E-05	--
LOC_Os04g27670	0.2100	16.4833	6.2945	2.99E-07	4.74E-05	-
LOC_Os01g48620	0.8733	0.0967	-3.1754	3.07E-07	4.84E-05	-
LOC_Os09g25880	23.6333	6.6233	-1.8352	3.18E-07	4.99E-05	AAP3
LOC_Os02g42190	4.9033	0.2967	-4.0468	3.46E-07	5.39E-05	WAK4
LOC_Os11g37950	7.9633	120.2000	3.9159	3.54E-07	5.48E-05	PR4A
LOC_Os09g19140	4.1167	1.0000	-2.0415	4.01E-07	6.18E-05	At1g51810
LOC_Os04g52110	0.4833	4.7333	3.2918	4.25E-07	6.51E-05	-
LOC_Os01g48610	4.5967	0.7167	-2.6812	4.29E-07	6.52E-05	-
LOC_Os12g42910	0.3533	3.8433	3.4433	4.30E-07	6.52E-05	CCX1
LOC_Os02g46030	3.2067	18.4900	2.5276	4.43E-07	6.67E-05	RVE1
LOC_Os10g39130	59.8000	7.3067	-3.0329	4.60E-07	6.88E-05	MADS56
LOC_Os07g48550	8.7367	32.6833	1.9034	4.62E-07	6.88E-05	NAC100
LOC_Os01g04120	7.1667	1.9700	-1.8631	4.77E-07	7.07E-05	ZFP6
LOC_Os01g12820	0.3700	3.8033	3.3617	4.97E-07	7.30E-05	-
LOC_Os10g13850	3.6867	121.0133	5.0367	4.99E-07	7.30E-05	-
LOC_Os01g71624	9.9600	146.1933	3.8756	5.00E-07	7.30E-05	-
MSTRG.21766	0.2200	3.2867	3.9010	5.04E-07	7.32E-05	--
LOC_Os01g03390	103.4300	552.5333	2.4174	5.32E-07	7.68E-05	RBBI3.3
LOC_Os04g49850	1.1000	5.7767	2.3927	5.52E-07	7.93E-05	-
LOC_Os02g42940	7.8267	36.1967	2.2094	5.64E-07	8.06E-05	PVA12
LOC_Os04g27190	0.0367	1.9333	5.7205	6.10E-07	8.67E-05	ZSS1
LOC_Os05g45090	0.7867	5.4100	2.7818	6.24E-07	8.83E-05	RhGT1
LOC_Os06g19095	0.1400	10.8500	6.2761	6.29E-07	8.86E-05	-
MSTRG.22038	0.3600	4.6467	3.6901	6.35E-07	8.89E-05	--
LOC_Os08g08840	19.5767	49.2400	1.3307	6.45E-07	8.99E-05	GPT2
LOC_Os03g62010	37.6867	7.7033	-2.2905	6.59E-07	9.13E-05	-
LOC_Os06g46740	12.8567	169.0400	3.7168	6.67E-07	9.20E-05	At4g27520
LOC_Os10g33240	0.8033	2.5033	1.6398	6.74E-07	9.25E-05	-
LOC_Os03g58610	6.1133	2.0967	-1.5439	6.85E-07	9.36E-05	-
LOC_Os04g01740	0.1100	0.8633	2.9724	7.12E-07	9.68E-05	HSP83A
LOC_Os10g33370	1.9133	33.4367	4.1273	7.16E-07	9.68E-05	KCS12
LOC_Os07g48460	5.1267	30.7300	2.5836	7.35E-07	9.89E-05	-
LOC_Os05g49300	6.1600	31.1700	2.3392	7.49E-07	0.0001	ISU1
LOC_Os03g61920	8.9767	28.1233	1.6475	7.58E-07	0.0001	ETFA
LOC_Os03g46060	3.9567	78.6467	4.3130	7.63E-07	0.0001	tlp
LOC_Os04g19740	0.6200	3.0567	2.3016	7.77E-07	0.0001	-
LOC_Os02g17710	1.5700	0.2033	-2.9488	8.10E-07	0.00011	At1g35710

LOC_Os01g74160	5.7000	0.3400	-4.0674	8.37E-07	0.00011	-
LOC_Os12g12080	2.7700	86.1933	4.9596	8.70E-07	0.00011	-
LOC_Os08g29040	2.4133	0.1300	-4.2144	8.82E-07	0.00011	WAK3
LOC_Os07g03560	0.1100	0.0010	-6.7814	8.93E-07	0.00012	TY3B-I
LOC_Os07g14820	0.3833	0.0010	-8.5825	9.75E-07	0.00013	At3g47570
LOC_Os12g05210	1.3867	19.4867	3.8128	9.79E-07	0.00013	-
LOC_Os12g38180	53.9800	14.9733	-1.8500	9.95E-07	0.00013	HSC-2
LOC_Os12g13380	6.2567	13.5167	1.1113	1.01E-06	0.00013	ADK-A
LOC_Os02g39660	1.3700	0.1333	-3.3611	1.01E-06	0.00013	EFR
LOC_Os09g31478	5.8033	61.6400	3.4089	1.01E-06	0.00013	-
LOC_Os03g21040	11.9267	56.2333	2.2372	1.02E-06	0.00013	-
LOC_Os11g47560	0.6467	40.1533	5.9564	1.13E-06	0.00014	Chib3H-h
LOC_Os11g04720	48.6700	8.0733	-2.5918	1.15E-06	0.00014	ARR9
LOC_Os06g34970	5.6400	0.4433	-3.6692	1.16E-06	0.00014	CNR13
LOC_Os11g26780	5.9867	112.5667	4.2329	1.22E-06	0.00015	RAB16B
LOC_Os12g09220	3.7433	1.0500	-1.8339	1.24E-06	0.00015	-
LOC_Os01g06560	0.2067	8.0200	5.2782	1.25E-06	0.00015	TGA6
LOC_Os06g45184	2.2633	58.0700	4.6813	1.28E-06	0.00015	-
LOC_Os05g31670	0.8867	14.8133	4.0624	1.36E-06	0.00016	-
LOC_Os03g17800	3.1600	11.3433	1.8438	1.37E-06	0.00016	rdgBbeta
LOC_Os08g26350	0.8367	0.0010	-9.7085	1.39E-06	0.00017	-
LOC_Os06g44300	0.4433	2.0933	2.2393	1.42E-06	0.00017	CER3
LOC_Os10g30010	0.1233	1.8200	3.8833	1.46E-06	0.00017	-
LOC_Os08g02030	1.2000	21.2267	4.1448	1.48E-06	0.00017	At3g50280
LOC_Os03g51350	4.1300	45.3900	3.4582	1.49E-06	0.00017	-
LOC_Os01g51210	3.7567	12.0867	1.6859	1.52E-06	0.00018	LOGL1
LOC_Os07g43950	4.4233	28.5533	2.6905	1.54E-06	0.00018	SCL30A
LOC_Os10g28120	13.9267	55.6267	1.9979	1.55E-06	0.00018	-
LOC_Os01g58194	13.5467	46.1467	1.7683	1.60E-06	0.00018	Os01g0794400
LOC_Os04g50200	0.1267	1.1933	3.2359	1.66E-06	0.00019	-
LOC_Os11g47520	0.0200	3.5433	7.4690	1.70E-06	0.00019	Chib3H-h
LOC_Os04g48200	0.3967	7.0433	4.1503	1.70E-06	0.00019	CYP87A3
MSTRG.10545	0.7600	20.3667	4.7441	1.71E-06	0.00019	--
LOC_Os09g21919	0.0333	0.8133	4.6088	1.73E-06	0.0002	-
LOC_Os07g23640	0.5700	3.5667	2.6455	1.76E-06	0.0002	-
LOC_Os06g35940	36.8900	5.7567	-2.6799	1.77E-06	0.0002	HD3A
LOC_Os04g57140	5.1033	14.7700	1.5332	1.81E-06	0.0002	Os04g0666900
MSTRG.26475	2.7767	0.2833	-3.2928	1.89E-06	0.00021	--
LOC_Os11g20090	0.4067	7.4633	4.1979	1.95E-06	0.00022	OMT2
LOC_Os01g58670	4.3733	80.3933	4.2003	1.97E-06	0.00022	NSFBx
MSTRG.3995	0.0700	2.7933	5.3185	2.11E-06	0.00023	--
LOC_Os10g21590	0.9933	10.1500	3.3531	2.12E-06	0.00023	PLT6
LOC_Os11g45930	1.7233	0.3967	-2.1192	2.17E-06	0.00024	RXW24L

LOC_Os12g21789	6.8267	18.2233	1.4165	2.30E-06	0.00025	-
LOC_Os09g09930	16.4500	67.2000	2.0304	2.37E-06	0.00026	-
LOC_Os03g19427	2.7333	159.7967	5.8694	2.41E-06	0.00026	NAS1
LOC_Os03g59600	1.3800	0.1833	-2.9121	2.56E-06	0.00027	MIRO1
LOC_Os01g58640	1.0533	4.9767	2.2402	2.58E-06	0.00028	PAP27
LOC_Os02g51750	25.7933	89.2333	1.7906	2.69E-06	0.00029	ANNAT7
LOC_Os12g13270	2.5167	0.4567	-2.4623	2.72E-06	0.00029	-
LOC_Os02g38290	0.0267	0.7967	4.9009	2.72E-06	0.00029	CYP86B1
LOC_Os11g02389	1180.5167	3140.9033	1.4118	2.79E-06	0.00029	LTP
LOC_Os01g40280	1.8767	8.7700	2.2244	2.82E-06	0.0003	SPBC776.05
LOC_Os12g39360	20.4467	1.0467	-4.2880	2.83E-06	0.0003	nep1
LOC_Os11g19850	3.6267	40.7600	3.4904	2.85E-06	0.0003	-
LOC_Os01g62290	2.5233	33.4767	3.7298	2.90E-06	0.0003	HSP70
LOC_Os12g18410	7.6333	203.4400	4.7361	3.03E-06	0.00031	-
LOC_Os06g06560	1.1967	4.5767	1.9353	3.13E-06	0.00032	Os06g0160700
LOC_Os02g45860	17.8567	2.7100	-2.7201	3.19E-06	0.00033	-
LOC_Os05g31020	2.8300	36.2633	3.6796	3.22E-06	0.00033	ERF1-2
LOC_Os09g19280	8.0133	1.0233	-2.9691	3.25E-06	0.00033	RPM1
LOC_Os01g02790	8.1667	1.4267	-2.5171	3.25E-06	0.00033	At5g39030
LOC_Os05g24770	0.1000	2.5333	4.6630	3.25E-06	0.00033	RTNLB1
LOC_Os04g48416	0.5433	2.2633	2.0585	3.28E-06	0.00033	SBT1.7
LOC_Os07g10600	8.9800	25.5267	1.5072	3.55E-06	0.00036	Smt1-1
LOC_Os01g45640	8.2633	62.8967	2.9282	3.59E-06	0.00036	-
LOC_Os05g50380	1.7867	7.8133	2.1287	3.71E-06	0.00037	AGP-L
LOC_Os05g25430	2.0600	0.7467	-1.4641	3.90E-06	0.00039	FER
LOC_Os12g43490	1.0033	5.2667	2.3921	3.92E-06	0.00039	tlp
LOC_Os03g04770	27.1400	118.9233	2.1315	3.98E-06	0.00039	BAM1
LOC_Os12g42220	16.4500	5.3367	-1.6241	4.44E-06	0.00044	-
LOC_Os01g62900	2.3033	6.5400	1.5056	4.44E-06	0.00044	P5CS
LOC_Os01g28450	11.5933	1.0367	-3.4833	4.73E-06	0.00046	-
LOC_Os03g36279	0.2033	9.3267	5.5194	4.85E-06	0.00047	GIN1
LOC_Os06g29730	19.9867	2.0600	-3.2783	4.93E-06	0.00048	-
LOC_Os02g07160	11.6733	59.2500	2.3436	4.96E-06	0.00048	-
LOC_Os01g41780	1.0333	0.2367	-2.1264	5.19E-06	0.0005	BRI1
LOC_Os02g44770	0.3400	1.3367	1.9750	5.23E-06	0.0005	MSL8
LOC_Os10g42490	2.2133	0.7067	-1.6471	5.32E-06	0.00051	ROC3
LOC_Os01g36790	1.3767	10.6967	2.9579	5.35E-06	0.00051	CRK2
LOC_Os01g42320	0.0533	0.5567	3.3837	5.42E-06	0.00051	ASPG1
LOC_Os03g03070	22.6000	8.2300	-1.4574	5.42E-06	0.00051	MADS50
LOC_Os06g40180	7.0400	0.6733	-3.3862	5.46E-06	0.00051	PLD2
LOC_Os06g09560	0.9300	4.4567	2.2607	5.48E-06	0.00051	dnaJ
LOC_Os02g54180	0.6467	8.1833	3.6616	5.52E-06	0.00052	-
LOC_Os03g18130	7.5133	77.3300	3.3635	5.53E-06	0.00052	Os03g0291500

LOC_Os07g20340	0.0467	4.8367	6.6955	5.58E-06	0.00052	HIPP26
LOC_Os01g03680	5.1833	0.2567	-4.3359	5.67E-06	0.00053	RBBI3.3
LOC_Os01g72810	0.8267	14.7500	4.1573	5.71E-06	0.00053	EP1
LOC_Os09g04370	0.7967	15.8400	4.3135	5.85E-06	0.00054	-
LOC_Os03g60370	4.6533	21.3033	2.1947	5.86E-06	0.00054	mipp1
LOC_Os02g43410	0.1400	6.9500	5.6335	5.91E-06	0.00054	YSL15
LOC_Os01g28500	154.2000	319.8533	1.0526	5.92E-06	0.00054	PRMS
LOC_Os02g02930	3.9167	0.0833	-5.5546	5.94E-06	0.00054	-
LOC_Os03g61160	6.9100	89.0467	3.6878	6.04E-06	0.00055	-
LOC_Os06g38764	30.0833	4.5000	-2.7410	6.13E-06	0.00055	-
LOC_Os07g22390	10.6100	3.1000	-1.7751	6.16E-06	0.00055	SGR2
LOC_Os08g40250	11.4033	25.4433	1.1578	6.25E-06	0.00056	PPI1
LOC_Os03g27980	0.5667	5.7300	3.3380	6.27E-06	0.00056	At4g31140
LOC_Os04g50700	1.6967	16.5500	3.2861	6.28E-06	0.00056	-
LOC_Os05g39320	0.3067	3.6800	3.5850	6.30E-06	0.00056	PDC1
LOC_Os08g34500	0.1633	3.0667	4.2308	6.31E-06	0.00056	-
LOC_Os11g19840	8.6633	60.9900	2.8156	6.38E-06	0.00056	OMT2
LOC_Os11g40930	4.5200	0.3000	-3.9133	6.49E-06	0.00057	-
LOC_Os10g35070	77.8900	296.0533	1.9263	6.57E-06	0.00058	AGAL1
LOC_Os07g31960	0.2367	1.4067	2.5714	6.58E-06	0.00058	RhGT1
LOC_Os08g29600	4.1200	16.5767	2.0084	6.75E-06	0.00059	-
LOC_Os12g16720	8.3933	30.1367	1.8442	6.96E-06	0.00061	CYP71A9
LOC_Os09g29690	1.9267	21.9300	3.5087	7.37E-06	0.00064	CjBAp12
LOC_Os03g07200	1.1367	5.4300	2.2561	7.43E-06	0.00064	-
LOC_Os05g46480	28.4200	500.9567	4.1397	7.46E-06	0.00064	LEA3
LOC_Os02g37480	1.3700	21.3933	3.9649	7.57E-06	0.00065	-
LOC_Os02g15950	2.3633	7.1233	1.5917	7.74E-06	0.00066	At3g61590
LOC_Os01g71740	0.4467	1.4733	1.7218	8.08E-06	0.00069	BAT1
LOC_Os06g34960	1.8767	0.1400	-3.7447	8.14E-06	0.00069	At1g67520
LOC_Os05g32460	4.5300	0.8100	-2.4835	8.16E-06	0.00069	SIB1
LOC_Os09g29710	1.0267	22.4800	4.4526	8.26E-06	0.0007	CjBAp12
LOC_Os01g27449	0.7967	3.8233	2.2628	8.43E-06	0.00071	-
LOC_Os03g30950	0.4333	5.7800	3.7375	8.75E-06	0.00074	Os03g0423300
LOC_Os11g05800	4.5433	0.5533	-3.0375	8.79E-06	0.00074	HVA22J
LOC_Os12g43720	2.5867	14.6967	2.5063	8.92E-06	0.00074	RXW8
LOC_Os01g57370	0.0010	0.2333	7.8662	8.95E-06	0.00074	CNGC4
LOC_Os10g03570	1.1833	0.2367	-2.3219	8.96E-06	0.00074	RPM1
LOC_Os07g02850	0.4800	3.9533	3.0420	8.97E-06	0.00074	-
LOC_Os07g26550	0.4233	2.5767	2.6056	8.97E-06	0.00074	-
LOC_Os04g48390	4.6400	25.5467	2.4609	9.01E-06	0.00074	Os04g0573000
LOC_Os01g16170	11.7233	74.9000	2.6756	9.02E-06	0.00074	YPQ1
LOC_Os01g65110	5.5067	74.4367	3.7568	9.33E-06	0.00076	NPF5.10
LOC_Os06g14030	12.9767	34.6633	1.4175	9.34E-06	0.00076	Os06g0250600

LOC_Os02g54254	7.8867	78.9467	3.3234	9.57E-06	0.00078	LKR/SDH
LOC_Os10g42040	2.6600	0.1300	-4.3548	9.60E-06	0.00078	-
LOC_Os04g33460	16.2300	36.4833	1.1686	1.03E-05	0.00083	SBE2.2
LOC_Os07g19320	0.0010	0.1633	7.3517	1.07E-05	0.00086	RPM1
LOC_Os02g58100	0.2900	11.3400	5.2892	1.09E-05	0.00087	-
LOC_Os02g56380	5.0800	1.3733	-1.8871	1.09E-05	0.00087	WAK3
LOC_Os09g09490	1.1600	0.3867	-1.5850	1.09E-05	0.00087	RPM1
LOC_Os07g44330	14.7267	119.2667	3.0177	1.11E-05	0.00088	PDK
LOC_Os10g37660	1.1533	8.6167	2.9013	1.12E-05	0.00089	Os10g0521000
LOC_Os02g46860	1.3133	4.1600	1.6634	1.14E-05	0.0009	OPT4
LOC_Os07g46990	46.4233	153.1867	1.7224	1.15E-05	0.0009	SODCC2
LOC_Os01g27230	16.9033	1.9800	-3.0937	1.16E-05	0.00091	OPR1
LOC_Os01g14850	0.0800	3.4400	5.4263	1.23E-05	0.00096	MFS18
LOC_Os10g04540	0.0633	0.7300	3.5269	1.24E-05	0.00097	-
LOC_Os01g14630	9.0000	0.5000	-4.1699	1.25E-05	0.00098	GGPPS1
LOC_Os08g02460	6.1733	13.1967	1.0961	1.27E-05	0.00099	-
LOC_Os07g33580	0.5333	2.2167	2.0553	1.27E-05	0.00099	CYP716B1
LOC_Os12g16080	5.1800	16.4733	1.6691	1.30E-05	0.001	-
LOC_Os11g34920	8.1733	2.7800	-1.5558	1.31E-05	0.00101	RPM1
LOC_Os01g55540	33.2767	67.7433	1.0256	1.32E-05	0.00101	Os01g0760600
LOC_Os08g04240	0.0367	0.9067	4.6280	1.32E-05	0.00101	CRRSP55
LOC_Os01g19770	0.0433	1.8167	5.3897	1.33E-05	0.00102	OEP162
LOC_Os07g35350	0.0300	1.1367	5.2437	1.35E-05	0.00103	At5g56590
LOC_Os09g07180	0.3500	4.3400	3.6323	1.36E-05	0.00103	-
LOC_Os03g48780	52.1133	177.6367	1.7692	1.37E-05	0.00103	Os03g0694000
LOC_Os01g66760	6.7167	0.6667	-3.3327	1.39E-05	0.00105	At5g48380
LOC_Os06g47200	19.1200	46.8033	1.2915	1.39E-05	0.00105	VAS
LOC_Os01g07110	0.1700	0.5333	1.6495	1.40E-05	0.00105	BRCA2B
LOC_Os06g01630	12.5400	25.9800	1.0509	1.41E-05	0.00105	At1g54220
LOC_Os02g11760	4.4900	18.8567	2.0703	1.44E-05	0.00107	ABCG39
LOC_Os03g03730	17.0300	7.0533	-1.2717	1.44E-05	0.00107	agtA
LOC_Os01g02800	3.0400	1.1867	-1.3572	1.44E-05	0.00107	At1g67000
LOC_Os01g43370	1.6967	4.1900	1.3042	1.46E-05	0.00108	WRKY19
LOC_Os02g35940	3.1467	12.6667	2.0091	1.49E-05	0.0011	TCEA2
LOC_Os02g10290	11.3567	23.6700	1.0595	1.50E-05	0.00111	HMA5
LOC_Os03g62070	0.0400	0.4800	3.5850	1.55E-05	0.00114	ILL4
LOC_Os05g13770	5.5800	2.2233	-1.3275	1.56E-05	0.00114	LECRK43
LOC_Os03g50490	0.0667	1.8467	4.7918	1.58E-05	0.00115	GLN1-3
LOC_Os02g43820	2.6700	11.0000	2.0426	1.59E-05	0.00116	ERF5
LOC_Os03g51760	0.0300	0.6900	4.5236	1.60E-05	0.00116	-
LOC_Os10g42030	4.5767	1.0100	-2.1799	1.67E-05	0.00121	-
LOC_Os02g15930	0.2967	2.4100	3.0221	1.70E-05	0.00122	BAG6
LOC_Os03g10320	4.2400	14.7067	1.7943	1.72E-05	0.00123	-

LOC_Os04g47250	0.3500	2.5933	2.8894	1.77E-05	0.00127	CYP86A4
LOC_Os10g02880	0.0900	2.4167	4.7469	1.78E-05	0.00127	ZRP4
LOC_Os07g48160	6.9667	20.0100	1.5222	1.78E-05	0.00127	AGAL3
LOC_Os05g05040	27.9367	3.1400	-3.1533	1.83E-05	0.0013	-
LOC_Os08g25870	0.1067	0.9033	3.0821	1.84E-05	0.00131	-
LOC_Os09g09650	0.7367	9.4033	3.6741	1.86E-05	0.00132	-
LOC_Os03g59030	0.0567	1.0367	4.1933	1.88E-05	0.00133	UGT91A1
LOC_Os12g32986	14.3400	31.8700	1.1522	1.97E-05	0.00138	-
LOC_Os09g37200	0.1633	1.2967	2.9889	1.99E-05	0.00139	ACT-2
LOC_Os11g05550	6.2767	1.3467	-2.2206	2.01E-05	0.00141	At5g22090
LOC_Os03g27280	15.6733	32.7800	1.0645	2.02E-05	0.00141	SAPK1
LOC_Os01g62810	0.5200	13.7633	4.7262	2.03E-05	0.00142	UVR8
LOC_Os02g51930	5.6967	57.6667	3.3395	2.04E-05	0.00142	UGT85A24
LOC_Os01g67420	2.3800	5.0600	1.0882	2.10E-05	0.00146	Daglb
LOC_Os10g26110	1.5333	0.0200	-6.2605	2.11E-05	0.00146	TYDC5
LOC_Os01g03510	7.0933	17.4000	1.2946	2.13E-05	0.00147	-
LOC_Os08g08970	0.3500	4.2700	3.6088	2.14E-05	0.00147	GER2
LOC_Os01g14640	4.7067	1.4833	-1.6659	2.14E-05	0.00147	-
MSTRG.29114	0.3733	4.9133	3.7182	2.16E-05	0.00148	--
LOC_Os11g31530	1.0600	0.0010	-10.0498	2.18E-05	0.00149	SERK1
LOC_Os04g12530	2.2233	18.0633	3.0223	2.28E-05	0.00155	-
LOC_Os01g08440	5.1500	50.4867	3.2933	2.30E-05	0.00156	UGT75L6
LOC_Os07g31190	0.8167	0.1033	-2.9824	2.34E-05	0.00159	WAK2
LOC_Os01g72210	0.3933	1.4367	1.8689	2.36E-05	0.0016	At3g54510
MSTRG.4664	1.1033	0.0233	-5.5633	2.37E-05	0.0016	--
LOC_Os03g12510	45.8500	705.3500	3.9433	2.38E-05	0.0016	HB2
MSTRG.8902	1.9367	0.2600	-2.8970	2.41E-05	0.00162	--
LOC_Os12g33130	25.1200	2.4233	-3.3738	2.42E-05	0.00162	-
LOC_Os10g11500	0.0800	2.1400	4.7415	2.43E-05	0.00162	-
LOC_Os06g11980	3.9367	12.6233	1.6810	2.44E-05	0.00162	-
LOC_Os12g36940	3.7100	0.8767	-2.0813	2.44E-05	0.00162	CBP60B
LOC_Os11g37260	2.4933	6.5500	1.3934	2.51E-05	0.00166	Os11g0582300
LOC_Os04g31720	0.0367	1.2733	5.1180	2.54E-05	0.00168	-
LOC_Os12g12000	5.3700	1.4500	-1.8889	2.64E-05	0.00174	-
LOC_Os11g35850	1.4233	0.2267	-2.6506	2.67E-05	0.00176	GSO1
LOC_Os06g51050	2.1233	32.7833	3.9486	2.79E-05	0.00183	Cht3
LOC_Os07g06800	2.0267	23.8367	3.5560	2.80E-05	0.00184	gpsn2
MSTRG.7333	0.3467	0.0010	-8.4374	2.82E-05	0.00184	WAK1
LOC_Os11g41650	0.3300	1.9133	2.5356	2.85E-05	0.00186	APK3
LOC_Os12g15530	0.4567	7.6967	4.0750	2.86E-05	0.00186	LAC25
LOC_Os12g33120	830.5367	248.0700	-1.7433	2.87E-05	0.00186	-
LOC_Os01g55610	0.8000	0.1200	-2.7370	2.92E-05	0.00189	NPF1.2
LOC_Os01g09460	1.2267	7.1400	2.5412	2.93E-05	0.0019	HXK8

LOC_Os08g32850	23.4767	51.1800	1.1244	3.08E-05	0.00199	MCCB
LOC_Os05g47780	10.1800	89.0133	3.1283	3.12E-05	0.00201	SPAC2F3.16
LOC_Os01g45659	0.2100	3.7567	4.1610	3.27E-05	0.0021	-
LOC_Os03g40600	0.5267	0.0010	-9.0407	3.28E-05	0.0021	CYP78A4
LOC_Os02g49720	8.4933	44.6500	2.3943	3.39E-05	0.00217	ALDH2B7
LOC_Os12g07310	1.2967	11.6167	3.1633	3.43E-05	0.00219	CBP
LOC_Os03g48770	19.1367	71.9400	1.9105	3.48E-05	0.00221	Os03g0693900
LOC_Os08g42010	12.6233	29.8433	1.2413	3.53E-05	0.00224	NFD4
LOC_Os11g24510	9.0633	37.0400	2.0310	3.53E-05	0.00224	SCPL18
LOC_Os04g31290	0.2600	6.4867	4.6409	3.55E-05	0.00224	FIT
LOC_Os04g43200	19.3700	101.2367	2.3858	3.60E-05	0.00226	PXG
LOC_Os11g15340	3.8667	12.3367	1.6738	3.60E-05	0.00226	AAMT1
LOC_Os01g40499	3.3033	0.8800	-1.9083	3.61E-05	0.00226	At2g19130
LOC_Os06g19630	0.1467	1.0333	2.8167	3.66E-05	0.00229	At3g27390
LOC_Os05g39250	7.6667	69.7233	3.1850	3.69E-05	0.0023	MTH_273
LOC_Os04g07110	0.9400	0.1233	-2.9301	3.73E-05	0.00232	-
LOC_Os08g43334	1.0133	5.1133	2.3352	3.74E-05	0.00232	HSFB2B
LOC_Os04g44240	0.4100	2.1700	2.4040	3.75E-05	0.00233	UGT73C7
LOC_Os02g09810	14.1467	56.0133	1.9853	3.80E-05	0.00235	SLC38A1
LOC_Os03g19190	1.4667	3.2400	1.1435	3.85E-05	0.00237	At4g02110
LOC_Os04g24804	1.2433	10.3733	3.0606	3.89E-05	0.00239	-
LOC_Os08g40690	0.2100	9.8600	5.5531	3.89E-05	0.00239	RIXI
LOC_Os09g25690	0.0010	1.0967	10.0989	3.97E-05	0.00244	-
LOC_Os01g19290	0.0133	0.5800	5.4429	3.99E-05	0.00244	At1g09380
LOC_Os04g33150	0.0567	1.0933	4.2701	4.04E-05	0.00247	-
LOC_Os12g37350	68.1367	19.9100	-1.7749	4.06E-05	0.00248	LOX2.2
LOC_Os07g42714	27.3700	9.0800	-1.5918	4.20E-05	0.00255	-
LOC_Os09g39440	1.2467	4.5267	1.8604	4.31E-05	0.00262	URH2
LOC_Os09g35940	10.8567	2.8533	-1.9279	4.37E-05	0.00264	CYP78A6
LOC_Os02g48570	20.9400	127.6700	2.6081	4.55E-05	0.00275	NPF7.3
LOC_Os03g13250	4.6800	9.4967	1.0209	4.57E-05	0.00275	NPF8.3
LOC_Os11g32759	5.5433	1.6533	-1.7454	4.65E-05	0.0028	-
LOC_Os05g45350	8.5933	56.1900	2.7090	4.66E-05	0.0028	dnaJ
LOC_Os01g72460	5.9933	22.6800	1.9200	4.69E-05	0.00281	Os01g0954000
LOC_Os11g14080	0.0010	0.3033	8.2448	4.73E-05	0.00283	Hgsnat
LOC_Os01g53170	0.8433	0.0467	-4.1756	4.75E-05	0.00283	-
LOC_Os05g39310	0.0010	0.4967	8.9561	4.86E-05	0.00289	PDC1
LOC_Os03g41060	0.0200	0.7767	5.2792	4.87E-05	0.00289	At2g39540
LOC_Os12g11420	2.4367	0.0010	-11.2507	4.89E-05	0.0029	-
LOC_Os03g43410	3.6967	1.3967	-1.4042	4.92E-05	0.00291	IAA12
LOC_Os02g18150	4.5800	1.6600	-1.4642	5.04E-05	0.00297	Os02g0690500
LOC_Os01g33684	2.0967	4.4300	1.0792	5.06E-05	0.00298	RGA2
LOC_Os02g38920	28.2467	148.2000	2.3914	5.10E-05	0.00299	GAPC3

LOC_Os03g42280	1.9400	0.5100	-1.9275	5.18E-05	0.00304	Os03g0620400
LOC_Os03g01200	8.5267	3.7100	-1.2006	5.31E-05	0.00311	CHD1L
LOC_Os05g02450	2.1867	9.1067	2.0582	5.54E-05	0.00324	-
LOC_Os01g05770	0.4733	2.9933	2.6608	5.60E-05	0.00326	-
LOC_Os09g04360	1.1467	18.6667	4.0249	5.60E-05	0.00326	-
LOC_Os02g16680	8.3067	17.4733	1.0728	5.63E-05	0.00326	BZIP9
LOC_Os03g17230	2.1867	0.4067	-2.4268	5.63E-05	0.00326	UXS1
LOC_Os01g57580	4.3167	1.7633	-1.2916	5.74E-05	0.00332	BHLH123
LOC_Os02g28110	0.2967	3.9800	3.7459	5.94E-05	0.00342	-
LOC_Os04g48010	7.4400	17.9800	1.2730	5.95E-05	0.00342	WDR44
LOC_Os05g15880	1.0167	20.6833	4.3466	5.97E-05	0.00343	Chib3H-h
LOC_Os01g17396	0.4300	15.5900	5.1801	5.99E-05	0.00343	-
LOC_Os07g23660	0.1800	1.0267	2.5119	6.02E-05	0.00344	olpB
LOC_Os01g70590	0.0267	0.9100	5.0928	6.10E-05	0.00348	At3g14260
LOC_Os01g41550	21.8033	8.6400	-1.3354	6.18E-05	0.00352	ASPG1
LOC_Os04g43410	5.1667	19.8700	1.9433	6.30E-05	0.00357	BGLU18
LOC_Os01g66740	0.2900	0.0010	-8.1799	6.30E-05	0.00357	At5g48380
LOC_Os01g66710	0.1100	2.1533	4.2910	6.32E-05	0.00358	-
LOC_Os01g62790	3.9833	1.7133	-1.2172	6.36E-05	0.00359	cid1
LOC_Os11g06460	0.2300	0.0010	-7.8455	6.37E-05	0.00359	-
LOC_Os05g33644	3.2833	6.9800	1.0881	6.45E-05	0.00363	rihA
LOC_Os06g20790	0.5333	0.0600	-3.1520	6.52E-05	0.00366	AAMT1I
LOC_Os08g25720	28.3333	59.5033	1.0705	6.55E-05	0.00367	PFP-ALPHA
LOC_Os06g38340	4.4967	0.7433	-2.5968	6.59E-05	0.00368	At3g47570
LOC_Os01g38580	0.2400	3.7067	3.9490	6.63E-05	0.0037	CCD8A
LOC_Os05g45900	1.1867	3.8300	1.6904	6.67E-05	0.00371	IP5P3
LOC_Os02g46680	1.9067	5.3967	1.5010	6.70E-05	0.00372	ABCB2
LOC_Os09g09400	0.0333	0.2400	2.8480	6.75E-05	0.00374	TY3B-I
LOC_Os08g39730	3.2700	31.2900	3.2583	6.84E-05	0.00378	CYP76M5
LOC_Os03g07180	1.1633	10.1000	3.1180	6.92E-05	0.00381	-
LOC_Os07g13090	0.9500	0.0467	-4.3475	6.96E-05	0.00383	-
LOC_Os08g19590	21.6500	10.6500	-1.0235	7.13E-05	0.00391	ROC4
LOC_Os07g42040	0.6333	0.0010	-9.3068	7.33E-05	0.00402	-
LOC_Os05g07300	25.5600	11.0100	-1.2151	7.38E-05	0.00403	At2g19130
LOC_Os03g36270	0.0010	0.3000	8.2288	7.41E-05	0.00405	-
LOC_Os05g49730	0.7900	6.9600	3.1392	7.56E-05	0.0041	Os05g0572700
LOC_Os12g43140	0.1933	2.1800	3.4952	7.56E-05	0.0041	-
LOC_Os05g30350	11.2267	52.2700	2.2191	7.57E-05	0.0041	BGLU22
LOC_Os08g05780	0.2933	3.7633	3.6814	7.58E-05	0.0041	-
LOC_Os11g10710	0.5100	2.6333	2.3683	7.60E-05	0.0041	CRK15
LOC_Os08g32980	46.9933	143.5467	1.6110	7.60E-05	0.0041	-
LOC_Os03g15020	32.9033	78.8633	1.2611	7.63E-05	0.00411	Os03g0255100
LOC_Os09g22000	1.4100	4.1800	1.5678	7.68E-05	0.00413	yidA

LOC_Os07g15460	62.3567	416.0200	2.7380	7.79E-05	0.00418	NRAMP1
LOC_Os01g03360	18.4600	99.9133	2.4363	7.93E-05	0.00424	RBBI3.3
LOC_Os10g26940	7.0467	56.7500	3.0096	7.95E-05	0.00425	BURP16
LOC_Os02g36490	9.6400	28.2967	1.5535	8.00E-05	0.00427	HEX6
LOC_Os05g10670	6.9300	28.1800	2.0237	8.02E-05	0.00427	Os05g0195200
LOC_Os07g42250	13.5067	31.6033	1.2264	8.09E-05	0.0043	SSL4
LOC_Os01g55050	31.9833	11.1900	-1.5151	8.12E-05	0.0043	-
LOC_Os01g15900	4.4133	27.9500	2.6629	8.16E-05	0.00432	CDF2
LOC_Os02g18080	0.5400	0.0667	-3.0179	8.18E-05	0.00432	RGA2
LOC_Os05g13360	0.2367	3.8567	4.0264	8.30E-05	0.00438	-
LOC_Os04g39320	12.5033	1.0367	-3.5923	8.34E-05	0.00439	-
LOC_Os02g07380	0.6967	0.1333	-2.3854	8.42E-05	0.00442	TY3B-I
LOC_Os05g04770	4.7867	1.2700	-1.9142	8.50E-05	0.00446	At2g40910
LOC_Os06g44060	4.4333	1.9233	-1.2048	8.65E-05	0.00453	pldZ
LOC_Os12g18530	0.2133	1.1267	2.4009	8.67E-05	0.00453	-
LOC_Os01g22352	8.2300	20.0900	1.2875	8.72E-05	0.00455	PER1
LOC_Os01g74490	2.2300	19.8500	3.1540	8.76E-05	0.00456	ATX1
LOC_Os01g57700	2.4767	0.3967	-2.6424	8.81E-05	0.00458	-
LOC_Os01g66010	4.2067	0.1433	-4.8752	8.86E-05	0.00459	AAP2
LOC_Os10g41660	0.4433	3.8233	3.1084	8.91E-05	0.00461	ATL46
LOC_Os07g08240	0.0010	0.7067	9.4649	8.96E-05	0.00463	-
LOC_Os04g32320	0.0167	0.7000	5.3923	9.09E-05	0.00469	GDPD2
LOC_Os09g10340	3.4900	0.1833	-4.2507	9.18E-05	0.00472	CYP71D55
LOC_Os09g07230	0.0400	1.2200	4.9307	9.24E-05	0.00474	GIP
LOC_Os01g34010	0.1933	1.1833	2.6137	9.30E-05	0.00476	-
LOC_Os12g12390	8.5633	2.2667	-1.9176	9.31E-05	0.00476	-
MSTRG.5861	4.0333	26.5467	2.7185	9.42E-05	0.00481	--
LOC_Os05g47790	1.3667	20.1167	3.8797	9.43E-05	0.00481	-
LOC_Os11g09370	0.9133	0.1600	-2.5131	9.53E-05	0.00485	HDA19
LOC_Os04g32960	0.0033	0.1800	5.7549	9.62E-05	0.00489	SND1
LOC_Os03g47270	16.3600	75.2967	2.2024	9.74E-05	0.00494	-
LOC_Os02g44654	0.2033	1.4967	2.8798	9.81E-05	0.00496	CYP86A2
LOC_Os06g14400	7.1400	18.3400	1.3610	0.0001	0.00505	At1g03400
LOC_Os02g15740	0.0700	1.2000	4.0995	0.000101	0.00506	-
LOC_Os01g40950	1.2600	6.1967	2.2981	0.000101	0.00506	-
LOC_Os04g30330	0.5333	0.0133	-5.3219	0.000102	0.00509	WAKL2
LOC_Os07g02880	0.0010	0.4633	8.8559	0.000102	0.00509	-
LOC_Os11g19880	0.2767	2.1500	2.9581	0.000102	0.00509	OMT2
LOC_Os03g14560	0.0200	0.9033	5.4972	0.000103	0.00514	CYP76C1
LOC_Os09g23820	2.0267	0.1733	-3.5475	0.000103	0.00514	CYP735A1
LOC_Os12g16520	5.9300	1.9900	-1.5753	0.000105	0.00521	WAK2
LOC_Os03g06580	42.6467	101.5300	1.2514	0.000106	0.00526	notch1
LOC_Os10g40100	7.1300	2.7333	-1.3832	0.000106	0.00526	PUB33

LOC_Os06g46270	1.3600	3.9700	1.5455	0.000108	0.00534	NAC021
LOC_Os02g32860	1.8800	4.5800	1.2846	0.000108	0.00534	PARP3
LOC_Os03g11380	6.2333	1.5700	-1.9892	0.000109	0.00536	SNAP33
LOC_Os11g05700	11.3300	37.7500	1.7363	0.000111	0.00545	ABCC10
LOC_Os08g08850	0.0367	1.6100	5.4564	0.000112	0.00549	FAD4
LOC_Os04g28580	0.0010	0.2133	7.7370	0.000112	0.0055	FRS5
LOC_Os03g03980	0.5400	2.6333	2.2859	0.000113	0.00552	ccdc124-a
LOC_Os07g18120	4.9300	11.5400	1.2270	0.000113	0.00552	AO2
LOC_Os01g68770	53.0567	212.9433	2.0049	0.000118	0.00574	SBP1
LOC_Os07g46280	6.9667	17.4233	1.3225	0.000118	0.00574	BGLU26
LOC_Os10g09240	0.7300	7.9567	3.4462	0.000119	0.00576	-
LOC_Os04g23760	0.7767	0.2333	-1.7349	0.00012	0.00579	SD25
LOC_Os07g38450	0.5867	0.1067	-2.4594	0.000123	0.00592	AtMg00310
LOC_Os03g56900	18.3100	6.0167	-1.6056	0.000125	0.00603	RTNLB8
LOC_Os01g02020	22.4333	50.4900	1.1704	0.000125	0.00603	At5g47720
LOC_Os06g43640	19.2167	48.9367	1.3486	0.000126	0.00605	ACPEPP
LOC_Os12g10880	0.0010	0.9000	9.8138	0.000127	0.0061	-
LOC_Os05g49320	20.5533	5.8300	-1.8178	0.000129	0.00613	RPL12-2
LOC_Os07g48050	0.6467	0.0100	-6.0150	0.000129	0.00613	-
LOC_Os02g42150	7.0733	0.7900	-3.1625	0.000129	0.00613	WAK5
LOC_Os02g07400	3.5433	0.6833	-2.3744	0.000132	0.00626	-
LOC_Os01g66840	3.9867	0.2067	-4.2698	0.000132	0.00626	PAE5
LOC_Os09g25770	5.5367	14.1033	1.3489	0.000134	0.00632	At4g30420
LOC_Os06g50340	10.6100	4.3033	-1.3019	0.000135	0.00638	FON1
LOC_Os12g13360	0.1467	4.9100	5.0651	0.000136	0.00641	CUL1
MSTRG.19209	0.1233	2.1667	4.1348	0.000138	0.00648	--
LOC_Os09g04380	0.5800	6.7367	3.5379	0.00014	0.00655	-
LOC_Os06g17070	0.1333	1.2333	3.2095	0.00014	0.00655	-
LOC_Os04g25440	85.5833	212.9600	1.3152	0.000145	0.00676	UGT85A24
LOC_Os02g15820	0.0833	0.3967	2.2510	0.000145	0.00678	XLG1
MSTRG.7507	1.7567	8.6867	2.3060	0.000147	0.00684	--
LOC_Os06g35520	38.1167	160.6933	2.0758	0.000147	0.00685	-
LOC_Os01g04590	4.9400	10.9500	1.1483	0.000149	0.00693	-
LOC_Os01g46720	59.3033	313.6000	2.4027	0.000152	0.00705	PAP14
LOC_Os03g49126	0.9033	2.0767	1.2009	0.000152	0.00705	-
LOC_Os03g20680	1.9967	24.0700	3.5916	0.000153	0.00706	LEA1
LOC_Os05g51670	51.3267	147.9333	1.5272	0.000154	0.00711	UGE-1
LOC_Os11g10980	3.3433	8.8167	1.3989	0.000155	0.00714	-
LOC_Os03g05370	0.0167	0.9033	5.7602	0.00016	0.00732	-
LOC_Os07g34260	64.2700	1.9533	-5.0401	0.00016	0.00733	Os07g0271500
LOC_Os05g37470	9.4800	3.7567	-1.3354	0.000161	0.00735	Os05g0447200
LOC_Os01g66890	40.5200	13.2867	-1.6087	0.000162	0.00738	BT4
LOC_Os04g59440	4.2567	30.0533	2.8197	0.000162	0.00738	PSBS

LOC_Os06g30830	0.4033	2.6267	2.7032	0.000162	0.00738	AGL61
LOC_Os01g11750	0.2367	0.9967	2.0743	0.000163	0.00741	At5g45910
LOC_Os09g08570	0.0533	0.9567	4.1649	0.000164	0.00744	-
LOC_Os07g32060	2.9567	0.9500	-1.6380	0.000165	0.00749	RhGT1
LOC_Os06g40150	0.0700	2.1267	4.9251	0.000166	0.0075	WIN1
LOC_Os01g52480	3.1733	0.7067	-2.1669	0.00017	0.00769	-
LOC_Os07g26630	0.1433	2.4400	4.0894	0.000172	0.00777	PIP2-4
LOC_Os08g05980	1.9667	41.4433	4.3973	0.000173	0.00777	-
LOC_Os09g31482	46.9200	159.0133	1.7609	0.000175	0.00785	U2AF35A
LOC_Os04g39380	14.4900	2.6067	-2.4748	0.000176	0.00788	-
LOC_Os11g41380	0.2967	0.0100	-4.8908	0.000178	0.00795	FRS5
LOC_Os06g06570	2.5867	0.6933	-1.8995	0.000178	0.00796	-
LOC_Os06g23684	7.7467	20.8000	1.4249	0.000179	0.00799	naat-A
LOC_Os03g62330	1.0467	3.5000	1.7416	0.000182	0.00812	-
LOC_Os11g36030	3.1833	1.1100	-1.5200	0.000185	0.0082	CDCA7L
LOC_Os01g25360	1.4333	0.2833	-2.3388	0.000185	0.0082	MES7
LOC_Os12g37260	152.2767	43.4100	-1.8106	0.000185	0.0082	LOX2.1
LOC_Os04g15650	4.8067	0.6733	-2.8356	0.000185	0.0082	At3g47570
LOC_Os04g52640	28.9000	10.3800	-1.4773	0.000187	0.00825	At1g56130
LOC_Os02g40900	6.5800	18.2767	1.4738	0.000188	0.00828	RBM42
LOC_Os03g45779	19.5900	6.3133	-1.6336	0.000189	0.00829	-
LOC_Os04g34270	33.4933	3.2033	-3.3862	0.000189	0.00829	At2g19130
LOC_Os06g32990	2.4633	0.2833	-3.1200	0.000193	0.00845	PER36
LOC_Os06g05020	0.2533	10.1767	5.3281	0.000197	0.00861	-
LOC_Os03g45450	10.1867	1.8667	-2.4481	0.000198	0.00867	WRKY71
LOC_Os08g36570	1.5733	0.3367	-2.2244	0.000199	0.00867	At1g10780
LOC_Os04g54300	7.9900	54.7300	2.7761	0.000201	0.00875	-
MSTRG.26590	0.4300	3.3733	2.9718	0.000202	0.00877	--
LOC_Os03g60850	13.4333	5.2133	-1.3655	0.000204	0.00884	NPF5.7
LOC_Os07g14700	10.9600	4.4100	-1.3134	0.000204	0.00884	-
LOC_Os08g42040	36.8900	84.3967	1.1940	0.000206	0.00889	LTPG1
LOC_Os09g02270	2.0000	6.6667	1.7370	0.000206	0.00889	kynB
LOC_Os09g15170	4.1333	1.5367	-1.4275	0.000206	0.00889	NAT2
LOC_Os01g62610	0.5867	4.3467	2.8893	0.000211	0.00908	FKBP20-1
LOC_Os03g20910	0.0010	0.2267	7.8244	0.000212	0.00911	WOX6
LOC_Os11g37630	0.0067	0.2933	5.4594	0.000212	0.00911	TBL19
LOC_Os04g59540	0.2967	2.6367	3.1518	0.000213	0.00912	FAB1B
LOC_Os02g32690	0.0467	0.7667	4.0381	0.000214	0.00914	ABCG41
LOC_Os03g09250	8.8167	44.7833	2.3447	0.000214	0.00916	INO1
LOC_Os05g07710	0.5733	1.6800	1.5510	0.000216	0.00923	ATG18D
LOC_Os07g29940	0.7233	0.0867	-3.0611	0.000217	0.00923	-
LOC_Os07g43604	0.2633	1.8333	2.7995	0.000218	0.00924	-
LOC_Os06g13560	3.9100	8.9833	1.2001	0.000218	0.00924	AAMT2

LOC_Os01g56180	7.3467	30.7833	2.0670	0.000219	0.00927	-
LOC_Os09g09410	0.2700	1.9867	2.8793	0.00022	0.00928	-
LOC_Os01g03720	0.2867	2.2567	2.9767	0.00022	0.00928	MYB108
LOC_Os11g07911	1.7667	24.1633	3.7737	0.000231	0.0097	-
LOC_Os01g37200	0.1000	3.0267	4.9197	0.000232	0.00973	-
LOC_Os01g48640	5.3167	1.2267	-2.1158	0.000233	0.00977	-
LOC_Os09g11640	0.0010	0.0933	6.5443	0.000234	0.00977	-
LOC_Os08g26840	48.8733	6.7700	-2.8518	0.000234	0.00977	-
LOC_Os07g07930	9.8633	27.2800	1.4677	0.000245	0.01019	At2g13820
LOC_Os01g06790	2.5900	0.8267	-1.6476	0.000248	0.01027	RLP12
LOC_Os03g43750	0.3467	2.4000	2.7914	0.000248	0.01027	-
LOC_Os11g46950	1.0933	0.2767	-1.9825	0.000248	0.01027	WAK2
LOC_Os03g08624	5.9433	42.3600	2.8334	0.000248	0.01027	Rv1106c
LOC_Os11g04030	0.0200	0.5867	4.8745	0.000249	0.01028	ZIFL1
LOC_Os11g43760	0.0067	0.2867	5.4263	0.00025	0.01032	DAGLA
LOC_Os06g29220	0.0833	2.2867	4.7782	0.000251	0.01032	Os06g0486900
MSTRG.13841	1.2833	0.1667	-2.9449	0.000252	0.01035	--
LOC_Os09g32560	17.6300	7.9767	-1.1442	0.000252	0.01035	Os08g0520100
LOC_Os05g30660	0.1933	1.0633	2.4594	0.000257	0.01051	EXO70B1
LOC_Os11g32210	0.0100	0.2300	4.5236	0.000261	0.01064	-
LOC_Os05g38219	4.2000	1.8900	-1.1520	0.000261	0.01066	-
LOC_Os01g47290	0.1267	3.6967	4.8671	0.000262	0.01067	-
LOC_Os11g32890	0.3300	7.2000	4.4475	0.000263	0.01069	-
LOC_Os02g07260	41.0033	96.7400	1.2384	0.000265	0.01074	-
LOC_Os11g39020	42.0567	175.2200	2.0588	0.000265	0.01074	ABCF5
LOC_Os10g32810	1.1800	12.5800	3.4143	0.000267	0.01079	BAM1
LOC_Os07g37310	14.7233	32.2967	1.1333	0.00027	0.01088	-
LOC_Os02g57840	0.4800	1.9000	1.9849	0.000271	0.01091	-
MSTRG.27288	4.2767	0.9400	-2.1858	0.000272	0.01091	--
LOC_Os01g13480	14.3133	49.1233	1.7790	0.000272	0.01091	Grxcr1
MSTRG.3380	3.4300	1.3733	-1.3205	0.000272	0.01093	--
LOC_Os12g02210	4.0800	12.4567	1.6103	0.000275	0.01102	ATL44
LOC_Os07g05740	0.3767	1.0167	1.4325	0.000277	0.01107	At1g17230
LOC_Os04g01130	1.4600	0.2967	-2.2991	0.00028	0.0112	MRG1
LOC_Os09g39930	1.7033	4.1733	1.2928	0.000281	0.01121	PBS1
LOC_Os06g38450	34.8267	7.9200	-2.1366	0.000282	0.01123	SAG39
LOC_Os01g49280	0.3200	1.7733	2.4703	0.000284	0.01129	-
LOC_Os11g07230	11.0633	4.7733	-1.2127	0.000291	0.01153	At3g47570
LOC_Os02g57160	7.2367	40.4967	2.4844	0.000291	0.01153	At1g79600
LOC_Os02g08490	1.1367	4.5700	2.0074	0.000291	0.01153	CLPB3
LOC_Os01g15100	1.6300	0.0333	-5.6118	0.000296	0.0117	-
LOC_Os02g51900	5.3733	1.8600	-1.5305	0.000298	0.01174	UGT85A24
MSTRG.3054	0.8500	2.5233	1.5698	0.000301	0.01184	TULP9

LOC_Os07g46520	4.6667	28.6067	2.6159	0.000302	0.01189	HDHD3
LOC_Os11g44560	2.5067	0.4867	-2.3648	0.000304	0.01194	CCR4
LOC_Os01g05780	0.2033	2.6000	3.6766	0.000304	0.01194	PAS2A
LOC_Os10g22520	15.3000	42.8000	1.4841	0.000308	0.01206	exgA
LOC_Os02g14160	2.4433	10.0900	2.0460	0.00031	0.01212	PER52
LOC_Os05g51470	9.8933	32.9933	1.7376	0.00031	0.01212	PCO2
LOC_Os01g02830	5.6300	2.6533	-1.0853	0.000311	0.01214	At1g67000
LOC_Os10g11910	0.0010	0.2367	7.8867	0.000313	0.01218	-
LOC_Os08g04210	0.0010	0.2133	7.7370	0.000318	0.01236	CRRSP55
LOC_Os01g39020	1.1467	5.9467	2.3746	0.00032	0.01241	HSFA6B
LOC_Os03g42520	0.1467	1.7800	3.6013	0.000321	0.01242	-
MSTRG.18204	0.3533	1.2567	1.8305	0.000321	0.01243	--
LOC_Os01g10400	2944.9033	646.9400	-2.1865	0.000324	0.01249	-
LOC_Os06g08710	1.0900	0.2100	-2.3759	0.000324	0.01249	EFR
LOC_Os03g52380	2.0200	11.0500	2.4516	0.000328	0.01261	-
LOC_Os04g39560	5.7467	20.3733	1.8259	0.000328	0.01262	-
LOC_Os08g10250	48.4300	12.1667	-1.9930	0.000337	0.01291	At1g56140
LOC_Os07g46420	1.5200	5.7500	1.9195	0.000338	0.01296	-
LOC_Os02g36880	4.5667	16.0200	1.8107	0.000339	0.01298	NAC100
LOC_Os12g02420	4.0933	8.8100	1.1059	0.000341	0.01302	WRKY55
LOC_Os01g70500	0.9467	2.4933	1.3971	0.000341	0.01302	-
LOC_Os06g22925	2.8567	10.7800	1.9160	0.000344	0.01309	-
LOC_Os10g23240	1.7867	29.2400	4.0326	0.000345	0.0131	-
LOC_Os11g33970	4.8067	1.6667	-1.5281	0.000345	0.0131	At3g47200
LOC_Os01g68370	0.0010	0.1100	6.7814	0.000347	0.01315	VP1
LOC_Os10g36180	1.6500	24.1300	3.8703	0.000349	0.01323	LTI65
LOC_Os02g34250	0.1733	2.5633	3.8864	0.000353	0.01336	-
LOC_Os06g36680	0.2267	0.0133	-4.0875	0.000356	0.01346	ATH1
LOC_Os01g73604	0.8567	0.1033	-3.0514	0.000359	0.01356	snrpd2
LOC_Os03g04920	12.9100	29.1500	1.1750	0.000362	0.01365	ABCC13
LOC_Os07g43390	5.5267	13.0033	1.2344	0.000363	0.01366	DPE1
LOC_Os04g32620	2.6800	9.8900	1.8837	0.000364	0.01366	ERF113
LOC_Os08g39330	0.4167	4.3367	3.3796	0.000365	0.01369	-
LOC_Os10g40040	0.2600	2.5333	3.2845	0.000372	0.0139	-
LOC_Os01g70850	3.5000	27.0200	2.9486	0.000375	0.01398	PIR7B
LOC_Os05g47540	161.3100	24.0633	-2.7449	0.000382	0.0142	NMT1
LOC_Os01g06660	3.2967	12.2600	1.8949	0.000382	0.0142	PDC1
LOC_Os03g53740	17.1133	6.9300	-1.3042	0.000383	0.0142	-
LOC_Os04g55240	0.5433	3.0300	2.4794	0.000383	0.0142	VQ11
LOC_Os11g01869	2.7433	5.7133	1.0584	0.000384	0.0142	DRB7
LOC_Os08g04810	2.2267	4.4633	1.0032	0.00039	0.01443	-
LOC_Os06g36560	2.8167	38.4767	3.7719	0.000392	0.01449	Os06g0561000
LOC_Os04g35210	2.7267	0.7500	-1.8622	0.000395	0.01458	-

LOC_Os06g46436	68.6133	31.9500	-1.1027	0.000398	0.01464	psaC
LOC_Os09g33680	20.4300	68.8333	1.7524	0.000398	0.01464	BGLU31
LOC_Os07g04930	21.2833	126.2267	2.5682	0.000399	0.01466	Prx
LOC_Os07g22498	66.9133	30.9633	-1.1117	0.000406	0.01488	psaC
LOC_Os07g34520	3.8867	49.5867	3.6733	0.000408	0.01493	ICL
LOC_Os07g47100	11.7567	26.2400	1.1583	0.00041	0.015	NHX2
LOC_Os03g19700	2.4400	0.5767	-2.0811	0.000414	0.01512	At3g47200
LOC_Os03g55034	0.0800	1.1100	3.7944	0.000415	0.01513	-
LOC_Os03g12660	13.0133	2.9200	-2.1560	0.000417	0.01519	CYP90B1
LOC_Os04g28020	3.7567	1.5100	-1.3149	0.000418	0.01521	PRD1
LOC_Os10g37290	7.6767	2.6267	-1.5472	0.000424	0.01542	-
LOC_Os10g21406	40.2600	18.3533	-1.1333	0.000433	0.01572	psaC
LOC_Os04g58380	4.6467	2.0300	-1.1947	0.000435	0.01575	-
LOC_Os05g02040	0.5033	1.0833	1.1059	0.000435	0.01575	RPA1C
LOC_Os06g48720	0.4033	1.1167	1.4692	0.000438	0.01582	HMA3
LOC_Os10g38020	0.0010	0.1667	7.3808	0.000439	0.01586	-
LOC_Os02g44680	2.3067	0.5267	-2.1308	0.000442	0.01595	NCS1
LOC_Os02g53410	3.0833	9.7900	1.6668	0.000452	0.01625	-
LOC_Os02g07930	3.1967	13.7233	2.1020	0.000456	0.01635	BBX32
LOC_Os01g28970	0.3367	9.2133	4.7743	0.000458	0.01642	-
LOC_Os01g36710	0.4333	0.0700	-2.6301	0.000459	0.01643	-
LOC_Os04g32030	5.9333	19.4933	1.7161	0.00046	0.01643	HIPP26
LOC_Os08g20200	0.3733	1.6833	2.1728	0.000474	0.01691	FAR1
LOC_Os09g03200	0.3633	3.1933	3.1357	0.000475	0.01691	-
LOC_Os02g55070	0.1500	0.0010	-7.2288	0.000476	0.01691	-
LOC_Os09g08130	23.4600	2.4833	-3.2399	0.000482	0.0171	At2g04400
LOC_Os05g50930	3.3867	33.6767	3.3138	0.000483	0.01714	SIGE
LOC_Os01g06640	2.8867	8.7300	1.5966	0.000486	0.01722	BHLH51
LOC_Os03g57400	0.9800	0.1767	-2.4718	0.000487	0.01723	EPB2
LOC_Os04g27020	0.2033	0.0010	-7.6677	0.000492	0.01739	CYP71Z7
LOC_Os03g20440	3.0767	0.3933	-2.9675	0.000495	0.01746	CAMBP25
LOC_Os11g15570	23.2400	4.3567	-2.4153	0.000496	0.01748	At1g18480
LOC_Os01g66240	4.1233	11.1933	1.4408	0.000504	0.01772	SPAC22H12.03
LOC_Os02g54560	0.0010	0.2900	8.1799	0.000512	0.01798	-
LOC_Os12g17570	0.1967	1.2500	2.6681	0.000514	0.01803	At1g33811
LOC_Os12g29820	0.7733	0.1667	-2.2141	0.000516	0.01808	-
LOC_Os01g10440	6.8300	1.9000	-1.8459	0.00052	0.01819	oxt
LOC_Os11g05390	0.2133	4.0467	4.2456	0.000522	0.01825	tetA
LOC_Os05g27790	9.0967	23.6267	1.3770	0.000525	0.01833	Os05g0344400
LOC_Os11g37880	0.1267	0.8200	2.6946	0.000526	0.01835	RPP13
MSTRG.17405	0.6633	4.7700	2.8462	0.000528	0.01839	--
LOC_Os03g25790	2.7833	12.6533	2.1846	0.000529	0.01839	At3g13560
LOC_Os06g19260	3.1000	0.8600	-1.8499	0.000531	0.01846	-

LOC_Os02g21090	2.2033	0.7600	-1.5356	0.000538	0.01863	BHLH144
MSTRG.5222	14.5600	30.9433	1.0876	0.000538	0.01863	ecdK
LOC_Os07g47550	0.0100	0.3267	5.0297	0.000539	0.01864	UGT94E5
LOC_Os03g12500	6.2100	0.4267	-3.8634	0.000544	0.01876	CYP74A2
LOC_Os10g30832	2.1900	0.2967	-2.8840	0.000545	0.01876	-
LOC_Os01g05760	0.3467	3.6667	3.4028	0.000546	0.01876	PAS2A
LOC_Os01g71830	0.4833	3.4700	2.8438	0.000546	0.01876	-
LOC_Os12g32620	0.3100	1.9167	2.6283	0.000546	0.01876	WLIM1
LOC_Os04g37570	0.6567	4.0933	2.6400	0.000552	0.01894	nep2
LOC_Os01g27480	5.6033	0.9300	-2.5910	0.000553	0.01897	GSTF1
LOC_Os10g37120	1.1800	0.0667	-4.1457	0.000556	0.01904	CYP89A2
LOC_Os04g26320	2.9000	0.7867	-1.8822	0.000559	0.01911	JAL3
LOC_Os03g48310	2.6067	7.0233	1.4299	0.000563	0.01921	LHA1
LOC_Os04g38790	16.7167	3.4800	-2.2641	0.000566	0.01928	-
LOC_Os01g04370	0.5633	2.6000	2.2065	0.000571	0.0194	HSP16.9A
LOC_Os12g10870	0.1200	0.9767	3.0248	0.000571	0.0194	RLP12
LOC_Os01g16060	1.4100	0.4667	-1.5952	0.000574	0.01947	-
LOC_Os01g11160	23.9033	3.4300	-2.8009	0.00058	0.01963	CAT5
LOC_Os05g18670	1.4333	0.3067	-2.2246	0.000581	0.01965	SLAH1
LOC_Os07g36570	13.1667	1.5433	-3.0928	0.000582	0.01965	B120
LOC_Os12g37519	5.1867	36.0433	2.7969	0.00059	0.01988	-
MSTRG.8541	1.0867	3.9433	1.8595	0.000592	0.01995	--
LOC_Os08g07680	4.3267	1.3700	-1.6591	0.000595	0.02001	-
LOC_Os02g58170	1.4300	3.9500	1.4658	0.000596	0.02001	Os03g0733400
LOC_Os05g18446	0.9467	3.4133	1.8503	0.000596	0.02001	-
LOC_Os10g40570	19.4167	45.3933	1.2252	0.000601	0.02014	At1g63370
LOC_Os03g17810	0.1533	1.0900	2.8296	0.000604	0.02022	LBD1
LOC_Os08g42470	0.0233	0.5300	4.5055	0.000611	0.02045	BHLH137
LOC_Os03g45250	0.1633	1.6100	3.3012	0.000615	0.02055	PCO2
LOC_Os03g05440	0.2400	1.5800	2.7188	0.000629	0.02098	Os03g0148000
LOC_Os04g49350	7.5533	48.8500	2.6932	0.00063	0.021	At5g13770
MSTRG.26335	1.2700	9.5100	2.9046	0.000635	0.02114	--
LOC_Os03g61270	0.8467	6.9633	3.0399	0.000646	0.02149	MAN3
LOC_Os07g46540	0.1600	0.5667	1.8244	0.000651	0.02159	NCAPD2
LOC_Os04g53210	3.1400	18.0833	2.5258	0.000655	0.0217	GLO3
LOC_Os03g19000	0.1333	0.7267	2.4463	0.000658	0.02176	PEX11-2
LOC_Os04g32390	0.5267	0.0010	-9.0407	0.00066	0.0218	-
LOC_Os02g51119	3.9900	1.1100	-1.8458	0.000663	0.02187	-
LOC_Os02g32660	0.6300	1.5267	1.2770	0.000665	0.02191	SBE1
LOC_Os02g43330	2.2833	27.9333	3.6128	0.000666	0.02191	HOX24
LOC_Os03g63810	0.5700	1.8400	1.6907	0.000668	0.02196	WRKY35
LOC_Os07g43240	0.1700	1.8033	3.4071	0.000669	0.02199	ASK9
LOC_Os03g01160	3.8767	1.1200	-1.7913	0.000679	0.02224	PUB52

LOC_Os07g44410	36.8633	190.0167	2.3659	0.000686	0.02242	tolB
LOC_Os06g44260	0.5567	2.4733	2.1516	0.000692	0.0226	Os06g0652400
LOC_Os04g04390	0.2267	0.0567	-2.0000	0.000694	0.02264	RPA1C
LOC_Os01g32120	1.2067	3.6800	1.6087	0.000698	0.02275	CML11
LOC_Os07g24230	0.2000	1.9400	3.2780	0.000699	0.02275	At2g33280
LOC_Os01g16370	0.0767	0.3733	2.2838	0.000706	0.02292	RPM1
LOC_Os10g39044	1.5633	0.0433	-5.1730	0.000707	0.02292	-
LOC_Os07g36465	16.6567	34.2100	1.0383	0.000709	0.02295	Os07g0549700
LOC_Os11g12310	1.1933	0.1633	-2.8691	0.00071	0.02295	TY3B-I
LOC_Os05g31140	169.4933	674.7000	1.9930	0.00071	0.02295	-
LOC_Os06g51260	3.0500	30.1433	3.3050	0.00072	0.0232	RVE2
LOC_Os12g03594	2.2567	0.7967	-1.5021	0.00072	0.0232	PP2B10
LOC_Os07g09190	10.0467	1.5067	-2.7373	0.000721	0.0232	Os07g0190000
LOC_Os07g02810	0.5033	2.1900	2.1213	0.000723	0.02322	-
LOC_Os03g44150	9.4033	28.1900	1.5839	0.000728	0.02335	OAT
LOC_Os11g31090	2.2500	8.2633	1.8768	0.000729	0.02335	HHT1
LOC_Os09g29600	3.4467	1.3400	-1.3630	0.00073	0.02335	WAK2
LOC_Os05g25650	6.7933	16.3033	1.2630	0.000732	0.02335	-
LOC_Os06g36310	2.4767	0.9133	-1.4392	0.000732	0.02335	RLK5
LOC_Os05g37830	1.7500	8.0033	2.1932	0.000733	0.02335	-
LOC_Os12g29950	14.5400	4.9667	-1.5497	0.000733	0.02335	YMR155W
LOC_Os05g25950	6.9200	2.8700	-1.2697	0.00074	0.02353	CYCU4-2
LOC_Os12g07610	1.4733	0.1367	-3.4304	0.000743	0.0236	MYB98
LOC_Os04g12499	4.6067	18.4433	2.0013	0.000748	0.02373	ANT1
LOC_Os12g33150	301.9933	70.2400	-2.1042	0.000762	0.02413	-
LOC_Os09g14490	4.2133	1.3600	-1.6314	0.000764	0.02418	RGA2
MSTRG.4488	10.6700	4.7733	-1.1605	0.000767	0.02424	--
LOC_Os01g57964	6.0533	2.8500	-1.0868	0.000771	0.02433	psaA
LOC_Os08g39210	1.2133	0.4733	-1.3580	0.000772	0.02434	WAK3
LOC_Os05g36290	21.6400	53.4600	1.3048	0.000773	0.02434	ACT1
LOC_Os07g33440	0.1267	0.8267	2.7063	0.000779	0.02452	-
LOC_Os03g02874	1.4700	15.5000	3.3984	0.000785	0.02467	-
LOC_Os01g53090	1.4300	4.8700	1.7679	0.00079	0.02477	-
LOC_Os03g40040	1.4567	0.3833	-1.9260	0.000791	0.02479	-
LOC_Os07g22494	18.0600	7.6267	-1.2437	0.000792	0.02479	-
LOC_Os05g30980	7.7067	16.2133	1.0730	0.000802	0.02503	BRN1
LOC_Os07g48830	45.4100	122.4833	1.4315	0.000813	0.02536	GOLS2
LOC_Os01g45460	0.5400	2.6633	2.3022	0.000814	0.02537	IMCEL2
LOC_Os10g38610	1.4667	6.6767	2.1866	0.000819	0.02547	GSTU6
LOC_Os06g26234	15.0133	5.1033	-1.5567	0.00082	0.02549	SBE3
LOC_Os10g20794	6.3567	19.7167	1.6331	0.000825	0.0256	-
LOC_Os01g26280	5.6467	1.4000	-2.0120	0.000827	0.02565	WAK5
LOC_Os02g28340	3.1700	8.4800	1.4196	0.000831	0.02565	3AT1

LOC_Os01g57004	2.5667	7.1133	1.4706	0.000832	0.02565	-
LOC_Os05g37140	17.7967	53.5467	1.5892	0.000833	0.02565	FDX6
LOC_Os04g53120	3.1067	0.7433	-2.0633	0.000833	0.02565	RGA2
LOC_Os10g11930	0.0010	0.1667	7.3808	0.000833	0.02565	-
LOC_Os11g28540	0.3133	0.0010	-8.2916	0.000833	0.02565	-
LOC_Os06g12310	51.5800	13.9333	-1.8883	0.000834	0.02565	NIP2-2
LOC_Os09g10010	1.7800	12.3867	2.7988	0.000843	0.02585	-
LOC_Os07g26110	2.2500	13.6300	2.5988	0.000843	0.02585	Os07g0442900
LOC_Os02g24100	75.4033	22.9267	-1.7176	0.000848	0.02597	-
LOC_Os12g18700	8.1200	2.3700	-1.7766	0.000855	0.02615	-
LOC_Os11g42510	4.8167	25.9167	2.4278	0.000856	0.02615	naat-A
LOC_Os08g40910	0.1767	0.9467	2.4218	0.000857	0.02615	-
LOC_Os01g02600	2.4900	7.1367	1.5191	0.000861	0.02626	At5g39020
LOC_Os05g05060	110.7700	27.1067	-2.0308	0.000867	0.02639	-
LOC_Os02g01280	3.9700	10.3833	1.3871	0.000874	0.02656	-
LOC_Os03g55030	4.1000	18.8767	2.2029	0.000877	0.02662	UGT83A1
LOC_Os08g42990	0.1900	1.4233	2.9052	0.000882	0.02675	-
LOC_Os07g48490	204.3400	73.1233	-1.4826	0.00089	0.02698	-
LOC_Os12g36810	0.6367	2.6233	2.0428	0.000892	0.02701	GSVIVT00026920001
LOC_Os08g18974	0.0300	0.6833	4.5096	0.000895	0.02701	-
LOC_Os04g16760	7.6633	3.7733	-1.0221	0.000895	0.02701	psaA
LOC_Os03g49830	6.2100	1.6967	-1.8719	0.000898	0.02704	At5g22090
LOC_Os11g32750	7.7033	2.3000	-1.7438	0.000898	0.02704	NUDT13
LOC_Os11g05770	2.2367	5.4600	1.2876	0.000904	0.02719	OFP13
LOC_Os10g21250	4.3267	2.0500	-1.0776	0.000907	0.02725	psaA
LOC_Os02g49660	1.2633	0.2933	-2.1066	0.000923	0.0277	TBL19
LOC_Os05g15530	4.6333	19.0567	2.0402	0.000925	0.02772	DAAT
LOC_Os12g43130	12.8167	47.0033	1.8747	0.000934	0.02795	PSY
LOC_Os04g53930	1.8433	6.3367	1.7814	0.000935	0.02797	4-Oct
LOC_Os05g48390	32.2833	12.8533	-1.3286	0.00094	0.02807	UBC24
LOC_Os06g23780	0.0010	0.3233	8.3369	0.000946	0.02823	ACX4
LOC_Os05g04600	1.8533	10.1933	2.4594	0.000951	0.02834	ABCB11
LOC_Os04g47150	0.0933	0.0010	-6.5443	0.000958	0.02849	SBT1.2
LOC_Os10g38740	9.5267	39.7267	2.0601	0.000961	0.02856	GSTU6
LOC_Os01g47490	9.3967	19.6133	1.0616	0.000974	0.02893	-
LOC_Os01g44960	1.9333	7.4500	1.9462	0.00098	0.02906	ephx3
LOC_Os08g27170	2.3233	0.2133	-3.4450	0.000983	0.02912	SARD1
LOC_Os12g02470	2.6867	0.1033	-4.7004	0.000989	0.02922	WRKY53
LOC_Os08g12880	1.4800	0.2733	-2.4369	0.000989	0.02922	-
LOC_Os02g43170	50.9633	15.8433	-1.6856	0.00099	0.02922	BBX21
LOC_Os04g48460	4.4667	10.7100	1.2617	0.000994	0.02925	CYP704C1
LOC_Os07g45210	0.1433	0.0010	-7.1632	0.000997	0.02925	-
LOC_Os01g73110	28.1000	78.1333	1.4754	0.000998	0.02925	-

LOC_Os05g07980	0.3333	0.0600	-2.4739	0.000998	0.02925	-
LOC_Os06g23350	0.6867	6.2400	3.1839	0.000998	0.02925	-
LOC_Os06g01972	7.8233	1.3667	-2.5171	0.000998	0.02925	At3g30340
LOC_Os12g06010	0.0010	0.0367	5.1964	0.001001	0.0293	-
LOC_Os01g03310	970.3533	300.7800	-1.6898	0.001011	0.02957	RBBI3.3
LOC_Os03g51530	18.1533	69.2033	1.9306	0.001015	0.02965	-
LOC_Os03g04080	0.9367	11.4633	3.6133	0.00102	0.02977	-
LOC_Os02g27310	5.3000	2.1233	-1.3197	0.001022	0.02979	CRK41
LOC_Os03g32490	103.4500	485.5933	2.2308	0.001031	0.03	ycf36
LOC_Os11g12300	1.5833	0.2367	-2.7420	0.001031	0.03	RPM1
LOC_Os11g29050	2.6733	1.0367	-1.3667	0.001036	0.0301	RPM1
LOC_Os12g32470	6.8767	2.5867	-1.4106	0.001038	0.03013	-
LOC_Os03g61310	1.7933	0.5033	-1.8331	0.001039	0.03013	RLK1
LOC_Os01g65380	21.0833	51.6633	1.2930	0.001049	0.03034	PATL5
LOC_Os01g41770	0.1300	0.9067	2.8021	0.00105	0.03034	GSO2
LOC_Os05g06399	4.4433	16.2533	1.8710	0.00105	0.03034	-
LOC_Os03g16390	29.8900	8.2200	-1.8625	0.001051	0.03036	-
LOC_Os11g11960	23.0100	3.7667	-2.6109	0.001058	0.0305	RPM1
LOC_Os01g47420	14.6467	5.5167	-1.4087	0.001059	0.0305	phhB
LOC_Os06g24430	0.4267	2.9833	2.8057	0.001062	0.03056	-
LOC_Os07g24000	0.0010	0.1933	7.5949	0.001072	0.03078	-
LOC_Os06g10109	5.1200	1.1467	-2.1587	0.001074	0.03078	-
LOC_Os03g63580	0.3100	1.6967	2.4524	0.001082	0.03095	NPC4
LOC_Os05g01675	8.1067	3.9900	-1.0227	0.001089	0.03113	psaA
LOC_Os10g39220	14.1067	29.2667	1.0529	0.001094	0.03123	TOM3
LOC_Os04g48070	1.4433	4.0067	1.4730	0.0011	0.03139	ROC4
LOC_Os11g32030	0.9867	2.6567	1.4290	0.00111	0.0316	SDR2a
LOC_Os01g52100	0.7600	1.9567	1.3643	0.00111	0.0316	-
LOC_Os04g54070	2.6333	0.7467	-1.8184	0.001116	0.03173	SD18
LOC_Os01g43460	5.1933	51.1200	3.2992	0.001128	0.03198	SLAH3
LOC_Os01g62540	4.9033	1.7700	-1.4700	0.00113	0.03198	-
LOC_Os08g39840	30.4200	2.9333	-3.3744	0.001131	0.03198	CM-LOX1
LOC_Os07g27660	0.9267	0.1633	-2.5042	0.001132	0.03198	-
LOC_Os04g30250	3.6633	0.7467	-2.2946	0.001138	0.03214	WAK2
LOC_Os03g43720	7.9967	35.6067	2.1547	0.001146	0.03233	7-Oct
LOC_Os10g18370	17.3567	167.2200	3.2682	0.001149	0.03238	Cpar_0662
LOC_Os08g03290	240.1567	517.9467	1.1088	0.001152	0.03242	GAPC
LOC_Os12g23980	0.1333	0.8867	2.7334	0.001158	0.03256	SBT1.2
LOC_Os04g13140	0.1067	1.3567	3.6689	0.001161	0.03262	OsI_14861
LOC_Os02g48770	4.2333	0.3567	-3.5691	0.001168	0.03279	AAMT1
LOC_Os11g37200	2.8467	0.4533	-2.6506	0.00117	0.03279	At4g15470
LOC_Os06g45040	0.0200	0.6133	4.9386	0.001174	0.03287	BBX32
LOC_Os07g43820	0.5700	2.8200	2.3067	0.001177	0.03292	XIPI

LOC_Os02g12130	6.5200	1.3100	-2.3153	0.001181	0.03293	At1g61370
LOC_Os11g43320	0.7300	0.3067	-1.2512	0.001184	0.03293	RPM1
LOC_Os04g52440	0.0633	0.6233	3.2990	0.001185	0.03293	Os04g0614500
LOC_Os06g41880	0.2000	0.5800	1.5361	0.001185	0.03293	AIR3
LOC_Os06g41940	0.3800	2.0433	2.4269	0.001186	0.03293	-
LOC_Os11g41210	0.2233	0.0367	-2.6067	0.001186	0.03293	-
LOC_Os12g04595	0.9333	0.2633	-1.8255	0.001188	0.03293	gtf2e1-1
LOC_Os08g24750	0.5933	3.9333	2.7288	0.001189	0.03293	FT1
LOC_Os09g19300	10.9067	2.3767	-2.1982	0.00119	0.03293	-
LOC_Os03g40100	5.4267	21.4000	1.9795	0.001191	0.03293	ACR4
LOC_Os04g32240	1.0367	0.2667	-1.9588	0.001191	0.03293	-
LOC_Os08g29854	0.6033	1.7000	1.4945	0.001192	0.03293	RPM1
LOC_Os09g07200	0.1900	2.9433	3.9534	0.001193	0.03293	FRS5
LOC_Os01g24820	0.0267	0.5467	4.3576	0.001199	0.03304	RGA2
LOC_Os02g53620	2.3700	0.7600	-1.6408	0.0012	0.03304	NFYA5
LOC_Os12g38170	771.1267	154.8667	-2.3159	0.001203	0.03308	-
LOC_Os10g02814	0.2133	0.6833	1.6795	0.001206	0.03313	XLG3
LOC_Os04g27430	1.2000	11.3000	3.2352	0.001215	0.03333	-
LOC_Os02g10310	49.9267	115.6733	1.2122	0.001215	0.03333	FAH
LOC_Os05g03920	18.9233	58.7633	1.6347	0.001231	0.03371	CRK2
LOC_Os06g21380	6.5233	38.5767	2.5640	0.001233	0.03375	CTPA3
LOC_Os12g05120	1.1967	0.3533	-1.7599	0.001235	0.03375	PXC1
LOC_Os04g12970	1.6100	15.7233	3.2878	0.001247	0.03407	UGT74E2
LOC_Os07g47010	0.0010	0.1233	6.9464	0.001254	0.03421	At1g48120
LOC_Os09g14670	3.0900	7.3633	1.2528	0.001264	0.03445	-
LOC_Os07g34850	1.1367	0.1867	-2.6063	0.001268	0.03449	nep1
LOC_Os06g08560	8.7033	3.7300	-1.2224	0.001269	0.03449	ABCC13
LOC_Os04g28820	0.0010	0.0333	5.0589	0.001269	0.03449	pif1
LOC_Os07g48790	0.2333	0.0010	-7.8662	0.001276	0.03458	KINB1
MSTRG.17988	0.2100	1.8633	3.1494	0.001276	0.03458	--
LOC_Os09g07190	0.1300	1.9233	3.8870	0.001283	0.03474	Tf2-11
LOC_Os12g23150	12.6800	5.3367	-1.2485	0.001294	0.03497	PGLP1B
LOC_Os09g38920	4.4133	17.6733	2.0016	0.001295	0.03497	SAG12
LOC_Os11g02310	1.0400	0.3000	-1.7935	0.0013	0.03505	PCMP-E101
LOC_Os11g04150	4.0600	15.1567	1.9004	0.001301	0.03505	ZIFL1
LOC_Os11g29210	3.4600	0.7833	-2.1431	0.001307	0.03519	-
LOC_Os08g01480	0.0100	0.3633	5.1832	0.001312	0.03525	CYP71C4
LOC_Os08g10290	8.6467	1.1900	-2.8612	0.001312	0.03525	At1g56130
LOC_Os11g37960	5.5867	22.9700	2.0397	0.001314	0.03526	PR4A
LOC_Os04g43360	2.9100	1.3067	-1.1551	0.001315	0.03527	BGLU14
LOC_Os06g14770	1.1600	0.0800	-3.8580	0.001323	0.0354	-
LOC_Os12g25090	1.9533	10.3933	2.4116	0.001324	0.0354	-
LOC_Os11g40470	0.5100	0.1367	-1.8998	0.001337	0.03572	TY3B-G

LOC_Os12g41650	16.7333	41.8733	1.3233	0.001363	0.03638	PIF1
LOC_Os08g07890	1.6233	0.4333	-1.9054	0.001367	0.03643	SERK1
LOC_Os09g13890	1.0400	0.3167	-1.7155	0.001369	0.03643	CBP60C
LOC_Os01g26210	1.1267	0.1067	-3.4009	0.001379	0.03664	WAK3
LOC_Os02g21750	1.6000	0.3267	-2.2922	0.00138	0.03664	ABCB11
LOC_Os12g02320	3430.8967	7497.5300	1.1278	0.001392	0.03689	LTP
LOC_Os09g16950	4.9433	0.6633	-2.8977	0.001396	0.03692	LECRK91
LOC_Os01g71820	0.2733	1.4833	2.4401	0.001396	0.03692	-
LOC_Os01g27190	0.0010	0.2900	8.1799	0.0014	0.03701	-
LOC_Os12g35490	0.1300	0.0010	-7.0224	0.001408	0.03717	-
LOC_Os03g63260	1.4167	0.4967	-1.5122	0.001413	0.03727	PCMP-E76
LOC_Os01g49710	2.0033	10.8500	2.4372	0.001418	0.03736	GSTU6
LOC_Os06g32890	0.3533	0.0533	-2.7279	0.001421	0.0374	Tf2-6
LOC_Os01g42860	9.3867	77.4900	3.0453	0.001425	0.03744	-
LOC_Os04g51796	0.1200	1.5067	3.6503	0.001426	0.03745	-
LOC_Os01g44130	0.1833	1.3433	2.8733	0.00144	0.03772	Os05g0567100
LOC_Os07g48260	2.3233	0.7067	-1.7171	0.001441	0.03772	WRKY54
LOC_Os01g56235	1.3767	0.1100	-3.6456	0.00145	0.03792	At4g15545
LOC_Os11g46870	0.2700	0.0500	-2.4330	0.001457	0.03808	WAK5
LOC_Os09g15480	1.4500	14.0767	3.2792	0.001459	0.0381	Tango2
LOC_Os04g44150	0.0567	0.5000	3.1414	0.001465	0.0382	GA2OX8
LOC_Os02g30190	9.3000	0.9967	-3.2220	0.001466	0.0382	RIN4
LOC_Os01g47900	5.9900	1.3900	-2.1075	0.00147	0.03826	At5g24080
LOC_Os07g02690	0.0967	0.6800	2.8144	0.001474	0.03834	-
MSTRG.4623	1.3667	0.6233	-1.1326	0.00148	0.03846	--
LOC_Os05g45200	1.0167	4.6033	2.1788	0.001486	0.03857	UGT88A1
LOC_Os04g51410	8.2500	25.5033	1.6282	0.001492	0.03871	-
LOC_Os04g34490	0.5467	0.0167	-5.0356	0.001508	0.03907	At3g30340
LOC_Os02g56820	0.3467	1.3600	1.9720	0.001513	0.03915	At2g02240
LOC_Os11g10480	13.8900	81.6033	2.5546	0.001515	0.03917	ADH1
LOC_Os09g38620	3.2067	10.7300	1.7425	0.001516	0.03918	CPR
MSTRG.6468	160.2600	67.8867	-1.2392	0.001519	0.03923	Os06g0207000
LOC_Os10g12480	9.0833	4.4867	-1.0176	0.001532	0.0395	-
LOC_Os06g43320	2.7033	12.9433	2.2594	0.001538	0.03962	CYP71D55
LOC_Os10g39610	0.1433	1.6633	3.5366	0.001539	0.03963	-
LOC_Os12g25660	10.9533	1.3400	-3.0311	0.001547	0.03978	CYP94B3
LOC_Os02g07170	0.5467	3.4133	2.6424	0.001548	0.03978	KAN2
LOC_Os12g34290	0.1433	0.0010	-7.1632	0.001564	0.04012	At3g52670
LOC_Os03g22790	12.0667	44.7600	1.8912	0.001565	0.04012	BAM9
LOC_Os05g47840	0.6800	0.2067	-1.7182	0.001566	0.04012	IPT1
LOC_Os05g38150	17.0333	36.6767	1.1065	0.00158	0.04035	P5CS
LOC_Os06g28000	0.0300	0.3967	3.7249	0.00158	0.04035	-
LOC_Os06g46500	1.3967	4.2633	1.6100	0.001581	0.04035	-

LOC_Os09g14610	3.9900	1.9500	-1.0329	0.001582	0.04035	DCL2B
LOC_Os03g29930	11.4300	3.1867	-1.8427	0.00159	0.04052	-
LOC_Os03g16950	10.1367	44.5733	2.1366	0.001594	0.04055	CRRSP55
LOC_Os08g07240	0.1500	2.5067	4.0627	0.001594	0.04055	-
LOC_Os03g16670	21.0367	106.7667	2.3435	0.001604	0.04072	SDT1
LOC_Os04g58030	0.7267	2.7367	1.9131	0.001612	0.04088	-
LOC_Os05g01760	18.4400	45.4267	1.3007	0.001614	0.04088	-
LOC_Os12g37780	1.3200	2.7833	1.0763	0.001614	0.04088	-
LOC_Os10g37400	4.0500	13.9667	1.7860	0.001628	0.04114	-
LOC_Os08g35630	3.1000	10.3467	1.7388	0.001628	0.04114	-
LOC_Os10g19160	0.0300	0.1533	2.3536	0.001634	0.04125	At3g47570
LOC_Os03g57690	38.0833	96.8233	1.3462	0.00164	0.04134	Os03g0790900
LOC_Os02g04510	1.3600	13.8433	3.3475	0.001643	0.04135	DVR
LOC_Os03g26930	2.9033	1.0333	-1.4904	0.001644	0.04135	CXP;2-3
LOC_Os09g37394	0.3233	3.5567	3.4594	0.001652	0.04148	SAUR39
LOC_Os04g33030	77.5700	33.6133	-1.2065	0.001665	0.04177	BRG3
LOC_Os08g09860	25.4833	12.6000	-1.0161	0.001675	0.04199	GLO1
LOC_Os02g18410	0.8367	6.9300	3.0501	0.001687	0.04226	PCAP1
LOC_Os10g30880	13.6467	3.0233	-2.1743	0.001689	0.04227	-
LOC_Os12g43660	3.7533	1.2500	-1.5862	0.001699	0.04246	HSL1
LOC_Os03g63970	7.2400	16.2867	1.1696	0.001705	0.04256	20ox1
LOC_Os09g25070	13.1400	1.1133	-3.5610	0.001709	0.04261	WRKY40
LOC_Os05g19910	0.7700	2.1167	1.4589	0.001714	0.04267	TAT
LOC_Os09g24260	6.6867	13.9200	1.0578	0.001714	0.04267	-
LOC_Os04g07260	0.6967	0.1467	-2.2479	0.001732	0.04302	-
LOC_Os04g40990	7.2000	74.3200	3.3677	0.001756	0.04359	MS
MSTRG.5533	0.8767	0.0333	-4.7170	0.00176	0.04365	--
LOC_Os10g31320	1.6600	4.4967	1.4377	0.001763	0.04367	-
LOC_Os10g25180	0.0010	0.3133	8.2916	0.001769	0.04374	SAC2
LOC_Os06g48600	52.9867	25.0167	-1.0827	0.001771	0.04374	-
LOC_Os09g31490	3.8367	8.5367	1.1538	0.001772	0.04374	CCR1
LOC_Os06g46160	0.2400	0.0010	-7.9069	0.001779	0.04388	-
LOC_Os10g07340	4.2300	13.4900	1.6732	0.001787	0.04403	-
LOC_Os01g71790	14.0133	3.9500	-1.8269	0.001788	0.04403	NAC018
MSTRG.27573	1.6367	0.1433	-3.5133	0.001796	0.04413	--
LOC_Os05g03130	0.2000	2.6667	3.7370	0.001797	0.04413	LT101.2
LOC_Os03g17350	1.6100	6.2133	1.9483	0.001799	0.04413	RCN1
LOC_Os10g11920	0.0067	0.1900	4.8329	0.001801	0.04414	Tf2-6
MSTRG.10455	0.0400	1.0000	4.6439	0.001802	0.04414	--
LOC_Os02g27000	0.8533	2.1633	1.3421	0.00181	0.0443	Morc4
LOC_Os01g08470	0.5600	1.8400	1.7162	0.001828	0.04463	LRX4
LOC_Os07g29960	0.1767	0.0010	-7.4649	0.001829	0.04463	CYP87A3
LOC_Os05g38000	0.1500	2.4600	4.0356	0.00183	0.04463	ROPGEF7

LOC_Os07g44850	0.3700	2.5067	2.7602	0.001832	0.04463	CXE18
LOC_Os04g47080	0.0900	0.5500	2.6114	0.001833	0.04463	LC
LOC_Os04g33080	14.0867	36.0467	1.3555	0.001833	0.04463	Os04g0403701
LOC_Os05g11750	0.2533	0.0267	-3.2479	0.001865	0.04535	PBS1
ChrSy.fgenesh.gene.78	0.0100	0.2033	4.3458	0.001868	0.04538	-
LOC_Os10g40614	0.0867	1.7733	4.3548	0.001871	0.04542	EARLI1
LOC_Os12g31860	30.2433	72.3767	1.2589	0.001873	0.04542	UPS2
LOC_Os01g68960	0.2933	1.3733	2.2271	0.001877	0.04545	-
LOC_Os08g13250	0.7167	0.0867	-3.0478	0.001886	0.0456	BPM2
MSTRG.826	0.7067	1.7600	1.3165	0.001891	0.04566	--
LOC_Os03g58290	5.3533	0.3233	-4.0493	0.001898	0.0458	BX1
LOC_Os03g55180	7.0600	2.2567	-1.6455	0.0019	0.04581	-
LOC_Os04g28250	4.1367	19.8067	2.2594	0.001915	0.04613	Os04g0350100
LOC_Os07g25540	0.6033	2.1833	1.8555	0.001927	0.04638	YUC9
LOC_Os10g01800	0.0667	1.2433	4.2211	0.001936	0.04655	-
LOC_Os09g10950	2.1433	5.5667	1.3770	0.001939	0.04659	-
MSTRG.6459	23.5333	11.3200	-1.0558	0.001965	0.04714	--
MSTRG.5947	12.0233	4.7233	-1.3480	0.001969	0.04719	--
LOC_Os01g04200	6.7833	13.7400	1.0183	0.001972	0.04721	lactb2
LOC_Os07g33620	1.3233	3.6400	1.4598	0.001979	0.04727	CYP716B1
LOC_Os04g52460	12.9400	31.2933	1.2740	0.001987	0.04737	-
LOC_Os01g53650	0.0010	0.3000	8.2288	0.00199	0.04738	Os01g0738400
LOC_Os09g15044	0.0233	0.6767	4.8580	0.001991	0.04738	-
LOC_Os10g41170	0.1567	0.6833	2.1249	0.002004	0.04766	caa43
LOC_Os03g05225	16.5300	7.5567	-1.1293	0.002011	0.04774	-
LOC_Os06g40580	1.1933	0.2700	-2.1440	0.002021	0.04789	-
MSTRG.27746	9.9133	4.0533	-1.2903	0.002026	0.04797	--
LOC_Os02g34630	4.5833	10.2633	1.1630	0.00204	0.04827	TERF1
LOC_Os02g54870	0.1867	0.5833	1.6439	0.00205	0.04845	-
LOC_Os04g11400	55.5867	17.0433	-1.7055	0.002062	0.04869	-
LOC_Os03g49430	0.5833	1.7867	1.6149	0.002064	0.04869	prpf18
LOC_Os03g05880	0.6200	2.3467	1.9203	0.002065	0.04869	nicC
LOC_Os01g35230	1.1533	0.3867	-1.5766	0.002067	0.0487	At5g59530
LOC_Os04g56430	23.1800	95.3433	2.0403	0.002072	0.04876	CRRSP38
LOC_Os09g29520	6.1333	1.4867	-2.0446	0.002073	0.04876	WAK3
LOC_Os04g56470	0.3633	2.2033	2.6003	0.002084	0.04896	AAP2
LOC_Os08g03420	0.3967	1.7033	2.1024	0.002091	0.04908	At3g27220
LOC_Os05g05030	16.3133	2.8033	-2.5408	0.002108	0.04944	-
LOC_Os03g61410	0.0010	0.2633	8.0407	0.002115	0.04952	-
LOC_Os06g50910	0.1833	0.3700	1.0131	0.002115	0.04952	Os06g0724700
LOC_Os09g36810	0.8733	2.1900	1.3263	0.002124	0.0497	Os09g0539200

Table S3. Statistical data of total differentially expressed genes (DEGs) for WR_CK vs. WR_D.

id	WR_CK _mean	WR_D_ mean	log2(fc)	PValue	FDR	Symbol
LOC_Os02g58720	8.4100	0.6333	-3.7311	2.11E-32	5.96E-28	PER64
LOC_Os03g61160	6.9100	93.5767	3.7594	2.28E-27	3.23E-23	-
LOC_Os02g37480	1.3700	13.9600	3.3491	1.82E-25	1.72E-21	-
LOC_Os05g09704	42.7133	0.9867	-5.4360	7.05E-25	4.99E-21	APS1
LOC_Os09g31478	5.8033	32.0067	2.4634	2.03E-23	1.15E-19	-
LOC_Os10g13700	5.6800	46.0000	3.0177	5.23E-23	2.47E-19	PCKA
LOC_Os12g14440	39.9733	0.7067	-5.8219	1.03E-21	4.16E-18	SALT
LOC_Os05g33900	2.8333	0.2200	-3.6869	8.26E-21	2.92E-17	At5g07050
LOC_Os01g01650	52.5100	1.4100	-5.2188	2.41E-20	7.58E-17	IRL
LOC_Os06g16640	22.9667	0.3733	-5.9429	9.55E-20	2.70E-16	-
LOC_Os02g06210	0.0267	0.7567	4.8265	5.17E-19	1.33E-15	RLP2
LOC_Os01g06640	2.8867	13.4033	2.2151	6.79E-19	1.60E-15	BHLH51
LOC_Os03g57460	15.5900	2.3133	-2.7526	2.60E-18	5.66E-15	FLA16
LOC_Os05g07060	3.0233	0.1467	-4.3655	3.18E-18	6.43E-15	FLA11
LOC_Os01g54620	12.9100	1.6067	-3.0063	8.67E-18	1.64E-14	CESA4
LOC_Os01g62490	1.5800	0.0500	-4.9819	1.52E-17	2.70E-14	LAC12
LOC_Os01g62260	0.1300	4.2233	5.0218	3.44E-17	5.72E-14	-
LOC_Os03g01300	71.3733	6.1800	-3.5297	8.31E-17	1.31E-13	AZI1
LOC_Os11g46900	2.1567	0.0667	-5.0157	1.03E-16	1.49E-13	WAK5
LOC_Os05g04500	66.0467	2.2767	-4.8585	1.05E-16	1.49E-13	GSVIVT00037159001
LOC_Os03g13200	24.5633	0.3567	-6.1058	6.11E-16	8.23E-13	pod
LOC_Os02g49720	8.4933	62.1233	2.8707	7.21E-16	9.27E-13	ALDH2B7
LOC_Os08g26230	34.9133	8.1767	-2.0942	7.73E-16	9.50E-13	-
LOC_Os05g31750	137.1800	22.0500	-2.6372	8.06E-16	9.50E-13	ANN4
LOC_Os10g16974	59.6967	4.9500	-3.5921	9.05E-16	1.02E-12	CYP75B1
LOC_Os05g35290	7.4200	1.0367	-2.8395	2.08E-15	2.27E-12	ZB8
LOC_Os05g10210	71.3433	1.6867	-5.4025	2.81E-15	2.94E-12	APS1
LOC_Os01g32780	20.1500	88.4800	2.1346	1.34E-14	1.35E-11	At3g01520
LOC_Os03g43410	3.6967	0.5333	-2.7931	1.63E-14	1.59E-11	IAA12
LOC_Os07g45100	10.6267	2.4700	-2.1051	2.06E-14	1.94E-11	-
LOC_Os06g38160	7.5833	0.5233	-3.8570	5.06E-14	4.62E-11	LECRK82
LOC_Os08g33150	4.7133	0.2600	-4.1802	7.16E-14	6.29E-11	ODO1
LOC_Os06g48210	1.1433	6.6800	2.5466	7.51E-14	6.29E-11	Os06g0697200
LOC_Os06g38764	30.0833	2.8133	-3.4186	7.56E-14	6.29E-11	-
LOC_Os06g40170	29.0733	3.2467	-3.1627	7.81E-14	6.32E-11	PLD2
LOC_Os08g04500	5.9433	0.0433	-7.0997	8.54E-14	6.71E-11	TPS2
LOC_Os01g55000	2.2967	0.2167	-3.4060	9.18E-14	7.02E-11	-
LOC_Os10g01920	19.7933	0.6467	-4.9358	1.94E-13	1.44E-10	-

LOC_Os03g03790	7.6300	0.2733	-4.8029	2.06E-13	1.49E-10	AAE12
LOC_Os02g56380	5.0800	0.5167	-3.2975	2.33E-13	1.65E-10	WAK3
LOC_Os12g08760	105.2367	7.0367	-3.9026	2.58E-13	1.76E-10	At1g77060
LOC_Os03g60340	49.6133	2.7133	-4.1926	2.61E-13	1.76E-10	TBL33
LOC_Os11g07460	2.5833	0.4267	-2.5981	3.41E-13	2.25E-10	PCF3
LOC_Os05g37140	17.7967	59.6867	1.7458	4.13E-13	2.66E-10	FDX6
LOC_Os04g39320	12.5033	0.5700	-4.4552	5.44E-13	3.42E-10	-
LOC_Os06g39390	2.8433	0.0567	-5.6489	6.63E-13	4.08E-10	AMAT
LOC_Os04g26870	20.3467	2.0767	-3.2925	6.89E-13	4.15E-10	Os04g0337500
LOC_Os03g30250	2.6133	0.5567	-2.2310	7.54E-13	4.45E-10	BC1
LOC_Os04g54810	29.6300	0.6800	-5.4454	7.80E-13	4.50E-10	BXL4
LOC_Os07g23120	28.3000	3.1667	-3.1598	8.59E-13	4.86E-10	CDI
LOC_Os03g10320	4.2400	14.2400	1.7478	9.94E-13	5.51E-10	-
LOC_Os09g04050	11.2233	1.4700	-2.9326	1.06E-12	5.78E-10	CCR1
LOC_Os11g07670	10.1033	1.6767	-2.5912	1.37E-12	7.34E-10	DIR21
LOC_Os10g35950	29.9333	0.9667	-4.9526	1.62E-12	8.49E-10	HSR201
LOC_Os02g09359	11.4033	3.3400	-1.7715	1.73E-12	8.92E-10	At3g15890
LOC_Os04g28620	59.0133	1.1933	-5.6280	1.90E-12	9.58E-10	FAR1
LOC_Os02g41780	2.4400	0.1967	-3.6331	2.30E-12	1.14E-09	At1g06890
LOC_Os09g25000	6.1667	0.8500	-2.8590	2.35E-12	1.15E-09	PUB45
LOC_Os05g38230	32.8400	3.1367	-3.3881	4.18E-12	2.00E-09	AKR4C10
LOC_Os07g01370	4.1933	0.3600	-3.5420	4.80E-12	2.27E-09	PRX74
LOC_Os03g38470	3.1700	0.0010	-11.6303	5.01E-12	2.33E-09	At5g55050
LOC_Os01g22249	24.1833	3.5967	-2.7493	5.72E-12	2.61E-09	PER1
LOC_Os03g29150	6.8733	0.4933	-3.8004	6.04E-12	2.71E-09	3BETAHSD/D1
LOC_Os05g09740	134.8033	4.5200	-4.8984	6.12E-12	2.71E-09	APS1
LOC_Os01g18240	5.6233	0.8067	-2.8014	7.00E-12	3.05E-09	MYB86
LOC_Os04g43800	9.2967	0.7167	-3.6973	7.14E-12	3.06E-09	PAL
LOC_Os03g63540	6.7533	0.4400	-3.9400	8.31E-12	3.51E-09	-
LOC_Os05g02450	2.1867	8.6433	1.9829	1.34E-11	5.56E-09	-
LOC_Os11g38010	4.8767	0.4500	-3.4379	1.87E-11	7.65E-09	WVD2
LOC_Os06g11490	21.1567	0.9400	-4.4923	2.08E-11	8.41E-09	-
LOC_Os10g38540	8.5000	0.6200	-3.7771	2.17E-11	8.64E-09	GSTU6
LOC_Os07g14740	48.8300	11.9967	-2.0251	2.80E-11	1.09E-08	-
LOC_Os07g40940	8.3233	1.2067	-2.7861	2.81E-11	1.09E-08	PDCB3
LOC_Os04g31350	1.8533	0.0633	-4.8710	2.94E-11	1.12E-08	-
LOC_Os08g40680	0.4633	4.3067	3.2164	3.15E-11	1.18E-08	Chib3H-h
LOC_Os12g35350	2.1767	9.9633	2.1945	3.17E-11	1.18E-08	ACBP4
LOC_Os03g12680	5.4633	1.4333	-1.9304	3.22E-11	1.18E-08	At4g34500
LOC_Os07g07290	15.9300	3.6833	-2.1127	3.47E-11	1.26E-08	-
LOC_Os10g25040	0.9733	5.3500	2.4585	3.56E-11	1.28E-08	rccR
LOC_Os11g29840	2.1533	0.2000	-3.4285	4.05E-11	1.43E-08	-
LOC_Os06g14370	48.4700	12.7500	-1.9266	4.33E-11	1.51E-08	PXG4

LOC_Os11g27264	16.2567	2.1533	-2.9164	4.85E-11	1.67E-08	SCPL2
LOC_Os03g48770	19.1367	73.3167	1.9378	5.00E-11	1.71E-08	Os03g0693900
LOC_Os02g37800	15.7133	1.6933	-3.2141	5.08E-11	1.71E-08	Os03g0232800
LOC_Os01g14670	132.3667	9.1300	-3.8578	7.04E-11	2.34E-08	Os05g0277500
LOC_Os09g20684	10.4633	0.8667	-3.5937	7.95E-11	2.62E-08	SSL10
LOC_Os03g06940	2.1967	0.4867	-2.1743	8.15E-11	2.65E-08	Os03g0165400
LOC_Os03g13140	1.5433	23.0200	3.8988	8.49E-11	2.73E-08	HB1
LOC_Os06g48250	4.3467	14.2967	1.7177	9.12E-11	2.90E-08	SPAC644.07
LOC_Os03g44420	5.3400	1.0233	-2.3836	9.51E-11	2.99E-08	FTSZ2-1
LOC_Os02g05744	13.8600	1.3933	-3.3143	9.76E-11	3.04E-08	Os02g0151300
LOC_Os06g18670	142.6800	24.0967	-2.5659	1.09E-10	3.37E-08	GmIF7GT1
LOC_Os10g40710	1.9900	0.0533	-5.2216	1.30E-10	3.96E-08	EXPB2
LOC_Os12g01560	8.7600	0.6633	-3.7231	1.48E-10	4.47E-08	TBL34
LOC_Os11g01570	6.5433	0.4167	-3.9731	1.53E-10	4.56E-08	TBL34
LOC_Os09g25490	10.2000	2.6567	-1.9409	1.97E-10	5.81E-08	CESA9
LOC_Os06g09340	1.5067	0.0600	-4.6503	2.43E-10	7.09E-08	PME12
LOC_Os04g53800	8.0967	0.7400	-3.4517	2.49E-10	7.18E-08	BAN
LOC_Os01g47070	16.7533	1.8000	-3.2184	3.29E-10	9.38E-08	CHIT3
LOC_Os03g25490	0.1400	1.2333	3.1391	3.32E-10	9.38E-08	CYP734A1
LOC_Os04g02640	2.8633	0.1167	-4.6172	4.19E-10	1.17E-07	CUT1
LOC_Os04g20330	2.1867	0.4167	-2.3918	4.50E-10	1.24E-07	UGT92A1
LOC_Os01g68900	1.7800	0.1700	-3.3883	4.54E-10	1.24E-07	-
LOC_Os04g39150	2.6433	0.1333	-4.3092	4.54E-10	1.24E-07	MLP423
LOC_Os05g38410	0.7100	0.0010	-9.4717	4.96E-10	1.32E-07	LAC12
LOC_Os10g32980	9.1233	1.4833	-2.6207	4.96E-10	1.32E-07	CESA7
LOC_Os01g65690	254.5633	13.8267	-4.2025	5.07E-10	1.34E-07	DODA1
LOC_Os12g42876	174.0233	44.0333	-1.9826	5.51E-10	1.44E-07	Os12g0623900
LOC_Os03g03164	16.0133	2.4267	-2.7222	5.69E-10	1.48E-07	HOS66
LOC_Os12g40890	6.1000	1.4133	-2.1097	5.89E-10	1.51E-07	IAA30
LOC_Os07g48770	36.9500	5.8533	-2.6582	6.24E-10	1.59E-07	dfr1
LOC_Os03g32470	42.8200	5.1400	-3.0584	6.46E-10	1.63E-07	SRG1
LOC_Os06g46680	1.1867	0.0500	-4.5688	6.53E-10	1.64E-07	CYP77A3
LOC_Os08g06100	437.8933	38.0533	-3.5245	7.02E-10	1.73E-07	ROMT-9
LOC_Os08g13920	434.6467	89.2000	-2.2847	7.04E-10	1.73E-07	XTH8
LOC_Os12g02370	68.9500	6.0133	-3.5193	7.23E-10	1.76E-07	CHI3
LOC_Os04g32820	6.5233	0.2400	-4.7645	7.44E-10	1.80E-07	-
LOC_Os04g40100	20.3333	6.5500	-1.6343	7.61E-10	1.82E-07	At1g67900
LOC_Os07g41600	2.7433	0.2700	-3.3449	7.73E-10	1.84E-07	-
LOC_Os08g34550	5.7433	1.5200	-1.9178	7.83E-10	1.85E-07	ATL13
LOC_Os06g21270	31.2600	0.9267	-5.0761	8.71E-10	2.04E-07	-
LOC_Os06g43304	5.8567	1.0133	-2.5310	9.12E-10	2.12E-07	CYP71D10
LOC_Os01g67090	15.0567	1.1267	-3.7403	9.58E-10	2.20E-07	IQD14
LOC_Os03g48780	52.1133	137.6733	1.4015	1.00E-09	2.27E-07	Os03g0694000

LOC_Os07g44090	6.4533	1.2400	-2.3797	1.00E-09	2.27E-07	MYB86
LOC_Os11g02440	67.9933	9.2200	-2.8826	1.04E-09	2.34E-07	CHI3
LOC_Os08g43270	10.3767	2.6133	-1.9894	1.06E-09	2.35E-07	BAG1
LOC_Os04g59150	116.5333	42.7433	-1.4470	1.07E-09	2.35E-07	PER12
LOC_Os08g01274	2.9633	0.3433	-3.1095	1.07E-09	2.35E-07	-
LOC_Os01g59819	8.1400	0.9067	-3.1664	1.14E-09	2.48E-07	BGLU2
LOC_Os04g32800	1.6667	0.0833	-4.3219	1.22E-09	2.63E-07	-
LOC_Os01g01660	11.4967	1.3533	-3.0866	1.23E-09	2.63E-07	IRL
LOC_Os05g31760	85.4967	34.1900	-1.3223	1.30E-09	2.77E-07	ANN3
LOC_Os11g30560	14.5467	1.4633	-3.3134	1.34E-09	2.84E-07	HSD1
MSTRG.1894	16.3767	2.7767	-2.5602	1.35E-09	2.84E-07	--
LOC_Os11g30810	4.4467	0.4233	-3.3929	1.38E-09	2.87E-07	SOT5
LOC_Os05g29710	11.3633	65.1367	2.5191	1.60E-09	3.30E-07	ATL9
LOC_Os03g22010	15.8000	0.7367	-4.4228	1.62E-09	3.32E-07	PER70
LOC_Os02g33550	44.3200	2.0467	-4.4366	1.66E-09	3.37E-07	-
MSTRG.24111	1.8667	0.0767	-4.6057	1.74E-09	3.51E-07	--
LOC_Os04g33450	73.2600	5.1100	-3.8416	1.90E-09	3.82E-07	-
LOC_Os11g19850	3.6267	28.0300	2.9503	2.03E-09	4.02E-07	-
LOC_Os02g09260	0.4133	2.5700	2.6364	2.03E-09	4.02E-07	CYP71D8
LOC_Os02g09810	14.1467	57.4133	2.0209	2.15E-09	4.23E-07	SLC38A1
LOC_Os06g38960	4.5767	0.7067	-2.6952	2.24E-09	4.37E-07	-
LOC_Os03g12110	7.9433	1.9867	-1.9994	2.31E-09	4.48E-07	HMT-1
LOC_Os03g44150	9.4033	24.1867	1.3630	2.37E-09	4.56E-07	OAT
LOC_Os12g16080	5.1800	15.8400	1.6125	2.48E-09	4.74E-07	-
LOC_Os05g06110	2.3600	0.2400	-3.2977	2.69E-09	5.10E-07	VLN1
LOC_Os06g03640	51.0900	10.8900	-2.2300	2.83E-09	5.34E-07	BAG3
LOC_Os03g12510	45.8500	398.0333	3.1179	2.85E-09	5.34E-07	HB2
LOC_Os07g26110	2.2500	8.2300	1.8710	2.93E-09	5.46E-07	Os07g0442900
LOC_Os12g25690	20.4833	3.5767	-2.5178	3.28E-09	6.06E-07	UGD4
LOC_Os12g16720	8.3933	27.0533	1.6885	3.69E-09	6.70E-07	CYP71A9
LOC_Os11g08569	6.8633	1.4767	-2.2166	3.70E-09	6.70E-07	Os11g0189600
LOC_Os06g04090	24.1400	2.2833	-3.4022	3.71E-09	6.70E-07	NAC043
LOC_Os01g41900	24.6067	5.3700	-2.1961	3.72E-09	6.70E-07	DIVARICATA
LOC_Os11g26190	6.7133	0.6533	-3.3611	4.19E-09	7.50E-07	-
LOC_Os04g01140	57.6967	6.4300	-3.1656	4.37E-09	7.78E-07	CYP93A2
LOC_Os01g32460	31.6200	4.2367	-2.8998	4.93E-09	8.71E-07	-
LOC_Os02g53840	2.1733	0.3067	-2.8252	4.95E-09	8.71E-07	SBT1.7
LOC_Os12g17160	2.5867	16.3667	2.6616	5.45E-09	9.51E-07	SOT5
LOC_Os09g26999	2.8200	0.2033	-3.7938	5.48E-09	9.51E-07	GG3
LOC_Os05g33630	389.0733	163.4133	-1.2515	5.99E-09	1.03E-06	rihA
LOC_Os05g35010	6.0467	19.1767	1.6651	6.29E-09	1.08E-06	CYP71A1
LOC_Os11g35930	2.4167	0.1000	-4.5949	6.62E-09	1.13E-06	CHS
LOC_Os01g03680	5.1833	0.1867	-4.7953	6.69E-09	1.13E-06	RBBI3.3

LOC_Os04g43290	1.3933	0.0233	-5.9000	6.95E-09	1.17E-06	ARPC2B
LOC_Os01g53240	70.5967	8.4633	-3.0603	7.06E-09	1.18E-06	BURP3
LOC_Os05g49240	2.8233	0.0367	-6.2668	7.23E-09	1.20E-06	RL3
LOC_Os11g06820	0.7700	0.0467	-4.0444	7.28E-09	1.20E-06	LAX2
LOC_Os08g30080	12.5500	2.7600	-2.1849	7.74E-09	1.27E-06	At1g06650
LOC_Os07g47830	1.9533	0.3033	-2.6870	8.51E-09	1.39E-06	-
LOC_Os01g16170	11.7233	84.9133	2.8566	8.75E-09	1.42E-06	YPQ1
LOC_Os02g42190	4.9033	0.2900	-4.0796	8.82E-09	1.43E-06	WAK4
LOC_Os01g71810	1.8067	0.0533	-5.0821	9.01E-09	1.44E-06	-
LOC_Os01g18744	2.0767	0.1633	-3.6684	9.03E-09	1.44E-06	TAX10
LOC_Os02g33070	95.0700	302.3167	1.6690	9.49E-09	1.51E-06	At2g27730
LOC_Os04g59120	0.9467	0.0467	-4.3424	9.56E-09	1.51E-06	At1g25270
LOC_Os10g34480	52.5100	5.8800	-3.1587	1.05E-08	1.64E-06	CYP86B1
LOC_Os02g30100	2.4833	9.8067	1.9815	1.05E-08	1.64E-06	CYP81E7
LOC_Os08g07890	1.6233	0.4333	-1.9054	1.13E-08	1.75E-06	SERK1
LOC_Os05g38420	0.6867	0.0067	-6.6865	1.13E-08	1.75E-06	LAC12
LOC_Os06g38450	34.8267	8.2800	-2.0725	1.18E-08	1.81E-06	SAG39
LOC_Os01g12640	1.2900	0.2267	-2.5087	1.18E-08	1.81E-06	MAKR2
LOC_Os01g52710	2.0600	0.2600	-2.9861	1.19E-08	1.81E-06	GAUT15
LOC_Os02g54140	0.5800	3.3767	2.5415	1.25E-08	1.88E-06	HSP18.6
LOC_Os11g42550	186.7533	11.9633	-3.9644	1.34E-08	2.02E-06	DIR21
LOC_Os06g05930	52.3800	4.8633	-3.4290	1.36E-08	2.04E-06	-
LOC_Os09g25850	55.0967	9.8533	-2.4833	1.42E-08	2.11E-06	CER3
LOC_Os03g61720	86.6333	5.9333	-3.8680	1.43E-08	2.12E-06	GPAT3
LOC_Os04g32810	9.9567	0.7200	-3.7896	1.47E-08	2.16E-06	-
LOC_Os05g38530	2.7267	13.5267	2.3106	1.55E-08	2.28E-06	HSP70
LOC_Os02g38040	0.8933	3.6167	2.0174	1.59E-08	2.31E-06	IRL2
LOC_Os07g44410	36.8633	197.5233	2.4218	1.59E-08	2.31E-06	tolB
LOC_Os02g52730	1.3600	4.3533	1.6785	1.60E-08	2.31E-06	Os01g0357100
LOC_Os02g15950	2.3633	8.3000	1.8123	1.64E-08	2.36E-06	At3g61590
LOC_Os11g19730	4.7667	0.9667	-2.3019	1.65E-08	2.36E-06	-
LOC_Os08g02030	1.2000	14.0033	3.5447	1.73E-08	2.45E-06	At3g50280
LOC_Os03g46060	3.9567	32.7533	3.0493	1.75E-08	2.47E-06	tlp
LOC_Os08g38900	209.9733	43.1133	-2.2840	1.80E-08	2.53E-06	ROMT-15
LOC_Os04g46130	4.2267	0.4733	-3.1586	1.98E-08	2.78E-06	-
LOC_Os02g50490	21.4000	4.3400	-2.3018	2.02E-08	2.82E-06	GLU10
LOC_Os08g38170	10.0367	1.2433	-3.0130	2.03E-08	2.82E-06	guaA
LOC_Os01g21960	3.0733	0.9000	-1.7718	2.06E-08	2.84E-06	At5g18500
LOC_Os05g10730	19.6100	54.2767	1.4687	2.13E-08	2.92E-06	ABCC8
LOC_Os07g03458	1.9567	0.0567	-5.1098	2.14E-08	2.92E-06	-
LOC_Os08g14570	33.6533	10.7300	-1.6491	2.17E-08	2.95E-06	CPR
LOC_Os09g31482	46.9200	131.7667	1.4897	2.18E-08	2.95E-06	U2AF35A
LOC_Os08g05980	1.9667	13.4267	2.7713	2.20E-08	2.96E-06	-

LOC_Os07g46930	1.6567	0.0500	-5.0502	2.21E-08	2.96E-06	-
LOC_Os12g40550	23.3333	53.8100	1.2055	2.23E-08	2.97E-06	UAH
LOC_Os01g51570	139.2400	8.6367	-4.0110	2.25E-08	2.99E-06	-
LOC_Os04g38540	4.3300	20.3700	2.2340	2.34E-08	3.09E-06	Galm
LOC_Os01g08380	4.6633	0.4100	-3.5077	2.39E-08	3.13E-06	TAX10
LOC_Os05g43940	1.2900	0.0010	-10.3332	2.40E-08	3.13E-06	ZRP4
LOC_Os12g03899	9.3967	24.7033	1.3945	2.40E-08	3.13E-06	ZIFL1
LOC_Os12g03150	3.2767	0.0900	-5.1862	2.56E-08	3.33E-06	MYB306
LOC_Os12g03040	0.2900	1.6267	2.4878	2.63E-08	3.40E-06	ONAC010
LOC_Os06g29730	19.9867	2.6300	-2.9259	2.83E-08	3.63E-06	-
LOC_Os04g43490	6.8600	2.1233	-1.6919	2.84E-08	3.63E-06	At4g26100
LOC_Os07g32630	24.0900	2.7567	-3.1274	2.97E-08	3.78E-06	UGT71B7
LOC_Os09g09290	0.5267	0.1033	-2.3496	3.13E-08	3.98E-06	-
LOC_Os03g25330	4.2100	0.2867	-3.8764	3.46E-08	4.38E-06	PER2
LOC_Os04g01874	0.1733	1.4567	3.0710	3.62E-08	4.56E-06	LECRKS5
LOC_Os05g48270	19.0933	2.3767	-3.0061	3.69E-08	4.60E-06	At3g61750
LOC_Os01g22560	13.1067	0.0433	-8.2406	3.69E-08	4.60E-06	GPAT3
LOC_Os02g20540	1.8167	0.0010	-10.8271	3.86E-08	4.78E-06	FLA6
LOC_Os07g30950	2.9000	0.7900	-1.8761	3.87E-08	4.78E-06	CYP725A1
LOC_Os05g48850	3.8600	0.8967	-2.1060	3.96E-08	4.88E-06	NAC073
LOC_Os03g19290	3.2833	21.1367	2.6865	4.04E-08	4.94E-06	OEP162
LOC_Os04g30030	2.1333	0.1633	-3.7072	4.11E-08	5.01E-06	CRK10
LOC_Os05g45180	12.4367	0.6933	-4.1649	4.36E-08	5.30E-06	RhGT1
LOC_Os01g45730	12.0267	0.6700	-4.1659	4.52E-08	5.46E-06	Os01g0645000
LOC_Os02g41590	56.2233	15.6633	-1.8438	4.63E-08	5.58E-06	ADK2
LOC_Os01g11550	1.0533	0.1333	-2.9819	4.75E-08	5.70E-06	PCF5
LOC_Os01g06580	2.5233	0.2833	-3.1548	4.90E-08	5.86E-06	FLA11
LOC_Os06g49660	3.2667	0.8333	-1.9709	4.99E-08	5.94E-06	HSR201
LOC_Os04g03980	7.4933	2.2567	-1.7314	5.16E-08	6.11E-06	YUC3
LOC_Os06g13720	0.9067	5.3833	2.5699	5.40E-08	6.36E-06	Os06g0246500
LOC_Os12g07580	12.6567	1.3700	-3.2076	5.42E-08	6.36E-06	DIR3
LOC_Os08g05960	3.9400	24.4667	2.6346	6.07E-08	7.10E-06	-
LOC_Os06g40180	7.0400	0.6800	-3.3720	6.45E-08	7.51E-06	PLD2
LOC_Os03g18300	2.5433	0.3300	-2.9462	6.98E-08	8.09E-06	CHUP1
LOC_Os01g62200	28.7367	6.3900	-2.1690	7.13E-08	8.23E-06	UFC
LOC_Os09g26660	12.1733	5.1400	-1.2439	7.15E-08	8.23E-06	RBOHE
LOC_Os04g44354	7.3867	1.0933	-2.7562	7.40E-08	8.48E-06	UGT82A1
LOC_Os04g51150	17.4167	61.0900	1.8105	7.77E-08	8.87E-06	AOX1A
LOC_Os03g61920	8.9767	21.5667	1.2646	8.17E-08	9.28E-06	ETFA
LOC_Os10g21272	0.0010	1.2333	10.2683	8.30E-08	9.40E-06	-
LOC_Os05g13900	1.4200	0.0600	-4.5648	8.67E-08	9.78E-06	p200
LOC_Os07g44890	21.8133	5.9200	-1.8815	8.95E-08	1.01E-05	CXE18
LOC_Os01g09880	13.5567	1.0333	-3.7136	9.01E-08	1.01E-05	-

LOC_Os11g35890	1.2100	0.2333	-2.3745	9.15E-08	1.02E-05	GSO2
LOC_Os07g03368	1.9800	0.0667	-4.8924	9.29E-08	1.03E-05	-
LOC_Os02g08100	157.1067	32.6667	-2.2659	9.34E-08	1.03E-05	4CL3
LOC_Os04g33390	28.6533	5.9900	-2.2581	9.59E-08	1.06E-05	ADT6
LOC_Os01g71624	9.9600	78.0233	2.9697	1.00E-07	1.10E-05	-
LOC_Os12g04690	0.7300	0.0300	-4.6049	1.03E-07	1.12E-05	-
LOC_Os06g01360	24.3467	62.8067	1.3672	1.04E-07	1.14E-05	HGO
LOC_Os03g28980	2.1600	0.2100	-3.3626	1.07E-07	1.17E-05	tolB
LOC_Os10g42390	8.0067	0.9167	-3.1267	1.11E-07	1.20E-05	ATL28
LOC_Os11g15340	3.8667	12.6667	1.7119	1.13E-07	1.22E-05	AAMT1
LOC_Os08g38710	43.7200	264.2833	2.5957	1.16E-07	1.25E-05	RFS1
LOC_Os01g12280	9.6433	23.6133	1.2920	1.18E-07	1.26E-05	-
LOC_Os11g02070	4.5800	0.6600	-2.7948	1.20E-07	1.28E-05	At3g56230
LOC_Os04g50770	7.0100	1.0233	-2.7761	1.21E-07	1.28E-05	-
LOC_Os11g19840	8.6633	32.0033	1.8852	1.25E-07	1.32E-05	OMT2
LOC_Os02g09490	45.7667	10.3933	-2.1386	1.26E-07	1.32E-05	CAD2
LOC_Os07g22350	33.7933	72.4867	1.1010	1.28E-07	1.35E-05	At5g13110
LOC_Os10g39260	1.1300	0.0700	-4.0128	1.32E-07	1.38E-05	nep1
LOC_Os05g03610	22.7933	5.5700	-2.0329	1.33E-07	1.39E-05	PLC2
LOC_Os04g46780	14.2100	1.2333	-3.5263	1.35E-07	1.40E-05	PVA13
LOC_Os11g39990	19.7833	4.3000	-2.2019	1.36E-07	1.40E-05	PLP1
LOC_Os01g16180	17.5033	44.5967	1.3493	1.38E-07	1.42E-05	EMB1691
LOC_Os01g08780	5.6733	1.2867	-2.1406	1.45E-07	1.49E-05	IP5P3
LOC_Os11g19864	1.7667	9.0833	2.3622	1.47E-07	1.51E-05	GSTT3
LOC_Os06g45710	1.7800	6.4533	1.8582	1.53E-07	1.56E-05	-
LOC_Os02g21700	9.3300	2.0733	-2.1699	1.54E-07	1.56E-05	ANP2
LOC_Os10g33920	15.3967	5.6633	-1.4429	1.58E-07	1.60E-05	At1g06890
LOC_Os05g05030	16.3133	2.3233	-2.8118	1.59E-07	1.60E-05	-
LOC_Os04g16970	2.1033	0.2233	-3.2354	1.64E-07	1.65E-05	rnf12
LOC_Os09g10650	0.1467	0.0010	-7.1964	1.67E-07	1.67E-05	FAB1B
LOC_Os12g08260	38.2100	85.6900	1.1652	1.67E-07	1.67E-05	At5g09300
LOC_Os08g39840	30.4200	4.2833	-2.8282	1.72E-07	1.71E-05	CM-LOX1
LOC_Os04g07110	0.9400	0.1000	-3.2327	1.95E-07	1.93E-05	-
LOC_Os04g38220	31.7867	5.5633	-2.5144	1.99E-07	1.96E-05	STP5
LOC_Os07g14820	0.3833	0.0010	-8.5825	2.02E-07	1.99E-05	At3g47570
LOC_Os07g01660	24.2000	5.0667	-2.2559	2.07E-07	2.03E-05	DIR1
LOC_Os10g42190	11.4900	28.6067	1.3160	2.08E-07	2.03E-05	IRL5
LOC_Os04g12970	1.6100	11.0600	2.7802	2.15E-07	2.09E-05	UGT74E2
LOC_Os01g02930	0.6933	2.6133	1.9143	2.17E-07	2.10E-05	pomgnt2
LOC_Os03g02514	45.9933	15.8400	-1.5379	2.18E-07	2.11E-05	-
LOC_Os07g46950	5.8333	17.4567	1.5814	2.23E-07	2.15E-05	-
LOC_Os11g17970	9.8433	2.3867	-2.0441	2.33E-07	2.23E-05	NPF4.5
MSTRG.10545	0.7600	10.0000	3.7179	2.50E-07	2.39E-05	--

LOC_Os06g45184	2.2633	23.9133	3.4013	2.55E-07	2.42E-05	-
LOC_Os07g43070	15.0200	3.2967	-2.1878	2.56E-07	2.42E-05	-
LOC_Os01g68740	11.8267	3.0233	-1.9678	2.56E-07	2.42E-05	-
LOC_Os09g02180	2.1400	17.2167	3.0081	2.62E-07	2.48E-05	-
LOC_Os02g14840	4.2433	1.2700	-1.7404	2.64E-07	2.48E-05	Os02g0245800
LOC_Os06g49680	4.7100	12.4633	1.4039	2.71E-07	2.54E-05	-
LOC_Os11g32650	253.4500	40.0067	-2.6634	2.72E-07	2.54E-05	CHS1
LOC_Os12g02030	2.1900	0.4400	-2.3154	2.75E-07	2.56E-05	At3g56230
LOC_Os08g42990	0.1900	1.7567	3.2088	2.81E-07	2.61E-05	-
LOC_Os02g52840	2.8600	0.2933	-3.2854	2.87E-07	2.65E-05	FL
LOC_Os05g08370	54.4933	21.6567	-1.3313	2.87E-07	2.65E-05	CESA1
LOC_Os02g54060	12.6100	1.7333	-2.8629	2.91E-07	2.67E-05	CPN21
LOC_Os07g02970	2.3167	0.2667	-3.1189	2.94E-07	2.70E-05	-
LOC_Os07g41060	2.4400	0.1800	-3.7608	3.01E-07	2.75E-05	DFR
LOC_Os12g43440	13.5200	53.3633	1.9808	3.02E-07	2.75E-05	Os12g0628600
LOC_Os06g09920	3.5033	14.0400	2.0027	3.03E-07	2.75E-05	-
LOC_Os02g48870	22.4133	3.5567	-2.6558	3.09E-07	2.79E-05	ASPG1
LOC_Os04g35890	3.6133	0.4900	-2.8825	3.24E-07	2.92E-05	CCR4
LOC_Os10g23820	9.6333	1.2600	-2.9346	3.30E-07	2.96E-05	ACT-1
LOC_Os04g58870	1.2067	7.2633	2.5896	3.33E-07	2.98E-05	EXO70A1
LOC_Os09g38239	40.9500	5.3633	-2.9327	3.34E-07	2.98E-05	SOT5
LOC_Os02g12380	5.7367	17.5833	1.6159	3.40E-07	3.02E-05	-
LOC_Os02g43194	18.3833	1.2933	-3.8292	3.50E-07	3.11E-05	ALDH3F1
LOC_Os04g39980	15.3033	73.9100	2.2719	3.55E-07	3.14E-05	DAO
LOC_Os01g25484	899.1667	2085.6900	1.2139	3.58E-07	3.16E-05	Os01g0357100
LOC_Os09g26960	43.0800	4.4367	-3.2795	3.61E-07	3.17E-05	CYP75B1
LOC_Os01g45110	1.1800	5.4667	2.2119	3.64E-07	3.19E-05	TOGT1
LOC_Os05g36990	3.3567	0.3700	-3.1814	3.65E-07	3.19E-05	OFP13
LOC_Os05g11990	15.9167	3.6733	-2.1154	3.83E-07	3.34E-05	TTL1
LOC_Os05g06780	22.2267	3.0233	-2.8781	3.87E-07	3.36E-05	-
LOC_Os06g48300	3.7833	15.9933	2.0797	3.93E-07	3.40E-05	Os06g0698300
LOC_Os01g33160	2.1233	0.0500	-5.4083	3.94E-07	3.40E-05	At3g17210
LOC_Os04g56990	35.6833	115.3300	1.6924	4.01E-07	3.45E-05	PHL1
LOC_Os11g42500	272.6533	23.6000	-3.5302	4.05E-07	3.48E-05	DIR1
LOC_Os03g63970	7.2400	17.1533	1.2444	4.08E-07	3.49E-05	20ox1
LOC_Os12g32760	0.7733	8.0133	3.3732	4.19E-07	3.57E-05	PLT5
LOC_Os08g35760	69.3567	10.7300	-2.6924	4.22E-07	3.59E-05	GER5
LOC_Os04g28780	0.6267	0.0533	-3.5546	4.30E-07	3.64E-05	At2g19130
LOC_Os04g58860	18.8767	5.7933	-1.7041	4.31E-07	3.64E-05	-
LOC_Os10g39130	59.8000	23.8533	-1.3260	4.38E-07	3.69E-05	MADS56
LOC_Os10g05970	9.0233	0.0267	-8.4025	4.46E-07	3.74E-05	PRP4
LOC_Os01g41730	1.7700	0.3800	-2.2197	4.47E-07	3.74E-05	At2g42960
LOC_Os02g41670	2.2800	0.0800	-4.8329	4.69E-07	3.91E-05	ZB8

LOC_Os01g68598	1.5667	0.2433	-2.6867	4.74E-07	3.94E-05	EPFL9
LOC_Os06g13830	7.8667	1.2133	-2.6968	5.19E-07	4.31E-05	Os06g0247900
LOC_Os02g22380	44.7967	18.8567	-1.2483	5.55E-07	4.59E-05	pomgnt2
LOC_Os01g11730	0.4867	0.0010	-8.9268	5.57E-07	4.60E-05	At5g45910
LOC_Os01g55050	31.9833	11.9500	-1.4203	5.79E-07	4.76E-05	-
LOC_Os11g03300	0.3733	1.6900	2.1785	6.17E-07	5.06E-05	ONAC010
LOC_Os01g51840	5.1667	0.7067	-2.8701	6.22E-07	5.09E-05	IQD31
LOC_Os07g45060	31.5633	6.0933	-2.3729	6.47E-07	5.28E-05	At3g06035
LOC_Os01g73790	5.2100	2.2833	-1.1901	6.54E-07	5.31E-05	Os01g0969100
LOC_Os03g43650	15.4567	2.1500	-2.8458	6.55E-07	5.31E-05	LRX6
LOC_Os03g56900	18.3100	6.1200	-1.5810	6.70E-07	5.42E-05	RTNLB8
LOC_Os01g42790	3.8867	0.8267	-2.2332	6.75E-07	5.44E-05	SAG39
LOC_Os01g62290	2.5233	9.1867	1.8642	6.77E-07	5.45E-05	HSP70
LOC_Os09g07130	3.0833	0.3900	-2.9829	6.82E-07	5.47E-05	-
LOC_Os01g16714	6.9433	0.5300	-3.7116	7.18E-07	5.74E-05	YUC10
LOC_Os09g37180	0.6833	0.1167	-2.5502	7.29E-07	5.81E-05	ACT-2
LOC_Os06g11400	6.7933	1.8833	-1.8508	7.37E-07	5.86E-05	CURT1D
LOC_Os01g12820	0.3700	2.8900	2.9655	7.42E-07	5.88E-05	-
LOC_Os07g44440	6.7000	35.8233	2.4187	7.45E-07	5.89E-05	Os07g0638400
LOC_Os11g03230	4.5633	1.1033	-2.0482	7.76E-07	6.12E-05	APY3
LOC_Os03g38800	0.5733	0.0633	-3.1783	7.86E-07	6.18E-05	SPAC644.07
LOC_Os03g18980	4.2267	0.6500	-2.7010	8.02E-07	6.28E-05	ATK4
LOC_Os01g06310	1.1567	10.3000	3.1546	8.22E-07	6.43E-05	-
LOC_Os02g56370	3.9800	0.2400	-4.0517	8.41E-07	6.56E-05	WAK5
LOC_Os06g30950	14.8433	3.2533	-2.1898	8.48E-07	6.59E-05	At1g06890
LOC_Os07g44280	1.2633	0.0333	-5.2441	8.55E-07	6.63E-05	DIR5
LOC_Os09g25070	13.1400	0.8600	-3.9335	8.60E-07	6.65E-05	WRKY40
LOC_Os04g32830	1.2767	0.0010	-10.3182	8.71E-07	6.71E-05	-
LOC_Os02g43740	2.9467	1.0067	-1.5495	8.83E-07	6.79E-05	D6PKL2
LOC_Os01g15320	2.7800	0.5200	-2.4185	8.93E-07	6.85E-05	RALFL33
LOC_Os02g54254	7.8867	33.6833	2.0945	9.06E-07	6.92E-05	LKR/SDH
LOC_Os08g41880	37.5467	9.6633	-1.9581	9.07E-07	6.92E-05	PAP27
LOC_Os01g22010	387.0033	135.2100	-1.5171	9.10E-07	6.92E-05	SAM2
LOC_Os06g34120	37.4833	14.2333	-1.3970	9.20E-07	6.98E-05	At5g45910
LOC_Os03g08624	5.9433	37.0800	2.6413	9.39E-07	7.11E-05	Rv1106c
LOC_Os11g47560	0.6467	5.1067	2.9813	9.75E-07	7.36E-05	Chib3H-h
LOC_Os04g15920	7.0333	0.3900	-4.1727	9.84E-07	7.40E-05	CAD6
LOC_Os10g37210	16.0367	38.0033	1.2448	9.98E-07	7.49E-05	Os10g0516300
LOC_Os05g04380	85.8167	19.1133	-2.1667	1.01E-06	7.57E-05	PER2
LOC_Os03g13820	17.3767	8.2233	-1.0794	1.02E-06	7.64E-05	At5g57670
LOC_Os04g51880	89.7833	201.8933	1.1691	1.08E-06	8.02E-05	GALAK
LOC_Os01g68050	6.8133	0.7700	-3.1454	1.08E-06	8.02E-05	PROT1
LOC_Os05g47650	5.3400	17.0533	1.6751	1.10E-06	8.14E-05	Os05g0549800

LOC_Os05g05680	56.5300	15.0300	-1.9112	1.11E-06	8.22E-05	MAO1B
LOC_Os05g42000	16.6367	3.8133	-2.1252	1.13E-06	8.32E-05	PER3
LOC_Os11g26760	8.6767	29.2900	1.7552	1.15E-06	8.43E-05	RAB16C
LOC_Os03g14590	45.4667	22.0000	-1.0473	1.15E-06	8.43E-05	-
LOC_Os06g38830	0.6867	0.1267	-2.4386	1.17E-06	8.59E-05	At3g47570
LOC_Os01g59840	21.2033	7.5400	-1.4917	1.18E-06	8.60E-05	BGLU3
LOC_Os02g13800	1.1600	3.8367	1.7257	1.19E-06	8.64E-05	HSFC2A
LOC_Os02g52490	1.4100	0.1067	-3.7245	1.19E-06	8.64E-05	-
LOC_Os09g26980	2.3267	0.7967	-1.5462	1.21E-06	8.77E-05	CYP75B1
LOC_Os07g42430	0.9367	0.0010	-9.8714	1.22E-06	8.77E-05	GLO4
LOC_Os03g21710	2.5300	0.2833	-3.1586	1.23E-06	8.86E-05	WRKY70
LOC_Os08g41860	15.0233	2.6733	-2.4905	1.24E-06	8.93E-05	-
LOC_Os01g44050	17.3667	54.5600	1.6515	1.25E-06	8.96E-05	cysG2
LOC_Os11g26790	32.7233	275.2533	3.0724	1.26E-06	9.02E-05	RAB21
LOC_Os05g07810	1.2933	7.0833	2.4533	1.27E-06	9.02E-05	At3g01520
LOC_Os02g48470	73.4233	26.5433	-1.4679	1.27E-06	9.02E-05	-
LOC_Os06g38340	4.4967	0.9967	-2.1737	1.28E-06	9.10E-05	At3g47570
LOC_Os03g19250	69.1133	158.2433	1.1951	1.37E-06	9.67E-05	AAE7
LOC_Os03g60580	31.3467	141.6433	2.1759	1.39E-06	9.78E-05	ADF3
LOC_Os04g56060	1.4267	0.2933	-2.2820	1.39E-06	9.78E-05	At5g57670
LOC_Os01g02780	6.7633	2.2267	-1.6028	1.41E-06	9.88E-05	At1g67000
LOC_Os01g59150	22.8500	4.9600	-2.2038	1.41E-06	9.90E-05	TUBB4
LOC_Os06g36380	3.5167	15.3500	2.1260	1.42E-06	9.90E-05	-
LOC_Os03g27480	2.0400	0.1967	-3.3747	1.47E-06	0.0001	SCPL51
LOC_Os01g27190	0.0010	0.6733	9.3952	1.49E-06	0.0001	-
LOC_Os04g02900	67.3200	149.3500	1.1496	1.51E-06	0.0001	Os04g0119400
LOC_Os02g43170	50.9633	24.6100	-1.0502	1.52E-06	0.0001	BBX21
LOC_Os07g32620	33.3300	6.4933	-2.3598	1.59E-06	0.00011	RhGT1
LOC_Os01g55610	0.8000	0.1233	-2.6974	1.60E-06	0.00011	NPF1.2
LOC_Os02g37654	17.7367	3.7800	-2.2303	1.64E-06	0.00011	Os03g0232800
LOC_Os02g53200	15.8100	5.3200	-1.5713	1.64E-06	0.00011	At5g56590
LOC_Os06g19530	6.8633	1.0100	-2.7646	1.66E-06	0.00011	KCR1
LOC_Os06g20920	2.8633	0.4800	-2.5766	1.68E-06	0.00011	AAMT1
LOC_Os05g41990	0.5267	0.0010	-9.0407	1.68E-06	0.00011	PRX74
LOC_Os06g20200	38.5200	97.1367	1.3344	1.70E-06	0.00012	CXE18
LOC_Os01g70200	23.6733	7.1767	-1.7219	1.73E-06	0.00012	Os01g0926700
LOC_Os05g27000	1.5167	0.1333	-3.5078	1.75E-06	0.00012	-
LOC_Os04g58030	0.7267	3.9767	2.4522	1.78E-06	0.00012	-
LOC_Os08g26220	2.3667	0.1300	-4.1863	1.84E-06	0.00012	-
LOC_Os03g40670	12.3033	1.9833	-2.6331	1.86E-06	0.00012	GDPD6
LOC_Os10g34770	2.2767	14.2400	2.6450	1.88E-06	0.00013	grip22
LOC_Os09g27910	0.4167	0.0010	-8.7027	1.92E-06	0.00013	nep1
LOC_Os03g32460	0.4433	0.0010	-8.7922	1.93E-06	0.00013	-

LOC_Os06g12290	0.2533	1.9333	2.9320	1.94E-06	0.00013	GSTU10
LOC_Os04g39300	1.4067	0.0667	-4.3992	1.94E-06	0.00013	-
LOC_Os12g27220	20.6567	2.5400	-3.0237	1.95E-06	0.00013	HSR201
LOC_Os03g62330	1.0467	3.4867	1.7360	2.02E-06	0.00013	-
LOC_Os04g55670	27.4367	8.7367	-1.6510	2.04E-06	0.00013	Os04g0650300
LOC_Os02g18990	2.7533	0.3133	-3.1354	2.06E-06	0.00014	At5g41890
LOC_Os04g53760	14.2300	1.7633	-3.0126	2.08E-06	0.00014	ATK5
LOC_Os02g26210	26.6400	8.1300	-1.7123	2.12E-06	0.00014	Os02g0460200
LOC_Os11g12300	1.5833	0.1433	-3.4655	2.13E-06	0.00014	RPM1
LOC_Os05g01140	1.2467	0.0533	-4.5469	2.15E-06	0.00014	SAMT
LOC_Os11g04660	4.9567	1.1133	-2.1545	2.17E-06	0.00014	SSL10
LOC_Os08g33740	4.1233	0.7967	-2.3718	2.20E-06	0.00014	CSLA11
LOC_Os06g46900	2.2067	6.2233	1.4958	2.23E-06	0.00014	comA
LOC_Os05g33140	3.0300	0.8533	-1.8281	2.37E-06	0.00015	Cht9
LOC_Os07g03730	12.1267	1.4733	-3.0410	2.38E-06	0.00015	-
LOC_Os03g49830	6.2100	2.0533	-1.5966	2.45E-06	0.00016	At5g22090
LOC_Os11g47120	10.0267	2.8167	-1.8318	2.46E-06	0.00016	ACLA-1
LOC_Os09g29540	3.8033	0.7133	-2.4146	2.48E-06	0.00016	WAK5
LOC_Os01g01840	8.9233	0.9300	-3.2623	2.50E-06	0.00016	-
LOC_Os07g30760	11.8500	3.3867	-1.8069	2.51E-06	0.00016	UGT709C2
LOC_Os10g41660	0.4433	2.5200	2.5070	2.58E-06	0.00016	ATL46
LOC_Os04g11970	0.6233	0.0010	-9.2839	2.60E-06	0.00016	OMT3
LOC_Os02g48770	4.2333	0.2867	-3.8843	2.63E-06	0.00017	AAMT1
LOC_Os01g66760	6.7167	0.7733	-3.1186	2.70E-06	0.00017	At5g48380
LOC_Os06g43780	0.9167	0.1033	-3.1491	2.77E-06	0.00017	-
LOC_Os08g14000	66.5200	14.4033	-2.2074	2.84E-06	0.00018	-
LOC_Os06g50930	16.1333	43.3300	1.4253	2.89E-06	0.00018	STR15
LOC_Os08g40180	1.5533	0.1767	-3.1363	2.89E-06	0.00018	HMG3
LOC_Os06g09240	2.2367	0.4367	-2.3567	2.93E-06	0.00018	BZ1
LOC_Os04g39360	6.0167	0.1700	-5.1454	2.94E-06	0.00018	-
LOC_Os01g04300	7.4033	1.2100	-2.6132	2.98E-06	0.00018	XYL2
LOC_Os12g12080	2.7700	26.0867	3.2354	2.98E-06	0.00018	-
LOC_Os12g37350	68.1367	21.6467	-1.6543	2.98E-06	0.00018	LOX2.2
LOC_Os01g39020	1.1467	5.0767	2.1464	3.03E-06	0.00019	HSFA6B
LOC_Os07g39750	2.7233	0.6000	-2.1823	3.05E-06	0.00019	At3g26430
LOC_Os02g11700	1.9867	0.0900	-4.4643	3.29E-06	0.0002	UGT73C1
LOC_Os01g50200	8.7600	2.8433	-1.6233	3.32E-06	0.0002	RhGT1
LOC_Os01g50720	4.2267	0.4667	-3.1791	3.33E-06	0.0002	MYB2
LOC_Os11g10510	0.2000	1.1767	2.5566	3.36E-06	0.0002	ADH2
LOC_Os10g38340	10.4067	47.9267	2.2033	3.38E-06	0.00021	GSTU6
LOC_Os02g47390	0.8167	7.2967	3.1594	3.43E-06	0.00021	-
LOC_Os11g10520	0.8733	0.0200	-5.4485	3.48E-06	0.00021	ADH2
LOC_Os02g58390	0.7833	0.0300	-4.7066	3.49E-06	0.00021	At1g60630

LOC_Os12g41060	0.6067	0.0933	-2.7004	3.51E-06	0.00021	EREBP1
MSTRG.17789	0.0767	1.3233	4.1094	3.55E-06	0.00021	--
LOC_Os11g18366	8.5467	2.7200	-1.6518	3.67E-06	0.00022	Os11g0285000
LOC_Os02g40260	0.9167	0.0033	-8.1033	3.73E-06	0.00022	LRX3
LOC_Os03g02040	33.0833	5.5300	-2.5808	3.74E-06	0.00022	-
LOC_Os06g02780	32.0367	4.6933	-2.7710	3.81E-06	0.00023	ASPG2
LOC_Os08g05780	0.2933	1.7933	2.6120	3.84E-06	0.00023	-
LOC_Os07g04020	2.9267	0.7300	-2.0033	3.84E-06	0.00023	EPFL2
LOC_Os03g50970	2.0100	0.2867	-2.8097	3.85E-06	0.00023	MAP65-6
LOC_Os09g26920	1.3967	0.3533	-1.9829	3.95E-06	0.00023	SBT1.7
LOC_Os05g06720	11.7433	3.5100	-1.7423	4.11E-06	0.00024	At1g09390
LOC_Os10g38470	7.5933	21.3667	1.4926	4.14E-06	0.00024	GSTU6
LOC_Os08g01830	4.5367	1.4300	-1.6656	4.18E-06	0.00025	CCR4
LOC_Os01g45990	12.0567	3.4500	-1.8052	4.27E-06	0.00025	AKT1
LOC_Os08g38580	1.3400	0.2733	-2.2935	4.39E-06	0.00026	-
LOC_Os06g17390	11.8000	2.1533	-2.4541	4.43E-06	0.00026	At1g04910
LOC_Os01g09460	1.2267	4.1767	1.7676	4.54E-06	0.00026	HXK8
LOC_Os09g26370	27.4567	10.2900	-1.4159	4.55E-06	0.00026	-
LOC_Os07g42960	154.7467	49.3600	-1.6485	4.57E-06	0.00026	DAHPS2
LOC_Os09g13650	48.3467	4.0100	-3.5917	4.62E-06	0.00027	TPX2
LOC_Os05g27790	9.0967	34.9000	1.9398	4.65E-06	0.00027	Os05g0344400
LOC_Os05g10370	27.0900	5.2700	-2.3619	4.73E-06	0.00027	APS1
LOC_Os11g29400	34.9233	103.0067	1.5605	4.94E-06	0.00028	G6PGH2
LOC_Os02g05610	0.3700	1.5467	2.0636	4.96E-06	0.00028	SE
LOC_Os01g63880	4.2833	1.4467	-1.5660	5.04E-06	0.00029	-
LOC_Os12g37260	152.2767	44.1433	-1.7864	5.08E-06	0.00029	LOX2.1
LOC_Os03g19220	13.0167	2.8833	-2.1746	5.09E-06	0.00029	-
LOC_Os09g03939	14.4800	3.5400	-2.0322	5.12E-06	0.00029	ABCG11
LOC_Os07g09190	10.0467	1.1567	-3.1187	5.38E-06	0.00031	Os07g0190000
LOC_Os11g45990	0.4500	0.0010	-8.8138	5.42E-06	0.00031	-
LOC_Os10g38360	28.4767	102.6533	1.8499	5.45E-06	0.00031	GSTU6
LOC_Os03g04310	0.7533	0.0433	-4.1197	5.48E-06	0.00031	BHLH93
LOC_Os05g05040	27.9367	5.8867	-2.2466	5.57E-06	0.00031	-
LOC_Os12g06100	38.9600	81.5833	1.0663	5.59E-06	0.00031	TLDC1
LOC_Os07g34260	64.2700	0.7200	-6.4800	5.72E-06	0.00032	Os07g0271500
LOC_Os08g35750	9.8200	2.0933	-2.2299	5.72E-06	0.00032	Os08g0459700
LOC_Os11g42230	0.7600	0.0533	-3.8329	5.89E-06	0.00033	At4g26340
LOC_Os05g04820	4.4100	1.2200	-1.8539	5.95E-06	0.00033	MYB86
LOC_Os02g02840	14.2333	5.6500	-1.3330	6.17E-06	0.00034	RAC6
LOC_Os12g41740	0.6567	0.0010	-9.3590	6.24E-06	0.00035	-
LOC_Os02g43280	65.0967	17.2200	-1.9185	6.31E-06	0.00035	ALDH3F1
LOC_Os12g32590	1.0067	2.7800	1.4655	6.41E-06	0.00035	RPM1
LOC_Os05g39770	2.5100	12.0867	2.2677	6.72E-06	0.00037	At3g08860

LOC_Os09g33710	7.9567	1.1100	-2.8416	6.82E-06	0.00038	BGLU33
LOC_Os07g06680	44.4133	15.8000	-1.4911	6.87E-06	0.00038	FLA16
LOC_Os03g40194	3.9467	11.2933	1.5168	6.91E-06	0.00038	RGA2
LOC_Os02g37830	56.2267	19.0500	-1.5615	6.92E-06	0.00038	NEK6
LOC_Os03g25550	0.8567	0.0400	-4.4207	7.22E-06	0.00039	MYB86
LOC_Os04g31460	1.5633	0.1833	-3.0921	7.28E-06	0.0004	-
LOC_Os10g11270	1.6233	0.0567	-4.8403	7.45E-06	0.00041	SOT5
LOC_Os03g30830	3.6833	0.9400	-1.9703	7.46E-06	0.00041	PDCB3
LOC_Os05g16430	3.9767	14.7600	1.8921	7.48E-06	0.00041	At1g56130
LOC_Os07g41280	61.7100	148.4400	1.2663	7.64E-06	0.00041	Os07g0604000
LOC_Os12g18530	0.2133	0.8900	2.0607	7.70E-06	0.00042	-
LOC_Os06g34960	1.8767	0.3533	-2.4091	7.70E-06	0.00042	At1g67520
LOC_Os06g11990	15.8400	7.8600	-1.0110	7.84E-06	0.00042	-
LOC_Os02g17330	36.6233	80.5067	1.1363	8.11E-06	0.00044	DAAT
LOC_Os03g44710	9.7600	3.3267	-1.5528	8.26E-06	0.00044	YAB2
LOC_Os06g18780	3.0333	0.6367	-2.2523	8.47E-06	0.00045	-
LOC_Os12g42070	6.3467	2.9367	-1.1118	8.48E-06	0.00045	WAK2
LOC_Os07g03279	0.9400	0.0300	-4.9696	8.52E-06	0.00045	-
LOC_Os12g38400	9.2600	1.8900	-2.2926	8.56E-06	0.00046	RS2
LOC_Os05g03460	1.0267	3.0233	1.5582	8.63E-06	0.00046	At3g07070
LOC_Os01g58640	1.0533	4.0200	1.9322	8.80E-06	0.00047	PAP27
LOC_Os03g21740	11.1233	26.4567	1.2500	8.82E-06	0.00047	TWN2
LOC_Os05g49140	42.5733	7.1933	-2.5652	8.89E-06	0.00047	MPK7
LOC_Os12g25660	10.9533	0.9867	-3.4727	8.93E-06	0.00047	CYP94B3
LOC_Os09g38020	173.1567	406.2800	1.2304	9.03E-06	0.00048	-
LOC_Os10g37710	3.3400	0.7367	-2.1808	9.25E-06	0.00049	-
LOC_Os01g19610	9.5100	3.8433	-1.3071	9.29E-06	0.00049	At3g47200
LOC_Os02g41680	8.4167	2.5100	-1.7456	9.44E-06	0.00049	ZB8
LOC_Os10g37660	1.1533	5.5467	2.2658	9.46E-06	0.00049	Os10g0521000
LOC_Os01g51980	50.0200	10.8433	-2.2057	9.59E-06	0.0005	TAR4
LOC_Os07g25800	0.8267	4.5167	2.4499	9.69E-06	0.0005	At2g05970
LOC_Os05g02820	4.8233	1.1133	-2.1151	9.71E-06	0.0005	At5g16730
LOC_Os06g04920	4.0967	0.4667	-3.1340	9.77E-06	0.00051	SPAC17H9.04c
LOC_Os11g02350	9.1900	0.5733	-4.0026	1.00E-05	0.00052	LTP110-A
LOC_Os10g25780	7.0400	18.0000	1.3543	1.02E-05	0.00053	DIM
LOC_Os06g36040	1.6167	0.1033	-3.9676	1.05E-05	0.00054	-
LOC_Os03g04220	0.4833	0.0010	-8.9169	1.06E-05	0.00055	GSTF11
LOC_Os05g35050	0.0100	0.8933	6.4811	1.07E-05	0.00055	COPT2
LOC_Os01g52130	1.3100	0.1633	-3.0037	1.07E-05	0.00055	SULTR3;5
LOC_Os03g55070	148.8767	36.5000	-2.0281	1.07E-05	0.00055	UGD3
LOC_Os04g06500	0.4100	0.0067	-5.9425	1.15E-05	0.00059	-
LOC_Os10g26110	1.5333	0.0433	-5.1451	1.16E-05	0.00059	TYDC5
LOC_Os12g36940	3.7100	1.1500	-1.6898	1.16E-05	0.00059	CBP60B

LOC_Os11g04104	5.0900	13.6967	1.4281	1.17E-05	0.00059	ZIFL1
LOC_Os03g06705	0.5933	0.0010	-9.2127	1.19E-05	0.00061	KCS11
LOC_Os07g48460	5.1267	38.3800	2.9043	1.20E-05	0.00061	-
LOC_Os06g49000	3.0733	1.0800	-1.5088	1.20E-05	0.00061	-
LOC_Os10g40620	7.0467	1.5467	-2.1878	1.21E-05	0.00061	QWRF7
LOC_Os03g52860	0.2267	0.0010	-7.8244	1.24E-05	0.00062	LOX1.1
LOC_Os08g36760	14.7200	2.2700	-2.6970	1.25E-05	0.00063	-
LOC_Os10g37570	0.8933	0.0233	-5.2587	1.32E-05	0.00066	-
LOC_Os03g62490	5.3833	13.2933	1.3041	1.34E-05	0.00067	PHB2
LOC_Os12g41650	16.7333	40.5300	1.2763	1.34E-05	0.00067	PIF1
LOC_Os07g06175	0.4033	0.0010	-8.6558	1.34E-05	0.00067	PER2
LOC_Os01g71970	9.4033	19.1433	1.0256	1.35E-05	0.00067	SCL3
LOC_Os02g51910	8.7033	18.6500	1.0995	1.36E-05	0.00068	UGT85A24
LOC_Os01g05820	4.5833	0.8000	-2.5183	1.38E-05	0.00068	GGT3
LOC_Os11g07270	1.0233	2.7400	1.4209	1.38E-05	0.00068	At3g47570
LOC_Os06g32990	2.4633	0.1400	-4.1371	1.39E-05	0.00069	PER36
LOC_Os03g14730	108.9100	28.8200	-1.9180	1.40E-05	0.00069	CXE8
LOC_Os07g23150	1.2600	0.2900	-2.1193	1.40E-05	0.00069	3MAT
LOC_Os12g08200	2.0433	0.2400	-3.0898	1.41E-05	0.00069	-
LOC_Os02g42150	7.0733	1.1167	-2.6632	1.42E-05	0.0007	WAK5
LOC_Os12g40900	1.5700	0.3500	-2.1653	1.42E-05	0.0007	IAA31
LOC_Os02g34390	1.1433	0.0500	-4.5152	1.46E-05	0.00071	-
LOC_Os12g04440	13.7367	37.8500	1.4623	1.47E-05	0.00072	IPMSA
LOC_Os11g05800	4.5433	1.1300	-2.0074	1.48E-05	0.00072	HVA22J
LOC_Os06g06780	41.8933	13.2833	-1.6571	1.49E-05	0.00073	-
LOC_Os02g12420	4.2667	10.6933	1.3255	1.55E-05	0.00075	At3g47570
LOC_Os01g70850	3.5000	12.5567	1.8430	1.56E-05	0.00076	PIR7B
LOC_Os03g14030	22.0167	4.9967	-2.1396	1.57E-05	0.00076	-
LOC_Os12g32230	35.7700	80.6067	1.1721	1.59E-05	0.00077	CNX2
LOC_Os02g40100	6.8200	1.7333	-1.9762	1.59E-05	0.00077	FPP7
LOC_Os04g12580	2.4167	0.9200	-1.3933	1.59E-05	0.00077	RLK1
LOC_Os05g50800	13.4600	1.6467	-3.0311	1.60E-05	0.00077	Os05g0585400
LOC_Os08g37730	0.8700	0.2000	-2.1210	1.60E-05	0.00077	BHLH96
LOC_Os01g45640	8.2633	32.0600	1.9560	1.60E-05	0.00077	-
LOC_Os11g02424	287.2133	127.0867	-1.1763	1.62E-05	0.00078	ATL72
LOC_Os01g57050	0.9500	0.1067	-3.1548	1.64E-05	0.00079	-
LOC_Os07g02140	4.1533	1.5467	-1.4251	1.65E-05	0.00079	At5g07800
LOC_Os06g10670	2.8567	0.4633	-2.6242	1.65E-05	0.00079	nep1
LOC_Os10g38920	0.6833	0.0533	-3.6795	1.66E-05	0.00079	At1g01540
LOC_Os01g16030	2.5233	0.6067	-2.0564	1.69E-05	0.0008	ARF
LOC_Os02g53790	11.5967	4.0100	-1.5320	1.70E-05	0.00081	Os02g0778400
LOC_Os11g02520	2.6600	0.2267	-3.5528	1.71E-05	0.00081	WRKY46
LOC_Os11g12040	0.3867	0.0700	-2.4657	1.72E-05	0.00081	RPM1

LOC_Os07g27810	37.0467	9.5367	-1.9578	1.73E-05	0.00082	-
LOC_Os11g35500	1.7700	0.5067	-1.8046	1.79E-05	0.00084	At3g47570
LOC_Os03g27230	43.8367	14.0033	-1.6464	1.80E-05	0.00085	DAHPS1
LOC_Os04g28250	4.1367	0.6533	-2.6626	1.81E-05	0.00085	Os04g0350100
LOC_Os09g18450	1.2267	0.1200	-3.3536	1.83E-05	0.00086	NCS1
LOC_Os03g16920	0.4167	2.6467	2.6672	1.86E-05	0.00087	HSP70
LOC_Os09g07450	27.9567	4.7100	-2.5694	1.87E-05	0.00087	At3g50210
LOC_Os05g09724	15.9667	2.9600	-2.4314	1.88E-05	0.00088	APS1
LOC_Os05g29750	0.4467	2.6100	2.5468	1.90E-05	0.00089	CYP71B34
LOC_Os02g46600	3.6767	1.5033	-1.2902	1.91E-05	0.00089	Os04g0590900
LOC_Os01g73980	2.7933	0.5267	-2.4070	1.94E-05	0.0009	XCP2
MSTRG.5861	4.0333	11.7833	1.5467	1.94E-05	0.0009	--
LOC_Os11g38640	0.0467	0.4267	3.1926	1.94E-05	0.0009	-
LOC_Os11g14544	167.5767	389.4733	1.2167	1.96E-05	0.00091	At5g02620
LOC_Os09g36700	910.3900	297.8733	-1.6118	1.97E-05	0.00091	RNS1
LOC_Os07g40220	0.3000	2.0500	2.7726	1.97E-05	0.00091	-
LOC_Os02g49310	3.4300	1.1733	-1.5476	1.98E-05	0.00091	OsI_021818
LOC_Os09g32080	26.3433	10.4367	-1.3358	1.99E-05	0.00091	CTL1
LOC_Os06g11760	17.9033	2.1667	-3.0467	1.99E-05	0.00091	-
LOC_Os06g06850	0.5800	3.6667	2.6603	2.00E-05	0.00091	RPP13
LOC_Os11g47500	4.3700	24.6067	2.4933	2.00E-05	0.00091	RIXI
LOC_Os12g37519	5.1867	15.6367	1.5921	2.01E-05	0.00092	-
LOC_Os06g05550	16.4900	2.1167	-2.9617	2.04E-05	0.00093	APG
LOC_Os12g08280	25.6467	9.9300	-1.3689	2.05E-05	0.00093	Os12g0183300
LOC_Os11g17540	43.3000	7.9933	-2.4375	2.05E-05	0.00093	-
LOC_Os01g50680	0.2367	0.0010	-7.8867	2.06E-05	0.00093	SBT5.4
LOC_Os03g17790	3.3633	21.6100	2.6837	2.06E-05	0.00093	LT101.2
LOC_Os01g61620	2.7700	0.7067	-1.9708	2.07E-05	0.00093	pom1
LOC_Os02g08270	7.2333	0.4867	-3.8937	2.08E-05	0.00094	guaAA
LOC_Os08g39090	34.9067	71.8933	1.0424	2.10E-05	0.00095	Cwc25
LOC_Os02g37690	99.8500	30.8467	-1.6946	2.11E-05	0.00095	UGT91B1
LOC_Os03g07200	1.1367	3.4733	1.6115	2.12E-05	0.00095	-
LOC_Os01g52010	37.2367	15.5833	-1.2567	2.13E-05	0.00095	TAR4
LOC_Os05g40630	2.2667	0.3967	-2.5146	2.15E-05	0.00096	At2g38640
LOC_Os01g51210	3.7567	8.9367	1.2503	2.16E-05	0.00096	LOGL1
LOC_Os11g26340	4.4300	0.2633	-4.0723	2.26E-05	0.00101	-
LOC_Os04g37880	11.2067	1.6100	-2.7992	2.27E-05	0.00101	-
LOC_Os03g63370	24.1867	7.1233	-1.7636	2.27E-05	0.00101	rsc5
LOC_Os05g50830	1.3067	0.3933	-1.7321	2.32E-05	0.00102	PHY1
LOC_Os04g48260	2.0633	5.4733	1.4074	2.32E-05	0.00102	BB
LOC_Os06g01972	7.8233	1.1833	-2.7249	2.33E-05	0.00103	At3g30340
LOC_Os11g32620	1.2900	0.1367	-3.2386	2.34E-05	0.00103	Os07g0271500
LOC_Os04g52880	1.8900	0.3167	-2.5773	2.40E-05	0.00106	-

LOC_Os03g04060	36.0767	7.6833	-2.2313	2.41E-05	0.00106	Cht11
LOC_Os05g33130	0.6333	5.9467	3.2310	2.41E-05	0.00106	Cht2
LOC_Os12g37650	410.8367	116.6700	-1.8161	2.46E-05	0.00108	-
LOC_Os06g35520	38.1167	96.9533	1.3469	2.47E-05	0.00108	-
LOC_Os10g36880	7.5367	3.6800	-1.0342	2.48E-05	0.00108	kif11
LOC_Os01g61370	0.9200	0.1033	-3.1543	2.48E-05	0.00108	-
LOC_Os01g02350	1.4200	4.0233	1.5025	2.49E-05	0.00108	GDPDL2
LOC_Os03g01290	0.6167	0.1000	-2.6245	2.51E-05	0.00109	NPF6.3
LOC_Os02g07160	11.6733	37.0767	1.6673	2.51E-05	0.00109	-
LOC_Os03g64320	8.6100	2.4667	-1.8035	2.51E-05	0.00109	-
LOC_Os03g08100	10.1800	2.5367	-2.0047	2.52E-05	0.00109	todF
LOC_Os01g54030	0.4867	2.6800	2.4612	2.53E-05	0.00109	-
LOC_Os01g47780	5.5300	0.6933	-2.9957	2.54E-05	0.00109	FLA11
LOC_Os10g04429	0.4267	0.0167	-4.6781	2.55E-05	0.0011	HSR201
LOC_Os05g10670	6.9300	20.7667	1.5833	2.55E-05	0.0011	Os05g0195200
LOC_Os11g40930	4.5200	0.4467	-3.3391	2.57E-05	0.0011	-
LOC_Os03g60850	13.4333	4.7567	-1.4978	2.59E-05	0.00111	NPF5.7
LOC_Os03g25790	2.7833	6.9033	1.3105	2.61E-05	0.00112	At3g13560
LOC_Os11g14390	0.7067	0.0300	-4.5580	2.61E-05	0.00112	-
LOC_Os04g11956	2.6367	0.2467	-3.4181	2.62E-05	0.00112	-
LOC_Os01g43710	36.1767	81.4333	1.1706	2.64E-05	0.00113	CYP72A15
LOC_Os04g44320	5.4900	1.9300	-1.5082	2.65E-05	0.00113	YSL12
LOC_Os10g05750	1.4000	0.0300	-5.5443	2.65E-05	0.00113	PRP2
LOC_Os01g38229	55.3533	18.3700	-1.5913	2.69E-05	0.00114	FKBP70
LOC_Os09g08990	1.2033	0.1100	-3.4515	2.69E-05	0.00114	CYP75B1
LOC_Os12g04410	1.5033	0.3000	-2.3251	2.71E-05	0.00115	NPR6
LOC_Os09g31031	13.5667	45.5700	1.7480	2.72E-05	0.00115	Ub-CEP52-2
LOC_Os02g22820	22.5333	50.1600	1.1545	2.73E-05	0.00115	-
LOC_Os04g53210	3.1400	9.2733	1.5623	2.76E-05	0.00116	GLO3
LOC_Os10g38740	9.5267	31.1367	1.7086	2.77E-05	0.00116	GSTU6
LOC_Os03g12500	6.2100	0.8500	-2.8691	2.77E-05	0.00116	CYP74A2
LOC_Os03g21730	0.4500	0.1000	-2.1699	2.78E-05	0.00116	IRK
LOC_Os05g04510	349.8100	148.6267	-1.2349	2.79E-05	0.00117	SAM1
LOC_Os06g46740	12.8567	82.5800	2.6833	2.82E-05	0.00118	At4g27520
LOC_Os11g03060	5.2667	2.1433	-1.2970	2.83E-05	0.00118	VPS32.2
LOC_Os04g34490	0.5467	0.0010	-9.0945	2.83E-05	0.00118	At3g30340
LOC_Os03g62010	37.6867	12.4967	-1.5925	2.84E-05	0.00118	-
LOC_Os01g36460	11.9100	1.6400	-2.8604	2.84E-05	0.00118	MYB2
LOC_Os06g37020	3.8300	0.9267	-2.0472	2.98E-05	0.00124	-
LOC_Os03g45619	45.4667	1.2967	-5.1319	3.09E-05	0.00128	CYP87A3
LOC_Os02g08330	0.7533	2.5033	1.7325	3.13E-05	0.00129	MIMI_L728
LOC_Os02g25630	0.5700	0.0400	-3.8329	3.14E-05	0.00129	FT1
LOC_Os05g32110	52.5300	21.2700	-1.3043	3.15E-05	0.00129	BC1L4

LOC_Os02g37700	47.0567	10.8067	-2.1225	3.17E-05	0.0013	Os03g0232800
LOC_Os09g32100	1.5633	0.3433	-2.1869	3.21E-05	0.00132	-
LOC_Os02g51930	5.6967	26.5367	2.2198	3.22E-05	0.00132	UGT85A24
LOC_Os04g38310	9.6567	1.7733	-2.4451	3.25E-05	0.00133	-
LOC_Os10g35070	77.8900	243.6800	1.6455	3.26E-05	0.00133	AGAL1
LOC_Os03g56540	4.8400	15.1433	1.6456	3.26E-05	0.00133	TIM14-3
LOC_Os04g08280	1.8467	0.1833	-3.3324	3.26E-05	0.00133	-
LOC_Os02g17000	7.9400	1.0600	-2.9051	3.27E-05	0.00133	SBT3.3
LOC_Os04g34270	33.4933	3.8067	-3.1373	3.27E-05	0.00133	At2g19130
LOC_Os08g34280	30.7533	6.9233	-2.1512	3.28E-05	0.00133	CCR1
LOC_Os03g54040	31.4433	11.1600	-1.4944	3.32E-05	0.00134	RPL6
LOC_Os01g46120	25.7067	5.8100	-2.1455	3.33E-05	0.00135	At1g28600
LOC_Os07g46280	6.9667	17.6300	1.3395	3.37E-05	0.00136	BGLU26
LOC_Os11g06980	18.6233	6.6100	-1.4944	3.39E-05	0.00137	BURP17
LOC_Os07g34020	2.7667	0.6933	-1.9965	3.42E-05	0.00138	-
MSTRG.3995	0.0700	1.2333	4.1391	3.43E-05	0.00138	--
LOC_Os01g71710	8.2200	2.0367	-2.0129	3.48E-05	0.0014	BAT1
LOC_Os01g73110	28.1000	86.4300	1.6210	3.54E-05	0.00142	-
LOC_Os05g51610	12.0333	25.9000	1.1059	3.55E-05	0.00142	CAX1b
LOC_Os11g05700	11.3300	33.2167	1.5518	3.64E-05	0.00146	ABCC10
LOC_Os01g66840	3.9867	0.2300	-4.1155	3.66E-05	0.00146	PAE5
LOC_Os01g47470	6.1000	2.6700	-1.1920	3.73E-05	0.00149	At2g42960
LOC_Os02g33330	27.0900	10.0933	-1.4244	3.79E-05	0.00151	-
LOC_Os11g45400	2.2767	0.4233	-2.4271	3.84E-05	0.00153	GPAT3
LOC_Os03g50560	2.5767	0.8200	-1.6518	3.87E-05	0.00154	BPA1
LOC_Os01g62010	3.4567	0.2767	-3.6432	3.88E-05	0.00154	CSE
LOC_Os02g37180	2.8433	0.4800	-2.5665	3.94E-05	0.00156	-
LOC_Os11g29720	3.0533	1.0033	-1.6056	3.94E-05	0.00156	CYP78A5
LOC_Os03g14880	5.4533	1.8667	-1.5467	3.97E-05	0.00157	-
LOC_Os12g40860	0.2033	0.0010	-7.6677	3.98E-05	0.00157	FBL17
LOC_Os02g12730	1.1467	0.1067	-3.4263	3.99E-05	0.00157	Os02g0219200
LOC_Os12g04020	15.4767	7.5100	-1.0432	4.00E-05	0.00157	HMGCL
LOC_Os01g58100	0.8833	0.0733	-3.5904	4.10E-05	0.00161	-
LOC_Os11g05410	0.8467	3.2033	1.9197	4.13E-05	0.00162	PAP20
LOC_Os12g27254	2.5800	0.1733	-3.8958	4.17E-05	0.00163	HSR201
LOC_Os01g54080	4.7900	1.2900	-1.8927	4.21E-05	0.00165	KP1
LOC_Os05g03480	79.8767	180.6800	1.1776	4.22E-05	0.00165	Os05g0125500
LOC_Os11g29290	50.5700	10.8400	-2.2219	4.23E-05	0.00165	CYP94B3
LOC_Os05g46830	22.1900	3.7933	-2.5484	4.25E-05	0.00166	-
LOC_Os11g26860	139.5733	67.0167	-1.0584	4.27E-05	0.00166	SHM4
LOC_Os01g08440	5.1500	17.9633	1.8024	4.30E-05	0.00167	UGT75L6
LOC_Os03g12890	7.0233	20.8967	1.5730	4.32E-05	0.00167	BCAT5
LOC_Os05g18660	18.3800	37.2533	1.0192	4.39E-05	0.0017	-

LOC_Os01g15830	1.7667	0.3700	-2.2554	4.43E-05	0.00171	PER72
LOC_Os07g02060	0.9300	3.1400	1.7555	4.44E-05	0.00172	WRKY57
LOC_Os11g30290	0.0233	2.1467	6.5236	4.46E-05	0.00172	At1g07170
LOC_Os01g19020	170.3500	49.6800	-1.7778	4.48E-05	0.00172	PER2
LOC_Os04g02620	37.5600	6.8800	-2.4487	4.50E-05	0.00173	KCR1
LOC_Os11g03440	2.5500	0.2967	-3.1036	4.56E-05	0.00175	MYB306
LOC_Os01g43740	2.4800	12.2667	2.3063	4.57E-05	0.00175	CYP72A13
LOC_Os09g27510	0.2467	0.9767	1.9853	4.60E-05	0.00176	CYP76M5
LOC_Os05g12400	58.5567	10.7600	-2.4442	4.66E-05	0.00178	BURP1
LOC_Os01g43350	2.1867	1.0100	-1.1144	4.67E-05	0.00178	PHY1
LOC_Os10g12190	0.4733	2.0633	2.1240	4.68E-05	0.00178	At3g30340
LOC_Os01g32670	14.1500	45.1867	1.6751	4.68E-05	0.00178	-
LOC_Os02g58170	1.4300	4.4100	1.6248	4.74E-05	0.0018	Os03g0733400
LOC_Os09g36350	29.0033	12.4567	-1.2193	4.75E-05	0.0018	Os09g0533900
LOC_Os01g03390	103.4300	295.2667	1.5134	4.76E-05	0.0018	RBBI3.3
LOC_Os07g34850	1.1367	0.1833	-2.6323	4.78E-05	0.00181	nep1
LOC_Os03g05530	17.8533	5.8767	-1.6031	4.79E-05	0.00181	At2g39510
LOC_Os08g39730	3.2700	14.6100	2.1596	4.88E-05	0.00184	CYP76M5
LOC_Os11g11960	23.0100	5.0933	-2.1756	4.89E-05	0.00184	RPM1
LOC_Os11g47550	0.1233	1.3500	3.4523	4.90E-05	0.00184	Chib3H-h
LOC_Os03g12250	32.1200	10.5867	-1.6012	4.90E-05	0.00184	RKL1
LOC_Os01g22570	7.6533	0.0867	-6.4645	4.91E-05	0.00184	GPAT3
LOC_Os08g31030	0.4667	0.0467	-3.3219	4.91E-05	0.00184	HTH
LOC_Os09g26210	23.4833	9.7033	-1.2751	4.92E-05	0.00184	ZFP2
LOC_Os09g26880	95.1767	245.5133	1.3671	4.96E-05	0.00186	-
LOC_Os12g07810	1.5067	0.1700	-3.1478	4.97E-05	0.00186	ALDH3H1
LOC_Os03g10640	15.3100	2.5133	-2.6068	5.01E-05	0.00187	ACA2
LOC_Os10g39420	9.7867	3.4533	-1.5028	5.02E-05	0.00187	SPK
LOC_Os01g66720	3.0133	0.6833	-2.1407	5.03E-05	0.00187	DBR
LOC_Os05g03920	18.9233	73.9567	1.9665	5.03E-05	0.00187	CRK2
LOC_Os04g58790	3.2733	0.3667	-3.1582	5.08E-05	0.00189	-
LOC_Os06g39240	1.7767	7.0133	1.9809	5.13E-05	0.0019	MBF1C
LOC_Os02g57350	0.7300	0.0200	-5.1898	5.16E-05	0.00191	-
LOC_Os07g37350	2.6500	1.0367	-1.3540	5.30E-05	0.00196	-
LOC_Os06g28820	5.5067	1.2733	-2.1126	5.33E-05	0.00197	Os11g0285000
LOC_Os04g54800	17.5567	3.4500	-2.3474	5.36E-05	0.00197	SK3
LOC_Os04g58390	8.3367	2.9233	-1.5119	5.37E-05	0.00198	ALN
LOC_Os01g47080	5.9467	2.5033	-1.2482	5.41E-05	0.00199	PKP2
LOC_Os09g36250	9.3300	3.6700	-1.3461	5.43E-05	0.00199	ODO1
LOC_Os03g43750	0.3467	1.1467	1.7258	5.43E-05	0.00199	-
LOC_Os11g04380	0.3000	0.0033	-6.4919	5.49E-05	0.00201	At4g32390
LOC_Os02g38920	28.2467	72.2700	1.3553	5.51E-05	0.00202	GAPC3
LOC_Os11g15570	23.2400	4.6367	-2.3255	5.53E-05	0.00202	At1g18480

LOC_Os03g02460	1.6067	0.3200	-2.3279	5.57E-05	0.00203	TIC32
LOC_Os09g03960	2.5433	0.8233	-1.6272	5.59E-05	0.00203	-
LOC_Os10g39300	0.8067	0.0667	-3.5969	5.59E-05	0.00203	nep1
LOC_Os09g19350	0.3300	0.0010	-8.3663	5.64E-05	0.00205	At1g51890
LOC_Os12g16200	59.7400	23.5633	-1.3422	5.74E-05	0.00208	GSH2
LOC_Os05g37470	9.4800	4.2867	-1.1450	5.75E-05	0.00208	Os05g0447200
LOC_Os02g48900	70.2000	21.9800	-1.6753	5.76E-05	0.00208	ASPG1
LOC_Os03g13250	4.6800	9.9667	1.0906	5.76E-05	0.00208	NPF8.3
LOC_Os02g46970	23.9700	6.2567	-1.9378	5.82E-05	0.0021	4CL2
LOC_Os04g55600	68.9633	182.0700	1.4006	5.85E-05	0.00211	-
LOC_Os05g32420	0.7200	0.0700	-3.3626	5.90E-05	0.00212	-
LOC_Os05g13620	1.2933	0.1433	-3.1736	6.08E-05	0.00218	-
LOC_Os11g02540	5.6433	0.4100	-3.7829	6.14E-05	0.00221	WRKY70
LOC_Os01g28500	154.2000	309.0700	1.0031	6.16E-05	0.00221	PRMS
LOC_Os12g43450	15.8867	59.4867	1.9047	6.16E-05	0.00221	tlp
LOC_Os04g41620	60.1367	150.5300	1.3237	6.19E-05	0.00222	Cht4
LOC_Os01g71474	2.9333	0.2200	-3.7370	6.22E-05	0.00222	-
LOC_Os02g36619	0.8033	3.1767	1.9834	6.27E-05	0.00224	-
LOC_Os05g41220	54.0767	121.3767	1.1664	6.27E-05	0.00224	KINB1
LOC_Os02g44680	2.3067	0.6833	-1.7551	6.45E-05	0.00229	NCS1
LOC_Os06g04370	0.0600	0.3933	2.7127	6.46E-05	0.00229	NIK2
LOC_Os04g11400	55.5867	19.7900	-1.4900	6.48E-05	0.0023	-
LOC_Os06g07960	2.1033	0.4033	-2.3826	6.56E-05	0.00232	At3g57050
LOC_Os11g26850	287.8033	113.1633	-1.3467	6.61E-05	0.00234	SAHH
LOC_Os04g27060	8.3233	28.9533	1.7985	6.62E-05	0.00234	Os04g0339400
LOC_Os04g33640	19.0767	6.1100	-1.6426	6.64E-05	0.00234	At2g27500
LOC_Os09g36880	3.7333	0.9033	-2.0471	6.70E-05	0.00236	EXL3
LOC_Os06g08080	30.1200	7.7533	-1.9578	6.70E-05	0.00236	-
LOC_Os11g34020	0.7800	0.1067	-2.8704	6.75E-05	0.00237	BOI
LOC_Os12g05210	1.3867	11.0500	2.9944	6.75E-05	0.00237	-
LOC_Os08g07720	0.1000	1.1567	3.5319	6.80E-05	0.00239	PMAT2
LOC_Os08g10290	8.6467	1.6633	-2.3781	6.93E-05	0.00243	At1g56130
LOC_Os10g32550	8.6133	17.8700	1.0529	6.94E-05	0.00243	CPN60II
LOC_Os07g33480	7.9833	22.6767	1.5061	7.09E-05	0.00248	CYP716B1
LOC_Os10g32110	6.3367	2.5067	-1.3380	7.11E-05	0.00248	Os03g0144800
LOC_Os10g26500	0.6867	0.1433	-2.2602	7.13E-05	0.00248	HOX23
LOC_Os01g62770	0.8467	2.5533	1.5925	7.21E-05	0.00251	-
LOC_Os06g04169	1.1833	0.1733	-2.7712	7.23E-05	0.00251	xylF
LOC_Os06g29790	4.3733	1.6900	-1.3717	7.38E-05	0.00256	PHO1-3
LOC_Os12g33300	4.0733	0.3233	-3.6551	7.50E-05	0.0026	At5g64700
LOC_Os03g60090	116.7767	47.7067	-1.2915	7.52E-05	0.0026	Os03g0815200
LOC_Os06g19070	43.3867	11.1333	-1.9624	7.56E-05	0.00261	CYP76C4
LOC_Os03g48760	0.2333	1.8067	2.9529	7.63E-05	0.00263	Os03g0694000

LOC_Os09g30486	2.0933	0.3333	-2.6508	7.64E-05	0.00263	FLA7
LOC_Os07g12780	44.0767	135.3567	1.6187	7.65E-05	0.00263	CYCP4-1
LOC_Os06g18930	1.4500	0.5400	-1.4250	7.65E-05	0.00263	-
LOC_Os11g40009	7.8067	2.6133	-1.5788	7.68E-05	0.00264	PLP1
LOC_Os06g36390	63.9700	153.0500	1.2585	7.72E-05	0.00264	-
LOC_Os11g27370	4.3700	1.5300	-1.5141	7.83E-05	0.00268	RT
LOC_Os06g38780	1.6967	0.5667	-1.5821	7.93E-05	0.00271	At3g47570
LOC_Os09g18360	0.6067	0.0600	-3.3379	8.00E-05	0.00273	At1g51860
LOC_Os05g01350	0.9100	2.2733	1.3209	8.08E-05	0.00276	FH14
LOC_Os05g28320	0.5200	0.0133	-5.2854	8.18E-05	0.00278	MYB44
LOC_Os01g25500	8.8267	2.1300	-2.0510	8.20E-05	0.00279	-
LOC_Os09g27040	6.5333	20.4600	1.6469	8.35E-05	0.00284	GEX1
LOC_Os01g65210	22.9033	6.5433	-1.8075	8.42E-05	0.00286	NPF5.10
LOC_Os11g10800	1.3733	0.1600	-3.1015	8.52E-05	0.00289	DIR1
LOC_Os10g37190	12.1567	3.7200	-1.7084	8.60E-05	0.00291	At1g33260
LOC_Os10g12750	6.5500	0.8233	-2.9919	8.61E-05	0.00291	-
LOC_Os01g58500	2.7800	0.7467	-1.8965	8.63E-05	0.00292	-
LOC_Os01g22640	21.4767	4.6700	-2.2013	8.69E-05	0.00293	At1g09390
MSTRG.26475	2.7767	0.4567	-2.6041	8.74E-05	0.00295	--
LOC_Os03g04030	4.0800	8.4133	1.0441	8.76E-05	0.00295	HVA22I
LOC_Os09g07350	32.1667	6.6567	-2.2727	8.89E-05	0.00299	FLA2
LOC_Os02g07830	3.4867	1.4500	-1.2658	8.92E-05	0.003	HKT6
LOC_Os02g09830	0.1767	1.2467	2.8190	9.01E-05	0.00302	BZIP43
LOC_Os06g05980	1.6567	0.4033	-2.0382	9.01E-05	0.00302	At1g06470
LOC_Os12g08720	6.8633	1.2067	-2.5079	9.04E-05	0.00302	PRL1-IFG
LOC_Os04g46830	0.6467	0.0133	-5.5999	9.04E-05	0.00302	At4g12490
LOC_Os02g49510	0.4800	0.0633	-2.9220	9.20E-05	0.00307	SLC38A1
LOC_Os01g53170	0.8433	0.1733	-2.2826	9.33E-05	0.00311	-
LOC_Os01g46130	10.6967	3.0500	-1.8103	9.39E-05	0.00313	-
LOC_Os01g26210	1.1267	0.2733	-2.0433	9.43E-05	0.00314	WAK3
LOC_Os01g14630	9.0000	0.9367	-3.2643	9.51E-05	0.00316	GGPPS1
LOC_Os06g07790	4.3500	1.5333	-1.5043	9.62E-05	0.00319	-
LOC_Os03g11600	0.9433	0.2233	-2.0786	9.72E-05	0.00322	DL
LOC_Os06g06980	188.6867	69.0333	-1.4506	9.75E-05	0.00322	CCOAOMT2
LOC_Os10g30390	0.0433	1.3100	4.9179	9.82E-05	0.00324	CYP71Z7
LOC_Os03g61740	1.3300	0.1967	-2.7576	9.96E-05	0.00328	SDR5
LOC_Os04g46350	3.8200	1.6967	-1.1709	0.0001	0.0033	HOX17
LOC_Os02g04840	21.2833	43.9633	1.0466	0.000101	0.00334	ARA1
LOC_Os07g10770	47.9233	17.3900	-1.4625	0.000102	0.00335	CESA8
LOC_Os06g30830	0.4033	2.0100	2.3172	0.000102	0.00335	AGL61
LOC_Os08g41280	40.9100	17.0567	-1.2621	0.000103	0.00338	At3g25290
LOC_Os11g48000	1.2733	0.3467	-1.8770	0.000104	0.00339	RBE
LOC_Os04g42610	16.9433	5.2900	-1.6794	0.000104	0.00341	FPP7

LOC_Os08g29570	29.4200	6.4733	-2.1842	0.000105	0.00344	ABCG44
LOC_Os11g05190	1.4800	0.2433	-2.6046	0.000106	0.00345	PSK2
LOC_Os07g32010	11.1667	25.0500	1.1656	0.000106	0.00346	RhGT1
LOC_Os06g21250	3.8967	0.2633	-3.8873	0.000107	0.00346	-
LOC_Os06g21360	0.2133	0.0010	-7.7370	0.000107	0.00346	NHX4
LOC_Os10g06000	9.6200	0.1100	-6.4505	0.000107	0.00346	PRP4
LOC_Os03g36279	0.2033	2.3267	3.5163	0.000109	0.00352	GIN1
LOC_Os09g27820	42.5033	12.7933	-1.7322	0.000109	0.00354	ACO1
LOC_Os03g61260	328.4800	139.3167	-1.2374	0.00011	0.00356	RPL18
LOC_Os02g57120	37.5733	8.9900	-2.0633	0.00011	0.00356	CLASP
LOC_Os06g06560	1.1967	3.7067	1.6311	0.000111	0.00359	Os06g0160700
LOC_Os08g16260	23.7033	0.7633	-4.9566	0.000112	0.00361	CYP96A15
LOC_Os07g34320	0.9900	0.1300	-2.9289	0.000112	0.00361	At5g64700
LOC_Os01g38580	0.2400	1.7233	2.8441	0.000112	0.00361	CCD8A
LOC_Os07g41360	7.6600	2.7500	-1.4779	0.000112	0.00361	UAM3
LOC_Os01g38530	55.9600	115.4067	1.0443	0.000113	0.00365	ELF3
LOC_Os10g02814	0.2133	0.7667	1.8455	0.000114	0.00365	XLG3
LOC_Os02g04780	0.1400	0.9533	2.7676	0.000114	0.00366	-
LOC_Os02g37000	3.6333	9.2567	1.3492	0.000114	0.00366	PHB3
LOC_Os10g39750	1.6833	0.2533	-2.7322	0.000115	0.00367	BHLH93
LOC_Os02g55330	7.3367	3.4000	-1.1096	0.000115	0.00367	abhd17c
LOC_Os04g51140	2.0000	5.2533	1.3932	0.000115	0.00368	-
LOC_Os11g46000	0.2667	0.0033	-6.3219	0.000115	0.00368	-
LOC_Os01g04230	1.2967	0.3167	-2.0338	0.000116	0.00369	At1g68400
LOC_Os07g41460	4.2700	11.7633	1.4620	0.000117	0.00374	SOT5
LOC_Os01g68290	0.1733	1.7067	3.2996	0.000118	0.00374	-
LOC_Os07g42200	26.3367	12.4033	-1.0863	0.000118	0.00375	At5g01020
LOC_Os11g12320	2.1067	0.2900	-2.8608	0.000119	0.00378	RPM1
LOC_Os09g07460	3.5833	1.5833	-1.1783	0.00012	0.00379	NSP5
LOC_Os11g04600	1.4833	0.1700	-3.1252	0.00012	0.00379	NPR6
LOC_Os12g28015	214.2967	519.0133	1.2762	0.000122	0.00386	-
LOC_Os06g18010	61.7933	18.0133	-1.7784	0.000123	0.00387	GmIF7GT1
LOC_Os10g17260	7.4067	1.7733	-2.0624	0.000123	0.00389	CYP75B1
LOC_Os07g09050	0.4067	1.6467	2.0176	0.000125	0.00393	At5g03795
LOC_Os02g02120	7.4467	2.0067	-1.8918	0.000127	0.00399	WAK5
LOC_Os09g23620	0.6900	0.1400	-2.3012	0.000128	0.00401	ODO1
LOC_Os08g44220	1.0267	0.1667	-2.6229	0.000131	0.00409	At4g39670
LOC_Os09g25150	10.7967	3.8367	-1.4927	0.000132	0.00412	CCR1
LOC_Os07g43990	12.8500	5.4233	-1.2445	0.000133	0.00413	-
LOC_Os08g07080	0.2067	0.0010	-7.6912	0.000133	0.00413	TPS2
LOC_Os08g07240	0.1500	1.8867	3.6528	0.000133	0.00413	-
LOC_Os11g26920	7.7733	1.0033	-2.9537	0.000134	0.00415	RT
LOC_Os03g53360	2.7833	0.7400	-1.9112	0.000135	0.00419	CER26L

LOC_Os06g49320	28.2467	8.8567	-1.6732	0.000136	0.0042	pomgnt2
LOC_Os02g28340	3.1700	9.7600	1.6224	0.000136	0.00422	3AT1
LOC_Os10g39000	5.9600	1.5800	-1.9154	0.000137	0.00424	-
LOC_Os05g50190	25.9500	60.4633	1.2203	0.000139	0.00428	HT1
LOC_Os03g02260	2.7733	6.9667	1.3288	0.000141	0.00433	-
LOC_Os02g43700	101.0267	40.0600	-1.3345	0.000142	0.00434	At4g16820
LOC_Os08g14109	0.7933	0.0900	-3.1399	0.000142	0.00434	-
LOC_Os03g40770	13.9700	42.5333	1.6063	0.000142	0.00434	-
LOC_Os08g39860	66.0333	26.6267	-1.3103	0.000142	0.00434	BGLU27
LOC_Os01g43774	2.2367	7.7800	1.7984	0.000142	0.00435	CYP72A15
LOC_Os03g13740	2.5467	0.1600	-3.9925	0.000143	0.00437	PUB21
LOC_Os01g27360	2.7500	1.0600	-1.3754	0.000144	0.00439	GSTF1
LOC_Os05g03450	1.7733	0.6833	-1.3758	0.000144	0.00439	LECRK81
LOC_Os04g40070	0.3833	1.0867	1.5032	0.000144	0.00439	At5g50170
LOC_Os01g45840	1.7733	5.1633	1.5418	0.000144	0.00439	PCMP-E44
LOC_Os04g06520	32.9933	5.2700	-2.6463	0.000146	0.00442	-
LOC_Os03g30130	3.0700	1.4500	-1.0822	0.000146	0.00443	At5g67130
LOC_Os07g47480	3.7133	0.5433	-2.7728	0.000146	0.00443	HIPP26
LOC_Os02g20560	38.8533	1.4133	-4.7809	0.000147	0.00446	FLA11
LOC_Os10g20350	1.9533	9.2033	2.2362	0.000148	0.00446	DTXL1
LOC_Os01g15039	0.1367	0.0010	-7.0945	0.000149	0.00451	-
LOC_Os01g08110	0.7433	2.3433	1.6565	0.00015	0.00452	UGT73C3
LOC_Os08g38990	16.5567	50.5433	1.6101	0.00015	0.00452	WRKY2
LOC_Os02g03600	2.7833	6.1733	1.1492	0.00015	0.00452	DNAJB1
LOC_Os05g49320	20.5533	7.4700	-1.4602	0.00015	0.00452	RPL12-2
LOC_Os02g41800	9.3767	4.1000	-1.1935	0.000151	0.00454	ARF8
LOC_Os09g32670	30.1400	12.0933	-1.3175	0.000152	0.00456	GAE6
LOC_Os06g16960	5.6200	1.5000	-1.9056	0.000152	0.00457	OMT3
LOC_Os06g28770	0.6133	0.0667	-3.2016	0.000153	0.00457	-
LOC_Os04g31520	2.1600	0.4867	-2.1500	0.000153	0.00458	-
LOC_Os07g38470	23.8133	3.9267	-2.6004	0.000154	0.00458	-
LOC_Os02g52150	2.0600	5.3333	1.3724	0.000154	0.00458	HSP24.1
LOC_Os03g31750	8.5700	25.0533	1.5476	0.000154	0.00458	PPDK2
LOC_Os11g30360	4.3400	0.7967	-2.4456	0.000154	0.00458	-
LOC_Os05g51160	7.5133	2.1967	-1.7741	0.000154	0.00459	DIVARICATA
LOC_Os03g02710	5.7233	2.5633	-1.1588	0.000155	0.00461	HMGS
LOC_Os03g60880	1.2900	0.2100	-2.6189	0.000155	0.00461	NAT6
LOC_Os01g18050	7.1467	3.3333	-1.1003	0.000156	0.00463	TUBB1
LOC_Os06g06250	67.5933	25.8833	-1.3849	0.000157	0.00464	At5g45910
LOC_Os01g66020	8.9000	19.4000	1.1242	0.000157	0.00466	At1g11050
LOC_Os09g32470	6.6633	0.7133	-3.2236	0.000159	0.00469	At3g25290
LOC_Os04g38790	16.7167	5.2267	-1.6773	0.00016	0.00471	-
LOC_Os03g14250	30.2567	12.8767	-1.2325	0.00016	0.00472	FPP4

LOC_Os02g28310	2.2267	5.6833	1.3519	0.000161	0.00473	-
LOC_Os11g18660	0.5167	0.0700	-2.8838	0.000162	0.00477	PAZ7
LOC_Os08g40690	0.2100	2.1867	3.3803	0.000162	0.00477	RIXI
LOC_Os05g50770	0.3200	0.0033	-6.5850	0.000163	0.00479	SLAH2
LOC_Os08g29040	2.4133	0.2867	-3.0736	0.000165	0.00482	WAK3
LOC_Os11g12310	1.1933	0.1667	-2.8400	0.000165	0.00482	TY3B-I
LOC_Os10g41130	7.2800	2.3400	-1.6374	0.000165	0.00482	ERF034
LOC_Os03g25990	0.2300	0.0010	-7.8455	0.000166	0.00485	EXPA21
LOC_Os05g31290	3.9067	1.3867	-1.4943	0.000166	0.00485	MTACP2
LOC_Os03g25400	23.3900	11.1467	-1.0693	0.000167	0.00486	PDK
LOC_Os10g21130	3.7267	0.9133	-2.0287	0.000167	0.00486	-
LOC_Os02g38392	10.5433	2.3533	-2.1636	0.000167	0.00486	TAO1
LOC_Os01g51540	3.8167	1.4633	-1.3831	0.000169	0.0049	CDA1
LOC_Os05g47700	4.7033	0.4433	-3.4072	0.000169	0.00491	LTP-2
LOC_Os05g49300	6.1600	19.5200	1.6640	0.00017	0.00492	ISU1
LOC_Os06g35940	36.8900	6.3100	-2.5475	0.00017	0.00492	HD3A
LOC_Os03g28260	20.0100	5.9900	-1.7401	0.00017	0.00492	LEA5-A
LOC_Os06g38120	5.5267	1.5100	-1.8719	0.00017	0.00492	-
LOC_Os12g24650	0.0400	1.0200	4.6724	0.000172	0.00497	Os02g0794700
LOC_Os04g52320	2.1400	0.6967	-1.6191	0.000174	0.00501	QRT3
LOC_Os01g06660	3.2967	9.6100	1.5435	0.000174	0.00501	PDC1
LOC_Os04g40050	2.1733	0.6700	-1.6977	0.000175	0.00504	rnaseh2b
LOC_Os06g36090	40.7167	15.8467	-1.3614	0.000176	0.00506	ABCG42
LOC_Os03g53150	14.7133	6.4033	-1.2002	0.000178	0.00509	IAA13
LOC_Os01g41780	1.0333	0.4067	-1.3454	0.000181	0.00517	BRI1
LOC_Os06g13230	6.6567	2.4933	-1.4167	0.000183	0.00523	-
LOC_Os12g38360	7.1833	1.4167	-2.3422	0.000184	0.00525	-
LOC_Os02g08490	1.1367	2.6800	1.2374	0.000184	0.00525	CLPB3
LOC_Os07g13830	1.0367	3.4067	1.7164	0.000186	0.0053	-
LOC_Os08g44770	80.0133	24.3100	-1.7187	0.000187	0.00533	SODCP
LOC_Os03g55180	7.0600	2.8633	-1.3020	0.000191	0.00543	-
LOC_Os02g51280	1.9800	0.6467	-1.6144	0.000195	0.00553	TCP14
LOC_Os07g10600	8.9800	18.0367	1.0061	0.000196	0.00557	Smt1-1
LOC_Os02g42740	1.3700	0.4400	-1.6386	0.000199	0.00563	MSBP2
LOC_Os11g05494	17.0033	34.6533	1.0272	0.000199	0.00564	-
LOC_Os06g22330	6.2500	1.9367	-1.6903	0.000199	0.00564	-
LOC_Os12g29740	3.3533	11.0167	1.7160	0.0002	0.00566	MYOB3
LOC_Os03g58040	19.4100	9.1133	-1.0907	0.000201	0.00568	GDH1
LOC_Os04g15690	45.1633	18.5800	-1.2814	0.000201	0.00568	-
LOC_Os08g34320	0.3933	0.0633	-2.6347	0.000203	0.00571	QWRF8
LOC_Os08g43400	44.2167	14.2000	-1.6387	0.000206	0.00578	NACK1
LOC_Os08g32980	46.9933	133.0800	1.5018	0.000206	0.00578	-
LOC_Os12g28590	0.1767	0.0010	-7.4649	0.000207	0.00582	bcsl1b

LOC_Os01g12580	0.3000	1.6267	2.4389	0.000208	0.00585	LEA14-A
LOC_Os03g16980	93.5333	42.5533	-1.1362	0.00021	0.00589	UXS6
LOC_Os01g18660	2.5933	1.1700	-1.1483	0.00021	0.00589	LCD
LOC_Os04g46660	0.6800	0.1267	-2.4245	0.000211	0.00591	CYCP2-1
LOC_Os02g53850	0.3933	0.0333	-3.5607	0.000212	0.00591	SBT1.7
LOC_Os02g50630	0.4800	0.1000	-2.2630	0.000213	0.00594	E2FE
LOC_Os01g41890	0.8900	0.3033	-1.5529	0.000215	0.00598	RPP13
LOC_Os04g52450	68.8133	137.7367	1.0012	0.000217	0.00604	OSL2
LOC_Os11g05550	6.2767	2.9233	-1.1024	0.000219	0.00608	At5g22090
LOC_Os02g43290	1.6133	0.3933	-2.0362	0.00022	0.00609	At4g10390
LOC_Os02g35940	3.1467	7.8533	1.3195	0.00022	0.00609	TCEA2
LOC_Os04g52390	18.2433	43.8533	1.2653	0.000221	0.00612	HAK11
LOC_Os06g06900	7.7733	3.5867	-1.1159	0.000221	0.00612	SPT
LOC_Os04g55710	362.0000	71.6967	-2.3360	0.000222	0.00613	At3g16150
LOC_Os11g47530	0.0633	0.9333	3.8814	0.000224	0.00618	Chib3H-h
LOC_Os10g27170	0.9100	0.3100	-1.5536	0.000224	0.00619	-
LOC_Os03g15080	23.8633	8.6000	-1.4724	0.000225	0.00622	-
LOC_Os03g30950	0.4333	3.3700	2.9592	0.000227	0.00627	Os03g0423300
LOC_Os04g12720	0.1133	0.9900	3.1269	0.000228	0.00629	UGT74E1
LOC_Os05g12640	21.4567	7.5500	-1.5069	0.000229	0.0063	BURP2
LOC_Os07g08750	7.0033	2.5467	-1.4594	0.000229	0.0063	WNK3
LOC_Os08g01910	0.8833	3.7667	2.0923	0.000231	0.00633	-
LOC_Os10g02250	0.7967	0.0633	-3.6529	0.000231	0.00633	WAK2
LOC_Os12g12470	7.9833	2.3933	-1.7380	0.000233	0.00639	DBR
LOC_Os06g28780	0.6933	0.0267	-4.7004	0.000238	0.00652	-
LOC_Os01g51030	4.8100	9.9633	1.0506	0.000241	0.00657	-
LOC_Os01g26390	2.1100	5.6533	1.4219	0.000241	0.00657	CRK25
LOC_Os08g35710	6.4300	2.3867	-1.4298	0.000241	0.00658	-
LOC_Os02g10290	11.3567	24.0267	1.0811	0.000244	0.00664	HMA5
LOC_Os04g28280	1.0800	0.1767	-2.6119	0.000246	0.00669	BHLH62
LOC_Os06g41360	32.3500	69.6000	1.1053	0.000246	0.00669	Os06g0617800
LOC_Os09g37050	6.6367	2.0467	-1.6972	0.000247	0.00669	NIP1
LOC_Os04g45720	0.3767	0.0567	-2.7327	0.000247	0.00669	ALDH3F1
LOC_Os09g10600	5.0733	2.0900	-1.2794	0.000247	0.00669	Os09g0277800
LOC_Os04g37480	1.8200	0.5667	-1.6834	0.000249	0.00673	COR2
LOC_Os11g10460	1.0800	0.0400	-4.7549	0.000249	0.00673	PER43
LOC_Os01g68770	53.0567	150.7467	1.5065	0.000251	0.00676	SBP1
LOC_Os01g72910	10.6533	3.4767	-1.6155	0.000252	0.0068	ASR1
LOC_Os07g02910	3.2933	7.6500	1.2159	0.000253	0.00683	-
LOC_Os02g43970	4.5667	1.2567	-1.8615	0.000254	0.00684	ERF034
LOC_Os02g09940	138.2167	51.5467	-1.4230	0.000256	0.00688	PRXIIE-2
LOC_Os03g26910	5.7667	1.8767	-1.6196	0.000256	0.00688	TPP9
LOC_Os05g32960	7.2200	2.9667	-1.2832	0.000257	0.00689	-

LOC_Os01g12760	5.6967	11.8400	1.0555	0.00026	0.00696	CYP71A1
LOC_Os01g70190	30.1733	13.2800	-1.1840	0.00026	0.00697	Os01g0926600
LOC_Os02g43900	0.9533	0.2333	-2.0306	0.000261	0.00698	-
LOC_Os01g53260	2.3133	5.9467	1.3621	0.000262	0.00701	WRKY24
LOC_Os01g11010	0.5100	0.0600	-3.0875	0.000263	0.00701	-
LOC_Os07g30154	0.1767	1.4533	3.0403	0.000264	0.00705	-
LOC_Os10g25210	4.4367	9.7333	1.1335	0.000265	0.00705	IRL5
LOC_Os05g30970	11.8333	3.0067	-1.9766	0.000266	0.00707	BON3
LOC_Os04g40990	7.2000	34.9800	2.2805	0.000267	0.00709	MS
LOC_Os04g25560	25.4767	9.8433	-1.3720	0.000269	0.00713	CBP1
LOC_Os08g17140	0.3467	1.1400	1.7174	0.000269	0.00713	pol
LOC_Os03g10110	0.3333	0.0400	-3.0589	0.00027	0.00716	AMP2-2
LOC_Os12g41940	0.0010	0.2167	7.7593	0.000272	0.00721	-
LOC_Os11g44560	2.5067	0.9300	-1.4305	0.000274	0.00724	CCR4
LOC_Os11g02369	1655.0767	448.5433	-1.8836	0.000276	0.00727	LTP2-A
LOC_Os01g67390	1.2133	0.0733	-4.0484	0.00028	0.00738	RBM12B
LOC_Os01g48620	0.8733	0.2600	-1.7480	0.000281	0.00739	-
LOC_Os10g36848	14.6833	2.3800	-2.6251	0.000281	0.00739	CYP84A1
LOC_Os09g10274	64.8900	22.5833	-1.5227	0.000281	0.00739	-
LOC_Os03g14610	6.8167	1.6733	-2.0263	0.000282	0.0074	HVA22I
LOC_Os09g18230	2.1267	0.3867	-2.4594	0.000282	0.0074	SIRK
LOC_Os03g60509	241.4533	68.3233	-1.8213	0.000283	0.00741	CHI
LOC_Os02g46260	39.6767	14.9900	-1.4043	0.000284	0.00745	SCPL17
LOC_Os05g32740	0.9100	0.0667	-3.7708	0.000285	0.00746	-
LOC_Os07g46920	1.5433	0.1833	-3.0735	0.000288	0.00754	-
LOC_Os02g44670	0.9267	0.0600	-3.9490	0.000293	0.00764	-
LOC_Os01g08190	0.2300	0.0333	-2.7866	0.000293	0.00765	LUG
LOC_Os08g39870	17.1133	5.9933	-1.5137	0.000296	0.00771	BGLU28
LOC_Os09g39850	0.6900	3.4900	2.3386	0.000296	0.00772	ERF1B
LOC_Os03g24930	56.8267	125.1467	1.1390	0.000297	0.00772	At5g01020
LOC_Os12g04340	1.9000	0.4967	-1.9356	0.000297	0.00772	SOT16
LOC_Os08g10400	2.1633	0.7633	-1.5029	0.000298	0.00774	-
LOC_Os05g45100	1.1367	5.1833	2.1891	0.000302	0.00782	RhGT1
LOC_Os08g41730	3.1867	7.7800	1.2877	0.000305	0.00789	PBF1
LOC_Os04g58130	12.8967	6.3467	-1.0229	0.000309	0.00798	At5g23430
LOC_Os02g47840	19.9233	43.7833	1.1359	0.000311	0.00803	At3g01520
LOC_Os03g32314	113.7300	38.2867	-1.5707	0.000313	0.00807	AOC3
LOC_Os01g03940	1.7700	0.2933	-2.5931	0.000314	0.00809	-
LOC_Os01g59350	10.0200	26.9867	1.4294	0.000317	0.00816	-
LOC_Os08g07730	0.1167	1.0300	3.1422	0.000321	0.00826	-
LOC_Os06g02650	0.0333	0.3833	3.5236	0.00033	0.00846	-
LOC_Os03g63390	1.6433	0.1433	-3.5192	0.000332	0.0085	-
LOC_Os01g56810	13.6833	1.7400	-2.9753	0.000334	0.00856	CKX5

LOC_Os11g01550	3.7000	9.4500	1.3528	0.000335	0.00857	LBD12
LOC_Os02g12350	0.4433	1.3467	1.6029	0.000336	0.00859	-
LOC_Os04g59380	1.8300	0.4200	-2.1234	0.000339	0.00865	-
LOC_Os01g47410	3.4500	1.5433	-1.1605	0.000339	0.00866	Os05g0567100
LOC_Os03g26530	52.9000	22.5267	-1.2316	0.00034	0.00866	KCS12
LOC_Os12g34802	3.4533	0.8700	-1.9889	0.000341	0.00868	-
LOC_Os08g40060	7.4133	1.9667	-1.9144	0.000342	0.0087	-
LOC_Os04g53180	2.6800	0.2433	-3.4612	0.000345	0.00876	-
LOC_Os04g42620	25.8933	9.2267	-1.4887	0.000346	0.00879	LRX4
LOC_Os11g12290	1.7833	0.3400	-2.3910	0.000348	0.00881	-
LOC_Os12g03594	2.2567	0.9800	-1.2033	0.000348	0.00882	PP2B10
LOC_Os09g37344	3.9667	8.8800	1.1626	0.000352	0.00889	-
LOC_Os10g23240	1.7867	11.6367	2.7033	0.000353	0.00889	-
LOC_Os12g41710	20.4633	48.5000	1.2449	0.000353	0.00889	At1g56140
LOC_Os06g39880	0.9833	0.1333	-2.8826	0.000353	0.00889	CYP734A4
LOC_Os08g16359	2.4733	0.3533	-2.8074	0.000353	0.00889	-
LOC_Os01g03870	2.3933	6.3833	1.4153	0.000353	0.00889	-
LOC_Os08g15149	27.8200	6.9900	-1.9928	0.000355	0.00892	-
LOC_Os04g38042	14.6367	3.8267	-1.9354	0.000355	0.00892	-
LOC_Os10g23090	1.7500	8.3733	2.2584	0.000355	0.00892	HOX8
LOC_Os03g52040	15.3500	5.0500	-1.6039	0.000358	0.00898	SCPL18
LOC_Os04g51830	0.1767	0.0010	-7.4649	0.000361	0.00903	HKT7
LOC_Os01g69290	0.6867	0.0867	-2.9861	0.000361	0.00903	-
LOC_Os07g04980	0.7133	2.0733	1.5393	0.000361	0.00903	-
LOC_Os01g67240	4.8367	1.7233	-1.4888	0.000362	0.00905	FH1
LOC_Os07g33000	0.1067	0.0010	-6.7370	0.000363	0.00906	-
LOC_Os07g46870	7.0200	1.7000	-2.0459	0.000369	0.00919	SDR3b
LOC_Os11g24484	0.7533	0.1300	-2.5348	0.000369	0.00919	KCR1
MSTRG.22000	3.1233	1.1133	-1.4882	0.000371	0.00921	--
LOC_Os04g27430	1.2000	0.0800	-3.9069	0.000371	0.00921	-
LOC_Os02g58214	3.9167	0.8433	-2.2155	0.000372	0.00922	-
LOC_Os03g10680	2.6800	0.9500	-1.4962	0.000375	0.00929	FH8
LOC_Os12g27830	0.0400	0.6100	3.9307	0.000376	0.00929	HSD1
LOC_Os10g25130	30.3167	62.5400	1.0447	0.000378	0.00935	-
LOC_Os06g06760	6.4867	3.0100	-1.1077	0.000379	0.00936	PUB33
LOC_Os03g61270	0.8467	3.1633	1.9016	0.000379	0.00936	MAN3
LOC_Os02g20850	1.5900	0.3600	-2.1430	0.000383	0.00943	RAC7
LOC_Os07g13390	0.4933	0.1667	-1.5656	0.000388	0.00954	At1g48120
LOC_Os03g58300	24.1067	3.6633	-2.7182	0.000389	0.00956	BX1
LOC_Os09g22490	15.1400	7.0033	-1.1123	0.000391	0.0096	-
LOC_Os01g53370	2.2967	0.4467	-2.3623	0.000392	0.00961	RhGT1
LOC_Os05g41240	27.8533	65.0533	1.2238	0.000397	0.00972	PHL1
LOC_Os05g30350	11.2267	38.4367	1.7756	0.000398	0.00974	BGLU22

LOC_Os12g03240	6.3833	1.4867	-2.1022	0.000399	0.00975	-
LOC_Os07g07300	13.2100	5.3967	-1.2915	0.000401	0.00978	-
LOC_Os05g35440	7.2467	14.8467	1.0347	0.000401	0.00979	SHM7
LOC_Os04g33990	38.7600	13.2500	-1.5486	0.000405	0.00986	YLS9
LOC_Os07g24190	100.2000	31.0433	-1.6905	0.000408	0.00993	CESA3
LOC_Os01g61380	1.8533	7.3833	1.9941	0.000412	0.01002	At3g47520
LOC_Os07g35940	8.7567	2.4933	-1.8123	0.000416	0.0101	BMY1
LOC_Os02g18070	1.6200	0.4900	-1.7251	0.000417	0.01011	RGA2
LOC_Os10g36650	7.5800	2.9700	-1.3517	0.00042	0.01018	ACT2
LOC_Os07g36570	13.1667	2.3567	-2.4821	0.000425	0.01026	B120
LOC_Os09g38320	13.4967	4.1633	-1.6968	0.000426	0.01028	PSY
LOC_Os10g08026	1.1133	0.1533	-2.8601	0.00043	0.01037	Os03g0232800
LOC_Os03g40170	0.4400	1.2767	1.5368	0.000435	0.01047	At3g02290
MSTRG.26335	1.2700	3.2467	1.3541	0.000435	0.01047	--
LOC_Os01g67190	7.2800	2.2367	-1.7026	0.000435	0.01047	RNS2
LOC_Os02g14680	7.8400	2.9233	-1.4232	0.000439	0.01056	UGT72B1
LOC_Os03g11650	14.0400	6.8700	-1.0312	0.000442	0.01061	MAP70.4
LOC_Os01g55240	5.9233	0.7267	-3.0270	0.000442	0.01061	GA2OX1
LOC_Os07g32020	29.1500	9.2833	-1.6508	0.000443	0.01062	GT1
LOC_Os09g07140	2.4233	0.4100	-2.5633	0.000443	0.01062	-
MSTRG.22412	17.0633	5.1233	-1.7357	0.000445	0.01063	--
LOC_Os03g47810	1.3767	0.4133	-1.7358	0.000445	0.01063	NFD4
LOC_Os05g25680	10.5400	21.9267	1.0568	0.000445	0.01063	-
LOC_Os02g41450	0.4767	0.0010	-8.8968	0.000451	0.01075	NAC031
MSTRG.27573	1.6367	0.2033	-3.0088	0.000453	0.01078	--
LOC_Os10g38660	0.9100	0.0333	-4.7708	0.000455	0.01081	GSTU6
LOC_Os07g28850	5.4833	1.5233	-1.8478	0.000457	0.01085	AGO18
LOC_Os01g07420	1.5833	0.3100	-2.3526	0.000458	0.01085	Mb2734
LOC_Os01g03740	2.9933	1.1467	-1.3843	0.000461	0.01092	ENDO2
LOC_Os03g52630	65.8067	26.4233	-1.3164	0.000462	0.01093	GLU2
LOC_Os02g43670	17.2733	6.6733	-1.3721	0.000465	0.01098	CER26L
LOC_Os04g57800	1.9067	0.7933	-1.2651	0.000466	0.01101	At3g19850
LOC_Os01g43372	27.8667	58.3367	1.0659	0.000468	0.01105	WRKY19
LOC_Os09g32010	2.0333	0.9400	-1.1131	0.000469	0.01106	-
LOC_Os05g24770	0.1000	1.1167	3.4811	0.00047	0.01107	RTNLB1
LOC_Os08g38910	149.1033	37.7633	-1.9813	0.00047	0.01107	ROMT-17
LOC_Os11g05740	0.3933	0.0233	-4.0753	0.000471	0.01108	Os11g0156000
LOC_Os08g09840	10.5633	3.7200	-1.5057	0.000473	0.0111	Os03g0733400
LOC_Os03g62480	1.0133	4.0667	2.0047	0.000476	0.01117	RhGT1
LOC_Os10g32680	22.2433	56.3200	1.3403	0.000482	0.01128	-
LOC_Os05g05020	8.8733	2.1400	-2.0519	0.000482	0.01128	-
LOC_Os05g04584	5.6133	2.1400	-1.3912	0.000482	0.01128	TAX10
LOC_Os03g40210	2.6333	11.0533	2.0695	0.000486	0.01136	-

LOC_Os11g04540	0.8100	0.1433	-2.4985	0.000487	0.01138	SOT1
LOC_Os08g15710	1.3033	0.0833	-3.9672	0.000504	0.01174	-
LOC_Os09g26780	25.7333	7.0400	-1.8700	0.000506	0.01177	TIFY10A
LOC_Os03g15020	32.9033	78.0067	1.2454	0.000506	0.01177	Os03g0255100
LOC_Os02g31860	8.7933	2.9500	-1.5757	0.000508	0.0118	-
LOC_Os12g01590	24.0000	48.3967	1.0119	0.000508	0.0118	-
LOC_Os03g62670	27.3633	7.4667	-1.8737	0.000509	0.0118	HOL3
LOC_Os02g40900	6.5800	13.5433	1.0414	0.000514	0.01189	RBM42
LOC_Os04g32540	4.2200	1.1900	-1.8263	0.000522	0.01207	SCPL45
LOC_Os12g02330	255.0433	117.2767	-1.1208	0.000523	0.01207	LTP3
LOC_Os01g67870	2.2800	5.9733	1.3895	0.000527	0.01215	-
LOC_Os02g37280	4.0700	1.3900	-1.5499	0.00053	0.01221	-
LOC_Os07g22730	0.0167	0.5367	5.0090	0.000536	0.01233	ERF1B
LOC_Os05g09280	1.3933	0.3100	-2.1682	0.000541	0.01244	-
LOC_Os01g11570	7.4933	15.6967	1.0668	0.000541	0.01245	At5g45920
LOC_Os06g04070	63.1600	21.3067	-1.5677	0.000545	0.01252	ADC1
LOC_Os08g33720	35.8300	10.2867	-1.8004	0.000546	0.01253	At3g47520
LOC_Os04g10940	1.1533	0.2100	-2.4573	0.000549	0.01259	-
LOC_Os08g24750	0.5933	2.5567	2.1073	0.000551	0.01262	FT1
LOC_Os06g50830	0.5533	0.0933	-2.5677	0.000553	0.01266	FD
LOC_Os11g04350	0.4367	0.0533	-3.0334	0.000557	0.01272	CXE17
LOC_Os05g35594	12.5733	5.7300	-1.1338	0.000558	0.01273	NPF5.10
LOC_Os06g46350	2.0600	0.3700	-2.4770	0.000569	0.01297	PLP7
LOC_Os12g41680	9.7700	19.5833	1.0032	0.000575	0.01307	NAC021
LOC_Os06g49890	15.3800	5.5033	-1.4827	0.000583	0.01325	FMP32
LOC_Os06g21280	0.2733	0.0300	-3.1876	0.000585	0.01328	-
LOC_Os01g11700	0.2900	0.0100	-4.8580	0.000585	0.01328	At2g27360
LOC_Os04g48840	13.2533	5.7000	-1.2173	0.00059	0.01337	FAX4
LOC_Os07g48790	0.2333	0.0010	-7.8662	0.000592	0.01341	KINB1
LOC_Os06g36050	4.9800	1.9800	-1.3306	0.000594	0.01343	-
LOC_Os01g57340	1.6767	0.7933	-1.0796	0.000596	0.01347	RGA2
LOC_Os09g39930	1.7033	3.6767	1.1100	0.000599	0.01352	PBS1
LOC_Os02g34860	26.6333	10.3167	-1.3683	0.000607	0.01371	UVR8
LOC_Os03g53950	21.3433	10.2400	-1.0596	0.000608	0.01371	SSL3
MSTRG.4225	0.6833	2.1200	1.6334	0.00061	0.01373	--
LOC_Os06g35960	5.1800	13.3867	1.3698	0.000614	0.01382	HSFC2B
LOC_Os01g47830	0.1567	0.0010	-7.2916	0.000615	0.01383	-
LOC_Os04g45090	40.5300	18.1900	-1.1558	0.000623	0.01398	Os04g0533500
LOC_Os02g43110	1.6000	0.1933	-3.0489	0.000625	0.01401	MHX2
LOC_Os03g51350	4.1300	23.7133	2.5215	0.000627	0.01402	-
LOC_Os01g25560	12.8667	3.8233	-1.7507	0.000627	0.01402	RALFL33
LOC_Os10g21590	0.9933	5.3700	2.4346	0.000627	0.01402	PLT6
LOC_Os12g08920	4.3233	1.5967	-1.4371	0.000628	0.01402	PER43

LOC_Os01g52830	0.0333	0.5767	4.1127	0.000628	0.01402	-
LOC_Os11g10870	4.8567	1.4667	-1.7274	0.000631	0.01406	DIR19
LOC_Os08g32550	0.5900	3.1333	2.4089	0.000634	0.01413	-
LOC_Os05g39800	10.1333	4.0400	-1.3267	0.000635	0.01414	NFD4
LOC_Os02g42940	7.8267	17.7400	1.1805	0.00064	0.01425	PVA12
LOC_Os04g46110	0.7433	0.0333	-4.4790	0.000642	0.01426	-
LOC_Os09g34150	10.0267	2.6300	-1.9307	0.000642	0.01427	RPM1
LOC_Os11g42030	3.7500	1.4200	-1.4010	0.000646	0.01435	-
LOC_Os04g43200	19.3700	62.8867	1.6989	0.000657	0.01458	PXG
LOC_Os04g49980	1.4033	8.1767	2.5427	0.000658	0.01458	-
LOC_Os11g14400	1.0967	0.1300	-3.0765	0.000661	0.01464	-
LOC_Os05g18446	0.9467	2.9733	1.6512	0.000663	0.01466	-
LOC_Os01g02360	0.2267	0.8267	1.8667	0.000673	0.01488	At5g39020
LOC_Os11g04210	13.9667	6.3533	-1.1364	0.000674	0.01488	HMGCL
LOC_Os01g19170	3.5900	1.3167	-1.4471	0.000675	0.0149	At1g48100
LOC_Os01g47300	0.2100	1.1133	2.4064	0.000676	0.01491	-
LOC_Os11g17480	10.8000	3.9433	-1.4535	0.000681	0.01501	-
LOC_Os11g20080	0.5333	0.0200	-4.7370	0.000686	0.0151	OMT2
LOC_Os06g03580	42.6667	14.8033	-1.5272	0.000688	0.01512	BB
LOC_Os06g36680	0.2267	0.0300	-2.9175	0.000689	0.01513	ATH1
LOC_Os01g64520	51.4933	119.6800	1.2167	0.000694	0.01522	At2g26230
LOC_Os04g33560	0.7267	0.0933	-2.9608	0.000698	0.01529	ZPR4
LOC_Os02g54640	11.2800	3.0267	-1.8980	0.000699	0.0153	GLR2.7
MSTRG.21766	0.2200	1.0967	2.3175	0.0007	0.0153	--
LOC_Os05g10620	3.8967	8.7800	1.1720	0.0007	0.0153	NAC073
LOC_Os09g21180	14.6267	32.9100	1.1699	0.000702	0.01533	HOX25
LOC_Os02g43330	2.2833	16.4900	2.8524	0.000703	0.01534	HOX24
LOC_Os02g33944	2.3267	1.0567	-1.1387	0.000705	0.01538	-
LOC_Os01g65620	0.1667	1.1033	2.7268	0.000706	0.01539	-
LOC_Os09g31300	22.6833	54.0533	1.2528	0.000707	0.01539	BHLH130
LOC_Os08g38092	3.7033	1.6633	-1.1547	0.000708	0.01539	abhd17c
LOC_Os09g22480	7.3833	2.7733	-1.4127	0.000712	0.01547	-
LOC_Os12g02810	14.1300	5.0067	-1.4968	0.000713	0.01548	At5g01020
LOC_Os07g37230	24.7167	11.8200	-1.0643	0.000713	0.01548	SAL1
LOC_Os12g13300	2.3933	0.5967	-2.0040	0.000714	0.01549	At1g66830
LOC_Os01g48020	0.9500	0.2733	-1.7973	0.000718	0.01553	At1g34300
LOC_Os02g32690	0.0467	0.4233	3.1813	0.000718	0.01553	ABCG41
LOC_Os01g70710	58.4067	126.1700	1.1112	0.00072	0.01554	-
LOC_Os01g64020	10.6167	23.4567	1.1437	0.00072	0.01554	-
LOC_Os12g03640	0.0900	0.4333	2.2675	0.00072	0.01554	At1g34300
LOC_Os08g14190	13.6100	4.1000	-1.7310	0.000722	0.01556	SOT1
LOC_Os02g04640	14.2167	30.7500	1.1130	0.000728	0.01568	PHL1
LOC_Os04g24110	23.0167	6.9300	-1.7318	0.000734	0.01578	UGT90A1

LOC_Os01g57700	2.4767	0.6767	-1.8719	0.000734	0.01578	-
LOC_Os10g37720	2.1867	0.6867	-1.6711	0.000734	0.01578	-
LOC_Os03g60190	6.1567	2.4567	-1.3254	0.000739	0.01585	alkB
LOC_Os09g10340	3.4900	0.6700	-2.3810	0.000749	0.01605	CYP71D55
LOC_Os01g13280	1.4167	3.0900	1.1251	0.000749	0.01605	UBC26
LOC_Os12g24320	2.9633	0.9567	-1.6311	0.000753	0.01613	bcsl1b
LOC_Os03g41330	43.1467	150.1600	1.7992	0.000754	0.01613	LBD37
LOC_Os02g50320	5.0433	2.4333	-1.0514	0.000755	0.01614	MAP70.2
LOC_Os01g62610	0.5867	2.0467	1.8027	0.000759	0.01621	FKBP20-1
LOC_Os03g05370	0.0167	0.4900	4.8777	0.00076	0.01621	-
LOC_Os08g32960	0.1633	0.0010	-7.3517	0.000762	0.01625	IP5P9
LOC_Os12g43820	9.5333	3.9267	-1.2797	0.000763	0.01625	-
LOC_Os08g28790	26.6933	7.3233	-1.8659	0.000764	0.01626	DIR1
LOC_Os02g17940	7.9367	3.7433	-1.0842	0.000773	0.01642	SRG1
LOC_Os09g20540	1.6033	0.5133	-1.6431	0.000775	0.01646	-
LOC_Os03g02190	12.8600	1.9067	-2.7538	0.00078	0.01653	At1g33260
LOC_Os07g49250	0.1133	0.0010	-6.8244	0.000783	0.0166	PDC3
LOC_Os03g17850	6.7800	2.1900	-1.6304	0.000786	0.01664	Os03g0287800
LOC_Os05g29676	2.5933	8.7400	1.7528	0.000791	0.01672	ATL11
LOC_Os02g34430	2.3633	1.1567	-1.0308	0.000792	0.01673	At1g01540
LOC_Os03g02050	0.4100	2.2133	2.4325	0.000796	0.01679	LTP-2
LOC_Os04g52190	7.7600	2.1933	-1.8229	0.000797	0.0168	VSR7
LOC_Os08g01480	0.0100	0.3833	5.2605	0.000798	0.0168	CYP71C4
LOC_Os02g11770	122.7600	46.5567	-1.3988	0.000799	0.01682	-
MSTRG.10455	0.0400	0.8133	4.3458	0.0008	0.01683	--
LOC_Os10g33780	2.7233	0.8667	-1.6518	0.000801	0.01683	G1L5
LOC_Os02g55320	10.3733	21.4533	1.0483	0.000802	0.01685	ARR12
LOC_Os12g29580	3.5233	1.7067	-1.0458	0.000804	0.01686	KIPK
LOC_Os06g17490	0.4600	0.1000	-2.2016	0.000809	0.01697	LECRKS7
LOC_Os09g36320	16.5567	3.6100	-2.1973	0.000818	0.01711	At5g47070
LOC_Os08g09830	1.3433	0.5333	-1.3327	0.000818	0.01711	BPM1
LOC_Os01g21560	16.7467	6.2633	-1.4189	0.000819	0.01711	CSE
LOC_Os03g48560	1.3500	0.5100	-1.4044	0.000819	0.01711	Xylt1
LOC_Os02g52450	31.3400	13.3000	-1.2366	0.000822	0.01716	TTHA1322
LOC_Os12g04424	7.9400	3.2200	-1.3021	0.000823	0.01716	SSL10
LOC_Os07g01110	0.1267	0.0010	-6.9849	0.000824	0.01718	LAC14
LOC_Os02g14760	15.0367	32.9067	1.1299	0.000825	0.01718	CID9
LOC_Os09g21380	4.2567	1.4567	-1.5471	0.000832	0.01732	-
LOC_Os03g07170	0.2333	1.2033	2.3666	0.000835	0.01737	MFSD12
LOC_Os06g51070	22.1267	8.1767	-1.4362	0.000837	0.01738	NAC68
LOC_Os08g10130	2.5467	0.5400	-2.2376	0.000854	0.0177	-
LOC_Os01g39330	6.7533	1.4867	-2.1835	0.000858	0.01777	BHLH
LOC_Os10g38910	14.9567	3.6067	-2.0521	0.000859	0.01778	PAM68

LOC_Os06g50600	0.6600	0.1667	-1.9855	0.00086	0.0178	FD
LOC_Os04g43560	0.6367	0.0800	-2.9925	0.000864	0.01784	NAC054
LOC_Os03g06520	45.8433	15.3933	-1.5744	0.000864	0.01784	SULTR3;1
LOC_Os04g37680	0.9533	0.0467	-4.3525	0.000865	0.01785	CXE2
LOC_Os04g57180	1.6300	4.0000	1.2951	0.000869	0.01792	H6H
LOC_Os05g46480	28.4200	186.6667	2.7155	0.000876	0.01805	LEA3
LOC_Os02g53620	2.3700	1.0367	-1.1929	0.000885	0.01821	NFYA5
MSTRG.7333	0.3467	0.0133	-4.7004	0.000886	0.01823	WAK1
LOC_Os09g29520	6.1333	2.4467	-1.3259	0.000891	0.01831	WAK3
LOC_Os06g04870	3.5867	1.2467	-1.5246	0.000899	0.01846	HOX2
LOC_Os02g44320	20.9000	0.3033	-6.1065	0.000901	0.0185	-
LOC_Os01g21590	2.8267	12.3200	2.1238	0.000905	0.01855	-
LOC_Os10g10080	18.0000	8.0100	-1.1681	0.000905	0.01855	GUT1
LOC_Os08g07970	9.5567	19.9267	1.0601	0.000907	0.01857	TGA4
LOC_Os08g33160	32.6167	14.0233	-1.2178	0.000908	0.01858	TIFY6B
LOC_Os05g37600	2.5333	0.8133	-1.6391	0.000912	0.01863	GPAT6
LOC_Os07g12890	12.7800	2.7800	-2.2007	0.000913	0.01864	ZIP8
MSTRG.5533	0.8767	0.0900	-3.2840	0.000918	0.01874	--
LOC_Os10g40810	5.2733	2.0933	-1.3329	0.000928	0.01892	GATA2
LOC_Os01g04620	6.6200	2.3400	-1.5003	0.000934	0.01902	ntpR
LOC_Os05g02070	50.9533	24.4767	-1.0578	0.000935	0.01902	-
LOC_Os03g04650	52.7467	20.3933	-1.3710	0.000944	0.0192	CYP96A15
LOC_Os04g37570	0.6567	2.4167	1.8798	0.000948	0.01925	nep2
LOC_Os04g35520	53.7400	135.3667	1.3328	0.000953	0.01933	APX7
LOC_Os05g50340	18.4867	3.4600	-2.4176	0.000959	0.01942	RL6
LOC_Os01g19210	0.0867	0.0010	-6.4374	0.000962	0.01947	Os03g0733400
LOC_Os04g39010	40.2800	6.4067	-2.6524	0.000962	0.01947	-
LOC_Os01g48640	5.3167	2.2100	-1.2665	0.000964	0.01949	-
LOC_Os01g52610	0.2767	1.1833	2.0966	0.000968	0.01955	Os01g0725400
LOC_Os06g18790	19.8833	6.3067	-1.6566	0.000978	0.01971	UGT88A1
LOC_Os11g14480	0.3533	0.0010	-8.4649	0.000996	0.02003	ASB11
LOC_Os07g41050	0.8967	0.0600	-3.9015	0.001006	0.02017	VEP1
LOC_Os08g16660	0.1267	0.0010	-6.9849	0.001006	0.02017	ASPG2
LOC_Os05g27350	0.7567	4.5433	2.5860	0.001006	0.02017	-
LOC_Os04g29090	12.9100	4.8000	-1.4274	0.001015	0.02033	GULLO5
LOC_Os12g14480	1.1533	0.1133	-3.3472	0.001017	0.02035	CRK21
LOC_Os10g05980	7.0600	0.1267	-5.8006	0.001021	0.0204	PRP4
LOC_Os04g32510	3.9167	1.2633	-1.6324	0.001021	0.0204	DRIP2
LOC_Os11g42220	18.5133	0.3833	-5.5938	0.001024	0.02045	LAC20
LOC_Os05g03500	0.2400	0.9200	1.9386	0.001027	0.02049	LIMYB
LOC_Os06g35970	13.4267	0.4067	-5.0451	0.00103	0.02055	PDF1
LOC_Os03g09850	2.0733	0.9367	-1.1463	0.001035	0.0206	At2g04850
LOC_Os08g04340	3.2433	0.5500	-2.5600	0.001037	0.02064	-

LOC_Os01g37460	7.9933	1.4700	-2.4430	0.001038	0.02064	VAR3
LOC_Os04g57380	2.5467	5.2567	1.0455	0.001043	0.02071	Os04g0669600
LOC_Os10g36060	13.7033	6.3200	-1.1165	0.001044	0.02071	-
LOC_Os07g42370	485.0833	165.6433	-1.5502	0.001046	0.02075	TIFY10A
LOC_Os08g31250	0.8833	0.1767	-2.3219	0.001048	0.02077	Os03g0405500
LOC_Os06g42800	7.2967	3.2533	-1.1653	0.00105	0.02079	SRF8
LOC_Os11g10480	13.8900	41.4133	1.5760	0.00105	0.02079	ADH1
LOC_Os08g13440	0.9900	0.2133	-2.2143	0.001057	0.02091	Os08g0231400
LOC_Os07g13770	6.7733	2.9333	-1.2073	0.001063	0.021	BX9
LOC_Os01g61070	2.9100	7.8867	1.4384	0.001064	0.02101	ATX1
LOC_Os09g19380	2.4967	0.8067	-1.6300	0.001072	0.02115	At1g51890
LOC_Os02g01590	5.7333	1.6600	-1.7882	0.001072	0.02115	1-SST
LOC_Os03g45170	17.5600	8.0433	-1.1264	0.001074	0.02116	CAT2
LOC_Os01g61690	1.4700	0.1567	-3.2300	0.00108	0.02125	SCPL27
LOC_Os11g04710	9.8533	2.6133	-1.9147	0.001086	0.02135	CYP90A1
LOC_Os01g40280	1.8767	4.2533	1.1804	0.001089	0.0214	SPBC776.05
LOC_Os10g04090	4.0000	1.9400	-1.0439	0.001092	0.02145	RGA2
LOC_Os01g66740	0.2900	0.0133	-4.4429	0.001095	0.02148	At5g48380
LOC_Os09g38440	2.0867	0.8000	-1.3831	0.001101	0.02155	ATX2
LOC_Os07g29960	0.1767	0.0010	-7.4649	0.001101	0.02155	CYP87A3
LOC_Os04g48460	4.4667	10.1300	1.1814	0.001102	0.02155	CYP704C1
LOC_Os11g34720	0.7633	0.0300	-4.6693	0.001102	0.02155	PAP4
LOC_Os04g32010	1.5133	0.5333	-1.5046	0.001106	0.02162	ALS2
LOC_Os01g74490	2.2300	9.6367	2.1115	0.001107	0.02162	ATX1
LOC_Os07g47450	14.9167	3.2433	-2.2014	0.001112	0.0217	Os07g0671000
LOC_Os07g33915	4.6900	11.9233	1.3461	0.001113	0.02171	RPL36AA
LOC_Os10g33790	2.8933	0.8500	-1.7672	0.001121	0.02185	-
LOC_Os02g46210	1.7533	0.6033	-1.5391	0.001122	0.02186	-
LOC_Os04g34580	0.7867	0.0933	-3.0753	0.001124	0.02188	hpxO
LOC_Os06g03800	53.1567	16.0433	-1.7283	0.001134	0.02206	XBOS36
LOC_Os11g06900	3.5967	1.5967	-1.1716	0.001136	0.02206	FAAH
LOC_Os03g53350	0.8933	0.1033	-3.1119	0.001142	0.02218	TOGT1
LOC_Os03g06700	0.2200	0.0010	-7.7814	0.001158	0.02245	KCS11
MSTRG.1090	2.9333	0.9767	-1.5866	0.00116	0.02248	--
MSTRG.21425	1.0767	0.0567	-4.2479	0.001168	0.02259	--
LOC_Os03g48310	2.6067	5.8833	1.1744	0.001169	0.02259	LHA1
LOC_Os12g11420	2.4367	0.1433	-4.0875	0.00117	0.02259	-
LOC_Os04g25650	1.8033	0.6700	-1.4284	0.00117	0.02259	CRRSP38
LOC_Os02g57770	0.2633	0.0010	-8.0407	0.001184	0.02285	XTH32
LOC_Os07g31500	6.6800	3.1000	-1.1076	0.001191	0.02297	GSO1
LOC_Os07g32340	2.7800	1.2433	-1.1609	0.001215	0.02339	FPP3
LOC_Os04g31110	3.4033	1.4833	-1.1981	0.001216	0.02339	Y-2
LOC_Os04g35620	2.9100	6.1300	1.0749	0.001222	0.0235	-

LOC_Os09g31230	3.6867	0.9700	-1.9263	0.001233	0.02368	-
LOC_Os04g48130	35.0933	9.6433	-1.8636	0.00124	0.02379	RBL6
LOC_Os04g55620	2.8367	1.2233	-1.2134	0.001242	0.02382	At5g67200
LOC_Os04g55920	188.6633	85.9000	-1.1351	0.001244	0.02383	TIFY3B
LOC_Os03g62240	50.9300	108.7867	1.0949	0.001246	0.02386	-
LOC_Os10g38600	3.1167	11.4567	1.8781	0.001248	0.02388	GSTU6
LOC_Os09g31025	0.5200	1.8933	1.8643	0.001254	0.02399	-
LOC_Os07g01600	24.9267	6.9767	-1.8371	0.001264	0.02412	DIR1
LOC_Os04g15630	1.8633	0.3633	-2.3585	0.001265	0.02412	At3g47570
LOC_Os03g51080	118.3700	45.0567	-1.3935	0.001271	0.02419	GAD1
LOC_Os07g38450	0.5867	0.1233	-2.2500	0.001281	0.02437	AtMg00310
LOC_Os05g49950	0.3033	1.4333	2.2404	0.001282	0.02437	-
LOC_Os01g45860	2.6800	1.0900	-1.2979	0.001295	0.02457	GAI
LOC_Os05g30940	0.4333	0.0133	-5.0224	0.001297	0.02459	-
LOC_Os11g26780	5.9867	36.9533	2.6259	0.001301	0.02464	RAB16B
LOC_Os12g41140	4.5200	1.3200	-1.7758	0.001302	0.02464	VAB
LOC_Os03g18370	19.2533	8.2833	-1.2168	0.00131	0.02478	MRH1
LOC_Os04g33980	0.8500	0.3667	-1.2130	0.001318	0.02487	-
LOC_Os06g17440	10.6333	4.4300	-1.2632	0.001318	0.02487	-
LOC_Os11g09180	5.3533	2.6300	-1.0254	0.00132	0.02489	-
LOC_Os01g46400	7.4967	3.1000	-1.2740	0.001322	0.0249	TBL19
LOC_Os01g55500	3.5733	1.0700	-1.7397	0.001325	0.02495	NAT2
LOC_Os12g01550	5.2900	12.9333	1.2898	0.001326	0.02495	LBD12
LOC_Os01g66200	35.3167	15.2667	-1.2100	0.00133	0.02498	-
LOC_Os06g10000	4.2667	10.8733	1.3496	0.001338	0.02511	-
LOC_Os12g37600	2.0700	0.9133	-1.1804	0.00135	0.02528	GPAT3
LOC_Os11g07911	1.7667	14.2333	3.0102	0.00135	0.02528	-
LOC_Os02g14160	2.4433	7.7067	1.6573	0.001354	0.02535	PER52
LOC_Os03g55350	10.2467	4.9300	-1.0555	0.001362	0.02547	SBT1.7
LOC_Os03g28170	30.3133	6.8467	-2.1465	0.001363	0.02547	-
LOC_Os03g61150	34.6833	103.0033	1.5704	0.001372	0.0256	-
LOC_Os12g06335	0.9267	0.1167	-2.9897	0.001378	0.02568	LHW
LOC_Os02g56820	0.3467	1.1167	1.6876	0.001385	0.0258	At2g02240
LOC_Os04g35690	0.4933	0.0933	-2.4021	0.001401	0.02608	OEP61
LOC_Os03g52070	22.2900	6.2767	-1.8283	0.001412	0.02628	SCPL18
LOC_Os04g36800	16.0833	7.6900	-1.0645	0.001429	0.02658	KAS12
LOC_Os06g49210	3.8900	1.4833	-1.3909	0.001434	0.02665	NPF8.3
LOC_Os01g68960	0.2933	1.1400	1.9584	0.001435	0.02665	-
LOC_Os01g56235	1.3767	0.1500	-3.1981	0.001438	0.02668	At4g15545
LOC_Os03g05334	0.8933	0.1833	-2.2847	0.001442	0.02675	-
LOC_Os03g14440	0.3433	0.0010	-8.4235	0.001447	0.02678	-
LOC_Os02g03294	5.4400	1.6333	-1.7358	0.001447	0.02678	CYCP4-1
LOC_Os01g52980	4.2900	8.7067	1.0211	0.001455	0.02689	At3g58900

LOC_Os10g26940	7.0467	24.5933	1.8033	0.001458	0.02693	BURP16
LOC_Os05g44070	2.4333	0.8300	-1.5518	0.001463	0.027	RIC2
LOC_Os01g41160	0.2100	0.0010	-7.7142	0.001476	0.02718	FOXRED1
LOC_Os03g45850	0.5933	0.0010	-9.2127	0.001476	0.02718	SAUR72
LOC_Os10g36100	1.1333	0.1200	-3.2395	0.001476	0.02718	LTP-2
LOC_Os01g37920	14.2667	6.1600	-1.2116	0.001479	0.02721	-
LOC_Os12g29500	0.9967	0.1633	-2.6093	0.001486	0.02733	DRIP1
LOC_Os12g05040	0.9967	0.2667	-1.9021	0.001495	0.02747	HIPP26
LOC_Os07g38440	27.7400	13.1333	-1.0787	0.001499	0.02752	-
LOC_Os04g38885	4.4633	1.7000	-1.3926	0.001499	0.02752	-
LOC_Os03g29920	21.9767	8.5300	-1.3654	0.001509	0.02769	-
LOC_Os04g37430	37.1667	11.2867	-1.7194	0.001511	0.0277	Os03g0179900
LOC_Os04g01320	6.1333	3.0600	-1.0031	0.001532	0.02805	At2g19130
LOC_Os01g48940	13.8133	52.0667	1.9143	0.001533	0.02806	-
LOC_Os01g24070	53.4667	18.7100	-1.5148	0.001552	0.02838	GATA23
LOC_Os08g06310	0.0767	0.0010	-6.2605	0.001553	0.02838	Tf2-6
LOC_Os03g36960	14.6967	6.2767	-1.2274	0.001556	0.02841	-
LOC_Os11g05050	125.0500	33.8133	-1.8868	0.001556	0.02841	TSJT1
LOC_Os11g47330	25.4767	11.6733	-1.1260	0.00156	0.02846	ACLA-2
LOC_Os01g72470	1.4167	0.2200	-2.6869	0.001565	0.02853	-
LOC_Os01g08840	0.4200	0.0010	-8.7142	0.001566	0.02853	-
LOC_Os02g46830	18.7367	6.7367	-1.4758	0.001571	0.02862	-
LOC_Os08g06210	14.5133	2.0133	-2.8497	0.001576	0.02868	-
LOC_Os01g49710	2.0033	4.8500	1.2756	0.001578	0.02868	GSTU6
LOC_Os07g13800	0.4700	1.1933	1.3443	0.001578	0.02868	UGT76C2
LOC_Os05g39560	1.8767	0.6233	-1.5901	0.001583	0.02875	ZIP5
MSTRG.19461	0.1833	0.0010	-7.5183	0.001589	0.02882	WRKY55
LOC_Os01g19480	0.3633	0.0467	-2.9608	0.001593	0.02889	RABEPK
LOC_Os03g01530	7.7900	3.3300	-1.2261	0.001595	0.02889	TUBB2
LOC_Os04g32480	10.5500	1.5000	-2.8142	0.001596	0.02889	TIFY9
LOC_Os03g57450	7.6067	2.6533	-1.5195	0.001604	0.02902	CPK1
LOC_Os08g37470	0.0010	0.2233	7.8031	0.001619	0.02926	PCO1
LOC_Os09g32290	4.5733	12.5100	1.4518	0.001621	0.02927	At2g24580
LOC_Os09g36070	2.8333	6.7133	1.2445	0.001624	0.02931	CYP71A9
LOC_Os07g04970	0.0167	0.3800	4.5110	0.001636	0.02947	At3g50280
LOC_Os03g45519	0.0567	0.0010	-5.8244	0.001638	0.02949	CYP87A3
LOC_Os05g11750	0.2533	0.0433	-2.5475	0.001645	0.0296	PBS1
LOC_Os12g05220	0.0010	0.2633	8.0407	0.001651	0.02968	FH14
LOC_Os07g34950	16.1500	8.0433	-1.0057	0.001653	0.0297	At5g52970
LOC_Os08g19680	0.4333	0.1167	-1.8931	0.001657	0.02975	-
LOC_Os09g07440	11.0500	2.8133	-1.9737	0.00166	0.02979	At3g49630
LOC_Os07g38000	14.7167	6.0000	-1.2944	0.001667	0.0299	petJ
LOC_Os09g39650	3.4200	1.3300	-1.3626	0.001677	0.03002	PUB33

LOC_Os11g06650	10.4500	4.6267	-1.1755	0.001677	0.03002	MED26C
LOC_Os11g02530	1.2100	0.1033	-3.5496	0.001678	0.03002	WRKY70
LOC_Os01g12210	2.0200	0.2500	-3.0144	0.001678	0.03002	ALMT9
LOC_Os03g03810	1.3533	0.1367	-3.3078	0.001688	0.03016	-
LOC_Os03g12390	86.0000	42.4500	-1.0186	0.00169	0.03018	MKK7
LOC_Os03g64130	0.9533	0.3200	-1.5749	0.001706	0.03044	-
LOC_Os08g44840	1.7767	0.4633	-1.9391	0.001708	0.03046	DCR
LOC_Os03g49126	0.9033	1.8267	1.0159	0.001714	0.03054	-
LOC_Os12g11680	0.6200	0.1900	-1.7063	0.001716	0.03056	RLP12
LOC_Os02g51110	23.8867	11.8900	-1.0065	0.001718	0.03058	NIP2-1
LOC_Os02g15860	6.5900	15.0800	1.1943	0.001719	0.03058	-
LOC_Os09g27060	0.3967	0.1233	-1.6854	0.001723	0.03062	DDM1
LOC_Os03g58020	3.2033	9.2333	1.5273	0.001743	0.03095	p20
LOC_Os09g15370	10.1133	22.4800	1.1524	0.001744	0.03095	At2g01680
LOC_Os11g32890	0.3300	3.9167	3.5691	0.00175	0.03099	-
LOC_Os01g57880	1.7367	0.3800	-2.1922	0.001751	0.03099	At4g27520
LOC_Os02g13870	16.2067	4.0000	-2.0185	0.001753	0.031	NIP1-1
LOC_Os02g10920	67.5900	26.5100	-1.3503	0.001754	0.031	-
LOC_Os03g59210	84.0300	19.2800	-2.1238	0.00176	0.03109	-
LOC_Os10g40450	0.1833	1.5367	3.0673	0.001766	0.03116	-
LOC_Os03g16290	2.8000	0.7367	-1.9263	0.001772	0.03124	-
LOC_Os01g02010	0.9900	0.2700	-1.8745	0.001775	0.03128	-
LOC_Os02g56920	24.5400	1.0800	-4.5060	0.001799	0.03164	CER1
LOC_Os03g57410	0.3900	0.0467	-3.0630	0.001799	0.03164	ATL70
LOC_Os03g38710	0.1233	0.0010	-6.9464	0.001806	0.03173	PERK1
LOC_Os10g22600	0.7967	0.2233	-1.8348	0.001806	0.03173	RAP2-13
LOC_Os05g25770	159.1533	34.8567	-2.1909	0.001822	0.03194	WRKY70
LOC_Os04g33950	2.5333	0.8500	-1.5755	0.001822	0.03194	E2FB
LOC_Os06g17560	0.6400	0.1000	-2.6781	0.001825	0.03194	-
LOC_Os05g34980	9.0367	3.4033	-1.4088	0.00183	0.03201	AAP2
LOC_Os02g51680	0.5600	0.1167	-2.2630	0.001832	0.03202	TPP4
LOC_Os01g09190	0.2300	0.0010	-7.8455	0.001839	0.03211	-
LOC_Os05g19670	0.6067	0.1067	-2.5078	0.00185	0.03228	Os05g0277500
LOC_Os06g07100	2.5533	0.9400	-1.4416	0.001867	0.03256	ATL46
LOC_Os10g41550	57.7267	153.0167	1.4064	0.001875	0.03267	BAM3
LOC_Os09g09490	1.1600	0.4233	-1.4543	0.001878	0.03269	RPM1
LOC_Os10g33240	0.8033	1.6900	1.0730	0.001881	0.03269	-
LOC_Os03g13460	27.1433	13.4867	-1.0091	0.001881	0.03269	MAP65-7
LOC_Os01g47570	8.2100	22.2367	1.4375	0.001883	0.03271	-
LOC_Os11g44350	0.2933	0.0067	-5.4594	0.001886	0.03273	CBP60F
LOC_Os11g35240	0.8067	2.0867	1.3712	0.001887	0.03273	WAK5
MSTRG.17146	3.3800	9.0967	1.4283	0.001888	0.03274	--
LOC_Os03g49750	0.3233	0.0933	-1.7926	0.001891	0.03276	mph1

LOC_Os09g35600	3.7767	0.6500	-2.5386	0.001897	0.03284	DTXL4
LOC_Os08g17160	4.3200	0.4667	-3.2106	0.0019	0.03285	At5g25090
LOC_Os05g34830	8.9367	21.1567	1.2433	0.00191	0.03297	NAC48
LOC_Os09g18594	45.7567	19.0600	-1.2634	0.001912	0.03299	SIRK
LOC_Os04g55680	1.2967	0.5033	-1.3652	0.001935	0.03333	UGT89B1
LOC_Os02g30380	0.4300	0.0067	-6.0112	0.001946	0.03346	IDD14
LOC_Os05g28690	0.1633	0.0300	-2.4448	0.001946	0.03346	Fam91a1
LOC_Os11g26570	4.5433	23.7933	2.3887	0.001957	0.03358	DHN3
LOC_Os01g22230	2.7233	1.1500	-1.2437	0.001958	0.03358	PER1
LOC_Os03g02840	6.9667	2.4900	-1.4843	0.001959	0.03359	-
LOC_Os08g28570	0.3400	0.1133	-1.5850	0.001962	0.0336	RPP13
LOC_Os08g42080	0.2933	0.0233	-3.6521	0.001963	0.0336	ACR5
LOC_Os12g37690	1.6967	5.0800	1.5821	0.001964	0.0336	MYB108
LOC_Os09g25610	1.2433	0.1500	-3.0512	0.001965	0.0336	guaAA
LOC_Os01g05220	3.4100	1.5067	-1.1784	0.001967	0.03362	-
MSTRG.23921	0.1967	0.8700	2.1453	0.001969	0.03363	--
LOC_Os12g38370	0.6400	0.0633	-3.3370	0.001995	0.03403	-
LOC_Os05g07420	3.5500	0.7767	-2.1925	0.001996	0.03403	At2g19130
LOC_Os01g09770	29.5600	10.0900	-1.5507	0.002032	0.03456	-
LOC_Os07g33650	7.3500	3.0900	-1.2501	0.00204	0.03466	-
LOC_Os05g05860	0.0367	0.3233	3.1405	0.002045	0.03472	NRPB1
LOC_Os02g13980	3.5567	0.9000	-1.9825	0.002054	0.03483	-
LOC_Os01g52250	156.8300	39.2433	-1.9987	0.002058	0.03487	SS4
LOC_Os12g42430	13.0533	5.7167	-1.1912	0.002067	0.03497	IQD1
LOC_Os07g07230	7.6367	2.9567	-1.3690	0.002068	0.03497	At3g03770
LOC_Os06g31090	0.1667	1.3133	2.9782	0.002073	0.03503	POB1
LOC_Os02g56340	6.7600	2.7033	-1.3223	0.002075	0.03504	-
LOC_Os04g08390	0.2833	0.1000	-1.5025	0.002084	0.03517	-
LOC_Os12g42910	0.3533	1.2433	1.8151	0.002092	0.03529	CCX1
LOC_Os11g29410	0.1867	1.2000	2.6845	0.002096	0.03532	-
LOC_Os05g50260	0.1000	0.4433	2.1484	0.002104	0.03544	At1g48100
LOC_Os06g43170	6.5133	2.4900	-1.3873	0.00211	0.03551	At4g34220
LOC_Os10g07998	0.0333	0.3500	3.3923	0.002117	0.03559	At1g09380
LOC_Os02g30190	9.3000	1.4100	-2.7215	0.002121	0.03563	RIN4
LOC_Os03g35940	0.0010	0.1133	6.8244	0.002128	0.0357	-
LOC_Os03g45450	10.1867	2.7967	-1.8649	0.002134	0.03577	WRKY71
LOC_Os12g38760	2.3600	0.7267	-1.6994	0.002136	0.03579	PAP1
LOC_Os07g34520	3.8867	24.3267	2.6459	0.002144	0.03586	ICL
LOC_Os07g25460	27.6000	67.6667	1.2938	0.002156	0.03605	Ankrd13b
LOC_Os09g18159	0.1267	0.0067	-4.2479	0.002171	0.03627	At1g51820
LOC_Os01g43580	5.1067	2.3133	-1.1424	0.002178	0.03637	KINESIN-13A
LOC_Os04g40470	2.1300	5.7467	1.4319	0.002189	0.0365	CYP71A9
LOC_Os07g10910	3.4267	8.1500	1.2500	0.002193	0.03652	EXO70A1

LOC_Os11g37700	0.1833	1.0100	2.4618	0.002194	0.03652	ABCG48
LOC_Os04g09770	0.3367	0.0010	-8.3952	0.002197	0.03656	At5g39020
LOC_Os05g49370	74.7767	31.2533	-1.2586	0.002204	0.03663	-
LOC_Os02g46100	0.7833	0.1233	-2.6671	0.002221	0.03689	ATL80
LOC_Os07g34240	0.5167	0.0067	-6.2761	0.002223	0.0369	HIDM
LOC_Os02g02930	3.9167	0.0833	-5.5546	0.002225	0.0369	-
LOC_Os08g32160	19.7033	6.0967	-1.6923	0.002226	0.03691	AOP1.2
LOC_Os08g37990	0.7733	0.2167	-1.8356	0.002232	0.03698	-
LOC_Os03g02230	0.1700	1.2167	2.8393	0.002244	0.03716	-
LOC_Os03g49430	0.5833	1.7467	1.5822	0.002248	0.0372	prpf18
LOC_Os04g58020	25.4733	56.5633	1.1509	0.002253	0.03727	DIVARICATA
LOC_Os10g40660	0.1200	0.0010	-6.9069	0.002269	0.03749	ZAT9
LOC_Os02g46560	1.2033	0.4033	-1.5770	0.002273	0.03751	BHLH96
LOC_Os01g60780	3.0367	1.1833	-1.3596	0.002273	0.03751	At1g06890
LOC_Os03g18070	68.5800	17.3933	-1.9793	0.002275	0.03751	FAD7A-1
LOC_Os07g35510	88.1167	190.4433	1.1119	0.002282	0.03761	At4g29360
LOC_Os01g53640	3.1467	1.4167	-1.1513	0.002284	0.03762	PERK9
LOC_Os03g17800	3.1600	6.5733	1.0567	0.002295	0.03777	rdgBbeta
LOC_Os10g35050	0.1333	0.5933	2.1538	0.002307	0.03792	TIP3-1
LOC_Os03g14654	963.8267	466.6433	-1.0465	0.002307	0.03792	-
LOC_Os03g05820	0.6900	1.4333	1.0547	0.002312	0.03798	Os03g0152900
LOC_Os04g46940	1.4200	0.5667	-1.3253	0.002314	0.03799	HMA5
LOC_Os05g43240	12.3867	2.8533	-2.1181	0.002318	0.03803	JAL3
LOC_Os07g35520	0.1033	0.0010	-6.6912	0.002327	0.03814	At5g56590
LOC_Os01g43480	20.1467	8.7967	-1.1955	0.002328	0.03814	atad1a
LOC_Os11g04960	8.4100	3.9867	-1.0769	0.002329	0.03814	NAC029
LOC_Os02g46030	3.2067	8.5400	1.4132	0.002337	0.03825	RVE1
LOC_Os01g10930	1.0067	0.4833	-1.0585	0.002347	0.03839	-
LOC_Os05g33690	2.1800	0.9267	-1.2342	0.002356	0.03849	ERL1
MSTRG.826	0.7067	1.8333	1.3754	0.002374	0.03873	--
LOC_Os08g01670	2.5567	0.6800	-1.9107	0.002377	0.03875	C/VIF2
LOC_Os03g61620	8.5267	2.9433	-1.5345	0.002395	0.03897	-
LOC_Os10g02340	1.4433	0.5033	-1.5198	0.002397	0.03897	NPF8.3
LOC_Os01g64120	215.2700	497.2633	1.2079	0.002398	0.03897	FDX6
LOC_Os11g42800	9.8467	4.1800	-1.2361	0.00241	0.03915	KP1
LOC_Os06g46149	54.0333	20.1400	-1.4238	0.002413	0.03918	-
LOC_Os11g40780	0.3333	0.0900	-1.8890	0.002424	0.03931	RGA2
LOC_Os03g29930	11.4300	4.9067	-1.2200	0.002429	0.03936	-
LOC_Os09g25810	0.8367	0.2000	-2.0647	0.002442	0.03954	At4g30420
LOC_Os02g51730	1.0100	0.0067	-7.2432	0.002449	0.03962	TTL3
LOC_Os03g08790	1.0367	0.2100	-2.3035	0.00245	0.03962	nep1
LOC_Os03g54820	0.4733	0.0010	-8.8867	0.002461	0.03975	-
LOC_Os10g26540	24.6667	57.7900	1.2283	0.002462	0.03975	-

LOC_Os12g36810	0.6367	1.4600	1.1974	0.002469	0.03984	GSVIVT00026920001
LOC_Os11g35080	2.5000	1.1300	-1.1456	0.002477	0.03992	-
LOC_Os03g06010	0.0010	0.2833	8.1464	0.002483	0.03997	EXPA25
LOC_Os09g34320	24.0967	8.3967	-1.5209	0.002484	0.03997	-
LOC_Os04g49194	2.7133	0.5300	-2.3560	0.002486	0.03997	-
LOC_Os08g37340	0.7667	3.7033	2.2722	0.00249	0.03999	-
LOC_Os05g05930	0.0967	1.0567	3.4504	0.002503	0.04016	TSPO
LOC_Os09g29120	0.3433	0.0010	-8.4235	0.002509	0.04019	GEM
LOC_Os01g19750	0.9167	0.2267	-2.0158	0.002511	0.04019	SE2
LOC_Os01g25360	1.4333	0.5400	-1.4083	0.002521	0.04031	MES7
LOC_Os06g28480	3.8133	1.3633	-1.4839	0.002522	0.04031	DRT100
LOC_Os04g52920	0.0900	0.0010	-6.4919	0.002523	0.04031	-
LOC_Os03g51459	2.1133	5.0900	1.2681	0.002541	0.04057	-
LOC_Os09g20980	3.6400	0.9633	-1.9178	0.002548	0.04064	ATL46
LOC_Os01g28970	0.3367	3.8167	3.5029	0.002557	0.04077	-
LOC_Os04g03920	9.1067	1.3467	-2.7575	0.002564	0.04086	-
LOC_Os09g38772	5.7133	11.8700	1.0549	0.002576	0.04102	2-Mar
LOC_Os12g02400	7.3300	1.8233	-2.0072	0.002593	0.04125	WRKY70
LOC_Os08g35860	6.1567	14.9300	1.2780	0.002599	0.0413	CKX11
LOC_Os02g33380	0.7033	0.0900	-2.9662	0.0026	0.0413	-
LOC_Os04g50930	2.4967	5.2300	1.0668	0.002612	0.04146	NPF8.3
LOC_Os03g10210	1.0800	4.1033	1.9258	0.002626	0.04163	HOX12
LOC_Os01g29280	2.8767	0.7167	-2.0050	0.002628	0.04164	-
LOC_Os08g26850	13.1933	4.8267	-1.4507	0.002636	0.04175	-
LOC_Os07g45530	0.1867	0.0633	-1.5594	0.002645	0.04186	-
LOC_Os01g07620	0.4367	1.6467	1.9149	0.002647	0.04187	-
LOC_Os07g01710	1.2067	2.5433	1.0757	0.002654	0.04196	PSKR2
LOC_Os06g47620	6.6600	1.5067	-2.1442	0.002662	0.04206	ILL6
LOC_Os07g08160	0.0767	0.8600	3.4877	0.002666	0.04211	-
LOC_Os05g15530	4.6333	14.2400	1.6198	0.002668	0.04211	DAAT
LOC_Os06g50480	0.7267	0.0500	-3.8613	0.002671	0.04214	TRAB1
LOC_Os12g14520	2.0800	0.3733	-2.4780	0.002674	0.04216	-
LOC_Os01g37040	20.7067	8.9267	-1.2139	0.002701	0.04256	-
LOC_Os04g38910	0.8567	0.2867	-1.5794	0.002705	0.04257	RKL1
LOC_Os05g27304	11.8900	4.9433	-1.2662	0.002709	0.0426	NPF2.3
LOC_Os09g02270	2.0000	5.0833	1.3458	0.002715	0.04266	kynB
LOC_Os03g12730	1.1767	0.4267	-1.4635	0.002722	0.04274	BAM1
LOC_Os03g60370	4.6533	10.5067	1.1750	0.002729	0.04282	mipp1
LOC_Os04g47840	0.7133	0.0433	-4.0410	0.002731	0.04283	-
LOC_Os12g13910	4.4300	9.6200	1.1187	0.002737	0.04288	-
LOC_Os03g59320	20.6400	43.3033	1.0690	0.002739	0.04288	-
LOC_Os03g11910	1.2200	2.7833	1.1899	0.002739	0.04288	HSP70-8
LOC_Os07g01530	0.8033	0.2500	-1.6841	0.002753	0.04306	At5g45440

LOC_Os12g41730	0.0900	0.0033	-4.7549	0.002755	0.04307	-
LOC_Os05g03130	0.2000	1.3700	2.7761	0.002761	0.04314	LT101.2
LOC_Os01g64860	4.4467	0.1200	-5.2116	0.002764	0.04317	SBT1.7
LOC_Os02g42160	5.2067	1.8200	-1.5164	0.002778	0.04334	WAK5
LOC_Os08g44360	0.5600	1.4200	1.3424	0.002783	0.04339	FAR1
LOC_Os08g14990	0.6667	0.3133	-1.0893	0.002786	0.04341	At3g47570
LOC_Os06g03900	1.9067	0.6667	-1.5160	0.002789	0.04342	At1g28600
LOC_Os01g48610	4.5967	1.7533	-1.3905	0.002793	0.04345	-
LOC_Os05g39200	0.0010	0.1767	7.4649	0.002803	0.04359	-
LOC_Os11g40570	2.5333	7.6167	1.5881	0.00282	0.04381	tmem45b
LOC_Os08g07490	8.0167	3.0733	-1.3832	0.002838	0.044	-
MSTRG.8403	1.1400	2.6800	1.2332	0.002841	0.04403	--
LOC_Os03g05610	0.5033	0.0933	-2.4310	0.002846	0.04408	PHT1-12
LOC_Os01g46950	0.6367	0.2467	-1.3680	0.002876	0.04452	YMR099C
LOC_Os01g37590	49.9600	12.3400	-2.0174	0.002923	0.04516	NPF6.2
ChrSy.fgenesh.gen	1.8600	0.5433	-1.7754	0.002925	0.04516	WAK3
LOC_Os01g42860	9.3867	41.3167	2.1380	0.002936	0.04531	-
LOC_Os03g58490	0.3533	0.0967	-1.8699	0.002945	0.04539	MYOB2
LOC_Os01g04350	279.5533	127.9033	-1.1281	0.002945	0.04539	HSP17.9B
LOC_Os09g32020	3.8500	8.1667	1.0849	0.002956	0.04554	TKPR1
LOC_Os01g53990	0.2533	0.0010	-7.9849	0.002967	0.04568	PME53
LOC_Os02g46473	30.8300	64.6767	1.0689	0.002973	0.04574	-
LOC_Os05g04460	0.0933	0.0010	-6.5443	0.002974	0.04574	WAK5
LOC_Os04g47810	2.0333	0.7933	-1.3578	0.002991	0.04598	BHLH111
LOC_Os04g56390	0.7333	0.0600	-3.6114	0.003027	0.04645	-
LOC_Os01g50010	13.7800	6.7467	-1.0303	0.003034	0.04653	At4g32285
LOC_Os12g08850	0.5700	0.0533	-3.4179	0.003035	0.04653	csd
LOC_Os05g02564	0.8033	0.3500	-1.1986	0.003037	0.04654	-
LOC_Os10g30880	13.6467	5.9367	-1.2008	0.003059	0.04683	-
LOC_Os01g09700	5.4133	1.1833	-2.1937	0.00306	0.04683	ACS7
LOC_Os01g12190	6.7300	3.0867	-1.1246	0.003062	0.04685	-
LOC_Os11g02470	1.6333	0.3333	-2.2928	0.003068	0.04691	WRKY54
LOC_Os03g53220	0.0067	0.1500	4.4919	0.003072	0.04695	Snrnp200
LOC_Os07g03820	0.3533	0.9833	1.4767	0.003082	0.04702	LECRK42
LOC_Os04g43670	1.4433	0.3233	-2.1583	0.003108	0.04739	-
LOC_Os10g37500	8.0200	3.5767	-1.1650	0.003129	0.04769	SPAC644.07
LOC_Os06g12560	4.0333	1.8100	-1.1560	0.003151	0.04792	ATL24
LOC_Os10g37290	7.6767	3.6067	-1.0898	0.003151	0.04792	-
LOC_Os11g40410	0.5633	0.0733	-2.9414	0.003155	0.04796	-
LOC_Os01g52260	16.9967	34.1567	1.0069	0.00316	0.048	SAT1
LOC_Os04g10924	2.0833	0.7567	-1.4612	0.003175	0.04817	-
LOC_Os04g30330	0.5333	0.0433	-3.6215	0.003192	0.04839	WAKL2
LOC_Os07g18158	1.7033	0.6967	-1.2898	0.003213	0.04863	AO1

LOC_Os08g34460	0.3700	0.0433	-3.0940	0.003236	0.04889	TET8
LOC_Os02g16050	0.0500	0.2067	2.0473	0.00326	0.04919	At1g48120
LOC_Os06g09900	540.3767	1116.0700	1.0464	0.00326	0.04919	-
LOC_Os03g28990	1.1367	0.1333	-3.0917	0.003294	0.04961	sll0103
LOC_Os07g13834	6.4000	13.8400	1.1127	0.003302	0.04971	-
LOC_Os12g02640	6.2733	2.8367	-1.1450	0.003312	0.04983	CYP714C2
LOC_Os07g47700	99.3667	43.8733	-1.1794	0.003323	0.04994	UXS2
LOC_Os01g68470	0.3833	0.0400	-3.2605	0.003327	0.04997	-

Table S4. Statistical data of total differentially expressed genes (DEGs) for SN_CK vs. SN_A.

id	SN_CK _mean	SN_A _mean	log2(fc)	PValue	FDR	Symbol
LOC_Os07g03730	471.4467	19.2633	-4.6132	1.13E-15	3.14E-11	-
LOC_Os01g68740	84.6033	8.1967	-3.3676	3.94E-15	5.46E-11	-
LOC_Os08g37300	2.9333	15.2633	2.3795	8.95E-13	8.27E-09	-
LOC_Os02g02930	8.4000	0.1267	-6.0513	1.37E-12	9.53E-09	-
LOC_Os04g29770	15.8367	1.7500	-3.1778	4.51E-11	2.24E-07	WAK3
LOC_Os01g09150	3.9767	0.1033	-5.2662	4.86E-11	2.24E-07	-
LOC_Os04g39320	73.8233	9.8067	-2.9122	1.21E-10	4.79E-07	-
LOC_Os07g03740	53.2900	4.2000	-3.6654	2.23E-10	7.73E-07	-
LOC_Os06g21240	3.0700	15.3067	2.3178	4.74E-10	1.46E-06	-
LOC_Os04g48200	0.0400	1.7267	5.4318	5.56E-10	1.54E-06	CYP87A3
LOC_Os04g39300	39.2800	2.3167	-4.0837	8.80E-10	2.21E-06	-
LOC_Os06g20790	4.4933	0.2133	-4.3966	9.57E-10	2.21E-06	AAMT1I
LOC_Os08g29980	0.3200	1.5400	2.2668	1.05E-09	2.24E-06	-
LOC_Os04g30330	1.5500	0.0633	-4.6132	1.16E-09	2.29E-06	WAKL2
LOC_Os10g02380	15.7500	34.7667	1.1424	2.73E-09	4.76E-06	Os10g0113000
LOC_Os02g21925	73.9100	0.2700	-8.0967	2.74E-09	4.76E-06	-
LOC_Os11g46900	5.7300	0.0867	-6.0469	3.27E-09	5.34E-06	WAK5
LOC_Os04g27020	13.2767	0.2800	-5.5673	3.86E-09	5.56E-06	CYP71Z7
LOC_Os01g68730	4.5433	0.4467	-3.3465	4.01E-09	5.56E-06	-
LOC_Os07g29960	3.9500	0.1067	-5.2107	4.01E-09	5.56E-06	CYP87A3
LOC_Os07g35940	1.4833	11.2400	2.9217	5.02E-09	6.63E-06	BMY1
LOC_Os12g24320	58.7500	5.5467	-3.4049	6.26E-09	7.89E-06	bcsl1b
LOC_Os11g37970	10.5100	72.5933	2.7881	8.28E-09	9.54E-06	PR4A
LOC_Os04g56910	2.0233	0.0010	-10.9825	8.30E-09	9.54E-06	ACT-2
LOC_Os07g12890	37.8333	6.2433	-2.5993	8.60E-09	9.54E-06	ZIP8
MSTRG.26475	68.4433	2.4500	-4.8041	9.82E-09	1.05E-05	--
LOC_Os12g31540	3.5533	0.0667	-5.7361	1.09E-08	1.12E-05	CNR13
LOC_Os01g44960	1.4033	5.9100	2.0743	1.91E-08	1.89E-05	ephx3
LOC_Os06g21210	13.9033	48.5367	1.8036	2.07E-08	1.98E-05	-
LOC_Os09g39410	0.5233	1.7900	1.7742	2.60E-08	2.41E-05	FAR4
LOC_Os10g39130	105.8767	22.5467	-2.2314	3.90E-08	3.49E-05	MADS56
LOC_Os07g37385	2.3133	0.0010	-11.1758	8.31E-08	7.20E-05	fhaB
LOC_Os07g11970	3.9067	0.1300	-4.9094	9.94E-08	8.35E-05	CYP71Z7
LOC_Os11g24070	14.4933	70.4667	2.2816	1.18E-07	9.59E-05	LTP
LOC_Os10g34940	11.1233	0.2900	-5.2614	1.42E-07	0.00011	-
LOC_Os08g15710	6.3900	0.4800	-3.7347	1.43E-07	0.00011	-
LOC_Os11g31090	1.2733	4.4967	1.8202	2.16E-07	0.0001621	HHT1
LOC_Os05g46370	0.4700	2.5333	2.4303	2.27E-07	0.0001639	BHLH87

LOC_Os07g48260	9.9700	1.7033	-2.5492	2.31E-07	0.0001639	WRKY54
LOC_Os02g17590	9.4667	0.2767	-5.0966	2.81E-07	0.0001944	-
LOC_Os01g02780	15.5200	3.3467	-2.2133	2.87E-07	0.0001944	At1g67000
LOC_Os09g13440	130.0233	11.6367	-3.4820	3.46E-07	0.0002285	-
LOC_Os01g63180	4.4033	0.1500	-4.8756	3.61E-07	0.0002331	LAC6
LOC_Os09g31430	2.9933	14.2333	2.2494	3.92E-07	0.0002451	BGLU30
LOC_Os05g08420	18.1067	1.8900	-3.2601	3.98E-07	0.0002451	-
LOC_Os07g14820	1.1467	0.0010	-10.1632	4.33E-07	0.0002595	At3g47570
LOC_Os04g54240	3.0733	11.5600	1.9113	4.40E-07	0.0002595	-
LOC_Os11g44630	11.0967	0.5167	-4.4247	6.12E-07	0.0003538	-
LOC_Os05g47870	7.3800	16.2533	1.1390	6.80E-07	0.0003778	-
LOC_Os07g01340	0.3067	3.4833	3.5057	6.83E-07	0.0003778	GA2OX8
LOC_Os07g09050	0.2667	2.3233	3.1231	6.95E-07	0.0003778	At5g03795
LOC_Os04g22120	15.7333	0.9233	-4.0908	7.46E-07	0.0003978	LECRK91
LOC_Os04g07110	7.9700	0.3600	-4.4685	7.84E-07	0.00041	-
LOC_Os06g38120	32.6433	2.0533	-3.9907	8.11E-07	0.0004166	-
LOC_Os01g68460	5.0900	0.0767	-6.0529	9.73E-07	0.0004907	-
LOC_Os06g29730	90.2867	16.4567	-2.4558	1.01E-06	0.0005005	-
LOC_Os07g31070	5.3900	0.3367	-4.0009	1.08E-06	0.0005253	At2g01680
LOC_Os06g04010	4.2500	11.5967	1.4482	1.17E-06	0.0005597	Os06g0130600
LOC_Os11g47630	7.7100	0.5633	-3.7747	1.25E-06	0.0005867	ZAT8
LOC_Os08g08690	10.8067	1.3500	-3.0009	1.27E-06	0.0005867	-
LOC_Os07g48050	10.2867	0.5433	-4.2428	1.32E-06	0.0006004	-
LOC_Os06g34960	2.5800	0.3333	-2.9523	1.34E-06	0.0006004	At1g67520
LOC_Os11g05800	14.3733	1.3667	-3.3947	1.39E-06	0.0006129	HVA22J
LOC_Os10g37400	3.4933	11.5600	1.7265	1.53E-06	0.0006627	-
MSTRG.7258	10.7000	0.6733	-3.9901	1.59E-06	0.0006728	--
LOC_Os05g34170	3.6433	12.4100	1.7682	1.60E-06	0.0006728	TUBB6
LOC_Os12g36830	137.7633	7.6633	-4.1681	1.81E-06	0.0007485	-
LOC_Os02g50460	9.4800	0.3533	-4.7458	1.85E-06	0.0007534	PUB20
LOC_Os06g43384	1.4233	0.0967	-3.8801	1.90E-06	0.0007619	CYP71D7
LOC_Os01g28450	778.0033	28.0167	-4.7954	1.99E-06	0.0007864	-
LOC_Os11g19240	0.0010	0.3967	8.6318	2.12E-06	0.0008283	AVT1
LOC_Os01g10320	2.0667	6.7333	1.7040	2.16E-06	0.0008308	HOX29
LOC_Os06g34020	0.4467	2.0300	2.1842	2.43E-06	0.0009199	At1g32860
MSTRG.22412	131.1800	6.0433	-4.4401	2.45E-06	0.0009199	--
LOC_Os11g41034	2.1300	0.1367	-3.9621	2.51E-06	0.0009284	-
LOC_Os09g25070	37.3600	5.1600	-2.8561	2.79E-06	0.0010169	WRKY40
LOC_Os04g29790	3.0733	0.3333	-3.2048	2.89E-06	0.0010402	-
MSTRG.21425	5.9933	0.3900	-3.9418	2.95E-06	0.0010478	--
LOC_Os03g14642	5.4500	46.4333	3.0908	3.19E-06	0.0011189	-
LOC_Os07g34850	9.3867	0.7733	-3.6015	3.42E-06	0.0011855	nep1
LOC_Os04g50940	18.0467	0.6167	-4.8711	3.52E-06	0.0011882	NPF8.3

LOC_Os09g39650	32.5233	4.4200	-2.8794	3.55E-06	0.0011882	PUB33
LOC_Os02g18070	5.9833	0.5800	-3.3668	3.61E-06	0.0011882	RGA2
LOC_Os03g21710	15.8867	1.9067	-3.0587	3.67E-06	0.0011882	WRKY70
MSTRG.9448	1.7233	0.0010	-10.7510	3.68E-06	0.0011882	--
LOC_Os09g26144	3.6933	0.4133	-3.1595	3.68E-06	0.0011882	GLR2.8
LOC_Os08g20200	0.2533	1.0767	2.0875	3.78E-06	0.0012036	FAR1
LOC_Os08g13870	1.5333	0.1233	-3.6360	3.84E-06	0.0012036	RLK1
LOC_Os05g14820	1.4267	0.0100	-7.1565	3.86E-06	0.0012036	LHT1
MSTRG.17789	0.3667	4.2333	3.5293	3.93E-06	0.0012114	--
LOC_Os09g29560	1.4100	0.1500	-3.2327	4.03E-06	0.0012282	WAK3
LOC_Os02g36030	1.7467	0.0800	-4.4485	4.20E-06	0.0012575	CYP76M5
LOC_Os07g03040	9.1733	0.8733	-3.3928	4.22E-06	0.0012575	-
LOC_Os02g58100	0.5133	11.6600	4.5055	5.27E-06	0.0015556	-
LOC_Os11g46810	0.9633	0.0667	-3.8530	5.35E-06	0.0015608	WAK1
LOC_Os09g18050	3.0800	0.3300	-3.2224	5.71E-06	0.0016503	-
LOC_Os01g13610	6.3167	0.1467	-5.4286	5.92E-06	0.0016917	IRL
LOC_Os12g01490	10.2933	1.3600	-2.9200	5.98E-06	0.0016924	KAN4
LOC_Os01g41820	12.8433	3.2167	-1.9974	6.31E-06	0.0017678	CYP72A15
LOC_Os01g09190	4.4933	0.2367	-4.2469	7.05E-06	0.0019446	-
LOC_Os01g09990	4.3167	0.2400	-4.1688	7.08E-06	0.0019446	BHLH
LOC_Os11g44700	2.6500	0.2033	-3.7041	7.18E-06	0.0019512	-
LOC_Os11g12320	4.9833	0.9433	-2.4013	7.94E-06	0.0021388	RPM1
LOC_Os05g04930	8.1000	0.2600	-4.9613	8.21E-06	0.0021902	HSR201
LOC_Os02g42450	5.6900	20.4067	1.8425	8.42E-06	0.0022239	grip22
LOC_Os04g49194	36.1600	3.8500	-3.2315	8.69E-06	0.002273	-
LOC_Os10g30560	10.4300	1.6000	-2.7046	1.03E-05	0.0026735	UGT86A1
LOC_Os05g30760	10.1500	0.2000	-5.6653	1.06E-05	0.0027275	PIR7B
LOC_Os10g04450	2.5633	10.8000	2.0749	1.08E-05	0.0027471	-
LOC_Os03g46180	0.1667	0.8767	2.3951	1.21E-05	0.0030409	-
LOC_Os07g33780	109.2567	12.1333	-3.1707	1.38E-05	0.0034543	ABCG43
LOC_Os12g38180	65.1200	20.2600	-1.6845	1.48E-05	0.0036686	HSC-2
LOC_Os02g18080	5.2733	0.7667	-2.7820	1.76E-05	0.0043144	RGA2
LOC_Os11g28540	3.2300	0.2500	-3.6915	1.82E-05	0.0044049	-
LOC_Os04g54020	0.3900	1.2333	1.6610	1.83E-05	0.0044049	SD18
LOC_Os03g24820	4.1567	0.1867	-4.4769	1.84E-05	0.0044049	-
LOC_Os02g13430	5.9900	0.9667	-2.6315	1.88E-05	0.0044467	HSL1
LOC_Os02g39660	9.7733	1.3300	-2.8774	1.90E-05	0.0044762	EFR
LOC_Os01g14630	8.6833	0.8333	-3.3813	1.93E-05	0.0045091	GGPPS1
LOC_Os06g38870	8.7833	1.0733	-3.0327	1.98E-05	0.00454	-
LOC_Os06g16350	1.4700	6.0167	2.0331	1.98E-05	0.00454	PER11
LOC_Os10g39710	22.4300	3.3500	-2.7432	2.02E-05	0.0045812	SSL5
LOC_Os09g18360	3.5000	0.4600	-2.9276	2.13E-05	0.004806	At1g51860
LOC_Os02g32650	0.0010	0.4933	8.9464	2.15E-05	0.0048141	-

LOC_Os02g36280	3.1833	0.0933	-5.0920	2.18E-05	0.0048322	CYP76M6
LOC_Os11g18194	2.0167	6.1633	1.6117	2.29E-05	0.0050337	Os11g0285000
LOC_Os06g41100	2.1400	0.5867	-1.8670	2.36E-05	0.0051574	-
LOC_Os04g37970	0.9933	0.0900	-3.4643	2.41E-05	0.005193	STP5
MSTRG.3477	7.7300	2.4333	-1.6675	2.42E-05	0.005193	HEXO3
LOC_Os06g38764	173.6867	12.0100	-3.8542	2.46E-05	0.0052375	-
LOC_Os09g22000	2.8900	10.4133	1.8493	2.51E-05	0.0053174	yidA
LOC_Os06g38830	6.0800	0.7667	-2.9874	2.57E-05	0.0053929	At3g47570
LOC_Os11g31540	26.6433	2.1433	-3.6358	2.60E-05	0.0054301	SERK1
LOC_Os07g25810	0.9467	4.6167	2.2859	2.71E-05	0.0056157	Sbsn
LOC_Os11g33394	2.8300	0.5133	-2.4628	2.82E-05	0.0057878	At3g47200
LOC_Os04g30250	32.5500	5.2333	-2.6369	3.23E-05	0.0065813	WAK2
LOC_Os02g36110	8.3467	0.3700	-4.4956	3.25E-05	0.0065813	CYP76M7
LOC_Os12g36850	135.6733	9.2200	-3.8792	3.44E-05	0.0069144	BETV1D
LOC_Os03g18060	0.0010	0.1100	6.7814	3.51E-05	0.0070033	At1g48120
LOC_Os06g32990	13.8400	0.2000	-6.1127	3.61E-05	0.0071284	PER36
LOC_Os06g06750	0.4300	0.0010	-8.7482	3.62E-05	0.0071284	MADS5
LOC_Os04g41680	11.3200	70.6367	2.6415	3.87E-05	0.0074975	Cht5
LOC_Os02g53970	0.1700	0.0010	-7.4094	3.89E-05	0.0074975	SBT1.7
LOC_Os12g36860	6.9867	0.2367	-4.8837	3.89E-05	0.0074975	BETV1G
LOC_Os12g36880	279.1700	30.7333	-3.1833	4.03E-05	0.0077084	MALD1
LOC_Os11g07270	0.7033	2.4467	1.7985	4.14E-05	0.0078627	At3g47570
LOC_Os07g33700	0.5733	0.0400	-3.8413	4.48E-05	0.0084503	At1g48120
LOC_Os04g41620	43.7500	307.7567	2.8144	4.64E-05	0.0087036	Cht4
LOC_Os01g68720	13.0100	2.1900	-2.5706	4.71E-05	0.008762	-
LOC_Os06g38340	9.4233	1.9200	-2.2951	4.83E-05	0.0089273	At3g47570
LOC_Os04g50700	1.9900	10.1800	2.3549	5.15E-05	0.0094517	-
LOC_Os11g33970	13.1900	3.3500	-1.9772	5.45E-05	0.0099409	At3g47200
LOC_Os02g17600	0.9333	0.0167	-5.8074	5.65E-05	0.0102232	FT1
LOC_Os08g39840	128.2300	6.1367	-4.3851	5.68E-05	0.0102232	CM-LOX1
LOC_Os04g30060	8.4067	0.7733	-3.4424	5.74E-05	0.0102704	WAKL10
LOC_Os12g43450	12.6267	132.0033	3.3860	5.92E-05	0.0105273	tlp
LOC_Os08g26230	211.2167	15.7633	-3.7441	5.97E-05	0.0105392	-
LOC_Os07g10910	1.8467	4.2467	1.2014	6.07E-05	0.0106545	EXO70A1
LOC_Os04g07260	2.8033	0.1833	-3.9346	6.39E-05	0.0111394	-
LOC_Os12g02320	3642.2367	7574.1300	1.0563	6.47E-05	0.0112138	LTP
LOC_Os02g50470	46.0333	4.7367	-3.2807	6.58E-05	0.011327	-
LOC_Os02g46810	8.5733	2.5733	-1.7362	6.64E-05	0.0113671	AAP4
LOC_Os01g42110	3.4533	10.3400	1.5822	6.86E-05	0.0116409	SWEET6A
LOC_Os01g43750	16.5600	1.5167	-3.4487	6.88E-05	0.0116409	CYP72A15
LOC_Os01g02930	0.9767	2.5767	1.3996	6.94E-05	0.0116546	pomgnt2
LOC_Os02g36190	7.3933	0.6867	-3.4285	6.98E-05	0.0116546	CYP71Z7
LOC_Os10g33550	1.7233	34.9533	4.3422	7.23E-05	0.012006	FAO1

LOC_Os07g31190	1.9167	0.1100	-4.1230	7.44E-05	0.012283	WAK2
LOC_Os05g29810	0.1767	1.1067	2.6471	7.62E-05	0.0124751	RAP2-3
LOC_Os05g37600	0.4733	2.0867	2.1403	7.65E-05	0.0124751	GPAT6
LOC_Os11g31530	58.9267	2.2733	-4.6960	7.73E-05	0.0124935	SERK1
LOC_Os04g30240	26.7000	4.7600	-2.4878	7.78E-05	0.0124935	WAK2
LOC_Os09g09510	3.1033	0.4900	-2.6630	7.79E-05	0.0124935	LECRK41
LOC_Os01g61160	0.8167	2.5100	1.6199	7.87E-05	0.0125456	LAC3
LOC_Os04g59540	0.5567	3.8767	2.7999	8.01E-05	0.0126979	FAB1B
LOC_Os04g54300	7.3233	24.0833	1.7175	8.14E-05	0.0128253	-
LOC_Os08g39850	74.3033	5.4567	-3.7673	8.23E-05	0.0128938	CM-LOX2
LOC_Os04g52190	44.0767	4.6400	-3.2478	8.37E-05	0.0130405	VSR7
LOC_Os06g37560	14.3200	89.4867	2.6436	8.44E-05	0.0130742	Os06g0573600
LOC_Os11g12340	39.3133	3.8033	-3.3697	8.64E-05	0.013304	RPM1
LOC_Os09g08130	85.6433	5.1700	-4.0501	8.89E-05	0.0136029	At2g04400
LOC_Os01g11720	5.0767	1.3367	-1.9252	8.93E-05	0.0136029	-
LOC_Os02g28340	4.9133	13.0700	1.4115	8.98E-05	0.0136029	3AT1
LOC_Os08g42570	1.4467	0.0500	-4.8547	9.26E-05	0.0138907	At3g47200
LOC_Os04g46130	0.1167	3.0467	4.7068	9.27E-05	0.0138907	-
LOC_Os03g58320	25.1067	1.0400	-4.5934	9.35E-05	0.0139334	BX1
LOC_Os09g27750	28.4767	74.7133	1.3916	9.79E-05	0.0145215	ACO1
LOC_Os01g71670	14.8900	67.8033	2.1870	0.0001	0.0147937	-
LOC_Os10g05250	6.9167	1.1800	-2.5513	0.0001	0.0149413	WAKL4
LOC_Os02g36140	21.0633	1.6700	-3.6568	0.0001	0.0149413	KSL7
LOC_Os09g26670	0.2267	4.5867	4.3388	0.00011	0.0151886	-
LOC_Os03g57970	3.6567	11.7100	1.6791	0.00011	0.0161455	LTPG2
LOC_Os03g06890	1.5300	0.2067	-2.8882	0.00012	0.0167085	MYOB1
LOC_Os01g58194	8.1700	17.7967	1.1232	0.00012	0.0167085	Os01g0794400
LOC_Os11g07690	2.2567	0.0400	-5.8180	0.00012	0.0170014	DIR19
LOC_Os09g27930	1.1300	0.1200	-3.2352	0.00012	0.0170014	Ub-CEP52-2
LOC_Os04g25060	31.9067	3.3933	-3.2331	0.00012	0.0170014	CRRSP38
LOC_Os03g09910	11.2567	1.5800	-2.8328	0.00012	0.0171597	ALD1
LOC_Os10g13850	7.1400	107.8833	3.9174	0.00012	0.0172303	-
LOC_Os09g29690	1.4433	9.4000	2.7033	0.00013	0.0174415	CjBAp12
LOC_Os11g12260	4.0833	0.3633	-3.4904	0.00013	0.0174445	RPM1
LOC_Os03g25850	0.5933	6.6133	3.4785	0.00013	0.0175621	-
LOC_Os05g46790	14.1667	0.4000	-5.1464	0.00013	0.0180067	-
LOC_Os01g19260	3.4767	0.4367	-2.9931	0.00014	0.0187412	bcsl1b
LOC_Os03g47016	2.7100	0.4100	-2.7246	0.00014	0.0188792	OSH10
LOC_Os02g37160	19.9433	6.2533	-1.6732	0.00014	0.0188792	-
LOC_Os02g53100	1.3100	0.0633	-4.3705	0.00015	0.0197583	WRKY9
LOC_Os04g03830	5.6733	0.6600	-3.1037	0.00015	0.0197802	WAK5
LOC_Os12g32850	2.3933	0.1667	-3.8440	0.00015	0.0201953	CYP71E1
LOC_Os11g28530	2.0533	0.2167	-3.2444	0.00016	0.020662	KSL8

LOC_Os08g15149	32.9633	5.5400	-2.5729	0.00016	0.020662	-
LOC_Os08g14760	0.8833	2.5967	1.5556	0.00016	0.020662	4CL1
LOC_Os12g26510	0.2700	1.1267	2.0610	0.00016	0.0207257	-
LOC_Os02g49920	14.2233	70.3600	2.3065	0.00017	0.02147	KCS5
LOC_Os11g42200	23.4100	1.5033	-3.9609	0.00017	0.0217745	LAC19
LOC_Os05g01370	2.7500	0.1833	-3.9069	0.00017	0.0218241	PGIP
LOC_Os07g34260	66.9467	5.5467	-3.5933	0.00017	0.0218825	Os07g0271500
LOC_Os11g01480	10.1200	1.9467	-2.3781	0.00018	0.0229868	KAN4
LOC_Os12g11510	0.1833	0.0010	-7.5183	0.00018	0.0231217	RLP12
LOC_Os01g05780	0.0010	0.6533	9.3517	0.00018	0.0231217	PAS2A
LOC_Os04g46210	1.4233	0.2267	-2.6506	0.00019	0.0231217	-
LOC_Os12g08930	7.8767	0.8133	-3.2757	0.00019	0.0232069	-
LOC_Os01g52070	0.0633	0.4433	2.8074	0.00019	0.023813	Os01g0718700
LOC_Os01g02790	3.5600	0.9100	-1.9679	0.0002	0.0246212	At5g39030
LOC_Os06g35700	9.5500	0.4133	-4.5301	0.00021	0.0252355	-
LOC_Os12g26690	0.1500	0.9000	2.5850	0.00021	0.025241	-
LOC_Os02g13260	0.5167	0.0167	-4.9542	0.00021	0.0254513	At4g22030
LOC_Os12g37650	88.7533	702.3700	2.9844	0.00021	0.0254513	-
LOC_Os12g32814	3.5867	0.4833	-2.8916	0.00021	0.0258031	ABCG50
LOC_Os06g45960	2.2467	0.0867	-4.6962	0.00022	0.0258769	CYP71Z6
LOC_Os04g10010	8.6633	0.4333	-4.3214	0.00022	0.0264756	Os04g0179200
LOC_Os01g11810	4.0467	11.3367	1.4862	0.00022	0.0264756	TBL38
MSTRG.4545	4.3333	0.7633	-2.5051	0.00022	0.0264756	--
LOC_Os01g63710	4.2400	1.2867	-1.7204	0.00022	0.0264756	CDC6
LOC_Os07g14740	64.2267	21.0733	-1.6078	0.00023	0.0266849	-
LOC_Os05g11910	27.5367	71.5833	1.3783	0.00023	0.027208	At5g45910
LOC_Os04g41640	22.0033	199.3667	3.1796	0.00023	0.027208	Cht4
LOC_Os11g22150	0.6100	0.0010	-9.2527	0.00024	0.027208	-
LOC_Os03g48626	162.7100	351.7333	1.1122	0.00024	0.027208	-
LOC_Os01g02130	21.7067	4.4900	-2.2734	0.00024	0.0274043	-
LOC_Os04g03180	8.6500	3.1300	-1.4665	0.00024	0.0274043	BRL2
LOC_Os11g40850	10.6167	0.9833	-3.4325	0.00024	0.0274166	-
LOC_Os01g41730	0.1533	1.5067	3.2966	0.00025	0.0279936	At2g42960
LOC_Os06g49970	1.5300	3.8867	1.3450	0.00025	0.0281522	AMY2A
LOC_Os01g04550	39.6600	5.6033	-2.8233	0.00025	0.0281522	GDPDL2
LOC_Os12g37580	0.2700	0.0433	-2.6394	0.00027	0.0299615	ABCC10
LOC_Os09g33720	0.0100	0.3033	4.9228	0.00027	0.0301954	sec61a
LOC_Os11g02369	448.2033	2952.4367	2.7197	0.00027	0.0302743	LTP2-A
LOC_Os02g07930	2.1967	9.0733	2.0463	0.00028	0.030336	BBX32
LOC_Os10g42040	50.6933	4.5800	-3.4684	0.00028	0.030336	-
LOC_Os07g30600	7.0233	0.2300	-4.9325	0.00028	0.030336	FAR1
LOC_Os05g37450	1.7933	4.3700	1.2850	0.00028	0.030336	GWD3
LOC_Os06g39080	0.2667	0.0010	-8.0589	0.00028	0.0305004	UGT73B5

LOC_Os03g17350	1.3900	3.7400	1.4280	0.00028	0.0306244	RCN1
LOC_Os01g02560	20.6500	6.6433	-1.6362	0.00028	0.0306244	At1g67000
LOC_Os06g39640	4.9800	11.7200	1.2348	0.00028	0.0306244	PHN1
LOC_Os01g28500	79.3167	353.8600	2.1575	0.00029	0.0315645	PRMS
LOC_Os02g55380	0.2700	1.6033	2.5700	0.0003	0.0316295	ERF003
LOC_Os01g32120	0.9200	2.6400	1.5208	0.0003	0.0320945	CML11
LOC_Os07g44060	2.2000	7.8633	1.8376	0.0003	0.0321539	-
LOC_Os12g44320	0.0800	0.4933	2.6245	0.0003	0.0321712	At5g35370
LOC_Os01g61940	0.7100	1.4900	1.0694	0.00031	0.0331157	ABCG1
LOC_Os05g39770	4.6400	35.3500	2.9295	0.00032	0.034045	At3g08860
LOC_Os05g48840	0.4533	3.3733	2.8955	0.00033	0.0346946	-
LOC_Os07g40870	0.3100	1.9100	2.6232	0.00033	0.0346946	-
LOC_Os08g39830	0.0367	0.3767	3.3607	0.00033	0.0347183	EIL3
LOC_Os06g43320	0.9967	7.3000	2.8727	0.00034	0.0347183	CYP71D55
LOC_Os11g07230	13.1200	5.1133	-1.3594	0.00034	0.0348454	At3g47570
LOC_Os05g32970	43.1033	100.0167	1.2144	0.00034	0.0348454	-
LOC_Os07g26630	0.1533	2.2300	3.8623	0.00034	0.0348454	PIP2-4
LOC_Os10g41980	168.8033	57.5133	-1.5534	0.00034	0.0348454	-
LOC_Os09g29210	2.6167	0.7800	-1.7462	0.00034	0.0348454	PUP3
LOC_Os02g16995	0.3500	0.0010	-8.4512	0.00034	0.0348596	-
LOC_Os05g31740	4.5600	0.5533	-3.0428	0.00035	0.0348774	CYP94A1
LOC_Os04g56040	18.4000	1.4500	-3.6656	0.00035	0.0349873	-
LOC_Os07g03170	13.9300	2.0467	-2.7668	0.00035	0.0349873	-
LOC_Os12g36940	17.1933	2.8100	-2.6132	0.00036	0.0361428	CBP60B
LOC_Os10g30880	28.4800	5.5000	-2.3724	0.00036	0.0361428	-
LOC_Os01g66760	8.2333	1.1433	-2.8482	0.00036	0.0361428	At5g48380
LOC_Os02g15930	0.4333	2.2500	2.3764	0.00038	0.0372215	BAG6
LOC_Os09g20090	7.9200	0.7033	-3.4932	0.00038	0.0374586	AAO
LOC_Os02g49480	20.8233	6.2033	-1.7471	0.00039	0.0380958	BIM1
LOC_Os03g05840	0.3200	1.4800	2.2095	0.00039	0.0382971	xlnD
LOC_Os11g45190	0.8600	0.0567	-3.9238	0.0004	0.0386181	RGA2
LOC_Os02g33380	0.0567	2.0967	5.2095	0.0004	0.0387008	-
LOC_Os12g02700	12.1633	27.4633	1.1750	0.0004	0.038721	-
LOC_Os05g13360	0.1567	3.4667	4.4678	0.00041	0.0389473	-
LOC_Os03g45860	3.5733	0.0433	-6.3656	0.00041	0.039072	SAUR71
LOC_Os06g37410	2.7033	0.2633	-3.3598	0.00041	0.0394916	BHLH
LOC_Os09g37880	1.1733	0.0933	-3.6521	0.00042	0.0395519	At1g11330
LOC_Os04g15650	9.4700	2.4100	-1.9743	0.00042	0.0395519	At3g47570
LOC_Os06g10830	0.5233	0.0233	-4.4873	0.00042	0.0395519	-
LOC_Os04g10000	5.6233	0.6167	-3.1889	0.00043	0.0400222	Os04g0179200
LOC_Os07g38250	1.3767	0.1267	-3.4421	0.00043	0.0405275	LECRK91
LOC_Os11g12240	4.8833	0.3433	-3.8302	0.00044	0.0407495	RPM1
LOC_Os10g07229	24.0800	53.7900	1.1595	0.00044	0.0407821	At1g64710

LOC_Os04g06734	3.3767	0.6767	-2.3191	0.00045	0.0416195	-
LOC_Os02g02380	1.0967	13.7333	3.6465	0.00046	0.0424505	RUP2
LOC_Os03g25490	0.4167	1.5867	1.9290	0.00047	0.0427305	CYP734A1
LOC_Os03g14630	1.4200	6.7467	2.2483	0.00048	0.0434227	-
LOC_Os06g09980	9.6567	1.7933	-2.4289	0.00048	0.0434227	-
LOC_Os08g41960	1.1067	6.5400	2.5631	0.00048	0.0434592	AGL3
LOC_Os12g11860	3.5900	1.1400	-1.6550	0.00048	0.0435523	RLP12
LOC_Os08g44870	20.4167	44.4733	1.1232	0.00049	0.0441008	TT12
LOC_Os07g02440	0.8333	5.4800	2.7172	0.00049	0.0441008	PER51
LOC_Os08g29760	0.5633	2.4533	2.1227	0.00049	0.0441008	-
LOC_Os01g05870	2.0100	0.3833	-2.3905	0.0005	0.0444075	At3g47570
LOC_Os03g53360	0.5733	3.3833	2.5610	0.00051	0.0453591	CER26L
LOC_Os11g07200	0.3533	0.0567	-2.6405	0.00051	0.0453591	At3g47570
LOC_Os05g13320	0.1900	0.0010	-7.5699	0.00052	0.0456617	SLAH3
LOC_Os12g43580	8.5600	1.8400	-2.2179	0.00053	0.0464721	-
LOC_Os09g39430	3.2233	23.7867	2.8835	0.00054	0.0471049	At5g03610
LOC_Os07g09190	20.6833	3.9767	-2.3788	0.00054	0.0471681	Os07g0190000
LOC_Os02g13460	1.5233	0.3733	-2.0287	0.00054	0.0475122	HSL1
LOC_Os09g25850	10.4467	40.3767	1.9505	0.00056	0.0485272	CER3

Table S5. Statistical data of total differentially expressed genes (DEGs) for SN_CK vs. SN_D.

id	SN_CK _mean	SN_D _mean	log2(fc)	PValue	FDR	Symbol
LOC_Os11g46900	5.7300	0.0500	-6.8405	1.00E-24	2.71E-20	WAK5
LOC_Os09g39770	0.0010	15.7500	13.9431	1.31E-20	1.77E-16	-
LOC_Os09g13440	130.0233	3.4933	-5.2180	4.04E-19	2.90E-15	-
LOC_Os07g03730	471.4467	9.1467	-5.6877	4.29E-19	2.90E-15	-
LOC_Os04g39320	73.8233	3.6200	-4.3500	3.90E-18	2.11E-14	-
LOC_Os02g37160	19.9433	3.7733	-2.4020	5.91E-18	2.66E-14	-
LOC_Os03g10110	3.6500	0.4067	-3.1660	1.51E-17	5.54E-14	AMP2-2
LOC_Os07g05700	17.2667	2.9033	-2.5722	1.64E-17	5.54E-14	-
LOC_Os03g21710	15.8867	1.5800	-3.3298	2.81E-16	8.45E-13	WRKY70
LOC_Os07g03740	53.2900	2.8333	-4.2333	5.02E-16	1.36E-12	-
LOC_Os01g68740	84.6033	8.4233	-3.3283	8.45E-15	2.08E-11	-
LOC_Os08g15710	6.3900	0.1700	-5.2322	4.62E-14	1.04E-10	-
LOC_Os08g08690	10.8067	1.8933	-2.5129	5.03E-14	1.05E-10	-
LOC_Os07g29960	3.9500	0.0010	-11.9476	1.00E-13	1.94E-10	CYP87A3
LOC_Os03g08480	7.9167	26.6533	1.7514	1.08E-13	1.94E-10	BP-73
LOC_Os06g29730	90.2867	13.3533	-2.7573	1.35E-13	2.29E-10	-
LOC_Os03g55180	13.7367	2.6900	-2.3524	1.90E-13	3.03E-10	-
LOC_Os11g03230	8.9200	1.1167	-2.9978	2.18E-13	3.27E-10	APY3
LOC_Os11g31530	58.9267	1.4100	-5.3852	3.00E-13	4.26E-10	SERK1
LOC_Os01g63180	4.4033	0.0833	-5.7236	3.41E-13	4.61E-10	LAC6
LOC_Os08g31250	4.9467	0.2733	-4.1777	1.04E-12	1.34E-09	Os03g0405500
LOC_Os01g19260	3.4767	0.1667	-4.3827	1.61E-12	1.98E-09	bcsl1b
LOC_Os06g38450	119.3033	17.4400	-2.7742	2.94E-12	3.36E-09	SAG39
LOC_Os10g39260	10.6667	0.2267	-5.5564	2.99E-12	3.36E-09	nep1
LOC_Os01g02790	3.5600	0.4867	-2.8709	3.42E-12	3.70E-09	At5g39030
LOC_Os11g31540	26.6433	0.8067	-5.0457	6.20E-12	6.45E-09	SERK1
LOC_Os12g29160	3.2133	0.1000	-5.0060	7.81E-12	7.82E-09	-
LOC_Os06g35700	9.5500	0.0667	-7.1624	2.79E-11	2.69E-08	-
LOC_Os01g13610	6.3167	0.0467	-7.0806	2.95E-11	2.75E-08	IRL
LOC_Os02g16940	16.5667	0.4867	-5.0892	4.93E-11	4.45E-08	SBT3.5
LOC_Os03g62200	10.3967	0.9167	-3.5036	6.20E-11	5.41E-08	AMT3-2
LOC_Os08g04500	6.9167	0.3667	-4.2375	7.17E-11	6.06E-08	TPS2
LOC_Os07g12890	37.8333	4.7300	-2.9997	7.67E-11	6.21E-08	ZIP8
LOC_Os11g42200	23.4100	0.4400	-5.7335	7.80E-11	6.21E-08	LAC19
LOC_Os08g13870	1.5333	0.0633	-4.5976	1.32E-10	1.02E-07	RLK1
LOC_Os10g30560	10.4300	0.9133	-3.5135	1.83E-10	1.38E-07	UGT86A1
LOC_Os01g04280	32.9233	6.7100	-2.2947	2.13E-10	1.56E-07	SARD1
LOC_Os03g01300	72.8067	10.5400	-2.7882	2.40E-10	1.71E-07	AZI1
LOC_Os12g15680	3.9733	0.0300	-7.0492	2.66E-10	1.84E-07	LAC24

LOC_Os07g31070	5.3900	0.2833	-4.2497	2.72E-10	1.84E-07	At2g01680
LOC_Os10g39300	4.4200	0.1000	-5.4660	3.17E-10	2.09E-07	nep1
LOC_Os01g09150	3.9767	0.2000	-4.3135	3.79E-10	2.44E-07	-
MSTRG.26475	68.4433	2.9867	-4.5183	3.92E-10	2.47E-07	--
LOC_Os01g58280	56.4200	4.7900	-3.5581	5.32E-10	3.27E-07	SBT3.5
LOC_Os11g38790	4.5533	0.3867	-3.5578	5.67E-10	3.41E-07	-
LOC_Os03g29190	35.6533	9.3400	-1.9325	7.94E-10	4.62E-07	Os03g0405500
MSTRG.22412	131.1800	9.0833	-3.8522	8.02E-10	4.62E-07	--
LOC_Os03g01320	5.8800	0.4500	-3.7078	8.63E-10	4.86E-07	At4g12490
LOC_Os09g20090	7.9200	0.2100	-5.2370	9.33E-10	5.15E-07	AAO
LOC_Os06g43384	1.4233	0.0467	-4.9307	1.14E-09	6.16E-07	CYP71D7
LOC_Os11g47630	7.7100	0.1567	-5.6210	1.18E-09	6.22E-07	ZAT8
LOC_Os01g28450	778.0033	37.1400	-4.3887	1.20E-09	6.22E-07	-
LOC_Os06g46330	8.0933	2.6733	-1.5981	1.23E-09	6.26E-07	At1g56140
LOC_Os08g38710	65.9533	242.9767	1.8813	1.84E-09	9.20E-07	RFS1
ChrSy.fgenesh.gene	11.4800	1.8333	-2.6466	2.80E-09	1.38E-06	WAK3
LOC_Os03g24820	4.1567	0.2100	-4.3070	4.57E-09	2.21E-06	-
LOC_Os11g44630	11.0967	0.4833	-4.5210	5.35E-09	2.51E-06	-
LOC_Os07g07290	27.9667	4.4200	-2.6616	5.38E-09	2.51E-06	-
LOC_Os11g13750	2.2000	0.0267	-6.3663	5.91E-09	2.68E-06	-
LOC_Os11g01030	0.0010	0.4367	8.7704	6.00E-09	2.68E-06	-
LOC_Os07g34850	9.3867	0.6300	-3.8972	6.04E-09	2.68E-06	nep1
MSTRG.7258	10.7000	0.4667	-4.5191	7.03E-09	3.06E-06	--
LOC_Os06g09980	9.6567	1.6233	-2.5726	8.15E-09	3.50E-06	-
LOC_Os01g02780	15.5200	3.6167	-2.1014	9.15E-09	3.87E-06	At1g67000
LOC_Os01g43750	16.5600	1.8733	-3.1440	1.04E-08	4.34E-06	CYP72A15
LOC_Os03g58290	7.5300	0.4733	-3.9917	1.16E-08	4.74E-06	BX1
LOC_Os01g68730	4.5433	0.5233	-3.1179	1.40E-08	5.63E-06	-
LOC_Os05g14820	1.4267	0.0010	-10.4784	1.44E-08	5.74E-06	LHT1
LOC_Os07g37385	2.3133	0.0010	-11.1758	1.66E-08	6.50E-06	fhaB
MSTRG.21425	5.9933	0.2367	-4.6624	1.89E-08	7.32E-06	--
LOC_Os08g01950	3.1133	0.3100	-3.3281	2.18E-08	8.32E-06	At3g50280
LOC_Os04g27020	13.2767	0.3800	-5.1267	2.39E-08	8.96E-06	CYP71Z7
LOC_Os10g36848	16.2600	1.5033	-3.4351	3.10E-08	1.13E-05	CYP84A1
LOC_Os05g19910	9.4133	0.7200	-3.7086	3.10E-08	1.13E-05	TAT
LOC_Os01g68720	13.0100	2.2300	-2.5445	3.22E-08	1.16E-05	-
LOC_Os02g17590	9.4667	0.1533	-5.9481	3.38E-08	1.19E-05	-
LOC_Os04g30060	8.4067	0.5367	-3.9694	3.44E-08	1.19E-05	WAKL10
LOC_Os02g53100	1.3100	0.0167	-6.2965	3.44E-08	1.19E-05	WRKY9
LOC_Os10g34700	2.8300	0.4367	-2.6962	3.95E-08	1.34E-05	-
LOC_Os05g04930	8.1000	0.3067	-4.7232	3.95E-08	1.34E-05	HSR201
LOC_Os08g26230	211.2167	47.2667	-2.1598	4.45E-08	1.49E-05	-
LOC_Os04g58870	2.0200	13.1100	2.6982	4.57E-08	1.51E-05	EXO70A1

LOC_Os02g13460	1.5233	0.2067	-2.8819	5.10E-08	1.66E-05	HSL1
LOC_Os04g49194	36.1600	2.1433	-4.0765	5.17E-08	1.66E-05	-
LOC_Os08g30100	4.4133	0.2433	-4.1809	6.98E-08	2.21E-05	At1g06620
LOC_Os03g38800	1.9800	0.2633	-2.9105	7.03E-08	2.21E-05	SPAC644.07
LOC_Os05g08910	9.1667	2.1300	-2.1055	8.11E-08	2.50E-05	-
LOC_Os12g43580	8.5600	1.3233	-2.6934	8.13E-08	2.50E-05	-
LOC_Os03g12500	29.7733	1.7033	-4.1276	9.10E-08	2.76E-05	CYP74A2
LOC_Os08g33150	4.0300	0.4867	-3.0498	9.29E-08	2.79E-05	ODO1
LOC_Os09g18360	3.5000	0.4400	-2.9918	1.06E-07	3.16E-05	At1g51860
LOC_Os02g37180	10.7767	1.2500	-3.1079	1.11E-07	3.26E-05	-
LOC_Os02g13430	5.9900	0.8500	-2.8170	1.18E-07	3.42E-05	HSL1
LOC_Os12g36830	137.7633	6.9000	-4.3195	1.21E-07	3.48E-05	-
LOC_Os01g47070	158.5767	15.5367	-3.3514	1.34E-07	3.81E-05	CHIT3
LOC_Os01g38359	27.0500	4.6967	-2.5259	1.35E-07	3.81E-05	FKBP65
LOC_Os11g44430	9.7367	0.8033	-3.5994	1.37E-07	3.82E-05	At3g07070
LOC_Os12g36880	279.1700	21.2200	-3.7176	1.46E-07	4.03E-05	MALD1
LOC_Os08g39850	74.3033	4.3367	-4.0988	1.56E-07	4.26E-05	CM-LOX2
LOC_Os07g40690	2.1233	0.1033	-4.3610	1.86E-07	5.03E-05	BAN
LOC_Os04g10940	3.9833	0.2367	-4.0730	1.90E-07	5.08E-05	-
LOC_Os06g22290	1.2967	0.1367	-3.2461	1.94E-07	5.15E-05	LECRKS5
LOC_Os09g15560	1.3167	0.0867	-3.9253	2.06E-07	5.40E-05	-
LOC_Os05g25770	307.9167	97.3033	-1.6620	2.23E-07	5.79E-05	WRKY70
LOC_Os09g39650	32.5233	4.7600	-2.7724	2.36E-07	6.07E-05	PUB33
LOC_Os04g08550	20.3233	4.4333	-2.1967	2.70E-07	6.89E-05	Os10g0113000
LOC_Os06g05480	53.3667	21.1467	-1.3355	2.80E-07	7.04E-05	-
LOC_Os01g18744	3.1667	0.7767	-2.0276	2.81E-07	7.04E-05	TAX10
LOC_Os11g28184	3.0533	8.6867	1.5084	2.88E-07	7.15E-05	-
LOC_Os04g59200	150.3967	12.3000	-3.6120	3.07E-07	7.55E-05	Sb03g046810
LOC_Os06g38120	32.6433	4.1100	-2.9896	3.20E-07	7.79E-05	-
LOC_Os07g41960	6.8600	0.3900	-4.1367	3.27E-07	7.90E-05	-
LOC_Os04g30030	6.6333	0.7533	-3.1384	3.64E-07	8.70E-05	CRK10
LOC_Os02g48770	9.4200	1.0467	-3.1699	3.76E-07	8.92E-05	AAMT1
LOC_Os10g39710	22.4300	3.6467	-2.6208	3.82E-07	8.98E-05	SSL5
LOC_Os02g36140	21.0633	1.3767	-3.9355	3.88E-07	9.05E-05	KSL7
LOC_Os11g07690	2.2567	0.0010	-11.1400	4.11E-07	9.46E-05	DIR19
LOC_Os11g41034	2.1300	0.1867	-3.5123	4.13E-07	9.46E-05	-
LOC_Os02g50470	46.0333	3.6867	-3.6423	4.64E-07	0.0001055	-
LOC_Os09g26144	3.6933	0.5167	-2.8376	4.69E-07	0.0001057	GLR2.8
LOC_Os06g38870	8.7833	1.0967	-3.0016	4.93E-07	0.0001102	-
LOC_Os07g48260	9.9700	2.4000	-2.0546	5.55E-07	0.0001231	WRKY54
LOC_Os01g60740	0.9433	0.0010	-9.8816	5.66E-07	0.0001244	-
LOC_Os04g56030	10.2800	1.1700	-3.1353	5.90E-07	0.0001286	-
LOC_Os03g44710	10.1267	3.5633	-1.5069	6.33E-07	0.0001369	YAB2

LOC_Os02g50460	9.4800	0.5400	-4.1339	6.89E-07	0.0001479	PUB20
LOC_Os03g06705	0.9467	0.0267	-5.1497	7.65E-07	0.0001629	KCS11
LOC_Os09g23450	3.9567	0.0010	-11.9501	7.92E-07	0.0001673	-
LOC_Os05g50100	25.0267	2.2467	-3.4776	8.06E-07	0.000169	-
LOC_Os05g30760	10.1500	0.4500	-4.4954	8.17E-07	0.0001699	PIR7B
MSTRG.4664	4.3967	0.7667	-2.5197	8.37E-07	0.0001726	--
LOC_Os09g29560	1.4100	0.1700	-3.0521	8.44E-07	0.0001726	WAK3
LOC_Os12g04360	11.6000	2.3567	-2.2993	8.49E-07	0.0001726	CML36
LOC_Os09g13400	0.6567	0.0010	-9.3590	8.66E-07	0.0001748	-
LOC_Os12g11860	3.5900	0.8633	-2.0560	8.96E-07	0.000179	RLP12
LOC_Os10g14170	3.0700	23.2900	2.9234	9.00E-07	0.000179	-
LOC_Os02g41680	11.2000	3.3667	-1.7341	9.12E-07	0.0001799	ZB8
LOC_Os01g72910	21.9500	4.0700	-2.4311	9.29E-07	0.000182	ASR1
LOC_Os02g18070	5.9833	1.1567	-2.3710	9.52E-07	0.0001853	RGA2
LOC_Os01g40430	1.3167	0.0010	-10.3627	1.09E-06	0.0002101	WRKY9
LOC_Os05g45410	13.4467	2.7300	-2.3003	1.10E-06	0.0002101	HSFA4D
LOC_Os12g04690	0.9267	0.1467	-2.6595	1.10E-06	0.0002101	-
LOC_Os01g48020	2.2933	0.2767	-3.0512	1.16E-06	0.000219	At1g34300
LOC_Os12g14440	5.9667	0.3667	-4.0244	1.19E-06	0.0002235	SALT
LOC_Os11g44700	2.6500	0.2367	-3.4851	1.27E-06	0.0002375	-
LOC_Os08g31200	0.7600	0.0200	-5.2479	1.31E-06	0.0002413	UGT85A24
LOC_Os11g05800	14.3733	1.9533	-2.8794	1.31E-06	0.0002413	HVA22J
LOC_Os04g51880	87.1767	205.8100	1.2393	1.34E-06	0.0002437	GALAK
LOC_Os06g38764	173.6867	11.5967	-3.9047	1.34E-06	0.0002437	-
LOC_Os04g56040	18.4000	1.0267	-4.1637	1.38E-06	0.0002493	-
LOC_Os06g29844	11.3300	4.5900	-1.3036	1.42E-06	0.0002541	DTXL1
LOC_Os07g43800	18.1000	8.0467	-1.1695	1.56E-06	0.0002771	CML45
LOC_Os12g36940	17.1933	2.1167	-3.0220	1.58E-06	0.0002791	CBP60B
LOC_Os07g41410	74.7200	15.0733	-2.3095	1.60E-06	0.0002802	Ea1
LOC_Os01g59730	5.6800	1.4933	-1.9274	1.64E-06	0.0002861	RPL30
LOC_Os04g47420	10.4833	2.6933	-1.9606	1.65E-06	0.0002864	AATL1
LOC_Os11g37860	4.0033	1.7767	-1.1720	1.66E-06	0.0002864	RPP13
LOC_Os04g50940	18.0467	0.8033	-4.4896	1.78E-06	0.0003053	NPF8.3
LOC_Os03g13740	3.6700	0.2133	-4.1046	2.00E-06	0.0003402	PUB21
LOC_Os06g40050	19.3467	49.2500	1.3480	2.05E-06	0.0003459	BT1
LOC_Os02g57350	2.1500	0.3300	-2.7038	2.25E-06	0.0003774	-
LOC_Os10g42040	50.6933	6.8333	-2.8911	2.29E-06	0.0003819	-
LOC_Os03g20330	73.4267	22.3433	-1.7165	2.36E-06	0.0003911	-
LOC_Os01g02420	3.2367	1.1933	-1.4395	2.39E-06	0.0003936	At5g39020
LOC_Os06g04010	4.2500	11.7533	1.4675	2.59E-06	0.0004251	Os06g0130600
LOC_Os12g24320	58.7500	8.4333	-2.8004	2.61E-06	0.0004258	bcsl1b
LOC_Os01g51754	25.8567	65.6767	1.3448	2.64E-06	0.0004278	AMY3
LOC_Os12g43970	12.0300	1.8833	-2.6753	2.77E-06	0.0004466	EPHX2

LOC_Os06g45960	2.2467	0.0933	-4.5892	2.80E-06	0.0004482	CYP71Z6
LOC_Os03g14642	5.4500	25.7000	2.2374	2.83E-06	0.00045	-
LOC_Os06g41770	25.5733	11.0233	-1.2141	2.85E-06	0.00045	BZIP60
LOC_Os05g07450	0.8033	0.0067	-6.9129	2.87E-06	0.000452	At2g19130
LOC_Os06g37070	48.0033	15.7800	-1.6050	2.90E-06	0.0004532	Tmem258
LOC_Os12g36850	135.6733	9.5300	-3.8315	3.15E-06	0.0004887	BETV1D
LOC_Os04g39180	0.1900	1.6633	3.1300	3.16E-06	0.0004887	At2g42960
LOC_Os09g20460	10.6533	2.8533	-1.9006	3.27E-06	0.0005011	-
LOC_Os05g01570	5.6000	1.8667	-1.5850	3.28E-06	0.0005011	At5g64700
LOC_Os11g02530	3.6467	0.4233	-3.1067	3.35E-06	0.000507	WRKY70
LOC_Os02g36190	7.3933	0.5967	-3.6312	3.38E-06	0.000507	CYP71Z7
LOC_Os02g49480	20.8233	5.7967	-1.8449	3.39E-06	0.000507	BIM1
LOC_Os06g09920	4.8933	12.0400	1.2989	3.39E-06	0.000507	-
LOC_Os08g39840	128.2300	11.1533	-3.5232	3.54E-06	0.0005253	CM-LOX1
LOC_Os12g27254	5.7400	0.9100	-2.6571	3.63E-06	0.0005367	HSR201
LOC_Os10g37570	3.3433	0.1967	-4.0875	3.78E-06	0.0005551	-
LOC_Os05g05030	9.5033	2.6133	-1.8625	3.93E-06	0.0005746	-
LOC_Os07g48050	10.2867	1.0400	-3.3061	4.00E-06	0.000581	-
MSTRG.15315	46.5367	17.9900	-1.3712	4.02E-06	0.000581	--
LOC_Os06g20790	4.4933	0.6633	-2.7600	4.06E-06	0.0005846	AAMT1I
LOC_Os04g29770	15.8367	1.9400	-3.0291	4.29E-06	0.0006142	WAK3
LOC_Os07g44890	17.6833	5.9533	-1.5706	4.34E-06	0.000617	CXE18
LOC_Os07g18230	3.0500	0.5533	-2.4626	4.38E-06	0.0006181	LECRK41
LOC_Os01g15830	3.6033	0.4467	-3.0121	4.39E-06	0.0006181	PER72
LOC_Os09g34160	5.3833	0.9133	-2.5593	4.41E-06	0.0006181	RPM1
LOC_Os12g01590	23.1500	50.2833	1.1191	4.73E-06	0.00066	-
LOC_Os02g40320	0.2533	1.0933	2.1096	4.97E-06	0.0006879	MEL1
LOC_Os10g39750	1.4400	0.0867	-4.0544	4.99E-06	0.0006879	BHLH93
LOC_Os02g41670	6.2500	0.3000	-4.3808	5.13E-06	0.0007046	ZB8
LOC_Os01g10110	3.1267	0.1533	-4.3499	5.43E-06	0.0007422	CKX2
LOC_Os11g02130	0.7567	3.2700	2.1116	5.59E-06	0.000759	GSVIVT00023967001
MSTRG.19622	29.6233	13.4100	-1.1434	5.92E-06	0.0008008	--
LOC_Os08g44210	17.0833	6.7533	-1.3389	6.65E-06	0.0008953	FOLB2
LOC_Os07g30590	17.1000	0.7900	-4.4360	6.86E-06	0.0009185	LTPL1
LOC_Os06g16640	41.8500	2.2500	-4.2172	6.91E-06	0.0009195	-
LOC_Os12g32814	3.5867	0.4800	-2.9015	6.94E-06	0.0009195	ABCG50
LOC_Os10g30410	5.3200	0.6933	-2.9398	6.97E-06	0.00092	CYP71Z6
LOC_Os11g04600	1.2733	0.1300	-3.2920	7.14E-06	0.0009379	NPR6
LOC_Os02g39660	9.7733	1.3500	-2.8559	7.21E-06	0.0009419	EFR
LOC_Os11g48000	2.7167	0.4200	-2.6934	7.30E-06	0.0009473	RBE
LOC_Os10g04570	3.2467	0.8000	-2.0209	7.32E-06	0.0009473	-
LOC_Os01g71700	3.2833	0.3233	-3.3441	7.62E-06	0.0009723	BAT1
LOC_Os01g40499	4.6333	1.5633	-1.5674	7.62E-06	0.0009723	At2g19130

LOC_Os07g33780	109.2567	16.2967	-2.7451	7.62E-06	0.0009723	ABCG43
LOC_Os08g02900	8.2733	21.6467	1.3876	8.31E-06	0.0010545	-
LOC_Os04g44354	6.5667	1.3500	-2.2822	8.98E-06	0.0011342	UGT82A1
LOC_Os09g39190	1.9233	0.3467	-2.4720	9.31E-06	0.0011712	RGLG1
LOC_Os07g03040	9.1733	1.1267	-3.0254	9.40E-06	0.0011742	-
LOC_Os11g46810	0.9633	0.1067	-3.1749	9.42E-06	0.0011742	WAK1
LOC_Os06g20920	4.3033	0.8467	-2.3456	9.49E-06	0.0011777	AAMT1
LOC_Os03g60850	18.3067	6.5933	-1.4733	9.78E-06	0.001202	NPF5.7
LOC_Os11g29840	1.7833	0.3000	-2.5715	1.00E-05	0.0012253	-
LOC_Os02g15810	0.7133	0.0400	-4.1565	1.09E-05	0.0013219	HMGB13
LOC_Os09g26960	49.0367	7.0367	-2.8009	1.10E-05	0.0013328	CYP75B1
LOC_Os04g03830	5.6733	0.6367	-3.1556	1.12E-05	0.0013538	WAK5
LOC_Os12g35940	1.6500	4.1000	1.3132	1.13E-05	0.0013538	-
LOC_Os08g04540	8.5433	0.4633	-4.2047	1.13E-05	0.0013547	TDC
LOC_Os06g46160	1.7400	0.1000	-4.1210	1.17E-05	0.0013979	-
LOC_Os03g47810	5.9467	0.5733	-3.3746	1.18E-05	0.0014015	NFD4
LOC_Os07g48790	0.4833	0.0010	-8.9169	1.20E-05	0.0014198	KINB1
LOC_Os02g36030	1.7467	0.1633	-3.4187	1.23E-05	0.0014518	CYP76M5
LOC_Os05g33900	4.1233	0.5900	-2.8050	1.27E-05	0.0014863	At5g07050
LOC_Os03g28260	20.5400	5.5200	-1.8957	1.28E-05	0.0014863	LEA5-A
LOC_Os02g18080	5.2733	1.0667	-2.3056	1.32E-05	0.0015292	RGA2
LOC_Os04g45590	1.2733	0.1267	-3.3295	1.32E-05	0.0015292	-
LOC_Os12g11660	7.7200	1.3333	-2.5336	1.35E-05	0.0015505	-
LOC_Os08g15149	32.9633	4.3333	-2.9273	1.41E-05	0.0016172	-
LOC_Os11g33394	2.8300	0.7467	-1.9223	1.42E-05	0.0016207	At3g47200
LOC_Os04g10010	8.6633	0.4733	-4.1940	1.43E-05	0.0016255	Os04g0179200
LOC_Os12g14150	1.0667	3.9400	1.8851	1.46E-05	0.0016542	MED15A
LOC_Os04g10924	4.4400	1.0333	-2.1033	1.47E-05	0.001659	-
LOC_Os02g01100	12.8167	39.6333	1.6287	1.48E-05	0.0016648	AVT1
LOC_Os07g34070	8.0133	1.8833	-2.0891	1.54E-05	0.0017153	At5g64700
LOC_Os05g47870	7.3800	17.5533	1.2501	1.54E-05	0.0017175	-
MSTRG.3494	20.7933	10.2133	-1.0257	1.64E-05	0.0018157	RPL5B
MSTRG.9499	38.3567	77.6300	1.0171	1.66E-05	0.0018255	--
LOC_Os06g09900	447.4700	914.1733	1.0307	1.66E-05	0.0018255	-
LOC_Os02g49720	8.5200	18.7000	1.1341	1.72E-05	0.0018785	ALDH2B7
LOC_Os07g41060	7.8133	0.8800	-3.1504	1.72E-05	0.0018785	DFR
LOC_Os08g39500	25.5967	9.8100	-1.3836	1.77E-05	0.0019199	RPL31
LOC_Os09g26999	2.2800	0.2967	-2.9421	1.78E-05	0.0019265	GG3
LOC_Os07g42310	14.8433	5.7367	-1.3715	1.84E-05	0.0019755	tmem87a
LOC_Os03g59770	3.0533	0.5733	-2.4129	1.85E-05	0.0019755	-
LOC_Os12g41650	18.9433	39.6733	1.0665	1.91E-05	0.0020284	PIF1
LOC_Os06g03580	104.0867	28.7000	-1.8587	1.91E-05	0.0020284	BB
LOC_Os04g03920	12.7500	2.0433	-2.6415	1.93E-05	0.0020405	-

LOC_Os09g29540	12.1167	1.8533	-2.7088	2.01E-05	0.002116	WAK5
LOC_Os04g15920	13.6033	1.2100	-3.4909	2.02E-05	0.0021196	CAD6
LOC_Os12g26470	0.5933	0.0010	-9.2127	2.20E-05	0.0022869	nep1
LOC_Os03g08460	12.3467	2.0200	-2.6117	2.20E-05	0.0022869	ERF073
LOC_Os05g01350	1.1233	2.9200	1.3782	2.42E-05	0.0025094	FH14
LOC_Os11g44380	30.2500	13.5300	-1.1608	2.49E-05	0.0025695	-
LOC_Os06g39120	74.8200	22.1433	-1.7566	2.55E-05	0.002625	-
LOC_Os04g58090	9.4867	1.2767	-2.8935	2.62E-05	0.0026868	YLS9
LOC_Os09g09650	2.3967	0.3767	-2.6697	2.65E-05	0.0027009	-
LOC_Os11g38640	0.0400	0.4067	3.3458	2.67E-05	0.0027151	-
LOC_Os02g32814	76.9700	221.9400	1.5278	2.70E-05	0.0027324	SLAH3
LOC_Os11g40850	10.6167	0.6500	-4.0297	2.76E-05	0.0027882	-
LOC_Os04g30330	1.5500	0.1067	-3.8611	2.77E-05	0.0027882	WAKL2
LOC_Os12g08930	7.8767	0.7467	-3.3990	2.90E-05	0.0028942	-
LOC_Os05g32170	4.7133	9.9600	1.0794	2.98E-05	0.002961	At3g51990
LOC_Os01g26210	1.4467	0.2700	-2.4217	3.01E-05	0.0029858	WAK3
LOC_Os05g03460	1.5400	3.7900	1.2993	3.04E-05	0.0030034	At3g07070
LOC_Os08g26850	12.6700	4.3533	-1.5412	3.25E-05	0.0031956	-
LOC_Os06g06560	1.6733	5.2733	1.6560	3.37E-05	0.0032953	Os06g0160700
LOC_Os07g04560	8.2867	0.7333	-3.4983	3.38E-05	0.0032953	JUB1
LOC_Os04g22120	15.7333	2.3800	-2.7248	3.45E-05	0.0033365	LECRK91
LOC_Os01g56080	5.4967	1.1400	-2.2695	3.45E-05	0.0033365	-
LOC_Os03g25490	0.4167	1.7467	2.0676	3.45E-05	0.0033365	CYP734A1
LOC_Os10g33760	2.9633	0.5133	-2.5293	3.60E-05	0.003453	NAC021
LOC_Os11g05290	2.1633	0.3300	-2.7127	3.60E-05	0.003453	At3g17210
LOC_Os10g05250	6.9167	1.3200	-2.3895	3.70E-05	0.0035218	WAKL4
LOC_Os09g04310	25.9767	5.9267	-2.1319	3.70E-05	0.0035218	-
MSTRG.19461	1.4267	0.0900	-3.9866	3.79E-05	0.0035876	WRKY55
LOC_Os03g04060	110.9533	20.6167	-2.4281	3.79E-05	0.0035876	Cht11
LOC_Os09g32100	1.3733	0.2433	-2.4967	3.92E-05	0.0036938	-
LOC_Os01g16180	22.3400	53.3633	1.2562	3.96E-05	0.0037201	EMB1691
LOC_Os07g02260	1.2633	0.1000	-3.6592	4.02E-05	0.0037618	-
LOC_Os01g02680	0.1667	0.0010	-7.3808	4.04E-05	0.0037702	At1g67000
LOC_Os11g38520	3.6933	1.1367	-1.7001	4.19E-05	0.0038907	RPM1
LOC_Os01g47580	1.0433	0.1333	-2.9681	4.27E-05	0.0039549	LPP2
LOC_Os08g10150	1.3200	0.2433	-2.4395	4.29E-05	0.0039549	At1g56130
LOC_Os08g42570	1.4467	0.1267	-3.5136	4.37E-05	0.0040236	At3g47200
LOC_Os10g34940	11.1233	0.9467	-3.5546	4.39E-05	0.0040242	-
LOC_Os03g57690	37.3033	80.5900	1.1113	4.41E-05	0.0040275	Os03g0790900
LOC_Os11g05614	3.5133	0.9900	-1.8273	4.46E-05	0.0040545	NAC090
LOC_Os06g05410	20.4733	4.7567	-2.1057	4.47E-05	0.0040545	-
MSTRG.24830	1.3433	0.0467	-4.8473	4.53E-05	0.0040985	--
LOC_Os05g48210	35.4033	14.0833	-1.3299	4.58E-05	0.0041252	-

LOC_Os04g58220	2.9500	0.3133	-3.2349	4.60E-05	0.0041283	PLT4
LOC_Os04g30250	32.5500	8.3767	-1.9582	4.61E-05	0.0041283	WAK2
LOC_Os01g03940	2.7533	0.6500	-2.0827	4.63E-05	0.0041298	-
LOC_Os01g73200	124.5500	7.7600	-4.0045	4.72E-05	0.0041886	Sb03g046810
LOC_Os01g02130	21.7067	5.0333	-2.1086	4.72E-05	0.0041886	-
LOC_Os04g05650	188.8000	41.3900	-2.1895	4.92E-05	0.004349	-
LOC_Os03g13250	4.2467	9.6433	1.1832	5.12E-05	0.0045105	NPF8.3
LOC_Os08g35490	1.1100	8.0567	2.8596	5.21E-05	0.0045722	COPT5
LOC_Os06g38660	173.4533	21.3600	-3.0216	5.27E-05	0.004611	-
LOC_Os02g17330	30.1233	63.4333	1.0744	5.37E-05	0.0046806	DAAT
LOC_Os02g48870	28.8033	5.6767	-2.3431	5.39E-05	0.0046846	ASPG1
LOC_Os01g48620	5.5467	1.0633	-2.3830	5.45E-05	0.0047174	-
LOC_Os04g33200	8.5033	21.7833	1.3571	5.46E-05	0.0047174	Hgsnat
LOC_Os08g09830	3.2433	1.2133	-1.4185	5.52E-05	0.004751	BPM1
LOC_Os06g34730	9.6300	1.4000	-2.7821	5.66E-05	0.0048598	-
LOC_Os03g13400	0.2467	0.0010	-7.9464	5.87E-05	0.0050092	IDD14
LOC_Os01g09990	4.3167	0.2567	-4.0719	5.87E-05	0.0050092	BHLH
LOC_Os02g26210	15.9233	5.1300	-1.6341	5.95E-05	0.0050586	Os02g0460200
LOC_Os01g49320	72.0833	25.4733	-1.5007	6.04E-05	0.0051242	-
LOC_Os01g63710	4.2400	1.9767	-1.1010	6.09E-05	0.0051422	CDC6
LOC_Os10g17940	1.1433	0.1433	-2.9958	6.17E-05	0.0051941	-
LOC_Os01g23580	3.0867	0.2467	-3.6454	6.20E-05	0.0052094	-
LOC_Os09g04320	1.8133	0.2400	-2.9175	6.30E-05	0.005248	-
LOC_Os11g28540	3.2300	0.3000	-3.4285	6.33E-05	0.005248	-
LOC_Os01g72150	2.5567	0.7300	-1.8083	6.34E-05	0.005248	-
LOC_Os04g44950	20.9100	7.0700	-1.5644	6.35E-05	0.005248	SALR
LOC_Os07g01560	104.2367	24.9167	-2.0647	6.35E-05	0.005248	STP1
LOC_Os02g36280	3.1833	0.3033	-3.3916	6.38E-05	0.0052568	CYP76M6
LOC_Os12g16520	4.8300	1.7100	-1.4980	6.53E-05	0.0053554	WAK2
LOC_Os10g10175	5.5633	16.2867	1.5497	6.54E-05	0.0053554	-
LOC_Os07g28850	15.6067	2.9567	-2.4001	6.82E-05	0.0055542	AGO18
MSTRG.4699	1.9367	0.2733	-2.8248	6.85E-05	0.0055542	--
LOC_Os08g38990	14.8500	41.9100	1.4968	6.88E-05	0.0055542	WRKY2
LOC_Os06g32890	0.9433	0.1833	-2.3633	6.88E-05	0.0055542	Tf2-6
LOC_Os04g25060	31.9067	3.6333	-3.1345	6.99E-05	0.0056232	CRRSP38
LOC_Os01g61180	10.4000	21.8533	1.0713	7.07E-05	0.0056729	EXO70B1
LOC_Os06g43980	0.6133	2.1233	1.7916	7.31E-05	0.0058293	-
LOC_Os05g50580	2.8100	0.4200	-2.7421	7.38E-05	0.0058665	SCPL50
LOC_Os01g61510	9.0833	1.2033	-2.9162	7.47E-05	0.0059206	AMT2-3
LOC_Os10g23310	4.9333	0.3367	-3.8732	7.59E-05	0.006004	ACT-1
MSTRG.5362	0.6867	0.0010	-9.4235	7.64E-05	0.0060228	--
LOC_Os06g01972	6.5600	1.1267	-2.5416	7.71E-05	0.006064	At3g30340
LOC_Os02g07820	7.2867	2.5867	-1.4942	7.86E-05	0.0061606	-

LOC_Os01g07370	56.6500	19.4500	-1.5423	7.90E-05	0.0061718	NET4B
LOC_Os04g06520	79.3767	14.0700	-2.4961	8.16E-05	0.006363	-
LOC_Os12g11510	0.1833	0.0010	-7.5183	8.40E-05	0.0065311	RLP12
LOC_Os08g13420	0.6100	0.1233	-2.3062	8.50E-05	0.0065792	RLK1
LOC_Os01g48360	26.9267	10.4167	-1.3701	8.52E-05	0.0065792	-
LOC_Os03g01580	4.1500	12.1200	1.5462	8.55E-05	0.0065802	-
LOC_Os07g07300	17.8700	5.2567	-1.7653	8.57E-05	0.0065802	-
LOC_Os01g53040	5.7000	1.3167	-2.1141	8.60E-05	0.0065802	WRKY65
LOC_Os05g40630	2.9967	0.8167	-1.8755	8.61E-05	0.0065802	At2g38640
LOC_Os05g40960	20.7200	3.1700	-2.7085	8.70E-05	0.0066248	APL
LOC_Os12g35350	1.5167	3.2033	1.0787	8.79E-05	0.0066746	ACBP4
LOC_Os10g41999	12.1233	5.0467	-1.2644	8.95E-05	0.0067768	-
LOC_Os01g57340	4.8600	2.0400	-1.2524	8.98E-05	0.0067863	RGA2
LOC_Os12g04440	14.8200	33.2033	1.1638	9.20E-05	0.0069133	IPMSA
LOC_Os04g29790	3.0733	0.4300	-2.8374	9.29E-05	0.0069605	-
LOC_Os07g46846	14.2400	1.7333	-3.0383	9.42E-05	0.0070366	SDR5
LOC_Os10g05020	6.3500	1.2667	-2.3257	9.46E-05	0.0070456	CYP89A2
LOC_Os12g02980	45.8167	16.6467	-1.4606	9.49E-05	0.0070483	APY3
LOC_Os07g33690	2.5233	0.6567	-1.9421	9.57E-05	0.0070652	RGA2
LOC_Os06g35940	17.0133	2.6300	-2.6935	9.58E-05	0.0070652	HD3A
LOC_Os07g46930	0.7533	0.0133	-5.8202	9.59E-05	0.0070652	-
LOC_Os07g42324	4.8500	1.6533	-1.5526	9.69E-05	0.0071036	-
LOC_Os10g02380	15.7500	38.7200	1.2977	9.69E-05	0.0071036	Os10g0113000
LOC_Os04g52190	44.0767	6.2800	-2.8112	9.73E-05	0.0071128	VSR7
LOC_Os11g44560	5.1667	1.7433	-1.5674	9.91E-05	0.0072226	CCR4
LOC_Os10g03540	9.3100	4.2333	-1.1370	0.0001006	0.0073159	nhp2l1
LOC_Os07g02970	3.6567	0.9400	-1.9598	0.0001019	0.0073882	-
LOC_Os06g36650	0.1633	0.6300	1.9475	0.0001024	0.0073882	ABCC8
LOC_Os01g66840	9.7200	0.6200	-3.9706	0.0001025	0.0073882	PAE5
LOC_Os02g53510	18.5933	7.0300	-1.4032	0.0001038	0.0074683	-
LOC_Os01g39330	15.7967	4.7000	-1.7489	0.000106	0.0075914	BHLH
LOC_Os04g34320	3.0933	1.0133	-1.6101	0.0001061	0.0075914	At2g19130
LOC_Os09g13470	5.1133	1.6933	-1.5944	0.0001081	0.0077165	-
LOC_Os01g42650	14.8133	6.4533	-1.1988	0.0001089	0.007751	COX5B-2
LOC_Os05g05020	6.3733	1.1133	-2.5172	0.0001095	0.0077729	-
LOC_Os01g58500	3.6900	1.1433	-1.6904	0.0001106	0.0078324	-
LOC_Os01g73940	256.5133	117.7667	-1.1231	0.0001116	0.0078766	-
LOC_Os11g07460	2.4700	0.7000	-1.8191	0.0001127	0.0079222	PCF3
LOC_Os04g41900	1.3767	0.2600	-2.4046	0.0001128	0.0079222	-
LOC_Os08g07080	7.7633	1.4633	-2.4074	0.0001134	0.007946	TPS2
LOC_Os03g49550	0.4700	1.6433	1.8059	0.0001157	0.0080828	UGT91B1
LOC_Os11g31090	1.2733	5.4633	2.1012	0.0001161	0.0080929	HHT1
LOC_Os04g39290	2.9967	0.5300	-2.4993	0.0001168	0.0081165	-

LOC_Os12g07310	10.0700	1.4000	-2.8466	0.0001187	0.0082103	CBP
LOC_Os04g56930	0.4267	0.0100	-5.4150	0.0001208	0.0083144	CIN5
LOC_Os04g07260	2.8033	0.3033	-3.2082	0.0001208	0.0083144	-
LOC_Os02g09980	3.8300	1.3200	-1.5368	0.0001214	0.0083318	-
LOC_Os09g29200	373.9200	163.0367	-1.1975	0.000122	0.0083496	-
LOC_Os03g20210	2.7900	0.3500	-2.9948	0.000124	0.0084513	nep1
LOC_Os09g32120	0.2367	0.0010	-7.8867	0.0001241	0.0084513	-
LOC_Os04g10000	5.6233	0.5633	-3.3194	0.0001253	0.008492	Os04g0179200
LOC_Os12g03640	2.4667	0.6033	-2.0315	0.0001264	0.0085454	At1g34300
LOC_Os06g14406	2.7267	19.1933	2.8154	0.0001268	0.0085519	SYD
LOC_Os07g14820	1.1467	0.0300	-5.2563	0.0001271	0.0085525	At3g47570
LOC_Os01g11660	5.3167	2.0667	-1.3632	0.0001277	0.0085657	At1g28590
LOC_Os02g33330	48.7533	12.5567	-1.9570	0.000128	0.0085702	-
LOC_Os03g56500	4.1567	1.1133	-1.9005	0.0001289	0.0086087	-
LOC_Os09g16950	13.4267	2.6700	-2.3302	0.0001294	0.0086183	LECRK91
LOC_Os10g40710	0.9100	0.0300	-4.9228	0.0001306	0.0086561	EXPB2
LOC_Os11g44680	19.0633	5.8100	-1.7142	0.0001321	0.0087321	CBP60B
LOC_Os12g31540	3.5533	0.3367	-3.3998	0.0001328	0.0087557	CNR13
LOC_Os03g52720	19.4133	3.4733	-2.4827	0.0001355	0.008918	MDP1
LOC_Os07g31190	1.9167	0.1700	-3.4950	0.0001376	0.0089925	WAK2
LOC_Os01g41430	8.4867	1.0300	-3.0426	0.0001377	0.0089925	TOGT1
LOC_Os08g07890	4.1067	0.8200	-2.3243	0.0001378	0.0089925	SERK1
LOC_Os02g43700	82.0367	39.5267	-1.0534	0.000138	0.0089925	At4g16820
LOC_Os03g10030	10.8600	2.9233	-1.8933	0.0001384	0.0089971	Os03g0196400
LOC_Os09g25070	37.3600	8.5467	-2.1281	0.0001438	0.009285	WRKY40
LOC_Os02g42190	8.1067	1.2467	-2.7010	0.0001444	0.009295	WAK4
LOC_Os05g03610	19.0400	5.5267	-1.7846	0.000145	0.0093166	PLC2
LOC_Os06g39880	1.3233	0.1400	-3.2407	0.0001464	0.0093816	CYP734A4
LOC_Os01g03680	1.7800	0.2500	-2.8319	0.0001497	0.0095251	RBBI3.3
LOC_Os01g32460	63.1567	12.0833	-2.3859	0.0001498	0.0095251	-
LOC_Os11g04954	11.3233	4.8300	-1.2292	0.0001498	0.0095251	LIM15
LOC_Os10g26500	1.0233	0.1433	-2.8358	0.0001501	0.0095251	HOX23
LOC_Os10g35950	58.1167	4.7300	-3.6190	0.0001505	0.0095301	HSR201
LOC_Os07g44440	14.3067	29.0633	1.0225	0.0001565	0.009873	Os07g0638400
LOC_Os10g42130	21.4167	2.9867	-2.8421	0.0001566	0.009873	NAC086
LOC_Os03g04310	0.7233	0.0867	-3.0611	0.0001619	0.0101469	BHLH93
LOC_Os01g40260	40.1067	8.1300	-2.3025	0.0001619	0.0101469	WRKY50
LOC_Os10g20350	10.7500	2.4667	-2.1237	0.0001621	0.0101469	DTXL1
LOC_Os07g03170	13.9300	2.8167	-2.3061	0.0001645	0.0102642	-
LOC_Os01g73780	13.7933	5.3600	-1.3637	0.0001647	0.0102642	-
LOC_Os01g14630	8.6833	1.4333	-2.5989	0.0001655	0.0102881	GGPPS1
LOC_Os07g10910	1.8467	5.5100	1.5771	0.0001701	0.010552	EXO70A1
LOC_Os01g11860	2.9733	0.7233	-2.0393	0.0001708	0.0105702	DJ1B

LOC_Os08g07100	0.8867	0.0567	-3.9678	0.0001768	0.0108944	TPS2
MSTRG.22410	4.2767	0.3167	-3.7554	0.0001769	0.0108944	--
LOC_Os09g25390	0.1267	0.0010	-6.9849	0.0001788	0.0109863	PLDALPHA1
LOC_Os01g19840	61.1867	28.6867	-1.0928	0.0001804	0.0110292	RPL29A
LOC_Os01g15340	1.2067	0.1100	-3.4555	0.0001807	0.0110292	RAA1
LOC_Os02g04120	8.2433	2.9500	-1.4825	0.0001807	0.0110292	Os02g0134000
LOC_Os04g10530	20.0133	4.8733	-2.0380	0.0001817	0.0110651	gatA2
LOC_Os02g49660	1.7800	0.5000	-1.8319	0.0001858	0.0112652	TBL19
LOC_Os01g09190	4.4933	0.3633	-3.6284	0.0001919	0.0116087	-
LOC_Os08g27170	9.5033	2.0367	-2.2222	0.0001936	0.0116882	SARD1
LOC_Os01g66710	0.2433	0.0010	-7.9268	0.0001954	0.01177	-
LOC_Os01g71760	9.5733	1.3433	-2.8332	0.0001995	0.0119897	BAT1
LOC_Os01g52260	16.6467	39.4100	1.2433	0.0002053	0.0122567	SAT1
LOC_Os05g47750	7.9667	1.4033	-2.5051	0.0002066	0.0123041	At1g18390
LOC_Os07g47450	18.1633	4.0300	-2.1722	0.000207	0.0123046	Os07g0671000
LOC_Os10g05490	2.5600	0.2967	-3.1092	0.0002088	0.0123809	CYP76C2
LOC_Os03g46910	0.8300	0.2133	-1.9600	0.0002095	0.0123968	-
LOC_Os05g39580	1.4733	0.1067	-3.7879	0.0002102	0.0124129	PYL5
LOC_Os08g03330	0.4133	0.0010	-8.6912	0.0002109	0.0124188	-
LOC_Os04g25650	9.2067	1.2633	-2.8654	0.0002113	0.0124188	CRRSP38
LOC_Os11g39310	10.1067	22.5700	1.1591	0.0002136	0.0124692	RPM1
LOC_Os03g10100	15.7400	5.1300	-1.6174	0.000214	0.0124692	PLT5
LOC_Os01g14590	36.9767	5.4800	-2.7544	0.0002177	0.012659	-
LOC_Os07g10850	0.0667	0.0010	-6.0589	0.0002192	0.0127196	-
LOC_Os04g47150	0.1700	0.0067	-4.6724	0.0002223	0.0128723	SBT1.2
LOC_Os01g68269	51.7100	19.8767	-1.3794	0.0002246	0.0129762	-
LOC_Os01g74110	15.0667	31.4867	1.0634	0.0002282	0.0131289	ZIP1
LOC_Os07g14740	64.2267	18.7200	-1.7786	0.0002289	0.0131403	-
LOC_Os11g44340	0.8233	0.1300	-2.6630	0.0002294	0.0131403	CBP60A
LOC_Os04g44320	9.9800	2.0767	-2.2648	0.0002306	0.013185	YSL12
LOC_Os02g13580	0.1567	0.7233	2.2070	0.0002321	0.0132429	KP1
LOC_Os05g46370	0.4700	2.2000	2.2268	0.0002337	0.0133023	BHLH87
LOC_Os05g01370	2.7500	0.2600	-3.4028	0.0002362	0.0134187	PGIP
LOC_Os03g06670	2.5900	0.5933	-2.1260	0.0002429	0.0137708	Os03g0162200
LOC_Os02g50740	1.5833	0.3867	-2.0338	0.0002438	0.0137921	alr3466
LOC_Os01g47690	19.6467	43.5067	1.1470	0.0002463	0.0139047	GLY3
LOC_Os03g61500	1.0500	0.0010	-10.0362	0.0002477	0.0139554	CNR10
LOC_Os04g38790	16.2033	5.6633	-1.5166	0.0002489	0.0139909	-
LOC_Os02g58554	12.9000	47.9367	1.8938	0.0002513	0.0140722	-
LOC_Os04g42130	1.0700	2.5533	1.2548	0.0002521	0.0140766	At5g25050
LOC_Os01g70490	14.7667	2.6733	-2.4656	0.0002535	0.0141027	HAK5
LOC_Os10g36100	0.5967	0.0010	-9.2208	0.0002551	0.0141665	LTP-2
LOC_Os06g39110	166.6200	44.7967	-1.8951	0.0002587	0.0143378	-

LOC_Os07g47210	1.6333	3.8967	1.2544	0.0002688	0.014808	At2g23540
LOC_Os09g25810	2.2967	0.7100	-1.6937	0.0002689	0.014808	At4g30420
LOC_Os05g11414	0.1233	0.7433	2.5914	0.0002725	0.0149648	MADS58
LOC_Os01g16980	10.3067	3.4800	-1.5664	0.0002728	0.0149648	-
LOC_Os01g48640	7.5467	3.3600	-1.1674	0.000276	0.0150764	-
MSTRG.3380	4.0767	2.0367	-1.0012	0.0002767	0.0150831	--
LOC_Os01g24490	1.9100	0.6833	-1.4829	0.000278	0.0151259	-
LOC_Os05g05040	28.7833	5.6367	-2.3523	0.000279	0.0151509	-
LOC_Os06g09350	2.5833	6.2333	1.2708	0.0002817	0.0152652	-
LOC_Os11g02540	19.3400	3.0733	-2.6537	0.0002852	0.0154232	WRKY70
LOC_Os10g04270	20.5667	4.7733	-2.1072	0.0002882	0.0155559	GOS9
LOC_Os01g72680	0.1500	0.0010	-7.2288	0.0002892	0.0155647	RPS5
LOC_Os02g43290	1.1967	0.3400	-1.8154	0.0002895	0.0155647	At4g10390
LOC_Os09g28650	62.8367	11.7667	-2.4169	0.0002916	0.0156476	TCEA-B1
LOC_Os04g09260	4.3067	0.4133	-3.3812	0.0002967	0.0158793	AMAT
LOC_Os09g34150	5.3933	1.8133	-1.5725	0.0002971	0.0158793	RPM1
LOC_Os09g23430	4.9700	0.1533	-5.0185	0.0002991	0.0159243	-
LOC_Os07g12780	62.4700	148.7967	1.2521	0.000302	0.0160463	CYCP4-1
LOC_Os09g38350	0.6167	0.0300	-4.3615	0.0003049	0.0161537	AAE11
LOC_Os09g08130	85.6433	9.7533	-3.1344	0.0003052	0.0161537	At2g04400
LOC_Os08g36630	5.3900	1.2133	-2.1513	0.0003079	0.0162637	ACA7
LOC_Os04g33210	9.7967	22.7033	1.2125	0.0003092	0.0162991	CLPD2
LOC_Os03g47016	2.7100	0.4500	-2.5903	0.0003121	0.0164192	OSH10
LOC_Os12g01400	0.5533	0.0010	-9.1120	0.000315	0.0165113	CML25
LOC_Os08g07830	41.0933	19.5100	-1.0747	0.0003151	0.0165113	IST1
LOC_Os03g07810	0.1833	0.0010	-7.5183	0.0003157	0.0165119	-
LOC_Os05g37470	9.2700	3.7767	-1.2955	0.0003169	0.0165317	Os05g0447200
LOC_Os06g37224	0.3467	0.0200	-4.1155	0.0003173	0.0165317	CYP701A9
LOC_Os11g18194	2.0167	5.7867	1.5208	0.0003205	0.016636	Os11g0285000
LOC_Os09g14610	58.9133	16.6267	-1.8251	0.0003268	0.0169257	DCL2B
LOC_Os05g39860	0.3800	0.0167	-4.5110	0.0003273	0.0169257	-
LOC_Os09g29520	12.5133	3.5433	-1.8203	0.0003288	0.0169683	WAK3
LOC_Os12g32850	2.3933	0.3067	-2.9643	0.0003294	0.0169692	CYP71E1
LOC_Os01g29330	33.4500	5.9633	-2.4878	0.0003315	0.0170417	-
LOC_Os03g18770	80.6033	30.4600	-1.4039	0.0003346	0.0171704	WUN1
LOC_Os10g36703	49.7667	9.8333	-2.3394	0.0003371	0.0172308	SAUR32
LOC_Os09g02300	1.5867	0.5467	-1.5373	0.0003382	0.0172335	-
LOC_Os09g30454	4.1500	1.3833	-1.5850	0.0003384	0.0172335	WAK3
LOC_Os07g10050	0.8233	4.9300	2.5820	0.0003411	0.0173372	TY3B-I
LOC_Os05g38860	13.2800	3.4433	-1.9474	0.0003444	0.0174427	-
LOC_Os12g41570	2.5867	0.4400	-2.5555	0.0003513	0.0176468	WAKL14
LOC_Os09g27500	3.5667	0.4267	-3.0634	0.0003522	0.0176468	CYP76M5
LOC_Os01g03549	10.7533	4.4600	-1.2697	0.0003531	0.0176468	LPR1

MSTRG.26610	4.5267	1.4533	-1.6391	0.0003536	0.0176468	--
LOC_Os05g36140	3.1067	0.7833	-1.9877	0.0003542	0.0176468	-
LOC_Os04g43550	9.1233	3.4200	-1.4156	0.0003548	0.0176468	Dctpp1
LOC_Os04g30260	0.2300	0.0010	-7.8455	0.0003549	0.0176468	WAK2
LOC_Os06g10580	22.3133	9.2600	-1.2688	0.000355	0.0176468	CNGC20
LOC_Os07g31720	108.5300	32.3333	-1.7470	0.0003584	0.017781	CAR9
LOC_Os05g35930	5.6233	1.4500	-1.9554	0.0003612	0.0178886	-
LOC_Os12g43820	6.2900	2.4000	-1.3900	0.0003624	0.0179145	-
LOC_Os03g43990	4.3200	13.2633	1.6183	0.0003636	0.0179411	At1g30440
LOC_Os06g21950	0.0010	0.1600	7.3219	0.0003677	0.0181128	PHT1-10
LOC_Os07g34260	66.9467	6.5833	-3.3461	0.0003697	0.0181743	Os07g0271500
LOC_Os10g34440	5.2267	1.2933	-2.0148	0.0003705	0.0181743	-
LOC_Os09g15050	1.9167	0.1800	-3.4125	0.000371	0.0181743	CPS4
LOC_Os03g20440	6.7967	1.1667	-2.5424	0.0003739	0.0182819	CAMBP25
LOC_Os04g39300	39.2800	5.1667	-2.9265	0.0003765	0.0183034	-
LOC_Os08g04350	3.0467	0.2433	-3.6462	0.0003766	0.0183034	-
LOC_Os01g55020	6.3333	2.2567	-1.4888	0.000377	0.0183034	NFD6
LOC_Os01g39020	2.4467	8.7167	1.8330	0.0003803	0.0184296	HSFA6B
LOC_Os06g38340	9.4233	2.7533	-1.7751	0.0003819	0.0184604	At3g47570
LOC_Os11g37960	11.2900	2.2467	-2.3292	0.0003823	0.0184604	PR4A
LOC_Os02g42150	14.1433	2.1933	-2.6889	0.0003855	0.0185504	WAK5
LOC_Os09g22000	2.8900	6.7133	1.2160	0.0003862	0.0185505	yidA
LOC_Os11g11970	56.6733	11.5467	-2.2952	0.0003939	0.0188883	-
LOC_Os03g61540	14.7467	29.6400	1.0072	0.0003993	0.0190615	-
LOC_Os04g29580	1.1233	0.1667	-2.7527	0.0004004	0.0190615	WAK2
LOC_Os11g30760	39.9733	9.5600	-2.0640	0.0004106	0.0195148	-
LOC_Os05g10370	10.6400	4.2967	-1.3082	0.0004133	0.0196089	APS1
LOC_Os01g72130	16.9167	4.8800	-1.7935	0.0004176	0.0197057	-
LOC_Os07g42160	8.3933	3.5733	-1.2320	0.0004176	0.0197057	RSH3
LOC_Os02g36000	5.9100	1.3133	-2.1699	0.0004188	0.0197057	-
LOC_Os06g37410	2.7033	0.3200	-3.0786	0.0004196	0.0197057	BHLH
LOC_Os05g11320	5.2600	0.6333	-3.0540	0.0004197	0.0197057	MT3B
LOC_Os05g48010	25.8333	7.3733	-1.8088	0.0004231	0.0198282	RAX3
LOC_Os08g37930	13.7500	3.4800	-1.9823	0.0004251	0.01989	CjBAp12
LOC_Os06g50920	3.0400	6.3933	1.0725	0.000427	0.0199423	HT1
LOC_Os07g09800	85.4467	210.4767	1.3006	0.0004282	0.0199664	ndhS
MSTRG.7332	1.2367	0.1933	-2.6773	0.0004311	0.0200639	WAK4
LOC_Os11g35450	1.0633	0.1200	-3.1475	0.0004325	0.0200928	GSO2
LOC_Os10g39680	160.0433	50.7700	-1.6564	0.0004332	0.0200928	Cht8
LOC_Os09g25720	1.4767	0.1167	-3.6619	0.0004351	0.0201465	-
LOC_Os05g30500	47.6833	10.4433	-2.1909	0.0004397	0.0202919	-
LOC_Os02g56014	76.1633	35.2567	-1.1112	0.0004397	0.0202919	RPS30A
LOC_Os11g23080	1.8733	0.5333	-1.8125	0.0004409	0.0203121	-

LOC_Os04g55710	229.4100	77.9100	-1.5580	0.0004432	0.0203484	At3g16150
LOC_Os04g28250	4.3567	0.5300	-3.0392	0.0004434	0.0203484	Os04g0350100
MSTRG.17789	0.3667	1.4633	1.9967	0.000444	0.0203484	--
LOC_Os03g40670	8.3500	2.6867	-1.6360	0.000448	0.0204971	GDPD6
LOC_Os05g11910	27.5367	68.1067	1.3064	0.0004494	0.0205278	At5g45910
LOC_Os02g17600	0.9333	0.0633	-3.8814	0.000457	0.0208141	FT1
LOC_Os04g15630	0.1700	0.0010	-7.4094	0.0004578	0.0208141	At3g47570
LOC_Os12g25660	3.1967	0.2800	-3.5131	0.000458	0.0208141	CYP94B3
LOC_Os11g39320	2.1733	4.5567	1.0681	0.0004637	0.0210041	RPM1
LOC_Os01g12210	3.1700	0.6233	-2.3464	0.000465	0.0210267	ALMT9
LOC_Os12g38770	0.6200	0.0333	-4.2172	0.0004688	0.0211624	PAP1
LOC_Os02g02120	19.2733	4.8800	-1.9817	0.0004696	0.0211628	WAK5
LOC_Os01g55610	0.7167	0.1700	-2.0758	0.000484	0.0217422	NPF1.2
LOC_Os04g15690	82.4667	23.5100	-1.8105	0.0004898	0.0219661	-
LOC_Os02g36110	8.3467	0.6867	-3.6035	0.0004927	0.0220603	CYP76M7
LOC_Os05g05080	3.0333	0.5400	-2.4899	0.0004937	0.0220682	-
LOC_Os05g33160	0.3967	0.0133	-4.8948	0.0004996	0.022259	At1g27190
LOC_Os05g48340	27.9433	60.0100	1.1027	0.0005064	0.0224856	-
LOC_Os01g31870	30.1633	61.3433	1.0241	0.0005198	0.0229685	NRAMP4
LOC_Os02g56370	5.8333	1.2133	-2.2653	0.0005241	0.0231201	WAK5
LOC_Os04g30240	26.7000	6.9033	-1.9515	0.000528	0.0231844	WAK2
MSTRG.14495	2.5233	0.8700	-1.5362	0.0005281	0.0231844	--
LOC_Os08g09610	10.3333	3.7133	-1.4765	0.0005296	0.0232116	-
LOC_Os03g47610	223.9567	513.7200	1.1978	0.0005331	0.023328	THIC
LOC_Os12g32590	0.7433	2.0067	1.4327	0.000541	0.0236355	RPM1
LOC_Os04g32480	14.0967	2.1500	-2.7129	0.0005433	0.0236925	TIFY9
LOC_Os04g47810	2.3333	0.5833	-2.0000	0.0005441	0.0236925	BHLH111
LOC_Os11g42930	0.4967	0.1000	-2.3123	0.0005465	0.023761	-
LOC_Os12g06060	1.5733	0.5167	-1.6065	0.0005539	0.0240253	CAT6
LOC_Os02g50450	1.6667	0.2900	-2.5228	0.000555	0.0240253	-
LOC_Os06g05284	0.4900	0.0500	-3.2928	0.0005553	0.0240253	-
LOC_Os01g49614	12.4600	4.6967	-1.4076	0.0005563	0.0240299	At1g67000
LOC_Os01g16160	0.0010	0.1133	6.8244	0.000572	0.0246288	-
LOC_Os11g43620	9.1567	3.2200	-1.5078	0.0005754	0.0247395	At4g30220
ChrSy.fgenesh.gene	0.0010	0.1100	6.7814	0.0005804	0.0249132	-
LOC_Os07g03458	3.0467	0.2267	-3.7486	0.0005832	0.0249852	-
LOC_Os03g03790	11.5367	1.3900	-3.0531	0.0005841	0.0249852	AAE12
LOC_Os01g50720	7.3133	1.5800	-2.2106	0.0005864	0.0249852	MYB2
LOC_Os09g39500	74.6267	35.2100	-1.0837	0.0005866	0.0249852	Ub-CEP52-2
LOC_Os02g46810	8.5733	3.4400	-1.3174	0.0005867	0.0249852	AAP4
LOC_Os01g38530	56.9933	115.2467	1.0159	0.0005939	0.025213	ELF3
LOC_Os06g38780	6.3733	0.9933	-2.6817	0.0005953	0.025231	At3g47570
LOC_Os06g27910	3.4133	1.3367	-1.3525	0.0005976	0.0252887	At3g01570

LOC_Os07g32060	5.1533	1.4967	-1.7838	0.0006031	0.0254843	RhGT1
LOC_Os10g40600	25.2667	50.7800	1.0070	0.0006044	0.0254959	NPF6.3
LOC_Os04g30490	17.5400	4.5533	-1.9457	0.000609	0.0255955	DTXL1
LOC_Os12g43340	15.8033	6.9433	-1.1865	0.0006096	0.0255955	ADF11
LOC_Os02g34740	2.7300	1.2200	-1.1620	0.0006211	0.0260408	-
LOC_Os11g04560	7.1067	1.6600	-2.0980	0.0006285	0.0263094	CML36
LOC_Os12g01030	24.6800	7.8767	-1.6477	0.0006398	0.0266991	CSE
LOC_Os09g10980	0.5967	0.0233	-4.6765	0.000645	0.0268735	WIP5
LOC_Os02g51110	16.9567	6.2267	-1.4453	0.0006469	0.0268735	NIP2-1
LOC_Os02g36780	1.7033	3.9533	1.2147	0.0006553	0.0271355	-
LOC_Os11g01040	20.4700	6.5667	-1.6403	0.0006636	0.0273979	CSE
LOC_Os11g06570	0.2600	0.0233	-3.4780	0.000669	0.0275764	SALT
LOC_Os11g12340	39.3133	6.5100	-2.5943	0.0006708	0.0275774	RPM1
LOC_Os07g03710	63.1533	8.1400	-2.9558	0.000671	0.0275774	-
LOC_Os07g03900	1.2133	0.0800	-3.9228	0.0006856	0.0280093	LECRK41
LOC_Os06g33970	15.4500	3.5767	-2.1109	0.0006857	0.0280093	-
LOC_Os08g34720	36.5867	7.7033	-2.2478	0.0006948	0.0283379	PGDH2
LOC_Os11g44860	1.6967	0.2767	-2.6165	0.000696	0.028344	At1g67520
LOC_Os07g03368	3.0700	0.2433	-3.6572	0.000697	0.0283447	-
LOC_Os04g02030	1.0633	0.2800	-1.9251	0.000703	0.0285172	RGA2
LOC_Os07g37400	1.5900	0.2500	-2.6690	0.0007034	0.0285172	At4g05010
LOC_Os11g28470	0.5900	0.1467	-2.0082	0.0007068	0.0286057	-
LOC_Os10g25140	1.6067	4.9367	1.6195	0.0007077	0.0286057	-
LOC_Os08g06170	3.4933	0.8267	-2.0792	0.0007096	0.0286057	BBE1
LOC_Os01g44960	1.4033	4.6967	1.7428	0.0007099	0.0286057	ephx3
LOC_Os04g30010	1.5067	0.2833	-2.4108	0.0007114	0.0286057	WAK2
LOC_Os06g46340	6.9033	1.0533	-2.7123	0.0007162	0.0287066	Os06g0675700
LOC_Os07g46920	3.3667	0.5767	-2.5455	0.0007212	0.0287169	-
LOC_Os06g06850	0.8900	2.9900	1.7483	0.0007216	0.0287169	RPP13
LOC_Os11g02520	7.9700	1.4733	-2.4355	0.0007221	0.0287169	WRKY46
MSTRG.23570	6.4133	2.1167	-1.5993	0.0007331	0.0290676	--
LOC_Os05g50340	12.0133	1.3967	-3.1046	0.000735	0.0290992	RL6
LOC_Os02g52910	0.4067	0.0600	-2.7608	0.0007388	0.0291919	-
LOC_Os11g08750	0.5100	1.5967	1.6465	0.0007395	0.0291919	PECS-2.1
LOC_Os11g10120	7.9167	3.7100	-1.0935	0.0007471	0.0294063	RGA2
LOC_Os01g29070	0.0010	0.2200	7.7814	0.0007513	0.0295285	-
LOC_Os01g48610	14.2167	4.1900	-1.7626	0.0007552	0.0296391	-
LOC_Os01g71710	6.5833	2.2233	-1.5661	0.0007731	0.0302549	BAT1
LOC_Os03g06700	0.1833	0.0010	-7.5183	0.000777	0.0303642	KCS11
LOC_Os12g33140	1.2467	0.1233	-3.3374	0.0007917	0.0308386	-
LOC_Os11g11960	55.0600	12.6200	-2.1253	0.0008053	0.031213	RPM1
LOC_Os07g33700	0.5733	0.0800	-2.8413	0.0008057	0.031213	At1g48120
LOC_Os01g19610	5.7333	2.2267	-1.3645	0.0008083	0.0312584	At3g47200

LOC_Os06g41980	2.9000	0.5900	-2.2973	0.0008091	0.0312584	LYK5
LOC_Os04g39360	3.8867	0.3433	-3.5009	0.0008141	0.0314041	-
LOC_Os03g11600	1.0600	0.1700	-2.6405	0.0008181	0.0314698	DL
LOC_Os02g17710	3.3667	0.9700	-1.7953	0.0008225	0.0315025	At1g35710
LOC_Os03g45450	39.4567	10.4667	-1.9145	0.0008316	0.0318089	WRKY71
LOC_Os06g38830	6.0800	1.2967	-2.2293	0.0008344	0.0318692	At3g47570
LOC_Os12g39120	4.0567	1.3867	-1.5487	0.0008431	0.0321577	Os12g0580900
LOC_Os07g42610	2.1900	5.5633	1.3450	0.0008523	0.0324169	ATL3
LOC_Os07g30600	7.0233	0.1333	-5.7190	0.0008586	0.0325701	FAR1
LOC_Os10g03570	3.4000	1.1300	-1.5892	0.0008735	0.0330377	RPM1
LOC_Os07g25460	43.0267	93.8833	1.1256	0.0008763	0.0330962	Ankrd13b
LOC_Os12g37560	4.7467	1.4067	-1.7546	0.0008799	0.0331847	PLC6
LOC_Os07g03600	1.6500	0.1133	-3.8638	0.0008812	0.033188	-
LOC_Os11g01390	0.6700	0.0010	-9.3880	0.0008991	0.0337754	CML25
LOC_Os11g45400	2.2333	0.7567	-1.5615	0.0009041	0.0339106	GPAT3
LOC_Os05g35690	1.6000	0.2700	-2.5670	0.0009055	0.0339143	SN2
LOC_Os01g06140	0.1833	0.0010	-7.5183	0.0009099	0.0340308	-
LOC_Os07g45074	2.6000	14.6600	2.4953	0.0009111	0.0340308	TRA1
LOC_Os02g27310	14.5500	4.5667	-1.6718	0.0009171	0.0341584	CRK41
LOC_Os07g23430	1.0433	0.1267	-3.0421	0.0009289	0.03446	FAD2-2
LOC_Os08g04370	0.8500	0.0200	-5.4094	0.000929	0.03446	-
LOC_Os02g34800	0.5500	0.0010	-9.1033	0.0009345	0.0346178	At4g08850
LOC_Os03g46424	11.0167	22.1400	1.0070	0.0009413	0.0347699	-
LOC_Os06g45970	0.7867	0.0010	-9.6196	0.0009437	0.0347699	SAUR71
LOC_Os10g28080	74.7967	22.6000	-1.7267	0.0009438	0.0347699	-
LOC_Os11g34880	1.3200	0.6067	-1.1216	0.0009456	0.0347903	RPM1
LOC_Os11g29720	5.4867	1.5067	-1.8646	0.00095	0.0348581	CYP78A5
LOC_Os03g43770	2.9900	0.8000	-1.9021	0.0009523	0.034893	At3g48880
LOC_Os06g04070	62.5467	23.8233	-1.3926	0.0009541	0.0349116	ADC1
LOC_Os06g10920	0.3067	0.0067	-5.5236	0.000956	0.0349343	FT1
LOC_Os01g13560	0.3400	1.5300	2.1699	0.0009592	0.0350056	Os01g0237000
LOC_Os01g70860	0.9267	0.1700	-2.4465	0.0009627	0.035087	PIR7A
LOC_Os01g56240	13.7767	3.1067	-2.1488	0.0009749	0.0353793	SAUR41
LOC_Os11g14480	0.9933	0.0733	-3.7597	0.000976	0.0353793	ASB11
LOC_Os01g51020	4.4200	1.6900	-1.3870	0.0009774	0.0353837	RPL28A
LOC_Os06g03180	1.7733	0.4133	-2.1011	0.0009807	0.035456	-
LOC_Os09g39060	3.4033	1.2333	-1.4644	0.0009884	0.0356484	SEN102
LOC_Os05g41370	2.1367	0.4400	-2.2798	0.0009887	0.0356484	CRK25
LOC_Os02g33670	1.6267	0.3033	-2.4229	0.0009936	0.035767	-
LOC_Os07g01410	0.1933	1.3733	2.8285	0.0009968	0.0357988	PRX74
LOC_Os07g35630	0.6867	0.0833	-3.0426	0.0010009	0.035897	CRK21
LOC_Os03g03730	20.6133	9.0500	-1.1876	0.0010055	0.0360042	agtA
LOC_Os10g37920	0.6667	0.0100	-6.0589	0.0010066	0.0360042	DTXL2

LOC_Os05g24660	3.8500	0.2633	-3.8699	0.0010091	0.0360487	IPT3
LOC_Os10g37680	1.5767	0.5300	-1.5728	0.001016	0.0361313	-
LOC_Os06g49760	2.8133	0.4367	-2.6877	0.0010164	0.0361313	-
MSTRG.4545	4.3333	1.1000	-1.9780	0.0010174	0.0361313	--
LOC_Os06g37150	7.3767	1.8300	-2.0111	0.0010181	0.0361313	-
LOC_Os01g45470	351.4367	117.4233	-1.5815	0.0010264	0.0363787	-
LOC_Os12g05080	2.5933	0.8700	-1.5757	0.0010316	0.0365142	TBL11
LOC_Os03g58820	6.9933	14.4467	1.0467	0.0010424	0.0367061	-
LOC_Os06g36380	6.3433	17.4800	1.4624	0.0010441	0.0367061	-
LOC_Os09g35780	0.9600	0.0833	-3.5261	0.0010446	0.0367061	BAP2
LOC_Os01g26300	0.6867	0.0900	-2.9316	0.0010457	0.0367061	WAK5
LOC_Os07g24000	0.0233	0.6100	4.7083	0.0010462	0.0367061	-
LOC_Os02g07830	3.0467	1.1800	-1.3684	0.0010474	0.0367061	HKT6
LOC_Os01g66680	1.1600	3.0700	1.4041	0.0010479	0.0367061	At2g19130
LOC_Os06g50950	12.9200	3.5733	-1.8543	0.0010517	0.0367934	At5g55050
LOC_Os03g27590	8.5200	3.1200	-1.4493	0.001057	0.0369284	SCPL51
LOC_Os03g50220	1.3300	0.4067	-1.7095	0.0010664	0.0372088	HOP2
LOC_Os08g16790	4.1600	1.9867	-1.0662	0.0010681	0.0372204	MYOB7
LOC_Os03g11890	14.8000	30.8533	1.0598	0.0010728	0.0373361	OBE4
LOC_Os07g27670	7.1533	2.5300	-1.4995	0.0010757	0.037388	WRKY41
LOC_Os10g23900	0.2067	0.0010	-7.6912	0.0010773	0.0373987	TYDC2
LOC_Os01g16714	4.2333	0.8667	-2.2882	0.0010898	0.0377829	YUC10
LOC_Os12g39360	55.7300	19.7000	-1.5003	0.0010931	0.0377947	nep1
LOC_Os08g44220	1.4867	0.1367	-3.4433	0.0010954	0.0377947	At4g39670
LOC_Os04g47360	1.0533	0.2433	-2.1140	0.0010959	0.0377947	Prep
LOC_Os10g34920	5.0267	1.5567	-1.6911	0.0010971	0.0377947	-
LOC_Os08g42910	32.9133	11.9433	-1.4625	0.0010971	0.0377947	MAP2A
LOC_Os04g49950	1.4400	0.2867	-2.3286	0.0011036	0.0379698	At2g43260
LOC_Os06g05450	20.3000	9.4167	-1.1082	0.00111	0.0381413	-
LOC_Os06g47470	5.4933	2.0833	-1.3988	0.0011274	0.0386899	CRK5
LOC_Os01g06790	2.5800	1.0600	-1.2833	0.0011299	0.0387257	RLP12
LOC_Os07g46060	2.9600	0.9533	-1.6345	0.0011433	0.0391121	-
LOC_Os10g04490	2.5833	0.6167	-2.0667	0.0011441	0.0391121	RGA2
LOC_Os06g51070	40.9067	16.0000	-1.3543	0.0011518	0.039326	NAC68
LOC_Os03g55010	22.8867	8.4367	-1.4398	0.0011533	0.0393286	UGT83A1
LOC_Os06g49970	1.5300	3.3933	1.1492	0.0011576	0.0393927	AMY2A
LOC_Os05g35050	0.0010	0.3000	8.2288	0.0011629	0.0395065	COPT2
LOC_Os04g41870	20.9367	10.1167	-1.0493	0.0011651	0.0395324	-
LOC_Os02g42710	0.8567	0.1800	-2.2507	0.0011737	0.0397736	ERG3
LOC_Os04g30110	6.8567	1.0633	-2.6889	0.0011771	0.0398399	WAK2
LOC_Os08g26520	0.9733	0.1567	-2.6352	0.0011801	0.0398645	-
LOC_Os05g05510	0.6133	0.0633	-3.2756	0.0011808	0.0398645	-
LOC_Os09g08990	0.4567	0.0867	-2.3976	0.0011916	0.0401275	CYP75B1

LOC_Os06g34960	2.5800	0.6100	-2.0805	0.0011999	0.0402577	At1g67520
LOC_Os01g41820	12.8433	5.0133	-1.3572	0.0012002	0.0402577	CYP72A15
LOC_Os04g09840	0.5767	1.6167	1.4872	0.0012014	0.0402577	TY3B-I
LOC_Os03g36130	0.1967	1.2200	2.6331	0.0012105	0.0405139	-
LOC_Os01g71350	33.2467	9.5033	-1.8067	0.0012207	0.0407262	-
LOC_Os12g36860	6.9867	0.9867	-2.8240	0.0012214	0.0407262	BETV1G
LOC_Os01g71340	118.9300	34.8067	-1.7727	0.0012274	0.0408686	-
LOC_Os04g55980	2.7367	0.1033	-4.7270	0.0012301	0.0408686	-
LOC_Os02g43690	23.8967	10.5533	-1.1791	0.001233	0.0409117	YPTM2
LOC_Os05g05680	91.7567	24.8633	-1.8838	0.0012357	0.04095	MAO1B
LOC_Os03g25450	4.5100	2.1167	-1.0913	0.0012447	0.0411986	CBF5
LOC_Os01g36294	10.4167	3.1700	-1.7163	0.0012473	0.0411987	CYP71C1
LOC_Os12g17430	2.4667	0.3300	-2.9020	0.0012484	0.0411987	RPM1
LOC_Os03g21730	0.1133	0.3800	1.7454	0.0012508	0.0411987	IRK
LOC_Os06g37080	4.7933	1.5333	-1.6444	0.001252	0.0411987	-
LOC_Os01g12680	0.4300	0.0667	-2.6893	0.0012523	0.0411987	SLAH3
LOC_Os05g13620	1.4667	0.2633	-2.4776	0.0012689	0.0416901	-
LOC_Os01g20980	0.8433	0.0467	-4.1756	0.0012703	0.0416901	PME41
LOC_Os02g37280	10.6033	2.7733	-1.9348	0.0013039	0.0426096	-
MSTRG.17201	7.5467	21.0967	1.4831	0.0013044	0.0426096	--
LOC_Os07g05420	3.6167	0.7100	-2.3488	0.0013306	0.0432997	BZ1
MSTRG.9462	0.6600	0.0010	-9.3663	0.0013448	0.0435536	--
LOC_Os04g54200	18.7100	5.0233	-1.8971	0.0013532	0.0437196	DGK5
LOC_Os12g08850	0.2833	0.0233	-3.6020	0.0013608	0.0439129	csd
LOC_Os08g31340	1.1000	0.3533	-1.6384	0.0013646	0.0439828	CCH
LOC_Os01g58240	27.5933	5.7967	-2.2510	0.001381	0.0444279	SBT3.5
LOC_Os03g36140	0.0010	0.2267	7.8244	0.0013817	0.0444279	-
LOC_Os05g49810	1.8433	0.5233	-1.8165	0.0013978	0.0448406	-
LOC_Os06g50930	16.4233	35.1500	1.0978	0.0014063	0.0450578	STR15
LOC_Os03g05530	17.3933	3.8767	-2.1656	0.0014151	0.0452874	At2g39510
LOC_Os07g25810	0.9467	2.6567	1.4887	0.0014295	0.0456484	Sbsn
LOC_Os01g49150	0.4000	0.0967	-2.0489	0.0014367	0.045763	ASF1A
LOC_Os06g11860	40.1833	17.2633	-1.2189	0.0014379	0.045763	RAP2-4
LOC_Os04g34290	6.8267	3.1000	-1.1389	0.0014384	0.045763	At2g19130
LOC_Os06g35560	32.7267	10.2867	-1.6697	0.0014405	0.0457734	CBDAS3
LOC_Os10g26940	11.0467	63.6200	2.5259	0.0014597	0.0463313	BURP16
LOC_Os05g41790	2.3500	5.0867	1.1141	0.0014721	0.0466682	Os05g0497500
LOC_Os12g27220	40.8900	9.4833	-2.1083	0.0014809	0.0468616	HSR201
LOC_Os07g48020	143.6500	47.7833	-1.5880	0.0014831	0.0468616	PRX112
LOC_Os05g03934	24.2267	10.7167	-1.1767	0.0014834	0.0468616	-
LOC_Os04g28780	1.4567	0.2467	-2.5620	0.0014985	0.047284	At2g19130
LOC_Os08g37300	2.9333	10.1833	1.7956	0.0015016	0.0473251	-
LOC_Os07g13810	4.4033	8.9733	1.0270	0.0015036	0.0473352	Bx8

LOC_Os01g58290	6.8700	2.0933	-1.7145	0.0015112	0.0474887	SBT3.5
LOC_Os07g09190	20.6833	5.6500	-1.8721	0.001512	0.0474887	Os07g0190000
LOC_Os09g21340	29.1600	59.7000	1.0337	0.0015198	0.0476768	NAT6
LOC_Os06g48240	1.1233	0.3833	-1.5511	0.0015422	0.0481572	bcsl1b
LOC_Os02g26790	23.7800	9.3933	-1.3400	0.0015454	0.0482012	-
LOC_Os05g35010	2.8867	5.8900	1.0289	0.0015529	0.0483354	CYP71A1
LOC_Os03g09880	19.2633	4.9900	-1.9487	0.0015605	0.0484485	At5g48750
LOC_Os01g41160	0.4100	0.0133	-4.9425	0.0015639	0.048492	FOXRED1
LOC_Os04g28390	3.0367	0.0633	-5.5834	0.0015655	0.048492	-
LOC_Os01g37460	5.1833	1.3267	-1.9661	0.0015673	0.048492	VAR3
LOC_Os12g30610	4.0067	8.1500	1.0244	0.0015695	0.048507	-
LOC_Os03g32314	84.7900	37.9800	-1.1587	0.0015887	0.0488965	AOC3
LOC_Os01g43480	30.0267	10.5900	-1.5035	0.0015923	0.0488965	atad1a
LOC_Os12g02440	16.5867	8.0633	-1.0406	0.0015991	0.0489722	WRKY46
LOC_Os11g07270	0.7033	1.9300	1.4563	0.001604	0.0490667	At3g47570
LOC_Os01g72900	20.8767	5.1133	-2.0296	0.0016113	0.0491947	ASR1
LOC_Os02g42440	16.0033	6.6233	-1.2727	0.0016118	0.0491947	At5g08350
LOC_Os06g29790	4.8400	1.4733	-1.7159	0.0016142	0.0492122	PHO1-3
LOC_Os04g12580	8.0700	2.3700	-1.7677	0.0016264	0.0495269	RLK1
LOC_Os12g42610	9.1733	4.0100	-1.1938	0.0016313	0.0496211	YAB6
LOC_Os06g40240	0.0467	0.0010	-5.5443	0.001639	0.0497555	Os03g0733400
LOC_Os07g03279	1.4067	0.1167	-3.5918	0.0016409	0.0497555	-
LOC_Os12g24650	7.4867	1.9467	-1.9433	0.0016478	0.0498998	Os02g0794700

Table S6. DEGs involved in hormone signal transduction in WR04-6 and SN9816 plants under ABA or diniconazole treatment.

		log2(FC)			
**Hormone**	**Gene ID**	**WR_A**	**WR_D**	**SN_A**	**SN_D**
ABA	LOC_Os05g49730(Os05g0572700)	3.139	0	0	0
	LOC_Os03g27280(SAPK1)	1.064	0	0	0
	LOC_Os01g64000(ABI5)	5.867	0	0	0
	LOC_Os05g39580(PYL5)	0	0	0	-3.787
IAA	LOC_Os05g37470(Os05g0447200)	-1.335	-1.145	0	-1.295
	LOC_Os03g43410(IAA12)	-1.404	-2.793	0	0
	LOC_Os11g06820(LAX2)	0	-4.044	0	0
	LOC_Os12g40900(IAA31)	0	-2.165	0	0
	LOC_Os12g40890(IAA30)	0	-2.109	0	0
	LOC_Os03g53150(IAA13)	0	-1.2	0	0
	LOC_Os10g36703(SAUR32)	0	0	0	-2.339
ETH	LOC_Os08g39830(EIL3)	0	0	3.36	0
	LOC_Os07g22730(ERF1B)	0	5.008	0	0
	LOC_Os09g39850(ERF1B)	0	2.338	0	0
GA	LOC_Os12g41650(PIF1)	1.323	1.276	0	1.066
	LOC_Os01g45860(GAI)	0	-1.297	0	0
CK	LOC_Os11g04720(ARR9)	-2.591	0	0	0
	LOC_Os02g55320(ARR12)	0	1.048	0	0
SA	LOC_Os01g28450(-)	-3.483	0	-4.795	-4.388
	LOC_Os01g28500(PRMS)	1.052	1.003	2.157	0
	LOC_Os10g11500(-)	4.741	0	0	0
	LOC_Os11g04600(NPR6)	0	-3.125	0	-3.292
	LOC_Os12g04410(NPR6)	0	-2.325	0	0
	LOC_Os01g64020(-)	0	1.143	0	0
	LOC_Os08g07970(TGA4)	0	1.06	0	0
	LOC_Os01g59350(-)	0	1.429	0	0
	LOC_Os07g03458(-)	0	-5.019	0	-3.748
	LOC_Os07g03279(-)	0	-4.969	0	-3.591
	LOC_Os07g03368(-)	0	-4.892	0	-3.657
	LOC_Os07g03730(-)	0	-3.041	-4.613	-5.687
	LOC_Os06g41100(-)	0	0	-1.867	0
	LOC_Os07g03740(-)	0	0	-3.665	-4.233
	LOC_Os07g03600(-)	0	0	0	-3.863
	LOC_Os07g03710(-)	0	0	0	-2.955
JA	LOC_Os04g32480(TIFY9)	0	-2.814	0	-2.712
	LOC_Os09g26780(TIFY10A)	0	-1.869	0	0
	LOC_Os07g42370(TIFY10A)	0	-1.55	0	0
	LOC_Os08g33160(TIFY6B)	0	-1.217	0	0

LOC_Os04g55920(TIFY3B)	0	-1.135	0	0

Table S7. Comparative analysis of defense-related genes in response to ABA or diniconazole treatment.

Gene ID	log2(FC)			
	WR_A	WR_D	SN_A	SN_D
**plant-pathogens interaction pathway**				
LOC_Os01g18240(MYB86)	0	-2.801	0	0
LOC_Os01g28500(PRMS)	1.052	1.003	2.157	0
LOC_Os01g36460(MYB2)	0	-2.86	0	0
LOC_Os01g50720(MYB2)	0	-3.179	0	-2.21
LOC_Os02g30190(RIN4)	-3.22	-2.72	0	0
LOC_Os03g06700(KCS11)	0	-7.78	0	-3.373
LOC_Os03g06705(KCS11)	0	-9.212	0	-5.149
LOC_Os03g25550(MYB86)	0	-4.42	0	0
LOC_Os03g26530(KCS12)	0	-1.23	0	0
LOC_Os03g57450(CPK1)	0	-1.519	0	0
LOC_Os04g02640(CUT1)	0	-4.617	0	0
LOC_Os04g50770(-)	0	-2.776	0	0
LOC_Os05g04820(MYB86)	0	-1.85	0	0
LOC_Os07g03279(-)	0	-4.969	0	-3.591
LOC_Os07g03368(-)	0	-4.892	0	-3.657
LOC_Os07g03458(-)	0	-5.109	0	-3.748
LOC_Os07g03730(-	0	-3.041	-4.613	-5.68
LOC_Os07g44090(MYB86)	0	-2.379	0	0
LOC_Os07g48770(dfr1)	0	-2.658	0	0
LOC_Os08g33150(ODO1)	0	-4.18	0	-3.049
LOC_Os08g38990(WRKY2)	0	1.61	0	1.496
LOC_Os09g09490(RPM1)	0	-1.454	0	-1.584
LOC_Os09g23620(ODO1)	0	-2.301	0	0
LOC_Os09g26660(RBOHE)	0	-1.243	0	0
LOC_Os09g36250(ODO1)	0	-1.346	0	0
LOC_Os10g39420(SPK)	0	-1.502	0	0
LOC_Os11g03440(MYB306)	0	-3.103	0	0
LOC_Os11g11960(RPM1)	-2.61	-2.175	0	-2.125
LOC_Os11g12040(RPM1)	0	-2.465	0	0
LOC_Os11g12300(RPM1)	-2.742	-3.465	0	0
LOC_Os11g12320(RPM1)	0	-2.86	-2.401	0
LOC_Os11g35500(At3g47570)	0	-1.804	0	0
LOC_Os12g03150(MYB306)	0	-5.186	0	0
LOC_Os12g37690(MYB108)	3.136	1.582	0	0
LOC_Os12g38400(RS2)	0	-2.292	0	0
LOC_Os01g03720(MYB108)	2.976	0	0	0
LOC_Os01g28450(-)	-3.483	0	-4.395	-4.388

LOC_Os01g32120(CML11)	1.608	0	1.52	0
LOC_Os02g56860(KCS12)	3.15	0	0	0
LOC_Os04g01740(HSP83A)	2.972	0	0	0
LOC_Os10g11500(-);	4.741	0	0	0
LOC_Os10g33370(KCS12)	4.127	0	0	0
LOC_Os12g07610(MYB98)	-3.43	0	0	0
LOC_Os12g32986(-)	1.152	0	0	0
LOC_Os02g49920(KCS5)	0	0	2.306	0
LOC_Os07g03740(-)	0	0	-3.665	-4.233
LOC_Os11g12240(RPM1)	0	0	-3.83	0
LOC_Os11g12260(RPM1)	0	0	-3.49	0
LOC_Os11g12340(RPM1)	-3.369	-2.594	0	0
LOC_Os01g72680(RPS5)	0	0	0	-7.228
LOC_Os03g59770(-)	0	0	0	-2.412
LOC_Os05g48010(RAX3)	0	0	0	-1.808
LOC_Os06g10580(CNGC20)	0	0	0	-1.268
LOC_Os07g03600(-)	0	0	0	-3.863
LOC_Os07g03710(-)	0	0	0	-2.955
LOC_Os11g01390(CML25)	0	0	0	-9.388
LOC_Os11g04560(CML36)	0	0	0	-2.097
LOC_Os12g01400(CML25)	0	0	0	-9.112
LOC_Os12g04360(CML36)	0	0	0	-2.299
**MAPK signaling pathway**				
LOC_Os01g28500(PRMS)	1.052	1.003	2.157	0
LOC_Os02g10290(HMA5)	1.059	1.081	0	0
LOC_Os02g21700(ANP2)	0	-2.169	0	0
LOC_Os03g04060(Cht11)	0	-2.231	0	-2.428
LOC_Os03g12390(MKK7)	0	-1.018	0	0
LOC_Os04g46940(HMA5)	0	-1.325	0	0
LOC_Os05g33130(Cht2)	4.188	5.946	0	0
LOC_Os05g33140(Cht9)	0	-1.828	0	0
LOC_Os07g03279(-)	0	-4.969	0	-3.591
LOC_Os07g03368(-)	0	-4.892	0	-3.657
LOC_Os07g03458(-)	0	-5.109	0	-3.748
LOC_Os07g03730(-)	0	-3.041	-4.613	-5.68
LOC_Os07g22730(ERF1B)	0	5.008	0	0
LOC_Os07g48770(dfr1)	0	-2.658	0	0
LOC_Os09g26660(RBOHE)	0	-1.243	0	0
LOC_Os09g39850(ERF1B)	0	2.338	0	0
LOC_Os01g28450(-)	-3.483	0	-4.395	-4.388
LOC_Os03g27280(SAPK1)	1.064	0	0	0
LOC_Os05g49730(Os05g0572700)	3.139	0	0	0
LOC_Os06g51050(Cht3)	3.948	0	0	0

LOC_Os06g51060(Cht1)	3.334	0	0	0
LOC_Os07g03740(-)	0	0	-3.665	-4.233
LOC_Os08g39830(EIL3)	0	0	3.363	0
LOC_Os10g11500(-)	4.741	0	0	0

Table S8. DEGs of transcription factors (TFs) expressed differentially in the four pairwise comparisons of transcriptomes.

Gene ID	Symbol	log2(FC)	PValue	FDR
**WR_CK vs. WR_A**				
LOC_Os02g13800	HSFC2A	3.376836436	8.12E-14	9E-11
LOC_Os06g09660	ARF16	1.820914999	8.62E-12	5.43E-09
LOC_Os04g45810	HOX22	2.412027539	5.27E-11	2.81E-08
LOC_Os12g03040	ONAC010	2.706803624	1.79E-10	8.28E-08
LOC_Os03g21710	WRKY70	-5.044394119	6.84E-10	2.67E-07
LOC_Os11g02480	WRKY55	1.510148503	1.26E-09	4.31E-07
LOC_Os12g37690	MYB108	3.136150121	1.32E-09	4.42E-07
LOC_Os11g03300	ONAC010	2.22881869	7.81E-09	2.06E-06
LOC_Os01g64000	ABI5	5.867896464	1.52E-08	3.8E-06
LOC_Os05g46370	BHLH87	2.125434235	6.38E-08	1.28E-05
LOC_Os02g46030	RVE1	2.527598926	4.43E-07	6.67E-05
LOC_Os10g39130	MADS56	-3.032860187	4.6E-07	6.88E-05
LOC_Os07g48550	NAC100	1.903400277	4.62E-07	6.88E-05
LOC_Os10g42490	ROC3	-1.647118977	5.32E-06	0.000508
LOC_Os02g43820	ERF5	2.042591877	1.59E-05	0.001155
LOC_Os04g31290	FIT	4.640893776	3.55E-05	0.002244
LOC_Os08g43334	HSFB2B	2.335155254	3.74E-05	0.002322
LOC_Os03g42280	Os03g062040(	-1.9274875	5.18E-05	0.003037
LOC_Os02g16680	BZIP9	1.072813285	5.63E-05	0.003259
LOC_Os01g57580	BHLH123	-1.29161247	5.74E-05	0.003316
LOC_Os08g19590	ROC4	-1.023513595	7.13E-05	0.003913
LOC_Os05g10670	Os05g019520(	2.023744354	8.02E-05	0.004265
LOC_Os01g15900	CDF2	2.662907662	8.16E-05	0.004318
LOC_Os06g46270	NAC021	1.545532356	0.000108	0.005337
LOC_Os04g28580	FRS5	7.736965594	0.000112	0.005498
LOC_Os06g30830	AGL61	2.703188582	0.000162	0.00738
LOC_Os06g40150	WIN1	4.925095191	0.000166	0.0075
LOC_Os03g45450	WRKY71	-2.448145811	0.000198	0.00867
LOC_Os03g20910	WOX6	7.824428435	0.000212	0.009111
LOC_Os01g03720	MYB108	2.976747269	0.00022	0.009285
LOC_Os01g39020	HSFA6B	2.374635145	0.00032	0.012409
LOC_Os02g36880	NAC100	1.810660755	0.000339	0.012978
LOC_Os12g02420	WRKY55	1.105865864	0.000341	0.01302
LOC_Os01g68370	VP1	6.781359714	0.000347	0.013154
LOC_Os06g36680	ATH1	-4.087462841	0.000356	0.013455
LOC_Os04g32620	ERF113	1.88373752	0.000364	0.013656
LOC_Os01g06640	BHLH51	1.59657713	0.000486	0.017219
LOC_Os03g17810	LBD1	2.829584869	0.000604	0.020216

LOC_Os08g42470	BHLH137	4.505528033	0.000611	0.020446
LOC_Os02g43330	HOX24	3.612774351	0.000666	0.021914
LOC_Os03g63810	WRKY35	1.690671942	0.000668	0.02196
LOC_Os06g51260	RVE2	3.304957815	0.00072	0.023203
LOC_Os12g07610	MYB98	-3.430350555	0.000743	0.023596
LOC_Os12g02470	WRKY53	-4.700439718	0.000989	0.029223
LOC_Os02g43170	BBX21	-1.685583741	0.00099	0.029223
LOC_Os12g06010	-	5.196397213	0.001001	0.029298
LOC_Os04g48070	ROC4	1.472997966	0.0011	0.031386
LOC_Os02g53620	NFYA5	-1.640815735	0.0012	0.033038
LOC_Os12g41650	PIF1	1.323306905	0.001363	0.036377
LOC_Os07g48260	WRKY54	-1.717094391	0.001441	0.037719
LOC_Os02g07170	KAN2	2.642447995	0.001548	0.039783
LOC_Os09g25070	WRKY40	-3.561007769	0.001709	0.042612
LOC_Os01g71790	NAC018	-1.82687561	0.001788	0.044031
LOC_Os04g47080	LC	2.611434712	0.001833	0.044627
LOC_Os01g53650	Os01g073840(	8.22881869	0.00199	0.047375
LOC_Os02g34630	TERF1	1.163	0.00204	0.048269
**WR_CK vs. WR_D**				
LOC_Os01g06640	BHLH51	2.215115406	6.79E-19	1.6E-15
LOC_Os08g33150	ODO1	-4.180164186	7.16E-14	6.29E-11
LOC_Os11g07460	PCF3	-2.5980525	3.41E-13	2.25E-10
LOC_Os01g18240	MYB86	-2.801381021	7E-12	3.05E-09
LOC_Os03g03164	HOS66	-2.722225796	5.69E-10	1.48E-07
LOC_Os07g44090	MYB86	-2.379704426	1E-09	2.27E-07
LOC_Os06g04090	NAC043	-3.402212284	3.71E-09	6.7E-07
LOC_Os01g41900	DIVARICAT/	-2.196055244	3.72E-09	6.7E-07
LOC_Os05g49240	RL3	-6.266786541	7.23E-09	1.2E-06
LOC_Os12g03150	MYB306	-5.186160104	2.56E-08	3.33E-06
LOC_Os12g03040	ONAC010	2.487793842	2.63E-08	3.4E-06
LOC_Os05g48850	NAC073	-2.105957175	3.96E-08	4.88E-06
LOC_Os01g45730	Os01g064500(	-4.165931932	4.52E-08	5.46E-06
LOC_Os01g11550	PCF5	-2.981852653	4.75E-08	5.7E-06
LOC_Os04g50770	-	-2.776138289	1.21E-07	1.28E-05
LOC_Os04g56990	PHL1	1.692445545	4.01E-07	3.45E-05
LOC_Os10g39130	MADS56	-1.325954598	4.38E-07	3.69E-05
LOC_Os11g03300	ONAC010	2.178487015	6.17E-07	5.06E-05
LOC_Os09g25070	WRKY40	-3.933484806	8.6E-07	6.65E-05
LOC_Os05g47650	Os05g054980(	1.675142116	1.1E-06	8.14E-05
LOC_Os02g13800	HSFC2A	1.725728622	1.19E-06	8.64E-05
LOC_Os03g21710	WRKY70	-3.158565139	1.23E-06	8.86E-05
LOC_Os02g43170	BBX21	-1.050214985	1.52E-06	0.000105
LOC_Os01g01840	-	-3.262280112	2.5E-06	0.000159

LOC_Os01g39020	HSFA6B	2.146435472	3.03E-06	0.000187
LOC_Os01g50720	MYB2	-3.179056013	3.33E-06	0.000203
LOC_Os12g41060	EREBP1	-2.700439718	3.51E-06	0.000212
LOC_Os03g04310	BHLH93	-4.119739244	5.48E-06	0.000309
LOC_Os05g04820	MYB86	-1.853897508	5.95E-06	0.000332
LOC_Os03g25550	MYB86	-4.420662048	7.22E-06	0.000395
LOC_Os03g44710	YAB2	-1.552803833	8.26E-06	0.000443
LOC_Os12g38400	RS2	-2.292625959	8.56E-06	0.000455
LOC_Os12g41650	PIF1	1.276265312	1.34E-05	0.000668
LOC_Os01g71970	SCL3	1.025597896	1.35E-05	0.000675
LOC_Os08g37730	BHLH96	-2.121015401	0.000016	0.000768
LOC_Os11g02520	WRKY46	-3.552782095	1.71E-05	0.000808
LOC_Os05g10670	Os05g019520(	1.583342405	2.55E-05	0.001096
LOC_Os01g36460	MYB2	-2.860405693	2.84E-05	0.001181
LOC_Os07g02060	WRKY57	1.755461938	4.44E-05	0.001715
LOC_Os11g03440	MYB306	-3.103582507	4.56E-05	0.00175
LOC_Os09g26210	ZFP2	-1.275084867	4.92E-05	0.001844
LOC_Os09g36250	ODO1	-1.346097018	5.43E-05	0.001991
LOC_Os11g02540	WRKY70	-3.782851753	6.14E-05	0.002206
LOC_Os10g26500	HOX23	-2.260235772	7.13E-05	0.002481
LOC_Os05g28320	MYB44	-5.285402219	8.18E-05	0.002784
LOC_Os02g09830	BZIP43	2.818974005	9.01E-05	0.003018
LOC_Os03g11600	DL	-2.078569052	9.72E-05	0.003218
LOC_Os04g46350	HOX17	-1.170869483	0.0001	0.003299
LOC_Os06g30830	AGL61	2.317150955	0.000102	0.003349
LOC_Os11g48000	RBE	-1.87698911	0.000104	0.003391
LOC_Os10g39750	BHLH93	-2.732212064	0.000115	0.003671
LOC_Os09g23620	ODO1	-2.301169535	0.000128	0.004012
LOC_Os08g38990	WRKY2	1.610108567	0.00015	0.004517
LOC_Os02g41800	ARF8	-1.193451237	0.000151	0.00454
LOC_Os05g51160	DIVARICAT/	-1.774137145	0.000154	0.004589
LOC_Os10g41130	ERF034	-1.637429921	0.000165	0.004823
LOC_Os02g51280	TCP14	-1.614406279	0.000195	0.005533
LOC_Os02g50630	E2FE	-2.263034406	0.000213	0.005937
LOC_Os06g06900	SPT	-1.115889711	0.000221	0.006123
LOC_Os04g28280	BHLH62	-2.611929548	0.000246	0.006687
LOC_Os02g43970	ERF034	-1.861539465	0.000254	0.006838
LOC_Os01g53260	WRKY24	1.362108047	0.000262	0.007009
LOC_Os09g39850	ERF1B	2.338558769	0.000296	0.007717
LOC_Os01g59350	-	1.429364281	0.000317	0.008158
LOC_Os11g01550	LBD12	1.352789059	0.000335	0.008567
LOC_Os04g59380	-	-2.123382416	0.000339	0.008654
LOC_Os10g23090	HOX8	2.258447136	0.000355	0.008916

LOC_Os05g41240	PHL1	1.223772991	0.000397	0.009724
LOC_Os02g41450	NAC031	-8.896836931	0.000451	0.01075
LOC_Os11g05740	Os11g015600(	-4.075288127	0.000471	0.011078
LOC_Os08g09840	Os03g073340(	-1.505690633	0.000473	0.011104
LOC_Os07g22730	ERF1B	5.008988783	0.000536	0.012332
LOC_Os06g50830	FD	-2.567684509	0.000553	0.012656
LOC_Os12g41680	NAC021	1.003195884	0.000575	0.013074
LOC_Os06g35960	HSFC2B	1.369772766	0.000614	0.013815
LOC_Os06g36680	ATH1	-2.91753784	0.000689	0.015129
LOC_Os05g10620	NAC073	1.171980416	0.0007	0.015304
LOC_Os09g21180	HOX25	1.169925001	0.000702	0.015327
LOC_Os02g43330	HOX24	2.852378006	0.000703	0.015338
LOC_Os09g31300	BHLH130	1.252750927	0.000707	0.01539
LOC_Os01g64020	-	1.143667141	0.00072	0.015537
LOC_Os02g04640	PHL1	1.11300317	0.000728	0.015679
LOC_Os03g41330	LBD37	1.799179543	0.000754	0.016134
LOC_Os02g55320	ARR12	1.048322266	0.000802	0.016847
LOC_Os06g51070	NAC68	-1.436201396	0.000837	0.017385
LOC_Os01g39330	BHLH	-2.183518559	0.000858	0.017767
LOC_Os06g50600	FD	-1.98550043	0.00086	0.017799
LOC_Os04g43560	NAC054	-2.992466327	0.000864	0.017843
LOC_Os02g53620	NFYA5	-1.192934979	0.000885	0.018212
LOC_Os06g04870	HOX2	-1.524567903	0.000899	0.018457
LOC_Os01g21590	-	2.123828587	0.000905	0.018548
LOC_Os08g07970	TGA4	1.060120992	0.000907	0.01857
LOC_Os10g40810	GATA2	-1.332913136	0.000928	0.018921
LOC_Os01g45860	GAI	-1.297904866	0.001295	0.024568
LOC_Os12g01550	LBD12	1.289754524	0.001326	0.024946
LOC_Os01g24070	GATA23	-1.514830177	0.001552	0.028384
LOC_Os11g02530	WRKY70	-3.549629428	0.001678	0.030019
LOC_Os10g22600	RAP2-13	-1.834777618	0.001806	0.03173
LOC_Os05g25770	WRKY70	-2.190910857	0.001822	0.031935
LOC_Os04g33950	E2FB	-1.575502171	0.001822	0.031935
LOC_Os05g34830	NAC48	1.243303623	0.00191	0.032971
LOC_Os12g37690	MYB108	1.582125341	0.001964	0.0336
LOC_Os03g45450	WRKY71	-1.864901828	0.002134	0.03577
LOC_Os04g58020	DIVARICAT/	1.150879385	0.002253	0.037268
LOC_Os02g46560	BHLH96	-1.57699179	0.002273	0.037511
LOC_Os11g04960	NAC029	-1.076922817	0.002329	0.038136
LOC_Os02g46030	RVE1	1.413161677	0.002337	0.03825
LOC_Os12g02400	WRKY70	-2.007234866	0.002593	0.041249
LOC_Os03g10210	HOX12	1.925765044	0.002626	0.041626
LOC_Os06g50480	TRAB1	-3.861293729	0.002671	0.042137

LOC_Os11g02470	WRKY54	-2.292781749	0.003068	0.046912
**SN_CK vs. SN_A**				
LOC_Os10g39130	MADS56	-2.231398616	3.9E-08	3.49E-05
LOC_Os05g46370	BHLH87	2.430304256	2.27E-07	0.000164
LOC_Os07g48260	WRKY54	-2.549232714	2.31E-07	0.000164
LOC_Os06g04010	Os06g013060(	1.448175432	1.17E-06	0.00056
LOC_Os11g47630	ZAT8	-3.774670114	1.25E-06	0.000587
LOC_Os09g25070	WRKY40	-2.856051484	2.79E-06	0.001017
LOC_Os03g21710	WRKY70	-3.058691899	3.67E-06	0.001188
LOC_Os12g01490	KAN4	-2.920031695	5.98E-06	0.001692
LOC_Os01g09990	BHLH	-4.168811381	7.08E-06	0.001945
LOC_Os06g41100	-	-1.866997868	2.36E-05	0.005157
LOC_Os06g06750	MADS5	-8.74819285	3.62E-05	0.007128
LOC_Os05g29810	RAP2-3	2.647118977	7.62E-05	0.012475
LOC_Os03g47016	OSH10	-2.724597037	0.00014	0.018879
LOC_Os02g53100	WRKY9	-4.370457989	0.000148	0.019758
LOC_Os11g01480	KAN4	-2.378131517	0.000182	0.022987
LOC_Os02g55380	ERF003	2.570043081	0.000295	0.031629
LOC_Os08g39830	EIL3	3.360747344	0.000335	0.034718
LOC_Os02g49480	BIM1	-1.747085475	0.000389	0.038096
LOC_Os06g37410	BHLH	-3.359777356	0.000414	0.039492
LOC_Os08g41960	AGL3	2.563069895	0.000478	0.043459
**SN_CK vs. SN_D**				
LOC_Os03g21710	WRKY70	-3.329819987	2.81E-16	8.45E-13
LOC_Os11g47630	ZAT8	-5.620960699	1.18E-09	6.22E-07
LOC_Os02g53100	WRKY9	-6.296457407	3.44E-08	1.19E-05
LOC_Os08g33150	ODO1	-3.04977397	9.29E-08	2.79E-05
LOC_Os05g25770	WRKY70	-1.661978825	2.23E-07	5.79E-05
LOC_Os07g48260	WRKY54	-2.054559099	5.55E-07	0.000123
LOC_Os03g44710	YAB2	-1.506860017	6.33E-07	0.000137
LOC_Os01g40430	WRKY9	-10.36267444	1.09E-06	0.00021
LOC_Os05g45410	HSFA4D	-2.300275727	1.1E-06	0.00021
LOC_Os06g04010	Os06g013060(	1.467535228	2.59E-06	0.000425
LOC_Os06g41770	BZIP60	-1.214079675	2.85E-06	0.00045
LOC_Os11g02530	WRKY70	-3.106712336	3.35E-06	0.000507
LOC_Os02g49480	BIM1	-1.844905597	3.39E-06	0.000507
LOC_Os10g39750	BHLH93	-4.054447784	4.99E-06	0.000688
LOC_Os11g48000	RBE	-2.693376326	7.3E-06	0.000947
LOC_Os12g41650	PIF1	1.066479402	1.91E-05	0.002028
LOC_Os03g08460	ERF073	-2.6116944	2.2E-05	0.002287
LOC_Os07g04560	JUB1	-3.498250868	3.38E-05	0.003295
LOC_Os10g33760	NAC021	-2.529253068	3.6E-05	0.003453
LOC_Os11g05614	NAC090	-1.827340031	4.46E-05	0.004055

LOC_Os03g13400	IDD14	-7.94641896	5.87E-05	0.005009
LOC_Os01g09990	BHLH	-4.071949842	5.87E-05	0.005009
LOC_Os08g38990	WRKY2	1.49683159	6.88E-05	0.005554
LOC_Os01g53040	WRKY65	-2.114071767	8.6E-05	0.00658
LOC_Os05g40960	APL	-2.708469258	8.7E-05	0.006625
LOC_Os01g39330	BHLH	-1.748887498	0.000106	0.007591
LOC_Os11g07460	PCF3	-1.819084215	0.000113	0.007922
LOC_Os09g25070	WRKY40	-2.128060694	0.000144	0.009285
LOC_Os10g26500	HOX23	-2.835830091	0.00015	0.009525
LOC_Os10g42130	NAC086	-2.842125817	0.000157	0.009873
LOC_Os03g04310	BHLH93	-3.061111514	0.000162	0.010147
LOC_Os01g40260	WRKY50	-2.302514809	0.000162	0.010147
LOC_Os05g46370	BHLH87	2.226770862	0.000234	0.013302
LOC_Os05g11414	MADS58	2.591446534	0.000272	0.014965
LOC_Os11g02540	WRKY70	-2.65371164	0.000285	0.015423
LOC_Os03g47016	OSH10	-2.590295945	0.000312	0.016419
LOC_Os01g39020	HSFA6B	1.832958978	0.00038	0.01843
LOC_Os06g37410	BHLH	-3.078595603	0.00042	0.019706
LOC_Os05g48010	RAX3	-1.808844925	0.000423	0.019828
LOC_Os01g50720	MYB2	-2.210604562	0.000586	0.024985
LOC_Os09g10980	WIP5	-4.676460855	0.000645	0.026874
LOC_Os11g02520	WRKY46	-2.435495855	0.000722	0.028717
LOC_Os03g11600	DL	-2.640457613	0.000818	0.03147
LOC_Os03g45450	WRKY71	-1.914467023	0.000832	0.031809
LOC_Os07g27670	WRKY41	-1.499478285	0.001076	0.037388
LOC_Os06g51070	NAC68	-1.354264077	0.001152	0.039326
LOC_Os06g11860	RAP2-4	-1.218886187	0.001438	0.045763
LOC_Os05g41790	Os05g049750(	1.1140598	0.001472	0.046668
LOC_Os12g02440	WRKY46	-1.040575716	0.001599	0.048972
LOC_Os12g42610	YAB6	-1.193843828	0.001631	0.049621

Table S9. Differentially expressed genes (DEGs) had unique expression profiles in WR04-6 under ABA or diniconazole treatment.

	log2(FC)			
**Gene ID**	**WR_A**	**WR_D**	**SN_A**	**SN_D**
**ROS homeostasis**				
LOC_Os06g23780	8.3368784	0	0	0
LOC_Os07g46990	1.7223687	0	0	0
LOC_Os04g53210	2.5258242	1.5623235	0	0
LOC_Os09g32290	0	1.4517638	0	0
LOC_Os08g44360	0	1.3423922	0	0
LOC_Os01g49710	2.4372206	1.2755823	0	0
LOC_Os10g38470	0	1.4925566	0	0
LOC_Os06g12290	4.959697	2.9319816	0	0
LOC_Os11g29400	0	1.5604745	0	0
LOC_Os07g22350	0	1.100977	0	0
LOC_Os04g35520	0	1.3328043	0	0
LOC_Os10g38600	0	1.8781116	0	0
LOC_Os10g38340	0	2.2033206	0	0
LOC_Os10g38360	0	1.8499283	0	0
LOC_Os10g38610	2.186587	0	0	0
LOC_Os10g38740	2.0600643	1.7085711	0	0
LOC_Os02g28110	3.7458537	0	0	0
**Hormone signal transduction**				
LOC_Os05g49730	3.139	0	0	0
LOC_Os03g27280	1.064	0	0	0
LOC_Os01g64000	5.867	0	0	0
LOC_Os07g22730	0	5.008	0	0
LOC_Os09g39850	0	2.338	0	0
LOC_Os02g55320	0	1.048	0	0
LOC_Os10g11500	4.741	0	0	0
LOC_Os01g64020	0	1.143	0	0
LOC_Os08g07970	0	1.06	0	0
LOC_Os01g59350	0	1.429	0	0
**defense-related genes**				
LOC_Os01g18240	0	-2.801	0	0
LOC_Os01g36460	0	-2.86	0	0
LOC_Os02g30190	-3.22	-2.72	0	0
LOC_Os03g25550	0	-4.42	0	0
LOC_Os03g26530	0	-1.23	0	0
LOC_Os03g57450	0	-1.519	0	0
LOC_Os04g02640	0	-4.617	0	0
LOC_Os04g50770	0	-2.776	0	0

LOC_Os05g04820	0	-1.85	0	0
LOC_Os07g44090	0	-2.379	0	0
LOC_Os07g48770	0	-2.658	0	0
LOC_Os09g23620	0	-2.301	0	0
LOC_Os09g26660	0	-1.243	0	0
LOC_Os09g36250	0	-1.346	0	0
LOC_Os10g39420	0	-1.502	0	0
LOC_Os11g03440	0	-3.103	0	0
LOC_Os11g12040	0	-2.465	0	0
LOC_Os11g12300	-2.742	-3.465	0	0
LOC_Os11g35500	0	-1.804	0	0
LOC_Os12g03150	0	-5.186	0	0
LOC_Os12g37690	3.136	1.582	0	0
LOC_Os12g38400	0	-2.292	0	0
LOC_Os01g03720	2.976	0	0	0
LOC_Os02g56860	3.15	0	0	0
LOC_Os04g01740	2.972	0	0	0
LOC_Os10g11500	4.741	0	0	0
LOC_Os10g33370	4.127	0	0	0
LOC_Os12g07610	-3.43	0	0	0
LOC_Os12g32986	1.152	0	0	0
LOC_Os11g12340	-3.369	-2.594	0	0
**MAPK signaling pathway**				
LOC_Os02g10290	1.059	1.081	0	0
LOC_Os02g21700	0	-2.169	0	0
LOC_Os03g12390	0	-1.018	0	0
LOC_Os04g46940	0	-1.325	0	0
LOC_Os05g33130	4.188	5.946	0	0
LOC_Os05g33140	0	-1.828	0	0
LOC_Os07g22730	0	5.008	0	0
LOC_Os07g48770	0	-2.658	0	0
LOC_Os09g26660	0	-1.243	0	0
LOC_Os09g39850	0	2.338	0	0
LOC_Os03g27280	1.064	0	0	0
LOC_Os05g49730	3.139	0	0	0
LOC_Os06g51050	3.948	0	0	0
LOC_Os06g51060	3.334	0	0	0
LOC_Os10g11500	4.741	0	0	0
**Transcription factors**				
LOC_Os02g13800	3.3768	1.7257	0	0
LOC_Os06g09660	1.8209	0.0000	0	0
LOC_Os04g45810	2.4120	0.0000	0	0
LOC_Os12g03040	2.7068	2.4878	0	0

LOC_Os11g02480	1.5101	0.0000	0	0
LOC_Os12g37690	3.1362	1.5821	0	0
LOC_Os11g03300	2.2288	2.1785	0	0
LOC_Os01g64000	5.8679	0.0000	0	0
LOC_Os02g46030	2.5276	1.4132	0	0
LOC_Os07g48550	1.9034	0	0	0
LOC_Os10g42490	-1.6471	0	0	0
LOC_Os02g43820	2.0426	0	0	0
LOC_Os04g31290	4.6409	0	0	0
LOC_Os08g43334	2.3352	0	0	0
LOC_Os03g42280	-1.9275	0	0	0
LOC_Os02g16680	1.0728	0	0	0
LOC_Os01g57580	-1.2916	0	0	0
LOC_Os08g19590	-1.0235	0	0	0
LOC_Os05g10670	2.0237	1.5833	0	0
LOC_Os01g15900	2.6629	0	0	0
LOC_Os06g46270	1.5455	0	0	0
LOC_Os04g28580	7.7370	0	0	0
LOC_Os06g30830	2.7032	2.3172	0	0
LOC_Os06g40150	4.9251	0	0	0
LOC_Os03g20910	7.8244	0	0	0
LOC_Os01g03720	2.9767	0	0	0
LOC_Os02g36880	1.8107	0	0	0
LOC_Os12g02420	1.1059	0	0	0
LOC_Os01g68370	6.7814	0	0	0
LOC_Os06g36680	-4.0875	-2.9175	0	0
LOC_Os04g32620	1.8837	0	0	0
LOC_Os01g06640	1.5966	2.2151	0	0
LOC_Os03g17810	2.8296	0	0	0
LOC_Os08g42470	4.5055	0	0	0
LOC_Os02g43330	3.6128	2.8524	0	0
LOC_Os03g63810	1.6907	0	0	0
LOC_Os06g51260	3.3050	0	0	0
LOC_Os12g07610	-3.4304	0	0	0
LOC_Os12g02470	-4.7004	0	0	0
LOC_Os02g43170	-1.6856	-1.0502	0	0
LOC_Os12g06010	5.1964	0	0	0
LOC_Os04g48070	1.4730	0	0	0
LOC_Os02g53620	-1.6408	-1.1929	0	0
LOC_Os02g07170	2.6424	0	0	0
LOC_Os01g71790	-1.8269	0	0	0
LOC_Os04g47080	2.6114	0	0	0
LOC_Os01g53650	8.2288	0	0	0

LOC_Os02g34630	1.1630	0	0	0
LOC_Os01g18240	0	-2.801381	0	0
LOC_Os03g03164	0	-2.722226	0	0
LOC_Os07g44090	0	-2.379704	0	0
LOC_Os06g04090	0	-3.402212	0	0
LOC_Os01g41900	0	-2.196055	0	0
LOC_Os05g49240	0	-6.266787	0	0
LOC_Os12g03150	0	-5.18616	0	0
LOC_Os05g48850	0	-2.105957	0	0
LOC_Os01g45730	0	-4.165932	0	0
LOC_Os01g11550	0	-2.981853	0	0
LOC_Os04g50770	0	-2.776138	0	0
LOC_Os04g56990	0	1.6924455	0	0
LOC_Os05g47650	0	1.6751421	0	0
LOC_Os01g01840	0	-3.26228	0	0
LOC_Os12g41060	0	-2.70044	0	0
LOC_Os05g04820	0	-1.853898	0	0
LOC_Os03g25550	0	-4.420662	0	0
LOC_Os12g38400	0	-2.292626	0	0
LOC_Os01g71970	0	1.0255979	0	0
LOC_Os08g37730	0	-2.121015	0	0
LOC_Os01g36460	0	-2.860406	0	0
LOC_Os07g02060	0	1.7554619	0	0
LOC_Os11g03440	0	-3.103583	0	0
LOC_Os09g26210	0	-1.275085	0	0
LOC_Os09g36250	0	-1.346097	0	0
LOC_Os05g28320	0	-5.285402	0	0
LOC_Os02g09830	0	2.818974	0	0
LOC_Os04g46350	0	-1.170869	0	0
LOC_Os09g23620	0	-2.30117	0	0
LOC_Os02g41800	0	-1.193451	0	0
LOC_Os05g51160	0	-1.774137	0	0
LOC_Os10g41130	0	-1.63743	0	0
LOC_Os02g51280	0	-1.614406	0	0
LOC_Os02g50630	0	-2.263034	0	0
LOC_Os06g06900	0	-1.11589	0	0
LOC_Os04g28280	0	-2.61193	0	0
LOC_Os02g43970	0	-1.861539	0	0
LOC_Os01g53260	0	1.362108	0	0
LOC_Os09g39850	0	2.3385588	0	0
LOC_Os01g59350	0	1.4293643	0	0
LOC_Os11g01550	0	1.3527891	0	0
LOC_Os04g59380	0	-2.123382	0	0

LOC_Os10g23090	0	2.2584471	0	0
LOC_Os05g41240	0	1.223773	0	0
LOC_Os02g41450	0	-8.896837	0	0
LOC_Os11g05740	0	-4.075288	0	0
LOC_Os08g09840	0	-1.505691	0	0
LOC_Os07g22730	0	5.0089888	0	0
LOC_Os06g50830	0	-2.567685	0	0
LOC_Os12g41680	0	1.0031959	0	0
LOC_Os06g35960	0	1.3697728	0	0
LOC_Os05g10620	0	1.1719804	0	0
LOC_Os09g21180	0	1.169925	0	0
LOC_Os09g31300	0	1.2527509	0	0
LOC_Os01g64020	0	1.1436671	0	0
LOC_Os02g04640	0	1.1130032	0	0
LOC_Os03g41330	0	1.7991795	0	0
LOC_Os02g55320	0	1.0483223	0	0
LOC_Os06g50600	0	-1.9855	0	0
LOC_Os04g43560	0	-2.992466	0	0
LOC_Os06g04870	0	-1.524568	0	0
LOC_Os01g21590	0	2.1238286	0	0
LOC_Os08g07970	0	1.060121	0	0
LOC_Os10g40810	0	-1.332913	0	0
LOC_Os01g45860	0	-1.297905	0	0
LOC_Os12g01550	0	1.2897545	0	0
LOC_Os01g24070	0	-1.51483	0	0
LOC_Os10g22600	0	-1.834778	0	0
LOC_Os04g33950	0	-1.575502	0	0
LOC_Os05g34830	0	1.2433036	0	0
LOC_Os04g58020	0	1.1508794	0	0
LOC_Os02g46560	0	-1.576992	0	0
LOC_Os11g04960	0	-1.076923	0	0
LOC_Os12g02400	0	-2.007235	0	0
LOC_Os03g10210	0	1.925765	0	0
LOC_Os06g50480	0	-3.861294	0	0
LOC_Os11g02470	0	-2.292782	0	0
**Glycolysis and citrate (TCA) cycle**				
LOC_Os01g09460	2.541	1.767	0	0
LOC_Os08g25720	1.07	0	0	0
LOC_Os04g38540	0	2.234	0	0
LOC_Os01g46950	0	-1.367	0	0
LOC_Os02g38920	2.391	1.355	0	0
LOC_Os08g03290	1.108	0	0	0
LOC_Os06g45710	3.32	1.858	0	0

LOC_Os02g07260	1.238	0	0	0
LOC_Os03g60370	2.294	1.174	0	0
LOC_Os10g13700	0	3.017	0	0
LOC_Os01g47080	0	-1.248	0	0
LOC_Os11g10980	1.398	0	0	0
LOC_Os04g02900	0	1.149	0	0
LOC_Os06g13720	3.935	2.569	0	0
LOC_Os07g49250	0	-6.824	0	0
LOC_Os01g06660	1.894	1.543	0	0
LOC_Os05g39320	3.584	0	0	0
LOC_Os05g39310	8.956	0	0	0
LOC_Os11g10520	0	-5.448	0	0
LOC_Os11g10480	2.554	1.576	0	0
LOC_Os11g10510	6.476	2.556	0	0
LOC_Os02g43194	0	-3.829	0	0
LOC_Os12g07810	0	-3.147	0	0
LOC_Os04g45720	0	-2.732	0	0
LOC_Os02g43280	0	-1.918	0	0
LOC_Os09g26880	0	1.367	0	0
LOC_Os06g01630	1.05	0	0	0
LOC_Os03g19250	0	1.195	0	0
LOC_Os10g13700	0	3.017	0	0
LOC_Os06g01630	1.05	0	0	0
LOC_Os06g13720	3.935	2.569	0	0
LOC_Os04g02900	0	1.149	0	0
LOC_Os11g47120	0	-1.831	0	0
LOC_Os11g47330	0	-1.125	0	0
LOC_Os08g33720	0	-1.8	0	0
LOC_Os01g61380	0	1.994	0	0

Table S10. Gene-specific primers for RT-qPCR analysis.

id	Primer Sequence	
	**Forward (5'-3')**	**Reverse (5'-3')**
LOC_Os11g46900	CTACATCGATCCTGCCTACATC	AATACTTGGCAAAGCTTAACGG
LOC_Os08g26230	TTTCTAGGTGTTTGTCCTTCGA	GATGGAGTTGATCGATTCGGG
LOC_Os06g29730	TTAAGCTGCTCGTTTTCTGATG	TATGCAAGGATCACGTGGATTA
LOC_Os04g39300	TTATCCGATCTACGAACCGATC	GAAACACACTAAAGACGCACAT
LOC_Os01g68740	AGTGTCATCTCCTCCACAAATT	GTCAGTGGAGGAATTGGAGTAG
LOC_Os01g02780	CAGCTGGATGATGATACAAACG	TCGATTGAAGACAGATCAGACC
LOC_Os02g28340	AACACGTACGTCGAGTAGATTT	TGGATTCATCCCAGGTTTTGAT
LOC_Os05g39770	GTAATATGTCCTGCAAACCCAC	TTTCAACATTTACATCGCCTGG
LOC_Os03g25490	TCAAAAGGAAGTGGATACGGAA	TGTCCAGGTAAGAAGAAGTGAC
LOC_Os01g28500	TACAATGTTTCACTGCTCCGAT	CCAGAAGATGTTCTCACCGTAC
LOC_Os04g41620	TTGTGACAAGAGCAACAAACAG	GTTGAAGTTCCATGAGATCTGC
LOC_Os10g26940	GCACCTTCACATACGAACATAC	AACTCATTCGCTATATCTGGCA
OsActin	ACCATTGGTGCTGAGCGTTT	CGCAGCTTCCATTCCTATGAA
